# Whole-Genome Survey of the Putative ATP-Binding Cassette Transporter Family Genes in *Vitis vinifera*

**DOI:** 10.1371/journal.pone.0078860

**Published:** 2013-11-11

**Authors:** Birsen Çakır, Ozan Kılıçkaya

**Affiliations:** 1 Department of Horticulture, Faculty of Agriculture, Ege University, Bornova, Izmir, Turkey; 2 Graduate School of Natural and Applied Sciences, Department of Biotechnology, Ege University, Bornova, Izmir, Turkey; National Key Laboratory of Crop Genetic Improvement, China

## Abstract

The ATP-binding cassette (ABC) protein superfamily constitutes one of the largest protein families known in plants. In this report, we performed a complete inventory of ABC protein genes in *Vitis vinifera*, the whole genome of which has been sequenced. By comparison with ABC protein members of *Arabidopsis thaliana*, we identified 135 putative ABC proteins with 1 or 2 NBDs in *V. vinifera*. Of these, 120 encode intrinsic membrane proteins, and 15 encode proteins missing TMDs. *V. vinifera* ABC proteins can be divided into 13 subfamilies with 79 “full-size,” 41 “half-size,” and 15 “soluble” putative ABC proteins. The main feature of the *Vitis* ABC superfamily is the presence of 2 large subfamilies, ABCG (pleiotropic drug resistance and white-brown complex homolog) and ABCC (multidrug resistance-associated protein). We identified orthologs of *V. vinifera* putative ABC transporters in different species. This work represents the first complete inventory of ABC transporters in *V. vinifera*. The identification of *Vitis* ABC transporters and their comparative analysis with the *Arabidopsis* counterparts revealed a strong conservation between the 2 species. This inventory could help elucidate the biological and physiological functions of these transporters in *V. vinifera*.

## Introduction

The ATP-binding cassette (ABC) protein family is one of the largest and most diverse protein families in plants. These genes encode integral membrane proteins that translocate a wide range of solutes across membranes [1]–[6]. ABC proteins can act as importers, exporters, receptors, and channels [2]. Members of this protein family are involved in diverse cellular processes, including cell division, nutrient uptake, lipid trafficking, antigen processing, drug efflux from cancer cells, and pathogenesis [1]–[4]. ABC proteins have been conserved between prokaryotes and eukaryotes.

A functional ABC protein contains a core unit of 2 transmembrane domains (TMDs) and 2 nucleotide-binding domains (NBDs). The TMD contains 4–6 transmembrane α-helices that are involved in translocating and possibly binding the substrate. The NBD contains highly conserved motifs of Walker A and Walker B boxes and an ABC signature, the H loop and the Q loop [7]. The ABC signature is situated between 2 Walker boxes. The sequences of the TMDs are highly variable compared with those of the NBDs, which contain the evolutionarily conserved Walker A and B consensus motifs for nucleotide binding [8], [9].

The domain organizations of ABC transporters are almost as varied as their function [10], [11]. In many prokaryotes, the NBDs and TMDs are encoded as separate subunits; however, in eukaryotic ABC proteins, the domains are fused to form a single polypeptide [2], [10], [12], also known as full-size ABC proteins, which contain 2 NBDs and 2 TMDs either in forward (TMD1-NBD1-TMD2-NBD2) or reverse orientation (NBD1-TMD1-NBD2-TMD2). The ABC transporters that have 1 NBD and 1 TMD are known as half-size ABC proteins.

Eukaryotic ABC proteins can be classified into 8 major subfamilies (A–H) according to domain organization, the presence of additional domains, and whether the protein is a half-size or full-size transporter, although some subfamilies contain both full-size and half-size transporters [13]. The subfamily H genes have been reported to be absent in plants [13]. Plant ABC subfamilies are usually named after their human or microbial prototypes (e.g., pleiotropic drug resistance (PDR) and multidrug resistance-associated protein (MRP), etc.) as described by Sanchez-Fernandez et al. (2001), while Garcia et al. (2004) used the ABC systems: information on sequence, structure, and evolution (ABCISSE) system of nomenclature for rice ABC proteins.

Several ABC transporters have been characterized in plants. The complete inventories of plant ABC transporters are available for *Arabidopsis*, rice, and *Lotus japonicus* [3], [14]–[16]. The *Arabidopsis* genome contains 131 open reading frames (ORFs) encoding ABC genes, including 54 full-size transporters [3], [14]. However, the physiological roles of these transporters remain to be determined. In the rice genome, 45 sequences encoding putative full-size ABC transporters have been identified [17], while the *Lotus* genome contains 91 putative ABC proteins with 43 full-size, 40 half-size, and 18 soluble proteins [16]. In plants, the best-characterized subfamilies are the multidrug resistance (MDR), MDR-associated proteins (MRP), pleiotropic drug resistance (PDR), and white-brown complex homolog (WBC) subfamilies. ABC transporters in plants can be regulated by a broad range of external signals. For example, PDR-type ABC transporters have been reported to be involved in the response to pathogens [18] and to be regulated by salinity, cold, and heavy metals [19]–[21]. The PDR/ABCG subfamily of plant ABC transporters is able to transport terpenoids [19], [22]. It has been also reported that AtPDR12/ABCG40 functions as a plasma membrane abscisic acid (ABA) uptake transporter and plays a role in the response to ABA [23].

The functions of 4 members of the WBC subfamily have been reported. AtABCG12 and AtABCG11 are required for wax export and elaboration of the cuticle [24]–[27]. AtABCG19 confers antibiotic resistance [28]. AtABCG25 has been shown to be responsible for ABA transport and involved in the ABA signaling pathway [29].

Multiple members of the MDR subfamily are involved in the transport of auxin [30], one of the most important hormones for cell differentiation and response to environmental signals [31]. MRP subfamily members have roles in detoxification and in the vacuolar transport of compounds, including glucuronides and chlorophyll catabolites, and they also show cadmium resistance when expressed in yeast [3], [32].

The recent sequencing of the whole genome of *Vitis vinifera* [33] makes analyses on a genomic scale possible. Here, we describe the first complete analysis of the ABC protein superfamily from the updated 12-fold sequencing and assembly of the grapevine genome. Using these databases, we characterized all members of the ABC protein superfamily of *V. vinifera* and carried out a phylogenetic analysis in comparison with members of *Arabidopsis* ABC superfamily. We employed in this report the nomenclature of human ABC proteins [34], which is commonly approved by the Human Genome Organization (HUGO).

## Materials and Methods

### Identification of ABC Transporter Genes in the *V. vinifera* Genome

The *Arabidopsis* Information Resource (TAIR) database was first used to retrieve *Arabidopsis* ABC protein sequences. *V. vinifera* putative ABC transporters were searched performing a BLASTP analysis (http://www.ncbi.nlm.nih.gov/blast) [35] against the *V. vinifera* proteome 12× database (http://www.genoscope.cns.fr/externe/GenomeBrowser/vitis) using *Arabidopsis* ABC transporter protein sequences as queries. Scores higher than 400 with an “E” value over e-120 were assigned as significant [15]. The sequences of polypeptides corresponding to *V. vinifera* ABC transporters were then analyzed in the Conserved Domain Database (CDD) at NCBI (http://www.ncbi.nlm.nih.gov/Structure/cdd/wrpsb.cgi) and PROSITE (http://prosite.expasy.org/) for the presence of ABC signature motifs [36], [37]. In addition, the NCBI non-redundant protein database was screened with each sequence in order to independently validate the automatic annotation.

### Sequence Analysis and Phylogenetics

The deduced amino acid sequences of the putative ABC proteins were aligned using CLUSTAL W and subjected to phylogenetic analysis by both the maximum parsimony and distance with neighbor-joining methods with 1000 bootstrap replicates [38], [39]. The phylogenetic tree was visualized using MEGA5. Because similar results were obtained with both methods, only the single tree retrieved from the distance analysis is discussed in detail.

For ABC protein subfamilies from both *V. vinifera* and *A. thaliana*, multiple sequence alignment was performed using the multiple sequence comparison by log-expectation (MUSCLE) alignment tool (http://www.ebi.ac.uk/Tools/msa/muscle/) [40] with default program options, and the phylogenetic analysis was performed using a neighbor-joining method with 1000 bootstrap replicates. The phylogenetic trees were constructed with MEGA5 software [41]. The protein theoretical molecular weight and isoelectric point were predicted using compute pI/MW (http://au.expasy.org/tools).

### Orthology Analysis

Orthology analysis was performed using the PHOG web server (http://phylofacts.berkeley.edu/orthologs/) [42]. The sequences that have similarity over 70% and an “E” value of 0.0 were selected. The selected sequences were used in a BLASTP search against the *V. vinifera* protein sequence database, and the best hits were annotated as putative orthologous sequences [43].

### Expressed Sequence Tags Database

The sequences of all of the ABC transporters that were identified were used to query the *V. vinifera* expressed sequence tag (EST) database (http://www.ncbi.nlm.nih.gov/dbEST) for ESTs. The positives sequences were then confirmed by alignment with the query ORF.

## Results/Discussion

### Identification of ABC Transporters in *Vitis vinifera*

Systematic BLAST searches of the grapevine genome proteome 12× database with the amino acid sequences of the ABC transporters from *A. thaliana* as queries identified 135 ORFs encoding putative ABC transporters in *V. vinifera* that contained at least 1 ABC signature (Table S1). Using the presence of TMDs followed by nucleotide-binding folds (NBFs) as criteria for ABC transporters, the *V. vinifera* genome possesses 135 ORFs encoding ABC transporters with 1 or 2 NBFs. Of these, 120 encode intrinsic membrane proteins and 15 encode proteins without TMDs (Table S2). The *V. vinifera* ABC transporter family consists of 79 full-size molecules and 41 half-size transporters.

### Phylogeny of *V. vinifera* ABC Transporters

**Figure 1:**
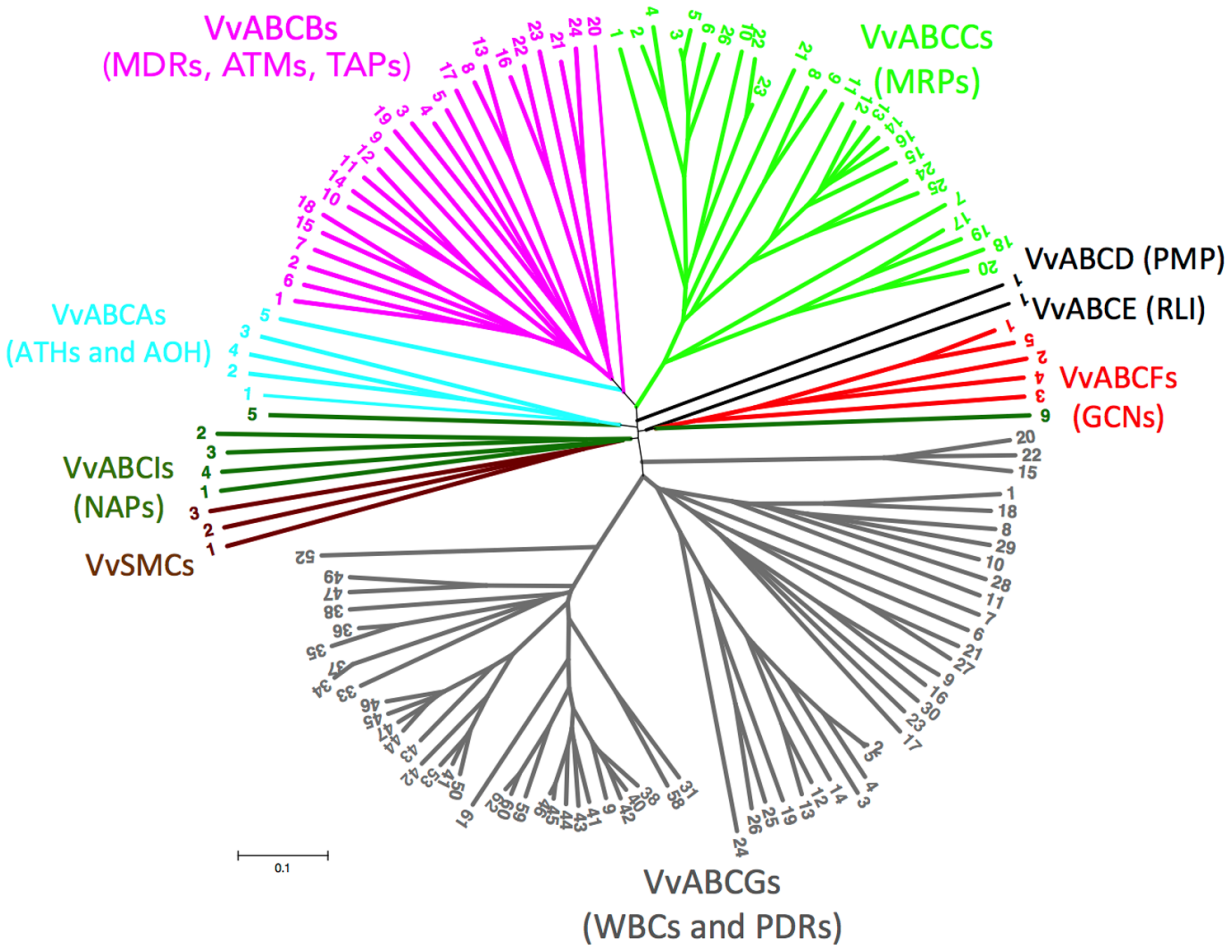
Phylogenetic tree of *Vitis* ABC proteins. The amino sequences of all *Vitis* ABC proteins were aligned using the ClustalW program and were subjected to phylogenetic analysis by the distance with neighbor-joining method. The reliabilities of each branch point, as assessed by the analysis of 1000 computer-generated trees (bootstrap replicates), were in excess of 90%, except for those discussed in the text. The Human Genome Organization (HUGO) nomenclature was used, and the abbreviations of ABC proteins are as follows: ATH, ABC-two-homolog; ATM, ABC transporter of mitochondria; GCN, general control non-repressible; MDR, multi-drug resistance; MRP, multi-drug resistance–associated protein; NAP, non-intrinsic ABC protein; PDR, pleiotropic drug resistance; PMP, peroxisomal membrane protein; RLI, RNase L inhibitor; SMC, structural maintenance of chromosome; TAP, transporter associated with antigen processing; WBC, white-brown complex. ATH belongs to the ABCA subfamily; MDR, TAP, and ATM belong to the ABCB subfamily; MRP belongs to the ABCC subfamily; PMP belongs to the ABCD subfamily; RLI belongs to the ABCE subfamily; GCN belongs to the ABCF subfamily; and WBC belongs to the ABCG subfamily, as described in the text.

All predicted protein sequences were aligned using ClustalW [39], and a phylogenetic tree was generated by the MEGA5 program and maximum parsimony and distance with neighbor-joining methods [38]. One thousand bootstrap replicates were performed for each analysis. Using the MEGA5 program, we built a phylogenetic tree of 135 sequences, which is presented in Fig. 1.

Plant ABC proteins can be divided into 13 subfamilies on the basis of protein size (full, half, or quarter molecules), orientation (forward or reverse), the presence or absence of idiotypic transmembrane and/or linker domains, and overall sequence similarity [14]. The *V. vinifera* genome contains all 13 subfamilies of ABC proteins. The members of each subfamily clustered together more closely with bootstrap values of at least 90% (Fig. 1). The members of most subfamilies grouped more tightly with each other than with members of other subfamilies. MRPs, PDRs, and general control non-repressible proteins (GCNs) grouped within their respective subfamilies. The VvABCG (WBC) subfamily clusters tightly, with the exception of VvABCG15, VvABCG20, and VvABCG22, which clustered closely with the main WBC cluster. The VvABCF (GCN) subfamily is composed of 5 members, and the 5 members cluster within the same clade.

Among the members of the VvABCI (NAP) subfamily, VvABCI1, VvABCI2, VvABCI3, and VvABCI4 clustered with structural maintenance of chromosome proteins (SMCs) within the same clade, whereas VvABCI5 grouped with VvABCAs (ATH) in the same clade, and VvABCI6 was closely related to the VvABCF (GCN) subfamily. The lack of coherence within the ABCI (NAP) subfamily was to be expected since this heterogeneous group of proteins lack contigous transmembrane domains and grouped together by their lack of any systematic resemblance to previously defined ABC proteins [14]. Similarly, in *Arabidopsis*, some NAPs did not group with each other or within other subfamilies with high confidence [14]. All of the VvABCB (MDR) subfamily members grouped together with bootstrap values of 90% with the exception of VvABCB16, which was distributed close to the transporter associated with antigen processing (TAP) subfamily. The only member of the ABCA (AOH) subfamily, VvABCA1, grouped with the members of the VvABCA (ATH) subfamily (Fig. 1). The peroxisomal membrane protein (PMP) subfamily contains only 1 member, and it is classified as a half-size transporter. Three members of the VvABCA (ATH) subfamily grouped within the same clade with bootstrap values up to 100%, whereas 1 member, VvABCA5, clustered within the VvABCB (MDR/TAP) clade. In accordance with our results, in *Arabidopsis*, none of AtATHs grouped within any of the other subfamilies with the exception of AtATH12 which grouped within the MDR/TAP/ATM clade [14]. The VvABCE (RLI) subfamily contains only 1 member, VvABCE1, and it clustered within the VvABCF (GCN) clade. The VvABCB (ATM) subfamily has only 1 member, and it grouped within the TAPs/MDRs/ATH clade.

#### ABCA subfamily

The plant ABCA subfamily consists of full-size and half-size proteins. Only 1 full-size ABCA gene (AtABCA1), also known as the ABC one homolog (AOH), is present in the *Arabidopsis* genome, whereas no homolog has been identified in the rice genome [14], [17]. In the *Lotus* genome, 1 ABCA member similar to AtABCA1 has been found [16]. The *Arabidopsis* genome contains 11 half-size ABCA genes, also known as ABC two homologs (ATH) [14], [15], while the *Lotus* genome has at least 2 half-size members of the ABCA subfamily [16].

The *Vitis* genome harbors only 1 ORF (VvABCA1) with high resemblance to AtAOH1, a full-size transporter in the forward orientation [14]. VvABCA1 is one of the longest ABC transporter proteins located on chromosome 8 with 2001 amino acid residues including a putative regulatory domain that is interrupted by a hydrophobic segment in the central region of the molecule (Table S1), which is similar to the human protein and AtAOH1. Its mammalian counterpart, ABC1 is localized to the plasma membrane and Golgi complex and is responsible for Tangier disease [44], [45]. The function and the localization of AtAOH1, the *Arabidopsis* homolog, remains unknown, and it is speculated that it may play a role in lipid accumulation during seed maturation or lipid mobilization during seed germination [19]. At present, the representation of VvABCA1 in EST databases (6 ESTs) is observed in leaves, berries, flowers, and roots tissues (Table S4), and no cDNA corresponding to VvABCA1 has been isolated.

**Figure 2:**
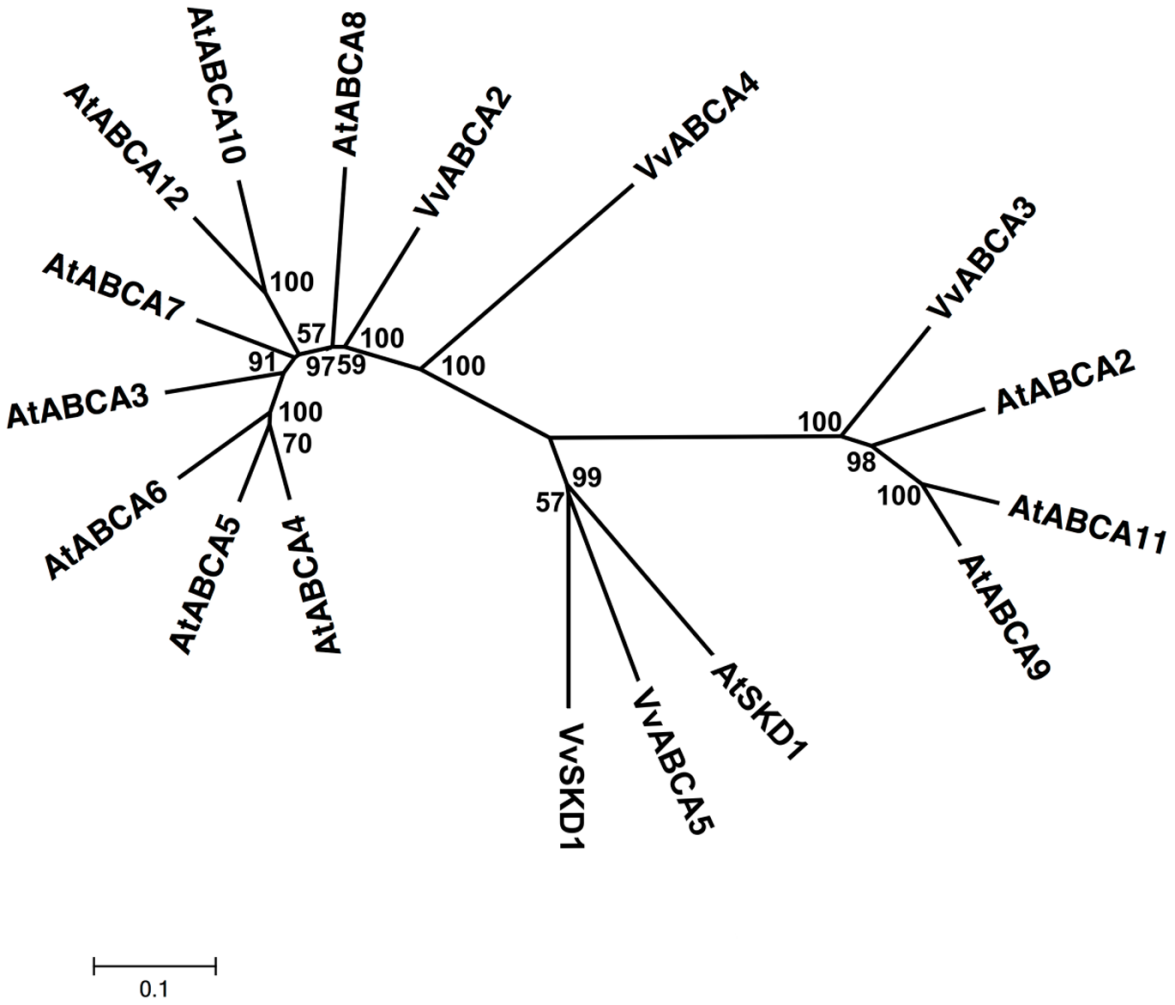
Phylogenetic relationship of *Arabidopsis* and *Vitis* ABCA (ATH) proteins. The amino acid sequences of all *Arabidopsis* ABCA (ATH) proteins and those of *Vitis vinifera* were aligned using the MUSCLE program and subjected to phylogenetic analysis by the distance with neighborjoining method using MEGA5 programme. Accession numbers for Arabidopsis sequences are AtABCA2 (NP_190357.2), AtABCA3 (NP_190358.2), AtABCA4 (NP_190359.4), AtABCA5 (NP_190360.2), AtABCA6 (NP_190361.2), AtABCA7 (NP_190362.2), AtABCA8 (NP_190363.3), AtABCA9 (NP_200981.1), AtABCA10 (NP_200982.1), AtABCA11 (NP_200977.1) and AtABCA12 (NP_200978.1). AtSKD1 (AEC08019.1) and VvSKD1 (XP_002266534.1) were used as outgroups.

The ATH subfamily, which has 4 members in the *Vitis* genome, is the half-size transporter category with 723–958 amino acid residues (Table S1, Fig. 2), whereas the *Arabidopsis* genome contains 11 ORFs. VvABCA2 and VvABCA3 are located on chromosome 17 and share 23% similarity. Twenty-two plant orthologs from different species have been found using an orthology analysis program for this subfamily (Table S3). The human orthologs are involved in Stargardt disease or fundus flavimaculatus [46], [47]. The expression of AtATH14 and AtATH15 in *Arabidopsis* is regulated in response to salt stress [48]. We identified 20 ESTs corresponding to *Vitis* ATH subfamily members in various tissues (Table S4).

#### ABCB subfamily

The ABCB subfamily consists of full-size members, which are conventionally named MDR or PGP, and half-size members such as TAPs and ATMs. In the *Arabidopsis* genome, 22 full-size members (MDR) and 6 half-size members (3 TAPs and 3 ATMs) are present [14], [15], whereas the rice genome contains 24 full-size (MDR) and 4 half-size (3 TAPs and 1 ATM) proteins [15]. In total, the number of *Lotus* ABCB proteins is estimated as 15, which implies 12 full-size MDR-type, 2 TAP-like, and 1 ATM-like protein [16]. The *Vitis* ABCB subfamily consists of 19 MDR-type, 5 TAP-like, and 1 ATM-like proteins.

**Figure 3:**
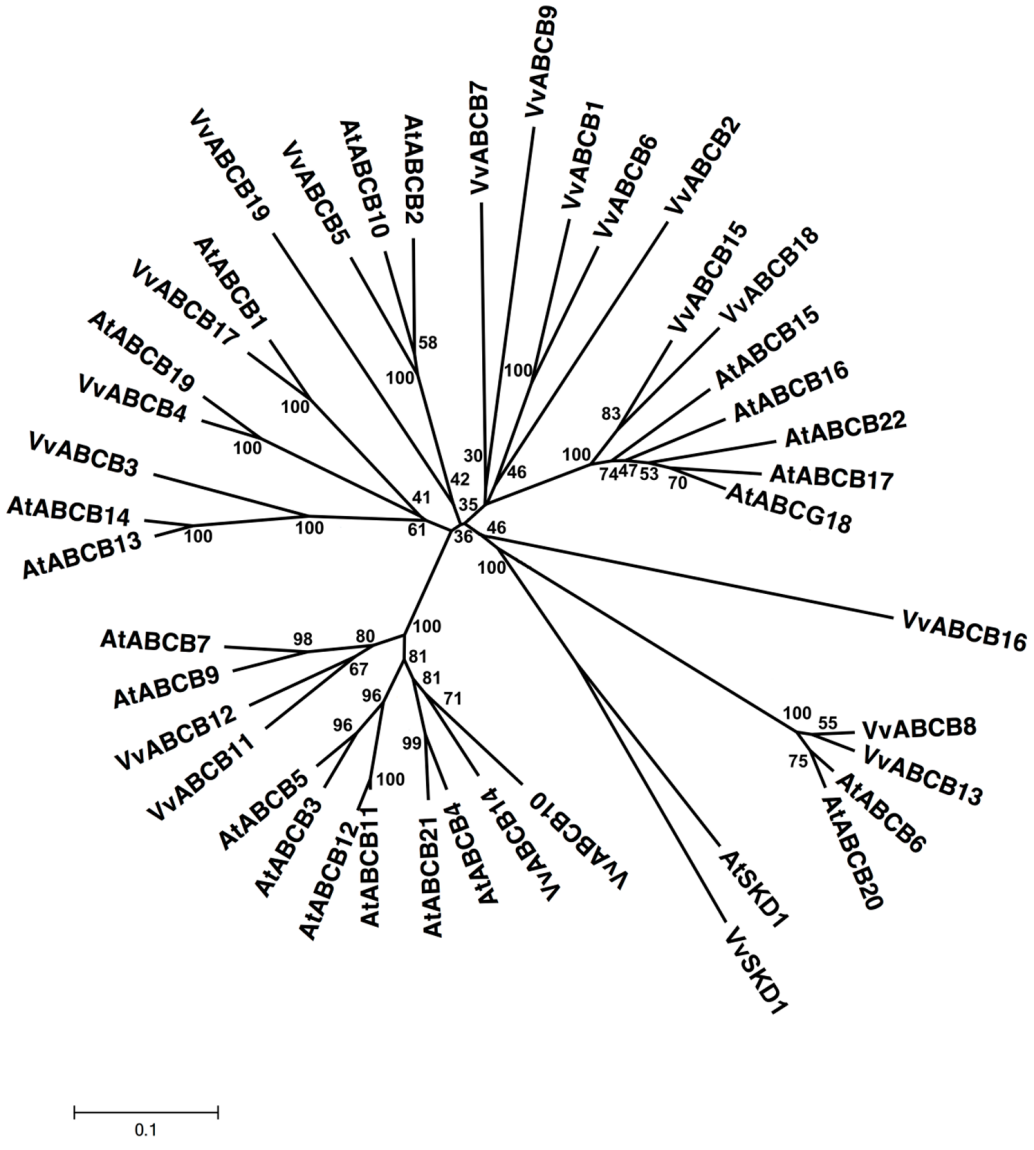
Phylogenetic tree of ABCB (MDR) protein sequences from *Arabidopsis* and *Vitis vinifera*. The amino acid sequences of all *Arabidopsis* ABCB (MDR) proteins and those of *Vitis vinifera* were aligned using the MUSCLE program and subjected to phylogenetic analysis by the distance with neighborjoining method using MEGA5 programme. Accession numbers for Arabidopsis sequences are AtABCB1 (NP_181228.1), AtABCB2 (NP_194326.2), AtABCB3 (NP_192091.1), AtABCB4 (NP_182223.1), AtABCB5 (NP_192092.1), AtABCB6 (NP_181480.1), AtABCB7 (NP_199466.1), AtABCB8 (NP_683599.1), AtABCB9 (NP_193539.6), AtABCB10 (NP_172538.1), AtABCB11 (NP_171753.1), AtABCB12 (NP_171754.1), AtABCB13 (NP_174115.1), AtABCB14 (NP_174122.1), AtABCB15 (NP_189475.1), AtABCB16 (NP_189477.4), AtABCB17 (NP_189479.1), AtABCB18 (NP_189480.1), AtABCB19 (NP_189528.1), AtABCB20 (NP_191092.1) and AtABCB21 (NP_191774.1). AtSKD1 (AEC08019.1) and VvSKD1 (XP_002266534.1) were used as outgroups.

With 19 members, the MDR subfamily represents the fourth largest full-size molecule ABC transporter subfamily in *V. vinifera* (Table S1, Table S2). All of the identified ORFs were named VvABCB1 through 19; they contain 2 TMDs and 2 NBDs in the forward orientation and range from 814 amino acids (VvABCB18) to 2405 amino acids (VvABCB12) in length (Table S1). Members of the *Vitis* MDR subfamily show 22–79% identity to each other. The VvABCB17 amino acid sequence shows 84% similarity with AtABCB1, and VvABCB4 shows 88% similarity with AtABCB19 from *A. thaliana*. VvABCB17 shares between 84.5% and 89.6% similarity to the MDR members from *A. thaliana*, *Oryza sativa*, and *Ricinus communis* (Table S3). The similarity between VvABCB17 and its homolog AtABCB1 and between VvABCB4 and AtABCB19 was confirmed in a phylogenetic tree that was constructed with all of the *Arabidopsis* members with bootstrap values of 100% (Fig. 3). Similarly, the phylogenetic analysis of *V. vinifera* and *A. thaliana* MDR subfamilies confirmed the orthologs of VvABCB8/AtABCB20/AtABCB6, VvABCB13/AtABCB20, and VvABCB14/AtABCB3/AtABCB11/AtABCB12/AtABCB21 (Fig. 3).

The ABCB proteins were first characterized in mammalian cells because their overexpression confers a multidrug resistance phenotype [49]. One member of this subfamily in *Arabidopsis*, AtMDR1, also known as AtPGP1, was reported to confer herbicide tolerance when it was overexpressed in plants [50]. Multiple members of the ABCB/PGP/MDR subfamily are involved in the transport of auxin [30], suggesting that the ABCB subfamily probably plays an important role in auxin transport. Recently, it was proposed that both ABCB14 and AtABCB15 in *Arabidopsis* promoted auxin transport, and reduced auxin transport was correlated with a mild disruption in vascular development [31]. All 19 identified ORFs encoding MDR proteins are transcriptionally active. We identified 177 ESTs corresponding to the members of *Vitis* MDR subfamily (Table S5). The BLAST analysis of ESTs revealed their expression in various tissues such as flowers, roots, tendrils, berries, buds, and leaves (Table S5).

**Figure 4:**
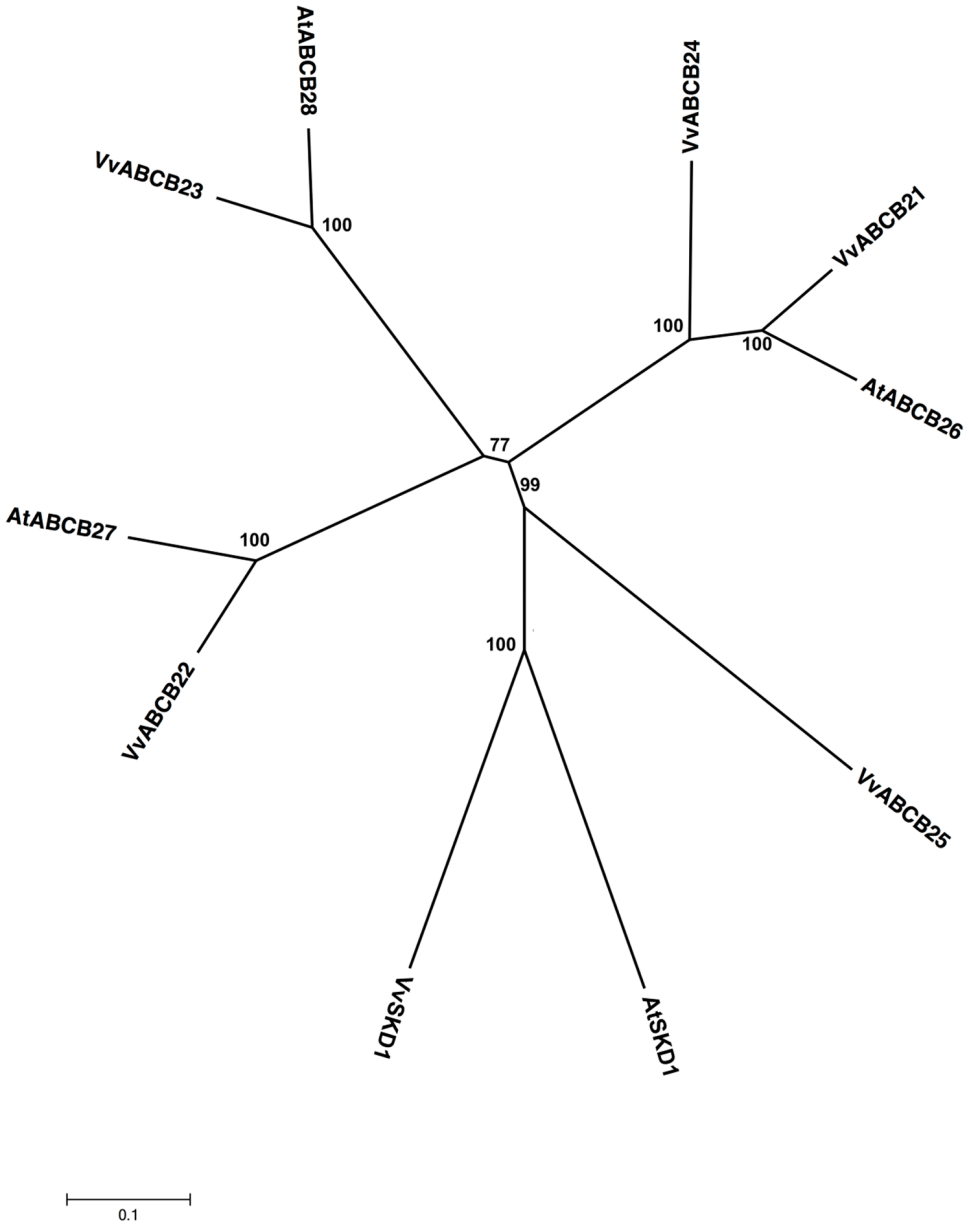
Phylogenetic tree of ABCB (TAP) protein sequences from *Arabidopsis* and *Vitis vinifera*. The amino acid sequences of all *Arabidopsis* ABCB (TAP) proteins and those of *Vitis vinifera* were aligned using the MUSCLE program and subjected to phylogenetic analysis by the distance with neighborjoining method using MEGA5 programme. Accession numbers for Arabidopsis sequences are AtABCB26 (NP_177218.3), AtABCB27 (NP_198720.2) and AtABCB28 (NP_194275.2). AtSKD1 (AEC08019.1) and VvSKD1 (XP_002266534.1) were used as outgroups.

Five ORFs (VvABCB21 through VvABCB25) were identified that encoded putative TAP-like proteins with high similarity with 3 TAPs from *A. thaliana* (Table S1). *Vitis* TAP-like proteins and their orthologs from *A. thaliana* grouped into the same clade and shared strong similarity (70–80%) with each other. *Vitis* TAP-like transporters encode half-size proteins in the forward orientation. VvABCB24 and VvABCB25, which are located on chromosome 14, contain 658 and 1265 amino acids, respectively (Table S1). VvABCB21 shares 71% similarity with VvABCB24, and VvABCB22 shares 73% similarity with VvABCB25. Based on phylogenetic analysis, 3 ortholog pairs between 2 species were identified: VvABCB21/AtABCB26 (80% similarity), VvABCB22/AtABCB27 (75% similarity), and VvABCB23/AtABCB28 (70% similarity) (Fig. 4). Orthology analysis by the PHOG program reveals that only VvABCB21 shares more than 80% identity with TAPs from *Populus trichocarpa*, *A. thaliana*, and *R. communis* (Table S3).

The function of AtABCB26, 27, and 28 remain to be determined. While the function of the yeast TAP homologs, MDL1 and MDL2 [51], as well as that of the *Arabidopsis* homolog are unknown, mammalian counterparts participate in peptide secretion and translocation across endoplasmic reticulum (ER) membranes [52]. The size of the *Vitis* TAP subfamily seems to be larger than that of *Arabidopsis*, which contains 3 TAP-like genes. Four of the 5 ORFs encoding *Vitis* TAP-like proteins are represented in the EST (67 ESTs) database (Table S5) and are expressed in different tissues such as leaves, roots, fruits, flowers, berries, and buds. No ESTs have been identified for VvABCB23, suggesting that it is not transcriptionally active.

The *Vitis* genome contains only 1 ORF encoding an ATM-like protein, and this ORF is located on chromosome 6 (Table S1). This subfamily is composed of a half-size transporter of 726 amino acids with the forward orientation that is named VvABCB20. To date, 11 ESTs corresponding to VvABCB20 have been described (Table S5). The ATM subfamily from *Arabidopsis*, which includes 3 ORFs, is larger than that of *V. vinifera*. The *Arabidopsis* ATM homolog, AtATM3, has been implicated in the biogenesis of iron-sulfur proteins [53] and has a crucial role in molybdenum cofactor (moco) biosynthesis [54]. AtATM3 was also reported to be involved in heavy metal resistance [55]. The deficiency of AtATM3 causes dwarfism and chlorosis [53], [56]. A barley half-size TAP-like protein, ID17, was identified as an iron deficiency–induced gene [57]. The biochemical roles of plant half-size TAP proteins of subfamily B have not yet been determined.

#### ABCC subfamily

**Figure 5:**
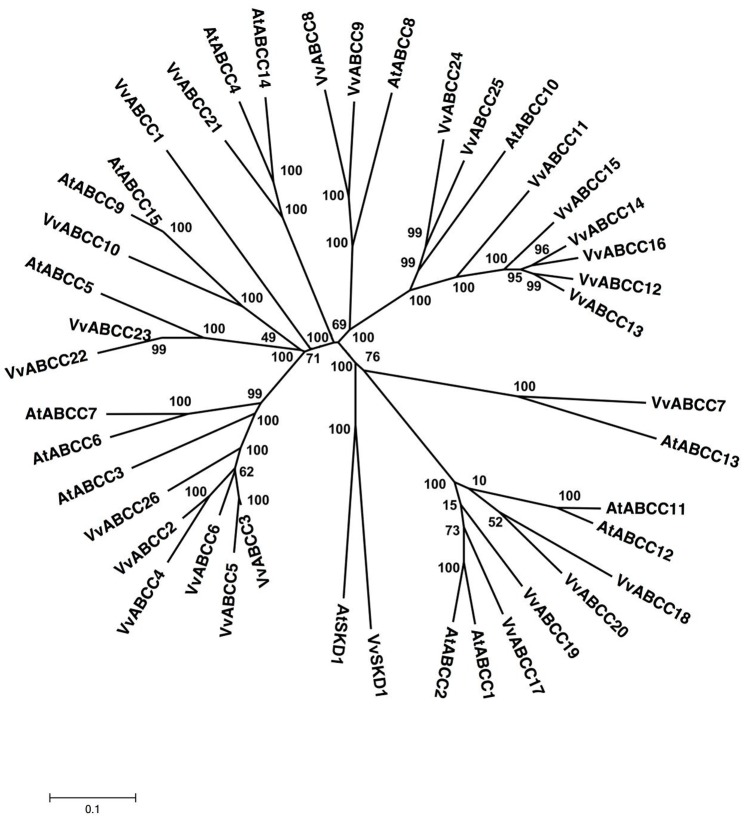
Phylogenetic tree of ABCC (MRP) protein sequences from *Arabidopsis* and *Vitis vinifera*. The amino acid sequences of all *Arabidopsis* ABCC (MRP) proteins and those of *Vitis vinifera* were aligned using the MUSCLE program and subjected to phylogenetic analysis by the distance with neighborjoining method using MEGA5 programme. Accession numbers for Arabidopsis sequences are AtABCC1 (NP_174329.1), AtABCC2 (NP_181013.1), AtABCC3 (NP_187915.1), AtABCC4 (NP_182301.1), AtABCC5 (NP_171908.1), AtABCC6 (NP_188762.3), AtABCC7 (NP_187917.3), AtABCC8 (NP_187916.3) AtABCC9 (NP_191575.2), AtABCC10 (NP_191829.1), AtABCC11 (NP_178811.7), AtABCC12 (NP_174331.2), AtABCC13 (NP_174330.3), AtABCC14 (NP_191473.2) and AtABCC15 (NP_191656.2). AtSKD1 (AEC08019.1) and VvSKD1 (XP_002266534.1) were used as outgroups.

ABCC subfamily proteins are full-size ABC transporters also known as MRPs, which contain an N-terminal extension of the TMD. This subfamily consists of 15 members in the *Arabidopsis* genome and 17 members in the rice genome [14], [15]. With 26 members, the ABCC (MRP) subfamily represents the third largest subfamily of *V. vinifera* full-size ABC transporters, which is larger than that of *A. thaliana* (15 members). ABCC (MRP) subfamily members are full-size molecules in the forward orientation containing (TMD-NBD)_2_ and ranging in size from 759 amino acids (VvABCC19) to 2772 amino acids (VvABCC8) (Table S1). The members of the MRP subfamily in the *Vitis* genome share 29–95% similarity with each other. Among them, 7 ORFs that share strong similarity (76–85%) are localized on chromosome 2 (Table S1). Similarly, VvABCC2, VvABCC3, VvABCC4, VvABCC5, VvABCC6, and VvABCC26 show between 73% and 95% similarity with each other with bootstrap values of 99% (Fig. 5). Interestingly, these 6 ORFs are located on chromosome 19 in tandem regions (Table S1). Four other ORFS (VvABCC17, VvABCC18, VvABCC19, and VvABCC20) displaying between 69% and 77% similarity with each other are located on chromosome 10. The phylogenetic analysis of MRP subfamilies from *V. vinifera* and *A. thaliana* reveals that these subfamilies can be classified into 5 major groups (Fig. 5). A first group contains MRPs mostly from *V. vinifera* and includes 6 ORFs located on chromosome 2, while a second group presents the *A. thaliana* orthologs of VvABCC21, AtABCC4, and AtABCC14. VvABCC21 is 74% and 76% identical to its *A. thaliana* orthologs, AtABCC4 and AtABCC14, respectively (Table S3), by phylogeny analysis (Fig. 5). The other groups include protein sequences from both species. By phylogeny analysis, we also identified orthologs of *Vitis* MRPs in *Arabidopsis* such as VvABCC17/AtABCC1/AtABCC2 (76 and 78% similarity), VvABCC22/AtABCC5 (78% similarity), and VvABCC21/AtABCC14 (75% similarity) with strong bootstrap values (99–100%). VvABCC9 shares 80.2% identity with its homolog from *Arabidopsis* (NP171908) on the basis of orthology analysis, which is confirmed by the phylogenetic analysis of the same sequences (Fig. 5, Table S3).

MRPs consist of 3 additional subfamily-specific structures: a 200–amino acid hydrophobic N-terminal extension (TMDO) containing 5 putative transmembrane spans, a linker (L) domain contiguous with NBF1 and rich in charged amino acid residues, and a hydrophilic C-terminal extension [58]. Interestingly both AtMRP11 and AtMRP15 lack the TMDO characteristic of many members of this subfamily [14], [59]. Human MRP1 and MRP2 can transport glutathione S (GS)-conjugates, whereas their orthologs from *Arabidopsis* are able to transport materials other than GS-conjugates. This structural divergence was also determined within this subfamily among human and yeast MRP transporters [58], [60]. The ABCC subfamily is also involved in the detoxification processes. These proteins have a role in vacuolar transport and confer cadmium tolerance in yeast [3], [61]. To date, none of *Vitis* MRP homologs have been cloned or characterized. However, 435 ESTs were found for this subfamily in various tissues in response to stresses (Table S6). The ESTs for all of the MRPs have been identified (Table S6).

#### ABCD subfamily

The ABCD subfamily contains predominantly half-size proteins that are conventionally designated as PMPs, which are localized at the peroxisome. The members of this subfamily homodimerize and heterodimerize to form transporters that are responsible for the import of fatty acids into the peroxisome.

The *Arabidopsis* and rice genomes contain 1 and 2 half-size ABCD members, respectively, in addition to 1 half-size protein for each plant [14], [15]. In the *Lotus* genome, 4 and 3 fragments have similarity to half-size and full-size ABCD proteins, respectively [16]. The *Vitis* ABCD subfamily consists of 1 member of the PMP type, which is named VvABCD1 (Table S1). VvABCD1 has orthologs from *O. sativa*, *R. communis*, *A. thaliana*, and *P. trichocarpa* with up to 93.8% similarity (Table S3).

In *Arabidopsis*, full-size ABCD proteins also known as peroxisomal ABC transporter (PXA1), peroxisome defective (PED3), and comatose (CTS) or AtPMP2 are involved in the peroxisomal import of acyl-CoA esters [62]–[64]. These mutants have defects in germination, fertility, and growth [65], [66]. There are 8 ESTs found in the *Vitis* genome (Table S7).

### Soluble ABC Proteins

#### ABCE subfamily

The members of the ABCE subfamily have 2 NBDs but no TMD, and they are also known as RNase L inhibitors (RLI) [67]. In the *Arabidopsis* and rice genomes, there are 2 members of this subfamily, and the *Lotus* genome has at least 1 member [16]. The *Vitis* genome contains only 1 ORF encoding an RLI-like protein, VvABCE1, which has 2 NBDs but no transmembrane spans (Table S1). RLI1 contains N-terminal “ferrodoxin” (4Fe4S-type) motifs. These motifs have been shown to interact with nucleic acids [68]. On the basis of sequence identity, VvABCE1 shares more than 90% identity with its orthologs from *Arabidopsis*, *O. sativa*, and *Triticum aestivum* (Table S3). The *Arabidopsis* ABCE protein AtRLI2 has been shown to suppress RNA silencing [69]. Nine ESTs have been identified for this subfamily in *V. vinifera* (Table S7).

#### ABCF subfamily

**Figure 6:**
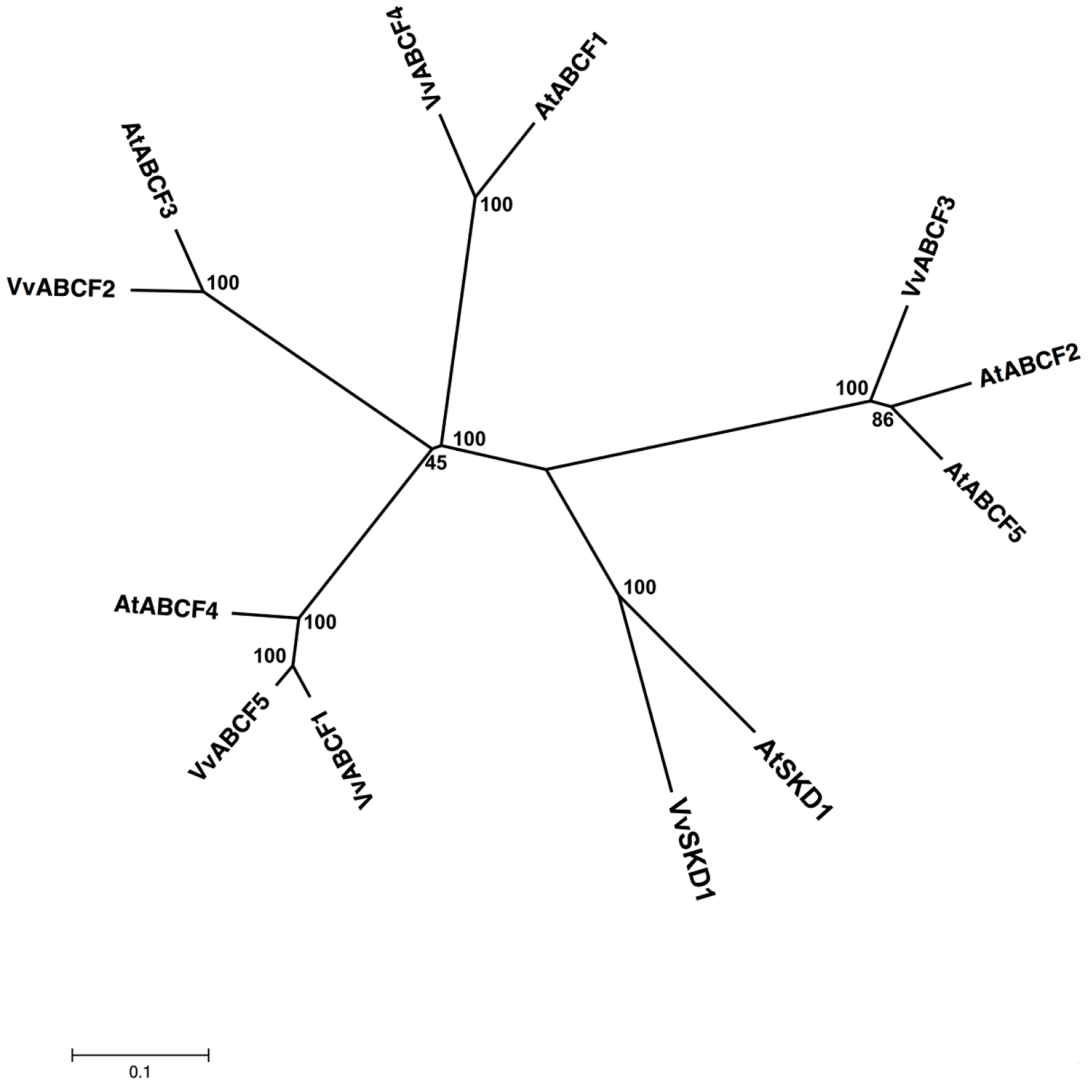
Phylogenetic tree of ABCF (GCN) protein sequences from *Arabidopsis* and *Vitis vinifera*. The amino acid sequences of all Arabidopsis ABCF (GCN) proteins and those of *Vitis vinifera* were aligned using the MUSCLE program and subjected to phylogenetic analysis by the distance with neighborjoining method using MEGA5 programme. Accession numbers for Arabidopsis sequences are AtABCF1 (NP_200887.1), AtABCF2 (NP_196555.2), AtABCF3 (NP_176636.1), AtABCF4 (NP_567001.1) and AtABCF5 (NP_201289.1). AtSKD1 (AEC08019.1) and VvSKD1 (XP_002266534.1) were used as outgroups.

Genes in the ABCF subfamily, which have 2 NBDs and no TMD, are also conventionally known as the GCN subfamily. Both the *Arabidopsis* and rice genomes have 5 members of this subfamily [14], [15]. The *Vitis* genome contains 5 members of ABCF/GCN subfamily that have 2 NBDs but no TMDs (Table S1). The members of the ABCF/GCN subfamily share 24–79% identity with each other and are distributed on various chromosomes (2, 6, 7, and 18) (Table S1). They also show 76–84% similarity to 5 ORFs that correspond to the GCN-like proteins in *Arabidopsis* (Fig. 6). Four members of the *Vitis* ABCF/GCN subfamily (VvABCF1, 2, 3, and 4) share 80.5–94.8% similarity with their orthologs from various plants (Table S3). At least 125 ESTs have been identified for all of the *Vitis* ABCFs (Table S8).

#### ABCG subfamily

The ABCG subfamily is a large group of half-size transporters with the reverse orientation (NBD-TMD) and is also known as the white-brown complex (WBC) subfamily. This subfamily has 29 and 30 members in the *Arabidopsis* genome and the rice genome, respectively [14], [15]. Since AtWBC15 and AtWBC22 were reassigned as AtABCG15 according to new nomenclature in the Arabidopsis Information Resources (TAIR), 28 ORFs were subjected to phylogenetic analysis.

**Figure 7:**
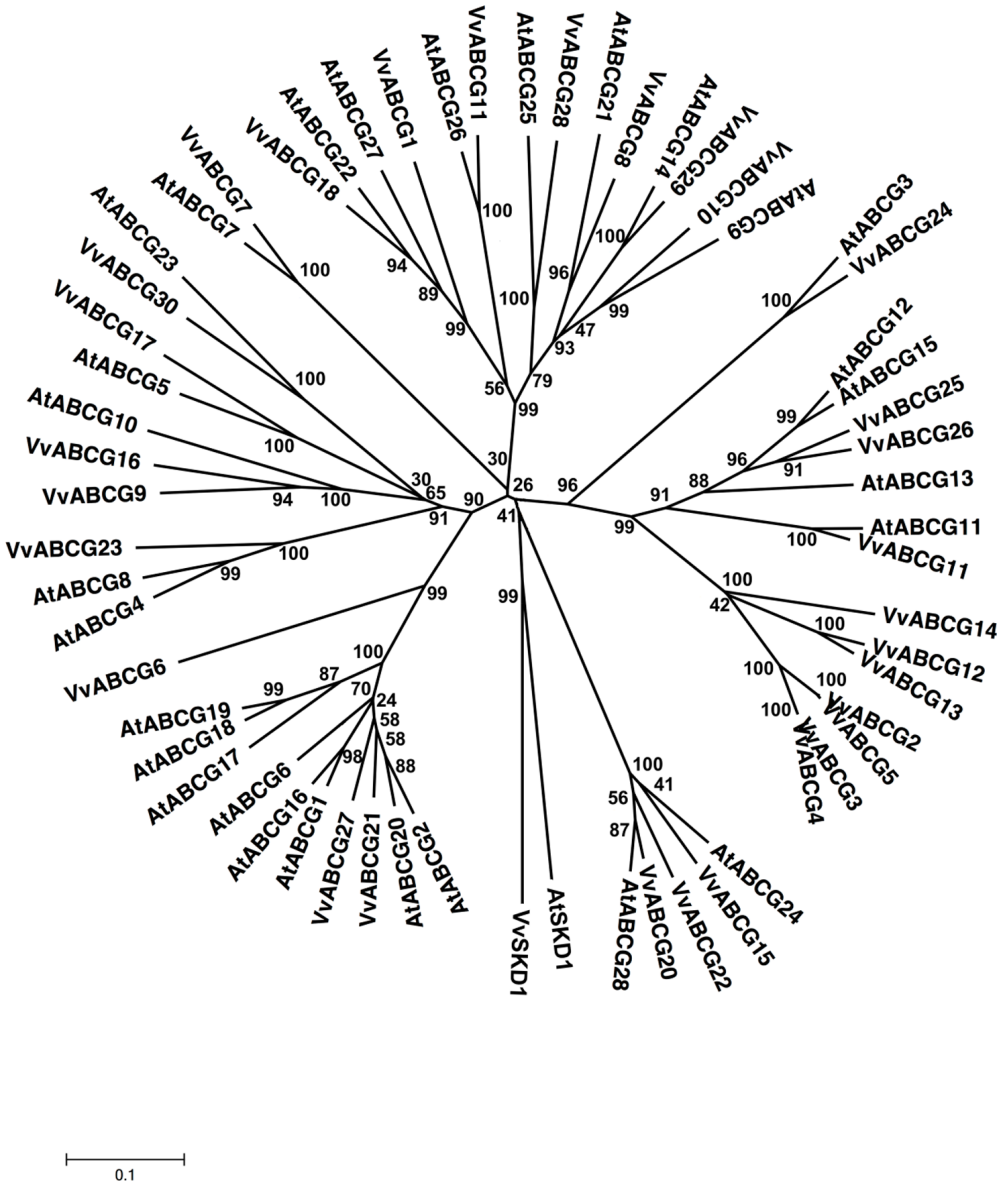
Phylogenetic tree of ABCG (WBC) protein sequences from *Arabidopsis* and *Vitis vinifera*. The amino acid sequences of all *Arabidopsis* ABCG (WBC) proteins and those of *Vitis vinifera* were aligned using the MUSCLE program and subjected to phylogenetic analysis by the distance with neighborjoining method using MEGA5 programme. Accession numbers for Arabidopsis sequences are AtABCG1 (NP_181467.1), AtABCG2 (NP_181272.1), AtABCG3 (NP_850111.1), AtABCG4 (NP_194305.1), AtABCG5 (NP_178984.1), AtABCG6 (NP_196862.1), AtABCG7 (NP_178241.1), AtABCG8 (NP_200098.1), AtABCG9 (NP_194472.2), AtABCG10 (NP_175734.1), AtABCG11 (NP_173226.2), AtABCG12 (NP_175561.1), AtABCG13 (NP_175557.1), AtABCG14 (NP_564383.1), AtABCG15 (NP_188746.2), AtABCG16 (NP_191069.2), AtABCG17 (NP_191070.1), AtABCG18 (NP_191071.1), AtABCG19 (NP_191073.1), AtABCG21 (NP_189190.2 ), AtABCG22 (NP_568169.1), AtABCG23 (NP_197442.1), AtABCG24 (NP_175745.4), AtABCG25 (NP_565030.1), AtABCG26 (NP_187928.2), AtABCG27 (NP_190799.1) and AtABCG28 (NP_200882.4). AtSKD1 (AEC08019.1) and VvSKD1 (XP_002266534.1) were used as outgroups.

Thirty ORFs showing strong similarity with the 28 *Arabidopsis* WBC-like proteins were identified (Fig. 7) and shared 17–99% similarity with each other. They have been named VvABCG1 through 30 and are half-size transporters with the NBD-TMD organization (Table S1). Among the identified ORFs encoding putative WBCs in *Vitis*, the VvABCG2 amino acid sequence shares 99% similarity with VvABCG5 (bootstrap values of 100%). The main difference between these 2 nucleotide sequences was found in some single nucleotide polymorphisms and in the 3′ untranslated region (UTR). Interestingly, VvABCG2 and VvABCG5 are located on different chromosomes, unknown and 7, respectively (Table S1). Similarly, VvABCG2 and VvABCG4 are located on unknown chromosomes and share 86% similarity with each other. In addition, VvABCG4 shows 84% similarity with VvABCG5. VvABCG12 displays 84% similarity with VvABCG13. The phylogenetic analysis of *Vitis* putative WBC transporters and those of *Arabidopsis* reveals that this subfamily is divided into 3 main groups with bootstrap values up to 90% (Fig. 7). The members of the *Vitis* ABCG/WBC subfamily are distributed almost equally in all 3 subclasses with their homologs from *Arabidopsis*. Finally, the phylogenetic analysis of *V. vinifera* and *A. thaliana* WBC proteins allowed us to identify several orthologs in the 2 species (Fig. 7) such as VvABCG7/AtABCG7 (76% similarity), VvABCG11/AtABCG26 (76% similarity), VvABCG17/AtABCG5 (74% similarity), VvABCG18/AtABCG22 (76% similarity), VvABCG21/AtABCG2 (74% similarity), VvABCG21/AtABCG20 (75% similarity), VvABCG24/AtABCG3 (80% similarity), and VvABCG29/AtABCG14 (79% similarity) that were grouped into the same clade and shared 74–80% similarity with each other. In addition, VvABCG19 showed 95.3% identity to its ortholog from *R. communis* by orthology analysis (Table S3).

The yeast genome harbors only 1 WBC homolog (ADP1) of unknown function [51], and the human genome contains 5 homologs, which participate in the transport of sterols and possibly other lipids [70]. *Drosophila* ABCG proteins are required in eye pigment formation, while human ABCG transporters are involved in sterol transport [71], [72]. Plant WBC homologs have been recently cloned. AtABCG11 and AtABCG12 were reported to be involved in the transport of cuticular wax, and AtWBC19 confers kanamycin resistance in *Arabidopsis* [24], [28]. It has been reported very recently that AtABCG25 is responsible for ABA transport and is involved in the ABA signaling pathway [29]. There are at least 198 ESTs for all members of this subfamily from *V. vinifera* (Table S9), but none of the ESTs have been cloned or characterized.

In addition to half-size ABC transporters, plant genomes contain a large group of full-size ABCG subfamily transporters in the reverse orientation (NBD1-TMD1-NBD2-TMD2), which are also PDR. In the *Arabidopsis* and rice genomes, 15 and 21 PDRs have been identified, respectively [14], [15].

**Figure 8:**
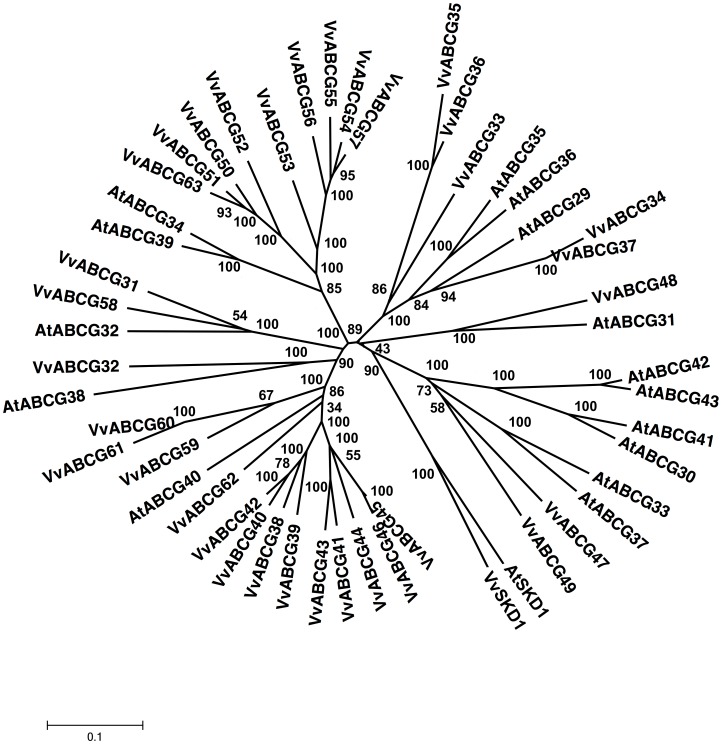
Phylogenetic tree of ABCG (PDR) protein sequences from *Arabidopsis* and *Vitis vinifera*. The amino acid sequences of all *Arabidopsis* ABCG (PDR) proteins and those of *Vitis vinifera* were aligned using the MUSCLE program and subjected to phylogenetic analysis by the distance with neighborjoining method using MEGA5 programme. Accession numbers for Arabidopsis sequences are AtABCG29 (NP_190919.1), AtABCG30 (NP_566543.1), AtABCG31 (NP_193258.2), AtABCG32 (NP_180555.2), AtABCG33 (NP_180259.1), AtABCG34 (NP_181265.1), AtABCG35 (NP_181179.2), AtABCG36 (NP_172973.1), AtABCG37 (NP_176196.1), AtABCG38 (NP_190916.1), AtABCG39 (NP_683617.1), AtABCG40 (NP_176867.2), AtABCG41 (NP_173005.1), AtABCG42 (NP_680692.1), AtABCG43 (NP_680693.5) and AtABCG44 (NP_680694.2). AtSKD1 (AEC08019.1) and VvSKD1 (XP_002266534.1) were used as outgroups.

The PDR subfamily in *V. vinifera* is the largest ABC transporter subfamily and includes full-size ABC transporters that are encoded by 33 ORFs, namely, VvABCG31 through VvABCG63 (Table S1). Its size is larger than that of the *A. thaliana* PDR subfamily, which contains 15 members [14], [73]. The PDR subfamily is characterized by the presence of NBDs and TMDs in the reverse orientation and is only found in fungi and plants [51], [74]. The sequence analysis of ORFs encoding *V. vinifera* putative PDR subfamily members revealed the presence of (NBD-TMD)_2_ in the reverse orientation (Table S1). Five PDR subfamily members are located on chromosome 4, 4 members on chromosome 13, 5 members on chromosome 6, 2 members on chromosome 11, 1 member on chromosome 5, 1 member on chromosome 8, 1 member on chromosome 14, and 14 members on chromosome 9 (Table S1). Most PDR subfamily members are distributed on chromosome 9. Members of the *Vitis* PDR subfamily share up to 92% similarity between each other and contain between 804 (VvABCG61) and 3142 (VvABCG46) amino acid residues (Table S1). The phylogenetic tree analysis of *V. vinifera* and *A. thaliana* subfamilies reveals that these proteins can be classified into 3 major groups (Fig. 8). The phylogenetic analysis of *V. vinifera* and *A. thaliana* PDR subfamilies identified 5 ortholog pairs that included VvABCG31/AtABCG32 (77% similarity), VvABCG37/AtABCG35 or AtABCG36 (73% similarity), VvABCG37/AtABCG29 (76% similarity), VVABCG53/AtABCG34 or AtABCG39 (76% similarity), and VvABCG46/AtABCG40 (73% similarity) with bootstrap values up to 75% (Fig. 8).

Members of this family confer resistance to various biotic and abiotic stresses [20], [21], [75]–[77]. The first plant PDR gene identified, SpTUR2, is regulated in response to abiotic stress [73], [78]. Another plant PDR, OsPDR29 from rice, participates in the abiotic stress response [79]. It was recently shown that NpPDR1 plays a role in plant defense responses [80], while AtPDR12 is a plasma membrane ABA uptake transporter in guard cells and is involved in resistance to lead [20], [23]. We identified 543 ESTs corresponding to 32 members of the *Vitis* PDR subfamily (Table S9). No ESTs have been identified for VvPDR31, suggesting that it is not transcriptionally active. Among the ORFs corresponding to the PDR subfamily in *Vitis*, 32 are transcriptionally active, but none of them have been cloned in their entirety and characterized.

#### ABCI Subfamily

**Figure 9:**
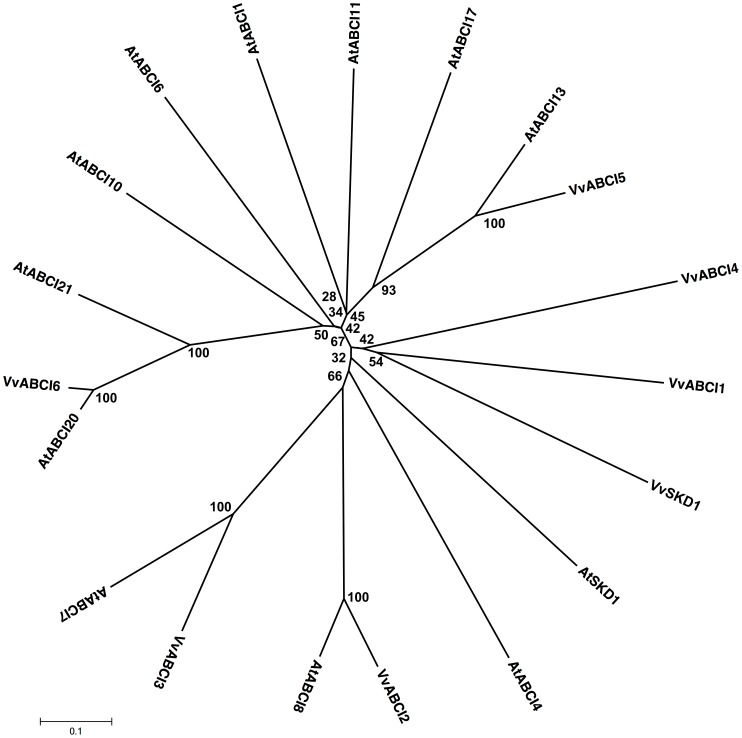
Phylogenetic tree of ABCI (NAP) protein sequences from *Arabidopsis* and *Vitis vinifera*. The amino acid sequences of all *Arabidopsis* ABCI (NAP) proteins and those of *Vitis vinifera* were aligned using the MUSCLE program and subjected to phylogenetic analysis by the distance with neighborjoining method using MEGA5 programme. Accession numbers for Arabidopsis sequences are AtABCI8 (NP_192386.1), AtABCI21 (NP_199224.1), AtABCI17 (NP_176961.1), AtABCI19 (NP_563694.1), AtABCI7 (NP_564404.1), AtABCI6 (NP_187678.1), AtABCI20 (NP_195847.1), AtABCI1 (NP_176516.1), AtABCI13 (NP_564850.1), AtABCI10 (NP_195072.2) and AtABCI11 (NP_196914.1). AtSKD1 (AEC08019.1) and VvSKD1 (XP_002266534.1) were used as outgroups.

The ABCI subfamily consists of ABC proteins with a single NBD that has similarity to prokaryotic soluble ABC proteins and is designated as non-intrinsic ABC proteins (NAPs). The *Arabidopsis* genome contains 15 members of this subfamily, whereas the rice genome has 10 members [14], [15]. Recently AtNAP8 and AtNAP15 were reassigned to AtABCB and AtABCE subfamilies, respectively in TAIR In addition, both AtNAP5 and AtNAP12 were identified as fragments of AtABCC and AtABCG subfamilies, respectively [15].We identified 6 ORFs showing the strongest similarity to the 11 putative NAPs from *A. thaliana* (Fig. 9). Members of the NAP subfamily in the *Vitis* genome contain only a single NBD and range from 329 to 511 amino acid residues (Table S1). The members of the NAP subfamily in *Vitis* share 1–18% similarity with each other and are distributed on different chromosomes (Fig. 9, Table S1). We identified orthologs for 4 of the *Vitis* NAPs with more than 80% identity from different plant species (Table S3).

These transporters have not yet been functionally characterized in plants. However, AtNAP1 (alias LAF6) is known to be a component of the plastid “mobilization of sulfur” system that is responsible for the biogenesis and repair of iron-sulfur clusters [81]. An interaction between AtNAP1 and AtNAP7 has been demonstrated [81], [82]. There are currently 70 ESTs for all of these transporters in various tissues in *V. vinifera* (Table S10).

### SMC Subfamily

**Figure 10:**
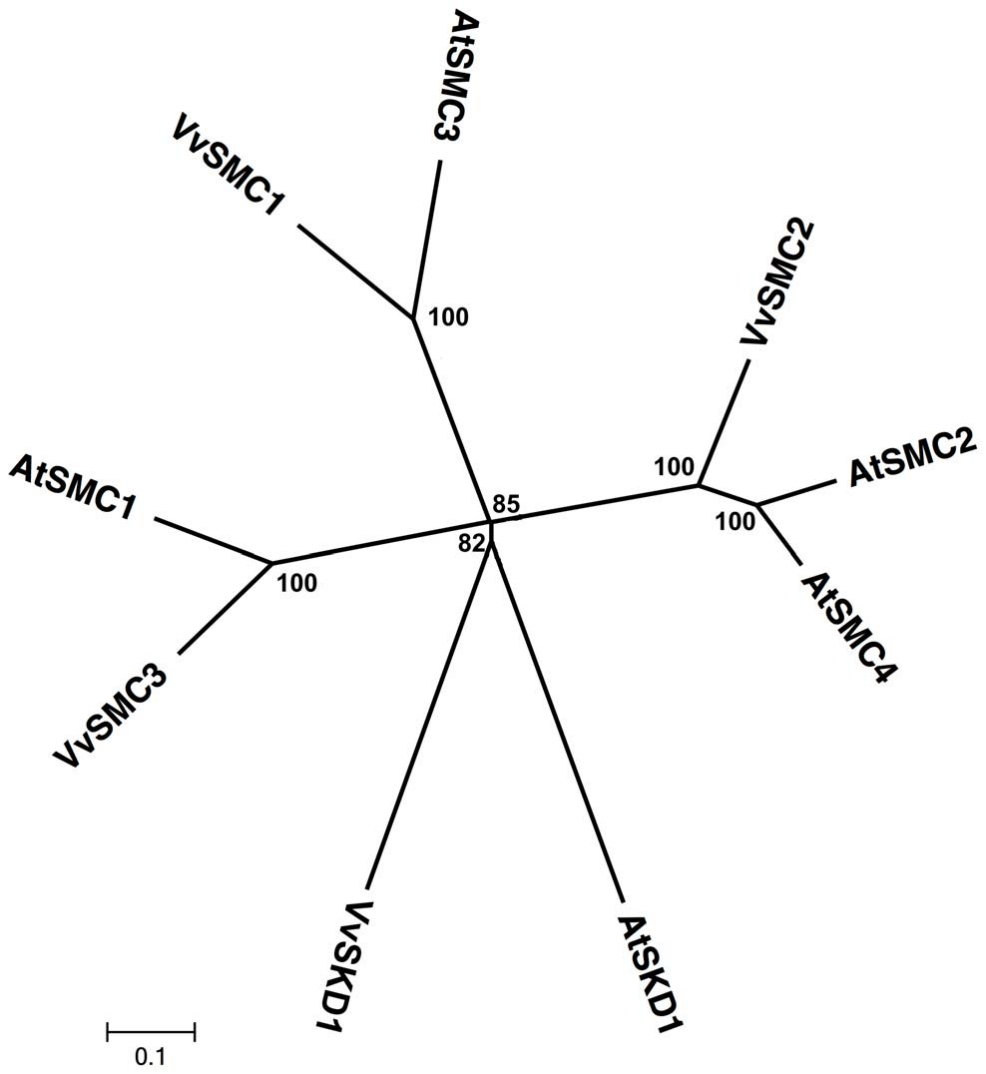
Phylogenetic tree of SMC protein sequences from *Arabidopsis* and *Vitis vinifera*. The amino acid sequences of all *Arabidopsis* SMC proteins and those of *Vitis vinifera* were aligned using the MUSCLE program and subjected to phylogenetic analysis by the distance with neighborjoining method using MEGA5 programme. Accession numbers for Arabidopsis sequences are AtSMC1 (CAB77587), AtSMC2 (CAB61972.1), AtSMC3 (BAB10693.1), AtSMC4 (BAB11491.1). AtSKD1 (AEC08019.1) and VvSKD1 (XP_002266534.1) were used as outgroups.

SMC proteins are not ordinarily classified as ABC proteins because they lack an ABC signature motif between the Walker A and the Walker B motifs. Both the *Arabidopsis* genome and the rice genome have 4 members of this subfamily [14], [15]. The *Vitis* genome contains 3 ORFs that encode putative SMC proteins with strong similarity to the 4 putative NAPs from *A. thaliana* (Fig. 10; Table S1), and they all contain an ABC signature motif between the Walker A and the Walker B motifs. *Vitis* SMCs have orthologs from *P. trichocarpa* and *R. communis* with more than 80% similarity (Table S3). The SMCs have functions in chromatin condensation, gene dosage compensation, and sister chromatin adhesion [83]. There are currently 13 ESTs for all of the *Vitis* SMCs in various tissues (Table S11). There is no report on the functions of SMC proteins in plants.

This work represents the first complete inventory of ABC transporters in *V. vinifera*. The identification of *Vitis* ABC transporters and their comparative analysis with the *Arabidopsis* ABC transporters revealed a strong conservation between the 2 species. In this report, we identified 135 ORFs encoding ABC proteins in *V. vinifera* using a bioinformatics approach. One of the most remarkable characteristics of the *V. vinifera* ABC proteins is its size. It is the largest family of ABC proteins reported to date with 135 members. Another remarkable characteristic of the *Vitis* ABC protein inventory is its large group of full-size transporters, including ABCB, ABCC, and ABCG subfamily members. This inventory could help elucidate the biological and physiological functions of these transporters from *V. vinifera*.

## Supporting Information

Table S1 **Summary of the** ***Vitis*** **ABC proteins.** The identified open reading frames (ORFs) are classified into 13 subfamilies, whose nomenclature is represented according to both Sanchez-Fernandez et al. (2001) and Verrier et al. (2008). The chromosomal (Chr) locations of the ORFs, the total number of ORFs for each category on each chromosome and in the whole genome, and the total numbers of full-size molecule and half-size molecule transporters and proteins lacking contiguous transmembrane domains (TMDs) (“soluble” proteins) are shown.
(DOC)

Table S2 **Detailed inventory of** ***Vitis*** **ABC proteins and their genes.** Columns 1–16 contain the protein acronym (Name), topology (number and orientation of nucleotide-binding folds [NBFs] and transmembrane domains [TMDs]), coding sequence (CDS), *Vitis* proteome 12× ID, GenBank ID, chromosome location (Chr), gene length, number of introns and exons, open reading frame (ORF) length, protein length, estimates of molecular weight, and pI of the protein for each gene are given.
(DOC)

Table S3 **Orthologs of** ***Vitis*** **ABC proteins identified in diverse plant species.** Columns 1–6 contain the protein name represented according to both Sanchez-Fernandez et al. (2001) and Verrier et al. (2008), *Vitis* proteome 12× ID, GenBank ID, species, percentage identity (%ID), UniprotKB ID.
(DOC)

Table S4 **Expressed sequence tags (ESTs) identified for the ABCA (ATH and AOH) subfamily in** ***Vitis vinifera*** **.** The protein name, *Vitis* proteome 12× ID, GenBank ID, EST name, cultivar/tissue type, and development stage are given for each gene.
(DOC)

Table S5 **Expressed sequence taqs (ESTs) identified for ABCB (MDR, TAP and ATM) subfamily in** ***Vitis vinifera*** **.** The protein name, *Vitis* proteome 12x ID, GenBank ID, EST name, cultivar/tissue type, and development stage are given for each gene.
(DOC)

Table S6 **Expressed sequence taqs (ESTs) identified for ABCC (MRP) subfamily in** ***Vitis vinifera*** **.** The protein name, *Vitis* proteome 12x ID, GenBank ID, EST name, cultivar/tissue type, and development stage are given for each gene.
(DOC)

Table S7 **Expressed sequence taqs (ESTs) identified for ABCD (PMP) and ABCE (RLI) subfamilies in** ***Vitis vinifera*** **.** The protein name, *Vitis* proteome 12x ID, GenBank ID, EST name, cultivar/tissue type, and development stage are given for each gene.
(DOC)

Table S8 **Expressed sequence taqs (ESTs) identified for ABCF (GCN) subfamily in** ***Vitis vinifera*** **.** The protein name, *Vitis* proteome 12x ID, GenBank ID, EST name, cultivar/tissue type, and development stage are given for each gene.
(DOC)

Table S9 **Expressed sequence taqs (ESTs) identified for ABCG (WBC and PDR) subfamily in** ***Vitis vinifera*** **.** The protein name, *Vitis* proteome 12x ID, GenBank ID, EST name, cultivar/tissue type, and development stage are given for each gene.
(DOC)

Table S10 **Expressed sequence taqs (ESTs) identified for ABCI (NAP) subfamily in** ***Vitis vinifera*** **.** The protein name, *Vitis* proteome 12x ID, GenBank ID, EST name, cultivar/tissue type, and development stage are given for each gene.
(DOC)

Table S11 **Expressed sequence taqs (ESTs) identified for SMC proteins in** ***Vitis vinifera*** **.** The protein name, *Vitis* proteome 12x ID, GenBank ID, EST name, cultivar/tissue type, and development stage are given for each gene.
(DOC)

## References

[1] Young J, Holland IB (1999) ABC transporters: bacterial exporters-revisited five years on. Biochimica et Biophysica Acta (BBA) - Biomembranes 1461: 177–200. 10581355 10.1016/s0005-2736(99)00158-3

[2] Higgins CF (1992) ABC Transporters: from microorganisms to man. Annual Review of Cell Biology 8: 67–113. 10.1146/annurev.cb.08.110192.000435 1282354

[3] Martinoia E, Klein M, Geisler M, Bovet L, Forestier C, et al (2002) Multifunctionality of plant ABC transporters - more than just detoxifiers. Planta 214: 345–355. 11855639 10.1007/s004250100661

[4] Bunting KD (2002) ABC Transporters as phenotypic markers and functional regulators of stem cells. STEM CELLS 20: 274–274. 10.1002/stem.200011 11796918

[5] Ames GF-L, Mimura C, Shyamala V (1990) Bacterial periplasmic permeases belong to a family of transport proteins operating from *Escherichia coli* to human: Traffic ATPases. FEMS Microbiol Rev 75: 429–446. 10.1111/j.1574-6968.1990.tb04110.x 2147378

[6] Fath MJ, Kolter R (1993) ABC transporters: bacterial exporters. Microbiol Rev 57: 995–1017. 8302219 10.1128/mr.57.4.995-1017.1993 PMC372944

[7] Higgins CF, Linton KJ (2004) The ATP switch model for ABC transporters. Nat Struct Mol Biol 11: 918–926. 15452563 10.1038/nsmb836

[8] Bianchet MA, Ko YH, Amzel LM, Pedersen PL (1997) Modeling of nucleotide binding domains of ABC transporter proteins based on a F1-ATPase/recA topology: structural model of the nucleotide binding domains of the cystic fibrosis transmembrane conductance regulator (CFTR). Journal of Bioenergetics and Biomembranes 29: 503–524. 9511935 10.1023/a:1022443209010

[9] Walker JE, Sarante M, Runswick MJ, Gay NJ (1982) Distantly related sequences in the alpha- and beta-subunits of ATP synthase, myosin, kinases and other ATP-requiring enzymes and a common nucleotide binding fold. EBMO Journal 1: 945–951. 10.1002/j.1460-2075.1982.tb01276.x PMC553140 6329717

[10] Dean M, Allikmets R (1995) Evolution of ATP-binding cassette transporter genes. Current Opinion in Genetics & Development 5: 779–785. 8745077 10.1016/0959-437x(95)80011-s

[11] Hyde SC, Emsley P, Hartshorn MJ, Mimmack MM, Gileadi U, et al (1990) Structural model of ATP-binding proteing associated with cystic fibrosis, multidrug resistance and bacterial transport. Nature 346: 362–365. 1973824 10.1038/346362a0

[12] Theodoulou FL (2000) Plant ABC transporters. Biochim Biophys Acta 1465: 79–103. 10748248 10.1016/s0005-2736(00)00132-2

[13] Verrier PJ, Bird D, Burla B, Dassa E, Forestier C, et al (2008) Plant ABC proteins ‚an unified nomenclature and updated inventory. Trends in Plant Science 13: 151–159. 18299247 10.1016/j.tplants.2008.02.001

[14] Sanchez-Fernandez R, Davies TGE, Coleman JOD, Rea PA (2001) The *Arabidopsis thaliana* ABC protein superfamily, a complete inventory. Journal of Biological Chemistry 276: 30231–30244. 11346655 10.1074/jbc.M103104200

[15] Garcia O, Bouige P, Forestier C, Dassa E (2004) Inventory and comparative analysis of Rice and *Arabidopsis* ATP-binding Cassette (ABC) Systems. Journal of Molecular Biology 343: 249–265. 15381434 10.1016/j.jmb.2004.07.093

[16] Sugiyama T, Kantake N, Wu Y, Kowalczykowski SC (2006) Rad52-mediated DNA annealing after Rad51-mediated DNA strand exchange promotes second ssDNA capture. EMBO J 25: 5539–5548. 17093500 10.1038/sj.emboj.7601412 PMC1679760

[17] Jasinski M, Ducos E, Martinoia E, Boutry M (2003) The ATP-binding cassette transporters: structure, function, and gene family comparison between Rice and *Arabidopsis* . Plant Physiology 131: 1169–1177. 12644668 10.1104/pp.102.014720 PMC1540298

[18] Stein Mn, Dittgen J, Sanchez-Rodriguez C, Hou B-H, Molina A, et al (2006) *Arabidopsis* PEN3/PDR8, an ATP-binding cassette transporter, contributes to nonhost resistance to inappropriate pathogens That Enter by direct penetration. The Plant Cell Online 18: 731–746. 10.1105/tpc.105.038372 PMC1383646 16473969

[19] Rea PA (2007) Plant ATP-binding cassette transporters. Annual Review of Plant Biology 58: 347–375. 10.1146/annurev.arplant.57.032905.105406 17263663

[20] Lee M, Lee K, Lee J, Noh EW, Lee Y (2005) AtPDR12 Contributes to lead resistance in *Arabidopsis.* . Plant Physiology 138: 827–836. 15923333 10.1104/pp.104.058107 PMC1150400

[21] Moons A (2003) Ospdr9, which encodes a PDR-type ABC transporter, is induced by heavy metals, hypoxic stress and redox perturbations in rice roots. FEBS Letters 553: 370–376. 14572653 10.1016/s0014-5793(03)01060-3

[22] Campbell EJ, Schenk PM, Kazan K, Penninckx IAMA, Anderson JP, et al (2003) Pathogen-responsive expression of a putative ATP-binding cassette transporter gene conferring resistance to the diterpenoid sclareol is regulated by multiple defense signaling pathways in *Arabidopsis* . Plant Physiology 133: 1272–1284. 14526118 10.1104/pp.103.024182 PMC281622

[23] Kang J, Hwang J-U, Lee M, Kim Y-Y, Assmann SM, et al (2010) PDR-type ABC transporter mediates cellular uptake of the phytohormone abscisic acid. Proceedings of the National Academy of Sciences 107: 2355–2360. 10.1073/pnas.0909222107 PMC2836657 20133880

[24] Pighin JA, Zheng H, Balakshin LJ, Goodman IP, Western TL, et al (2004) Plant cuticular lipid export requires an ABC transporter. Science 306: 702–704. 15499022 10.1126/science.1102331

[25] Bird D, Beisson F, Brigham A, Shin J, Greer S, et al (2007) Characterization of *Arabidopsis* ABCG11/WBC11, an ATP binding cassette (ABC) transporter that is required for cuticular lipid secretion†. The Plant Journal 52: 485–498. 17727615 10.1111/j.1365-313X.2007.03252.x

[26] Panikashvili D, Savaldi-Goldstein S, Mandel T, Yifhar T, Franke RB, et al (2007) The *Arabidopsis* DESPERADO/AtWBC11 transporter is required for cutin and wax secretion. Plant Physiology 145: 1345–1360. 17951461 10.1104/pp.107.105676 PMC2151707

[27] Ukitsu H, Kuromori T, Toyooka K, Goto Y, Matsuoka K, et al (2007) Cytological and biochemical analysis of COF1, an *Arabidopsis* mutant of an ABC transporter gene. Plant and Cell Physiology 48: 1524–1533. 17971336 10.1093/pcp/pcm139

[28] Mentewab A, Stewart CN (2005) Overexpression of an *Arabidopsis thaliana* ABC transporter confers kanamycin resistance to transgenic plants. Nat Biotech 23: 1177–1180. 10.1038/nbt1134 16116418

[29] Kuromori T, Miyaji T, Yabuuchi H, Shimizu H, Sugimoto E, et al (2010) ABC transporter AtABCG25 is involved in abscisic acid transport and responses. Proceedings of the National Academy of Sciences 107: 2361–2366. 10.1073/pnas.0912516107 PMC2836683 20133881

[30] Geisler M, Murphy AS (2006) The ABC of auxin transport: The role of p-glycoproteins in plant development. FEBS Letters 580: 1094–1102. 16359667 10.1016/j.febslet.2005.11.054

[31] Kaneda M, Schuetz M, Lin BSP, Chanis C, Hamberger B, et al. (2011) ABC transporters coordinately expressed during lignification of *Arabidopsis* stems include a set of ABCBs associated with auxin transport. Journal of Experimental Botany. 10.1093/jxb/erq416 PMC3060696 21239383

[32] Klein M, Burla B, Martinoia E (2006) The multidrug resistance-associated protein (MRP/ABCC) subfamily of ATP-binding cassette transporters in plants. FEBS Letters 580: 1112–1122. 16375897 10.1016/j.febslet.2005.11.056

[33] Jaillon O, Aury J, Noel B, Policriti A, Clepet C, et al (2007) The grapevine genome sequence suggests ancestral hexaploidization in major angiosperm phyla. Nature 449: 463–467. 17721507 10.1038/nature06148

[34] Dean M, Rzhetsky A, Allikmets R (2001) The human ATP-binding cassette (ABC) transporter superfamily. Genome Research 11: 1156–1166. 11435397 10.1101/gr.184901

[35] Altschul S, Gish W, Miller W, Myers E, Lipman D (1990) Basic local alignment search tool. Journal of Molecular Biology 215: 403–410. 2231712 10.1016/S0022-2836(05)80360-2

[36] Marchler-Bauer A, Bryant SH (2004) CD-Search: protein domain annotations on the fly. Nucleic Acids Research 32: W327–W331. 15215404 10.1093/nar/gkh454 PMC441592

[37] Marchler-Bauer A, Anderson JB, Chitsaz F, Derbyshire MK, DeWeese-Scott C, et al (2009) CDD: specific functional annotation with the Conserved Domain Database. Nucleic Acids Research 37: D205–D210. 18984618 10.1093/nar/gkn845 PMC2686570

[38] Saitou N, Nei M (1987) The neighbor-joining method: a new method for reconstructing phylogenetic trees. Molecular Biology and Evolution 4: 406–425. 3447015 10.1093/oxfordjournals.molbev.a040454

[39] Thompson JD, Higgins DG, Gibson TJ (1994) CLUSTAL W: improving the sensitivity of progressive multiple sequence alignment through sequence weighting, position-specific gap penalties and weight matrix choice. Nucleic Acids Research 22: 4673–4680. 7984417 10.1093/nar/22.22.4673 PMC308517

[40] Edgar RC (2004) MUSCLE: multiple sequence alignment with high accuracy and high throughput. Nucleic Acids Research 32: 1792–1797. 15034147 10.1093/nar/gkh340 PMC390337

[41] Tamura K, Peterson D, Peterson N, Stecher G, Nei M, et al.. (2011) MEGA5: Molecular Evolutionary Genetics Analysis using Maximum Likelihood, Evolutionary Distance, and Maximum Parsimony Methods. Molecular Biology and Evolution. 10.1093/molbev/msr121 PMC3203626 21546353

[42] Datta RS, Meacham C, Samad B, Neyer C, Sjölander K (2009) Berkeley PHOG: PhyloFacts orthology group prediction web server. Nucleic Acids Research 37: W84–W89. 19435885 10.1093/nar/gkp373 PMC2703887

[43] Moreno-Hagelsieb G, Latimer K (2008) Choosing BLAST options for better detection of orthologs as reciprocal best hits. Bioinformatics 24: 319–324. 18042555 10.1093/bioinformatics/btm585

[44] Lawn RM, Wade DP, Garvin MR, Wang X, Schwartz K, et al (1999) The tangier disease gene product ABC1 controls the cellular apolipoprotein-mediated lipid removal pathway. J Clinic Invest 14: R25–R31. 10.1172/JCI8119 PMC481052 10525055

[45] Oram JF, Vaughan A (2000) ABCA1-mediated transport of cellular cholesterol and phospholipids to HDL apolipoproteins. Curr Opin Lipidol 11: 253–260. 10882340 10.1097/00041433-200006000-00005

[46] Allikmets R, Raskind WH, Hutchinson A, Schueck ND, Dean M, et al (1999) Mutation of a putative mitochondrial iron transporter gene (ABC7) in X-linked sideroblastic anemia and ataxia (XLSA/A). Human Molecular Genetics 8: 743–749. 10196363 10.1093/hmg/8.5.743

[47] Sun H, Nathans J (1997) Stargardt’s ABCR is localized to the disc membrane of retinal rod outer segments. Nat Genet 17: 15–16. 9288089 10.1038/ng0997-15

[48] Maathuis FJM, Filatov V, Herzyk P, Krijger GC, Axelsen KB, et al (2003) Transcriptome analysis of root transporters reveals participation of multiple gene families in the response to cation stress. The Plant Journal 35: 675–692. 12969422 10.1046/j.1365-313x.2003.01839.x

[49] Gottesman M, Ambudkar S (2001) Overview: ABC transporters and human disease. J Bioenerg Biomembr 33: 453–458. 11804186 10.1023/a:1012866803188

[50] Windsor B, Roux SJ, Lloyd A (2003) Multiherbicide tolerance conferred by AtPgp1 and apyrase overexpression in *Arabidopsis thaliana* . Nat Biotech 21: 428–433. 10.1038/nbt809 12640467

[51] Decottignies A, Goffeau A (1997) Complete inventory of the yeast ABC proteins. Nat Genet 15: 137–145. 9020838 10.1038/ng0297-137

[52] Reits EAJ, Vos JC, Gromme M, Neefjes J (2000) The major substrates for TAP *in vivo* are derived from newly synthesized proteins. Nature 404: 774–778. 10783892 10.1038/35008103

[53] Kushnir S, Babiychuk E, Storozhenko S, Davey MW, Papenbrock J, et al (2001) A Mutation of the Mitochondrial ABC Transporter Sta1 Leads to Dwarfism and Chlorosis in the Arabidopsis Mutant starik. The Plant Cell Online 13: 89–100. 10.1105/tpc.13.1.89 PMC102216 11158531

[54] Teschner J, Lachmann N, Schulze J, Geisler M, Selbach K, et al (2010) A Novel Role for Arabidopsis Mitochondrial ABC Transporter ATM3 in Molybdenum Cofactor Biosynthesis. The Plant Cell Online 22: 468–480. 10.1105/tpc.109.068478 PMC2845412 20164445

[55] Kim DY, Bovet L, UGent SK, Noh E, Martinoia E, et al (2006) AtATM3 is involved in heavy metal resistance in Arabidopsis. Plant Physiology 140: 922–932. 16461380 10.1104/pp.105.074146 PMC1400565

[56] Babiychuk E, Fuangthong M, Van Montagu M, Inze D, Kushnir S (1997) Efficient gene tagging in Arabidopsis thaliana using a gene trap approach. Proceedings of the National Academy of Sciences 94: 12722–12727. 10.1073/pnas.94.23.12722 PMC25099 9356517

[57] Yamaguchi H, Nishizawa NÄ, Nakanishi H, Mori S (2002) IDI7, a new iron‚Äêregulated ABC transporter from barley roots, localizes to the tonoplast. Journal of Experimental Botany 53: 727–735. 11886893 10.1093/jexbot/53.369.727

[58] Klein I, Sarkadi B, Varadi A (1999) An inventory of the human ABC proteins. Biochim Biophys Acta 1461: 237–262. 10581359 10.1016/s0005-2736(99)00161-3

[59] Schulz B, Kolukisaoglu HU (2006) Genomics of plant ABC transporters: The alphabet of photosynthetic life forms or just holes in membranes? FEBS Letters 580: 1010–1016. 16413546 10.1016/j.febslet.2006.01.002

[60] Katzmann DJ, Hallstrom TC, Voet M, Wysock W, Golin J, et al (1995) Expression of an ATP-binding cassette Rice ABC Proteins Inventory 263 transporter-encoding gene (YOR1) is required for oligomycin resistance in Saccharomyces cerevisiae. Mol Cell Biol 15: 6875–6883. 8524254 10.1128/mcb.15.12.6875 PMC230942

[61] Szczypka MS, Wemmie JA, Moye-Rowley WS, Thiele DJ (1994) A yeast metal resistance protein similar to human cystic fibrosis transmembrane conductance regulator (CFTR) and multidrug resistance-associated protein. Journal of Biological Chemistry 269: 22853–22857. 7521334

[62] Zolman BK, Silva ID, Bartel B (2001) The Arabidopsis pxa1 mutant is defective in an ATP-binding cassette transporter-like protein required for peroxisomal fatty acid beta-oxidation. Plant Physiology 127: 1266–1278. 11706205 PMC129294

[63] Hayashi M, Nito K, Takei-Hoshi R, Yagi M, Kondo M, et al (2002) Ped3p is a peroxisomal ATP-binding cassette transporter that might supply substrates for fatty acid beta-oxidation. Plant and Cell Physiology 43: 1–11. 11828016 10.1093/pcp/pcf023

[64] Footitt S, Slocombe SP, Larner V, Kurup S, Wu Y, et al (2002) Control of germination and lipid mobilization by COMATOSE, the *Arabidopsis* homologue of human ALDP. EMBO J 21: 2912–2922. 12065405 10.1093/emboj/cdf300 PMC125387

[65] Footitt S, Marquez J, Schmuths H, Baker A, Theodoulou FL, et al (2006) Analysis of the role of COMATOSE and peroxisomal beta-oxidation in the determination of germination potential in *Arabidopsis* . Journal of Experimental Botany 57: 2805–2814. 16844736 10.1093/jxb/erl045

[66] Footitt S, Dietrich D, Fait A, Fernie AR, Holdsworth MJ, et al (2007) The COMATOSE ATP-binding cassette transporter is required for full fertility in *Arabidopsis* . Plant Physiology 144: 1467–1480. 17468211 10.1104/pp.107.099903 PMC1914130

[67] Bisbal C, Martinand C, Silhol M, Lebleu B, Salehzada T (1995) Cloning and characterization of a RNase L inhibitor. Journal of Biological Chemistry 270: 13308–13317. 7539425 10.1074/jbc.270.22.13308

[68] Bairoch A (1992) PROSITE: a dictionary of sites and patterns in proteins. Nucleic Acids Research 20. 10.1093/nar/20.suppl.2013 PMC333978 1598232

[69] Sarmiento C, Nigul L, Kazantseva J, Buschmann M, Truve E (2006) AtRLI2 is an endogenous suppressor of RNA silencing. Plant Molecular Biology 61: 153–163. 16786298 10.1007/s11103-005-0001-8

[70] Janvilisri T, Venter H, Shahi S, Reuter G, Balakrishnan L, et al.. (2003) Sterol transport by human breast cancer resistance protein (ABCG2) expressed in Lactococcus lactis. Journal of Biological Chemistry: 20645–20651. 10.1074/jbc.M301358200 12668685

[71] Chen H, Rossier C, Lalioti MD, Lynn A, Chakravarti A, et al (1996) Cloning of the cDNA for a human homologue of the *Drosophila* white gene and mapping to chromosome 21q22.3. Am J Hum Genet 59: 66–75. 8659545 PMC1915121

[72] Klucken J, Büchler C, Orso E, Kaminski WE, Porsch-Özcürümez M, et al (2000) ABCG1 (ABC8), the human homolog of the Drosophila white gene, is a regulator of macrophage cholesterol and phospholipid transport. Proceedings of the National Academy of Sciences 97: 817–822. 10.1073/pnas.97.2.817 PMC15414 10639163

[73] van den Brûle S, Smart C (2002) The plant PDR family of ABC transporters. Planta 216: 95–106. 12430018 10.1007/s00425-002-0889-z

[74] Crouzet J, Trombik T, Fraysse ÖS, Boutry M (2006) Organization and function of the plant pleiotropic drug resistance ABC transporter family. FEBS Letters 580: 1123–1130. 16506311 10.1016/j.febslet.2005.12.043

[75] Bauer B, Wolfger H, Kuchler K (1999) Inventory and function of yeast ABC proteins: about sex, stress, pleiotropic drug and heavy metal resistance. Biochim Biophys Acta 1461: 217–236. 10581358 10.1016/s0005-2736(99)00160-1

[76] Rogers B, Decottignies A, Kolaczkowski M, Carvajal E, Balzi E, et al (2001) The pleiotropic drug ABC transporters from *Saccharomyces cerevisiae* . J Mol Biotechnol 3: 207–214. 11321575

[77] Schoonbeek H, Sorbo GD, Waard MD (2001) The ABC transporter BcatrB affects the sensitivity of Botrytis cinerea to the phytoalexin resveratrol and the fungicide fenpiclonil. Mol Plant Microb 14: 562–571. 10.1094/MPMI.2001.14.4.562 11310744

[78] Smart CC, Fleming AJ (1996) Hormonal and environmental regulation of a plant PDR5-like ABC transporter. Journal of Biological Chemistry 271: 19351–19357. 8702621 10.1074/jbc.271.32.19351

[79] Moons A (2008) Transcriptional profiling of the PDR gene family in rice roots in response to plant growth regulators, redox perturbations and weak organic acid stresses. Planta 229: 53–71. 18830621 10.1007/s00425-008-0810-5

[80] Stukkens Y, Bultreys A, Grec S, Trombik T, Vanham D, et al (2005) NpPDR1, a pleiotropic drug resistance-type ATP-binding cassette transporter from *Nicotiana plumbaginifolia*, plays a major role in plant pathogen defense. Plant Physiology 139: 341–352. 16126865 10.1104/pp.105.062372 PMC1203383

[81] Xu XM, Adams S, Chua N-H, Møller SG (2005) AtNAP1 represents an atypical SufB protein in *Arabidopsis* plastids. Journal of Biological Chemistry 280: 6648–6654. 15611066 10.1074/jbc.M413082200 PMC1401503

[82] Xu XM, Møller SG (2004) AtNAP7 is a plastidic SufC-like ATPbinding cassette/ATPase essential for *Arabidopsis* embryogenesis. Proc Natl Acad Sci USA 101: 9143–9148. 15184673 10.1073/pnas.0400799101 PMC428487

[83] Strunnikov A, Jessberger R (1999) Structural maintenance of chromosomes (SMC) proteins: conserved molecular properties for multiple biological functions. Eur J Biochem 263: 6–13. 10429180 10.1046/j.1432-1327.1999.00509.x

