# Supplementary material for: Whole-Genome Survey of the Putative ATP-Binding Cassette Transporter Family Genes in Vitis vinifera
Source: PLoS One. 2013 Nov 11;8(11):e78860. doi: 10.1371/journal.pone.0078860 (PMC3823996; doi:10.1371/journal.pone.0078860)
Supplement: Table S1 — Summary of the Vitis ABC proteins. The identified open reading frames (ORFs) are classified into 13 subfamilies, whose nomenclature is represented according to both Sanchez-Fernandez et al. (2001) and Verrier et al. (2008). The chromosomal (Chr) locations of the ORFs, the total number of ORFs for each category on each chromosome and in the whole genome, and the total numbers of full-size molecule and half-size molecule transporters and proteins lacking contiguous transmembrane domains (TMDs) (“soluble” proteins) are shown. (DOC) [file pone.0078860.s001.doc]

**Table S1.** Detailed inventory of *Vitis* ABC proteins and their genes. Columns 1–16 contain the protein acronym (Name), topology (number and orientation of nucleotide-binding folds [NBFs] and transmembrane domains [TMDs]), coding sequence (CDS), *Vitis* proteome 12× ID, GenBank ID, chromosome location (Chr), gene length, number of introns and exons, open reading frame (ORF) length, protein length, estimates of molecular weight, and pI of the protein for each gene are given.

| **Sanchez-Fernandez**  **Subfamilyname** | **HGNC***  **Subfamily name** | **Topology** | **CDS** | **12X *Vitis vinifera* ID** | **NCBI GenBank ID** | **Chr** | **Strand** | **Location** | **Genomic**  **Copy**  **Length** | **Exon** | **Intron** | **Transcript**  **Length** | **AA** | **pI** | **mW (Da)** |
| --- | --- | --- | --- | --- | --- | --- | --- | --- | --- | --- | --- | --- | --- | --- | --- |
| **AOH** | **ABCAs** |  |  |  |  |  |  |  |  |  |  |  |  |  |  |
| VvAOH1 | VvABCA1 | (TMD-NBD)2 | Full | GSVIVT01033258001 | CBI29824.3 | 8 | + | 22099754 to 22129500 | 29747 | 43 | 42 | 6006 | 2001 | 8,07 | 223551,51 |
| **ATHs** |  |  |  |  |  |  |  |  |  |  |  |  |  |  |  |
| VvATH1 | VvABCA2 | TMD-NBD | Full | GSVIVT01008040001 | CBI15253.3 | 17 | + | 6396463 to 6408689 | 12227 | 19 | 18 | 2787 | 928 | 8,02 | 103106,71 |
| VvATH2 | VvABCA3 | TMD-NBD | Full | GSVIVT01008042001 | CBI15254.3 | 17 | - | 6379499 to 6387517 | 8019 | 16 | 15 | 2877 | 958 | 6,67 | 106822,30 |
| VvATH3 | VvABCA4 | TMD-NBD | Full | GSVIVT01025902001 | CBI39216.3 | 18 | + | 27367072 to 27377291 | 10220 | 17 | 16 | 2211 | 736 | 8,23 | 84023,60 |
| VvATH4 | VvABCA5 | TMD-NBD | Full | GSVIVT01033860001 | CBI30307.3 | 8 | + | 17187414 to 17198226 | 10813 | 12 | 11 | 2172 | 723 | 9,41 | 79729,51 |
| **MDRs** | **ABCBs** |  |  |  |  |  |  |  |  |  |  |  |  |  |  |
| VvMDR1 | VvABCB1 | (TMD-NBD)2 | Full | GSVIVT01000580001 | CBI26020.3 | 5 | + | 2719817 to 2732762 | 12946 | 34 | 33 | 6661 | 2226 | 8,80 | 112615,43 |
| VvMDR2 | VvABCB2 | (TMD-NBD)2 | Full | GSVIVT01007586001 | CBI14902.3 | 17 | - | 11622796 to 11629827 | 7032 | 11 | 10 | 3276 | 1091 | 6,39 | 118365,68 |
| VvMDR3 | VvABCB3 | (TMD-NBD)2 | Full | GSVIVT01009946001 | CBI19899.3 | 18 | + | 12710695 to 12716741 | 6047 | 16 | 15 | 3138 | 1045 | 9,10 | 113699,31 |
| VvMDR4 | VvABCB4 | (TMD-NBD)2 | Full | GSVIVT01011381001 | CBI22189.3 | 14 | - | 29196604 to 29203917 | 7314 | 15 | 14 | 3267 | 1088 | 6,31 | 117680,14 |
| VvMDR5 | VvABCB5 | (TMD-NBD)2 | Full | GSVIVT01013125001 | CBI33860.3 | 2 | - | 7963836 to 7972650 | 8815 | 12 | 11 | 3732 | 1243 | 8,61 | 136419,15 |
| VvMDR6 | VvABCB6 | (TMD-NBD)2 | Full | GSVIVT01014625001 | CBI20527.3 | 19 | + | 5812018 to 5816845 | 4828 | 17 | 16 | 2931 | 976 | 8,60 | 105974,61 |
| VvMDR7 | VvABCB7 | (TMD-NBD)2 | Full | GSVIVT01015306001 | CBI28004.3 | 11 | + | 2530672 to 2536481 | 5810 | 11 | 10 | 3032 | 1009 | 8,25 | 110394,28 |
| VvMDR8 | VvABCB8 | (TMD-NBD)2 | Full | GSVIVT01016617001 | CBI31730.3 | 3 | + | 2387669 to 2421339 | 3367 | 15 | 14 | 3948 | 1315 | 6,25 | 145993,94 |
| VvMDR9 | VvABCB9 | (TMD-NBD)2 | Full | GSVIVT01016706001 | CBI35827.3 | 9 | + | 502887 to 509976 | 7090 | 15 | 14 | 3423 | 1140 | 9,45 | 124708,56 |
| VvMDR10 | VvABCB10 | (TMD-NBD)2 | Full | GSVIVT01017696001 | CBI26020.3 | 5 | + | 2719817 to 2732762 | 12946 | 34 | 33 | 6681 | 2226 | 8,14 | 240703,89 |
| VvMDR11 | VvABCB11 | (TMD-NBD)2 | Full | GSVIVT01021365001 | CBI30804.3 | 10 | + | 4494206 to 4505281 | 11076 | 34 | 33 | 5793 | 1930 | 8,47 | 210258,31 |
| VvMDR12 | VvABCB12 | (TMD-NBD)2 | Full | GSVIVT01021366001 | CBI30805.3 | 10 | + | 4527597 to 4542982 | 15386 | 35 | 34 | 7218 | 2405 | 8,25 | 260868,23 |
| VvMDR13 | VvABCB13 | (TMD-NBD)2 | Partial | GSVIVT01025040001 | CBI16194.3 | 6 | + | 5064960 to 5071957 | 6998 | 12 | 11 | 3411 | 1136 | 5,50 | 126262,23 |
| VvMDR14 | VvABCB14 | (TMD-NBD)2 | Full | GSVIVT01028256001 | CBI37062.3 | 7 | - | 4974180 to 4981456 | 7277 | 17 | 16 | 3444 | 1147 | 8,11 | 124038,22 |
| VvMDR15 | VvABCB15 | (TMD-NBD)2 | Full | GSVIVT01032578001 | CBI35014.3 | 14 | + | 28532840 to 28538792 | 5953 | 9 | 8 | 3651 | 1216 | 8,81 | 132839,85 |
| VvMDR16 | VvABCB16 | (TMD-NBD)2 | Full | GSVIVT01032898001 | CBI21202.3 | 4 | + | 28532840 to 28538792 | 17949 | 35 | 34 | 3798 | 1265 | 8,97 | 136895,89 |
| VvMDR17 | VvABCB17 | (TMD-NBD)2 | Partial | GSVIVT01033645001 | CBI30138.3 | 8 | + | 18985319 to 18992118 | 6800 | 16 | 15 | 3000 | 999 | 9,11 | 109741,35 |
| VvMDR18 | VvABCB18 | (TMD-NBD)2 | Full | GSVIVT01036801001 | CBI24202.3 | 19 | - | 22379272 to 22383673 | 4402 | 15 | 14 | 2445 | 814 | 8,29 | 88169,92 |
| VvMDR19 | VvABCB19 | (TMD-NBD)2 | Full | GSVIVT01038687001 | CBI23123.3 | 16 | + | 21004457 to 21010493 | 6037 | 12 | 11 | 3474 | 1157 | 6,07 | 126931,33 |
| **ATM** |  |  |  |  |  |  |  |  |  |  |  |  |  |  |  |
| VvATM1 | VvABCB20 | TMD-NBD | Full | GSVIVT01024527001 | CBI15790.3 | 6 | - | 9155464 to 9213374 | 57911 | 20 | 19 | 2181 | 726 | 9,40 | 80313,32 |
| **TAPs** |  |  |  |  |  |  |  |  |  |  |  |  |  |  |  |
| VvTAP1 | VvABCB21 | TMD-NBD | Full | GSVIVT01005757001 | CBI18648.3 | Un | - | 21868386 to 21885325 | 16940 | 18 | 17 | 1764 | 587 | 8,85 | 65475,45 |
| VvTAP2 | VvABCB22 | TMD-NBD | Full | GSVIVT01008121001 | CBI15325.3 | 17 | + | 5620016 to 5627317 | 7302 | 17 | 16 | 1896 | 631 | 8,80 | 68132,59 |
| VvTAP3 | VvABCB23 | TMD-NBD | Full | GSVIVT01010634001 | CBI31850.3 | 16 | - | 15736963 to 15750200 | 13238 | 16 | 15 | 2154 | 717 | 8,91 | 77959,21 |
| VvTAP4 | VvABCB24 | TMD-NBD | Full | GSVIVT01032404001 | CBI34864.3 | 14 | - | 27051487 to 27063649 | 12163 | 18 | 17 | 1977 | 658 | 7,19 | 73033,72 |
| VvTAP5 | VvABCB25 | TMD-NBD | Full | GSVIVT01032898001 | CBI21202.3 | 14 | + | 23920911 to 23938859 | 17949 | 35 | 34 | 3798 | 1265 | 8,97 | 136895,89 |
| **MRPs** | **ABCCs** |  |  |  |  |  |  |  |  |  |  |  |  |  |  |
| VvMRP1 | VvABCC1 | (TMD-NBD)2 | Full | GSVIVT01010326001 | CBI27547.3 | 2 | - | 18345602 to 18354600 | 8999 | 11 | 10 | 1878 | 1363 | 8,79 | 152506.24 |
| VvMRP2 | VvABCC2 | (TMD-NBD)2 | Full | GSVIVT01014628001 | CBI39697.3 | 19 | - | 7925931 to 7935919 | 9989 | 17 | 16 | 3855 | 1284 | 7,97 | 143531.89 |
| VvMRP3 | VvABCC3 | (TMD-NBD)2 | Full | GSVIVT01014629001 | CBI39698.3 | 19 | - | 7936173 to 7941661 | 5489 | 14 | 13 | 2499 | 832 | 5,64 | 92523.80 |
| VvMRP4 | VvABCC4 | (TMD-NBD)2 | Full | GSVIVT01014631001 | CBI39700.3 | 19 | - | 7967803 to 7976826 | 9024 | 18 | 17 | 3063 | 1020 | 8,54 | 113843.21 |
| VvMRP5 | VvABCC5 | (TMD-NBD)2 | Full | GSVIVT01014632001 | CBI39701.3 | 19 | - | 7977107 to 7983555 | 6449 | 14 | 13 | 4068 | 1355 | 7,82 | 151844.30 |
| VvMRP6 | VvABCC6 | (TMD-NBD)2 | Full | GSVIVT01014633001 | CBI39702.3 | 19 | - | 8023417 to 8033145 | 9729 | 13 | 12 | 2397 | 798 | 5,32 | 88649.64 |
| VvMRP7 | VvABCC7 | (TMD-NBD)2 | Full | GSVIVT01015960001 | CBI25172.3 | 9 | - | 16187224 to 16273585 | 86362 | 28 | 27 | 3618 | 1205 | 6,00 | 134700.46 |
| VvMRP8 | VvABCC8 | (TMD-NBD)2 | Full | GSVIVT01016879001 | CBI35971.3 | 9 | + | 2205746 to 2225094 | 19349 | 35 | 34 | 8319 | 2772 | 8,38 | 309724.47 |
| VvMRP9 | VvABCC9 | (TMD-NBD)2 | Full | GSVIVT01016880001 | CBI35972.3 | 9 | + | 2225152 to 2233977 | 8826 | 20 | 19 | 3285 | 1094 | 7,97 | 122098.24 |
| VvMRP10 | VvABCC10 | (TMD-NBD)2 | Full | GSVIVT01018403001 | CBI16747.3 | 15 | + | 9661841 to 9670181 | 8341 | 21 | 20 | 3330 | 1109 | 5,15 | 124624.84 |
| VvMRP11 | VvABCC11 | (TMD-NBD)2 | Full | GSVIVT01019467001 | CBI34432.3 | 2 | - | 870871 to 883981 | 13111 | 26 | 25 | 4911 | 1636 | 9,05 | 183516.09 |
| VvMRP12 | VvABCC12 | (TMD-NBD)2 | Full | GSVIVT01019469001 | CBI34434.3 | 2 | - | 886847 to 894005 | 7159 | 16 | 15 | 3213 | 1070 | 5,85 | 118283.79 |
| VvMRP13 | VvABCC13 | (TMD-NBD)2 | Full | GSVIVT01019471001 | CBI34436.3 | 2 | - | 899832 to 914539 | 14708 | 17 | 16 | 3381 | 1126 | 5,77 | 125248.40 |
| VvMRP14 | VvABCC14 | (TMD-NBD)2 | Full | GSVIVT01019473001 | CBI34438.3 | 2 | - | 916178 to 932015 | 15838 | 19 | 18 | 4083 | 1360 | 7,23 | 150761.53 |
| VvMRP15 | VvABCC15 | (TMD-NBD)2 | Full | GSVIVT01019475001 | CBI34440.3 | 2 | - | 933100 to 947137 | 14038 | 19 | 18 | 3597 | 1198 | 6,29 | 133422.57 |
| VvMRP16 | VvABCC16 | (TMD-NBD)2 | Full | GSVIVT01019476001 | CBI34441.3 | 2 | - | 952858 to 960480 | 7623 | 12 | 11 | 3624 | 1207 | 6,09 | 133960.48 |
| VvMRP17 | VvABCC17 | (TMD-NBD)2 | Full | GSVIVT01021589001 | CBI30977.3 | 10 | + | 7565358 to 7609701 | 44344 | 27 | 26 | 4872 | 1623 | 6,82 | 182631.38 |
| VvMRP18 | VvABCC18 | (TMD-NBD)2 | Partial | GSVIVT01021593001 | CBI30981.3 | 10 | + | 7694908 to 7733343 | 38436 | 9 | 8 | 1068 | 355 | 8,57 | 39411,64 |
| VvMRP19 | VvABCC19 | (TMD-NBD)2 | Full | GSVIVT01021594001 | CBI30982.3 | 10 | + | 7733350 to 7752675 | 19326 | 8 | 7 | 2280 | 759 | 8,01 | 85532,78 |
| VvMRP20 | VvABCC20 | (TMD-NBD)2 | Full | GSVIVT01021595001 | CBI30983.3 | 10 | + | 7765400 to 7821944 | 56545 | 32 | 31 | 4503 | 1500 | 7,08 | 169093.47 |
| VvMRP21 | VvABCC21 | (TMD-NBD)2 | Full | GSVIVT01027990001 | CBI36841.3 | 7 | + | 3032722 to 3040092 | 7371 | 14 | 13 | 3240 | 1079 | 6,91 | 121278.34 |
| VvMRP22 | VvABCC22 | (TMD-NBD)2 | Full | GSVIVT01028440001 | CBI37201.3 | 7 | - | 7535201 to 7546064 | 10864 | 14 | 13 | 4083 | 1360 | 8,79 | 151339.00 |
| VvMRP23 | VvABCC23 | (TMD-NBD)2 | Partial | GSVIVT01028459001 | CBI37218.3 | 7 | + | 7912161 to 7916627 | 4467 | 6 | 5 | 846 | 287 | 5,39 | 31576.17 |
| VvMRP24 | VvABCC24 | (TMD-NBD)2 | Full | GSVIVT01028722001 | CBI22551.3 | 16 | + | 19447213 to 19454986 | 7774 | 13 | 12 | 4188 | 1395 | 6,07 | 155291.53 |
| VvMRP25 | VvABCC25 | (TMD-NBD)2 | Full | GSVIVT01028744001 | CBI22568.3 | 16 | - | 19218947 to 19224345 | 5399 | 15 | 14 | 3483 | 1160 | 6,81 | 130542.47 |
| VvMRP26 | VvABCC26 | (TMD-NBD)2 | Full | GSVIVT01037789001 | CBI26749.3 | 19 | - | 7910839 to 7917664 | 6826 | 14 | 13 | 3810 | 1269 | 6,65 | 140766.08 |
| **PMP** | **ABCD** |  |  |  |  |  |  |  |  |  |  |  |  |  |  |
| VvPMP1 | VvABCD1 | TMD-NBD | Full | GSVIVT01036685001 | CBI24120.3 | 19 | + | 23874564 to 23889349 | 14786 | 11 | 10 | 2094 | 697 | 5,19 | 78947,57 |
| **RLI** | **ABCE** |  |  |  |  |  |  |  |  |  |  |  |  |  |  |
| VvRLI1 | VvABCE1 | NBD-NBD | Full | GSVIVT01036876001 | CBI29193.3 | 2 | - | 18345602 to 18354600 | 8999 | 11 | 10 | 1878 | 625 | 8,21 | 70884.95 |
| **GCNs** | **ABCFs** |  |  |  |  |  |  |  |  |  |  |  |  |  |  |
| VvGCN1 | VvABCF1 | NBD-NBD | Full | GSVIVT01001694001 | CBI35509.3 | 18 | + | 14382175 to 14384416 | 2242 | 7 | 6 | 1221 | 406 | 5,36 | 46636,27 |
| VvGCN2 | VvABCF2 | NBD-NBD | Full | GSVIVT01019609001 | CBI34548.3 | 2 | - | 1919317 to 1929177 | 9861 | 18 | 17 | 2151 | 716 | 5,74 | 79923.08 |
| VvGCN3 | VvABCF3 | NBD-NBD | Full | GSVIVT01022235001 | CBI21514.3 | 7 | + | 17739420 to 17745441 | 6022 | 11 | 10 | 1968 | 655 | 7,97 | 73218.73 |
| VvGCN4 | VvABCF4 | NBD-NBD | Full | GSVIVT01031505001 | CBI17163.3 | 6 | - | 17837690 to 17845041 | 7352 | 8 | 7 | 1347 | 448 | 5,40 | 50625.96 |
| VvGCN5 | VvABCF5 | NBD-NBD | Full | GSVIVT01034906001 | CBI18557.3 | 18 | + | 16302857 to 16307318 | 4462 | 9 | 8 | 1860 | 619 | 6,17 | 69194.41 |
| **WBCs** | **ABCGs** |  |  |  |  |  |  |  |  |  |  |  |  |  |  |
| VvWBC1 | VvABCG1 | NBD-TMD | Full | GSVIVT01001660001 | CBI35490.3 | 18 | - | 14023104 to 14027197 | 4094 | 10 | 9 | 2331 | 776 | 9,33 | 86733,02 |
| VvWBC2 | VvABCG2 | NBD-TMD | Full | GSVIVT01002136001 | CBI25872.3 | Un | - | 33154621 to 33158014 | 3394 | 8 | 7 | 1779 | 592 | 8,68 | 66033.78 |
| VvWBC3 | VvABCG3 | NBD-TMD | Full | GSVIVT01002139001 | CBI25874.3 | Un | - | 33168714 to 33175056 | 6343 | 11 | 10 | 2004 | 667 | 8,34 | 74118.03 |
| VvWBC4 | VvABCG4 | NBD-TMD | Full | GSVIVT01002949001 | CBI35723.3 | Un | + | 37331797 to 37336392 | 4596 | 8 | 7 | 2676 | 891 | 8,57 | 99045.65 |
| VvWBC5 | VvABCG5 | NBD-TMD | Full | GSVIVT01003413001 | CBI33134.3 | 7 | - | 14762359 to 14765617 | 3259 | 8 | 7 | 1890 | 629 | 8,54 | 69971.33 |
| VvWBC6 | VvABCG6 | NBD-TMD | Full | GSVIVT01008456001 | CBI15591.3 | 17 | + | 1830227 to 1832876 | 2650 | 5 | 4 | 2118 | 705 | 9,27 | 79538.11 |
| VvWBC7 | VvABCG7 | NBD-TMD | Full | GSVIVT01011781001 | CBI26926.3 | 1 | - | 4194983 to 4204540 | 9558 | 12 | 11 | 2190 | 729 | 8,46 | 80103.18 |
| VvWBC8 | VvABCG8 | NBD-TMD | Full | GSVIVT01011981001 | CBI27088.3 | 1 | - | 2429294 to 2433043 | 3750 | 5 | 4 | 2031 | 676 | 8,58 | 75231.84 |
| VvWBC9 | VvABCG9 | NBD-TMD | Full | GSVIVT01014222001 | CBI20207.3 | 19 | - | 1636678 to 1638665 | 1988 | 5 | 4 | 1614 | 537 | 8,86 | 60408.03 |
| VvWBC10 | VvABCG10 | NBD-TMD | Full | GSVIVT01014402001 | CBI20349.3 | 19 | + | 3404282 to 3407783 | 3502 | 5 | 4 | 1725 | 574 | 8,98 | 63989.23 |
| VvWBC11 | VvABCG11 | NBD-TMD | Full | GSVIVT01014733001 | CBI39781.3 | 19 | + | 9060515 to 9063661 | 3147 | 9 | 8 | 2061 | 686 | 8,99 | 77933.84 |
| VvWBC12 | VvABCG12 | NBD-TMD | Full | GSVIVT01015767001 | CBI34070.3 | 3 | + | 17161781 to 17168980 | 7200 | 9 | 8 | 1827 | 608 | 8,24 | 66945.64 |
| VvWBC13 | VvABCG13 | NBD-TMD | Full | GSVIVT01015768001 | CBI34071.3 | 3 | - | 17178217 to 17218628 | 40412 | 21 | 20 | 3930 | 1309 | 8,42 | 144429.53 |
| VvWBC14 | VvABCG14 | NBD-TMD | Full | GSVIVT01015771001 | CBI34073.3 | 3 | - | 17271807 to 17276119 | 4313 | 8 | 7 | 1938 | 645 | 8,69 | 72367.38 |
| VvWBC15 | VvABCG15 | NBD-TMD | Full | GSVIVT01016240001 | CBI31434.3 | 13 | - | 6030373 to 6044405 | 14033 | 16 | 15 | 3216 | 1071 | 8,97 | 119215.03 |
| VvWBC16 | VvABCG16 | NBD-TMD | Full | GSVIVT01020687001 | CBI21966.3 | 12 | - | 3103958 to 3105784 | 1827 | 3 | 2 | 1656 | 551 | 9,26 | 62432.76 |
| VvWBC17 | VvABCG17 | NBD-TMD | Full | GSVIVT01022346001 | CBI21607.3 | 7 | - | 18781499 to 18783615 | 2117 | 4 | 3 | 1563 | 520 | 8,99 | 58219.67 |
| VvWBC18 | VvABCG18 | NBD-TMD | Full | GSVIVT01022526001 | CBI39105.3 | 8 | + | 4360478 to 4369702 | 9225 | 10 | 9 | 2217 | 738 | 9,17 | 81766.39 |
| VvWBC19 | VvABCG19 | NBD-TMD | Full | GSVIVT01024228001 | CBI26391.3 | 16 | - | 7726 to 12080 | 4355 | 10 | 9 | 2118 | 705 | 9,02 | 78764.15 |
| VvWBC20 | VvABCG20 | NBD-TMD | Full | GSVIVT01025230001 | CBI16347.3 | 6 | + | 2981734 to 2995237 | 13504 | 15 | 14 | 3123 | 1040 | 9,11 | 115527.07 |
| VvWBC21 | VvABCG21 | NBD-TMD | Full | GSVIVT01025582001 | CBI32651.3 | 8 | + | 13902809 to 13905474 | 2666 | 7 | 6 | 1887 | 628 | 9,13 | 69796.31 |
| VvWBC22 | VvABCG22 | NBD-TMD | Full | GSVIVT01025712001 | CBI32756.3 | 8 | + | 12741119 to 12758023 | 16905 | 19 | 18 | 3303 | 1100 | 9,01 | 122088.06 |
| VvWBC23 | VvABCG23 | NBD-TMD | Full | GSVIVT01028809001 | CBI22616.3 | 16 | - | 18530210 to 18534998 | 4789 | 3 | 2 | 1752 | 583 | 9,64 | 65178.21 |
| VvWBC24 | VvABCG24 | NBD-TMD | Full | GSVIVT01031516001 | CBI17169.3 | 6 | + | 17745838 to 17756072 | 10235 | 11 | 10 | 2169 | 722 | 8,99 | 80158.03 |
| VvWBC25 | VvABCG25 | NBD-TMD | Full | GSVIVT01031528001 | CBI17178.3 | 6 | + | 17500288 to 17564204 | 63917 | 32 | 31 | 6435 | 2144 | 9,10 | 238800.34 |
| VvWBC26 | VvABCG26 | NBD-TMD | Full | GSVIVT01031529001 | CBI17179.3 | 6 | + | 17493089 to 17498594 | 5506 | 9 | 8 | 1869 | 622 | 9,13 | 69881.00 |
| VvWBC27 | VvABCG27 | NBD-TMD | Full | GSVIVT01032625001 | CBI25312.3 | 13 | - | 2094819 to 2097062 | 2244 | 4 | 3 | 1992 | 663 | 8,59 | 73855.30 |
| VvWBC28 | VvABCG28 | NBD-TMD | Full | GSVIVT01034463001 | CBI18157.3 | 18 | - | 20935211 to 20942470 | 7260 | 4 | 3 | 1938 | 645 | 9,31 | 70787.98 |
| VvWBC29 | VvABCG29 | NBD-TMD | Full | GSVIVT01036869001 | CBI29188.3 | 19 | - | 7910839 to 7917664 | 6826 | 14 | 13 | 3810 | 657 | 9,03 | 73658.58 |
| VvWBC30 | VvABCG30 | NBD-TMD | Full | GSVIVT01037274001 | CBI24242.3 | 6 | - | 16610144 to 16612096 | 1953 | 3 | 2 | 1815 | 604 | 8,50 | 68650.94 |
| **PDRs** |  |  |  |  |  |  |  |  |  |  |  |  |  |  |  |
| VvPDR1 | VvABCG31 | (NBD-TMD)2 | Full | GSVIVT01015456001 | CBI28131.3 | 11 | + | 3825506 to 3837096 | 11591 | 24 | 23 | 4266 | 1421 | 7,27 | 162518.23 |
| VvPDR2 | VvABCG32 | (NBD-TMD)2 | Full | GSVIVT01015461001 | CBI28135.3 | 11 | - | 3887993 to 3897672 | 9680 | 21 | 20 | 5925 | 1974 | 6,41 | 222086.15 |
| VvPDR3 | VvABCG33 | (NBD-TMD)2 | Full | GSVIVT01016991001 | CBI36062.3 | 9 | + | 3229012 to 3244545 | 15534 | 19 | 18 | 2853 | 950 | 6,53 | 106600.06 |
| VvPDR4 | VvABCG34 | (NBD-TMD)2 | Partial | GSVIVT01016992001 | CBI36063.3 | 9 | + | 3244545 to 3246079 | 1535 | 5 | 4 | 969 | 322 | 9,14 | 37637.85 |
| VvPDR5 | VvABCG35 | (NBD-TMD)2 | Partial | GSVIVT01016993001 | CBI36064.3 | 9 | + | 3246544 to 3252734 | 6191 | 13 | 12 | 2073 | 690 | 7,23 | 77924.79 |
| VvPDR6 | VvABCG36 | (NBD-TMD)2 | Full | GSVIVT01016998001 | CBI36069.3 | 9 | - | 3318607 to 3327354 | 8748 | 25 | 24 | 4368 | 1455 | 6,82 | 164752.46 |
| VvPDR7 | VvABCG37 | (NBD-TMD)2 | Full | GSVIVT01016999001 | CBI36070.3 | 9 | - | 3328286 to 3336959 | 8674 | 21 | 20 | 4482 | 1493 | 8,42 | 169593.96 |
| VvPDR8 | VvABCG38 | (NBD-TMD)2 | Full | GSVIVT01017184001 | CBI36209.3 | 9 | - | 5099104 to 5114849 | 15746 | 29 | 28 | 4749 | 1582 | 8,51 | 179546.33 |
| VvPDR9 | VvABCG39 | (NBD-TMD)2 | Full | GSVIVT01017185001 | CBI36210.3 | 9 | - | 5099104 to 5114849 | 19436 | 31 | 30 | 4929 | 1642 | 6,85 | 185433.04 |
| VvPDR10 | VvABCG40 | (NBD-TMD)2 | Full | GSVIVT01017187001 | CBI36212.3 | 9 | - | 5146238 to 5168948 | 22711 | 40 | 39 | 5313 | 1770 | 8,51 | 201428.96 |
| VvPDR11 | VvABCG41 | (NBD-TMD)2 | Full | GSVIVT01017188001 | CBI36213.3 | 9 | - | 5146238 to 5168948 | 13785 | 27 | 26 | 4941 | 1646 | 8,71 | 185507.45 |
| VvPDR12 | VvABCG42 | (NBD-TMD)2 | Full | GSVIVT01017196001 | CBI36221.3 | 9 | - | 5216635 to 5238081 | 21447 | 28 | 27 | 4089 | 1362 | 8,50 | 153786.85 |
| VvPDR13 | VvABCG43 | (NBD-TMD)2 | Full | GSVIVT01017198001 | CBI36223.3 | 9 | - | 5257252 to 5268154 | 10903 | 32 | 31 | 5124 | 1707 | 8,76 | 191868.26 |
| VvPDR14 | VvABCG44 | (NBD-TMD)2 | Full | GSVIVT01017201001 | CBI36226.3 | 9 | - | 5281157 to 5294157 | 13001 | 29 | 28 | 4614 | 1537 | 6,10 | 174432.29 |
| VvPDR15 | VvABCG45 | (NBD-TMD)2 | Full | GSVIVT01017202001 | CBI36227.3 | 9 | - | 5281157 to 5294157 | 7242 | 24 | 23 | 4356 | 1451 | 8,45 | 163746.71 |
| VvPDR16 | VvABCG46 | (NBD-TMD)2 | Full | GSVIVT01017204001 | CBI36229.3 | 9 | - | 5316069 to 5343724 | 27656 | 60 | 59 | 9429 | 3142 | 8,86 | 356575.17 |
| VvPDR17 | VvABCG47 | (NBD-TMD)2 | Full | GSVIVT01017676001 | CBI26001.3 | 5 | + | 2543551 to 2558096 | 14546 | 25 | 24 | 4461 | 1486 | 8,51 | 169112.40 |
| VvPDR18 | VvABCG48 | (NBD-TMD)2 | Full | GSVIVT01024743001 | CBI15958.3 | 6 | + | 7284289 to 7297469 | 13181 | 27 | 26 | 4452 | 1483 | 8,83 | 166897.81 |
| VvPDR19 | VvABCG49 | (NBD-TMD)2 | Full | GSVIVT01031314001 | CBI39657.3 | 14 | + | 439701 to 448856 | 9156 | 24 | 23 | 4221 | 1406 | 8,72 | 159145.63 |
| VvPDR20 | VvABCG50 | (NBD-TMD)2 | Full | GSVIVT01031377001 | CBI17066.3 | 6 | - | 19360714 to 19368397 | 7684 | 23 | 22 | 4167 | 1388 | 5,92 | 156314.38 |
| VvPDR21 | VvABCG51 | (NBD-TMD)2 | Full | GSVIVT01031378001 | CBI17067.3 | 6 | - | 19347375 to 19360240 | 12866 | 21 | 20 | 4383 | 1460 | 6,12 | 165247.74 |
| VvPDR22 | VvABCG52 | (NBD-TMD)2 | Full | GSVIVT01031380001 | CBI17068.3 | 6 | - | 19331347 to 19339760 | 8414 | 24 | 23 | 3345 | 1114 | 6,88 | 125797.94 |
| VvPDR23 | VvABCG53 | (NBD-TMD)2 | Full | GSVIVT01033804001 | CBI30263.3 | 8 | + | 17659640 to 17669419 | 9780 | 20 | 19 | 4074 | 1357 | 6,49 | 153397.09 |
| VvPDR24 | VvABCG54 | (NBD-TMD)2 | Full | GSVIVT01034741001 | CBI40238.3 | 13 | - | 8817915 to 8828337 | 10423 | 23 | 22 | 4353 | 1450 | 7,98 | 164563.95 |
| VvPDR25 | VvABCG55 | (NBD-TMD)2 | Full | GSVIVT01034745001 | CBI40240.3 | 13 | - | 8859680 to 8875297 | 15618 | 26 | 25 | 6591 | 2196 | 8,81 | 250654.84 |
| VvPDR26 | VvABCG56 | (NBD-TMD)2 | Full | GSVIVT01034746001 | CBI40241.3 | 13 | - | 8876000 to 8895143 | 19144 | 25 | 24 | 4335 | 1444 | 8,29 | 165003.58 |
| VvPDR27 | VvABCG57 | (NBD-TMD)2 | Full | GSVIVT01034748001 | CBI40242.3 | 13 | - | 8897703 to 8904965 | 7263 | 21 | 20 | 4167 | 1388 | 6,38 | 157201.53 |
| VvPDR28 | VvABCG58 | (NBD-TMD)2 | Full | GSVIVT01035715001 | CBI20926.3 | 4 | - | 3596651 to 3605452 | 8802 | 25 | 24 | 4281 | 1426 | 8,55 | 161911.86 |
| VvPDR29 | VvABCG59 | (NBD-TMD)2 | Full | GSVIVT01035780001 | CBI20978.3 | 4 | - | 4227019 to 4234518 | 7500 | 24 | 23 | 4311 | 1436 | 8,49 | 162392.49 |
| VvPDR30 | VvABCG60 | (NBD-TMD)2 | Full | GSVIVT01035784001 | CBI20980.3 | 4 | + | 4258543 to 4265236 | 6694 | 25 | 24 | 4140 | 1379 | 7,08 | 156002.58 |
| VvPDR31 | VvABCG61 | (NBD-TMD)2 | Full | GSVIVT01035785001 | CBI20981.3 | 4 | + | 4265752 to 4286094 | 20343 | 16 | 15 | 2415 | 804 | 8,94 | 90981.55 |
| VvPDR32 | VvABCG62 | (NBD-TMD)2 | Partial | GSVIVT01035786001 | CBI20982.3 | 4 | + | 4286128 to 4295628 | 9501 | 12 | 11 | 1815 | 604 | 8,13 | 68711.39 |
| VvPDR33 | VvABCG63 | (NBD-TMD)2 | Full | GSVIVT01036184001 | CBI28361.3 | 6 | + | 19713908 to 19723295 | 9388 | 23 | 22 | 4638 | 1545 | 6,40 | 175053.95 |
| **NAPs** | **ABCIs** |  |  |  |  |  |  |  |  |  |  |  |  |  |  |
| VvNAP1 | VvABCI1 | NBD | Full | GSVIVT01010853001 | CBI36729.3 | 5 | - | 23355077 to 23362508 | 7432 | 5 | 4 | 1149 | 382 | 8,98 | 41902,86 |
| VvNAP2 | VvABCI2 | NBD | Full | GSVIVT01012742001 | CBI23255.3 | 10 | - | 1165899 to 1176476 | 10578 | 4 | 3 | 1536 | 511 | 6,96 | 57563,90 |
| VvNAP3 | VvABCI3 | NBD | Full | GSVIVT01013180001 | CBI33903.3 | 2 | + | 7019468 to 7043516 | 24049 | 3 | 2 | 1443 | 480 | 6,54 | 52326,38 |
| VvNAP4 | VvABCI4 | NBD | Full | GSVIVT01017866001 | CBI26163.3 | 5 | - | 4127406 to 4132500 | 5095 | 7 | 6 | 1200 | 399 | 10,02 | 44325,49 |
| VvNAP5 | VvABCI5 | NBD | Full | GSVIVT01019131001 | CBI17656.3 | 4 | + | 16404476 to 16417806 | 13331 | 11 | 10 | 1041 | 337 | 6,38 | 37116,37 |
| VvNAP6 | VvABCI6 | NBD | Full | GSVIVT01031286001 | CBI39636.3 | 14 | - | 613453 to 616484 | 3032 | 7 | 6 | 990 | 329 | 9,00 | 36883.56 |
| **SMCs** |  |  |  |  |  |  |  |  |  |  |  |  |  |  |  |
| VvSMC1 | - | NBD | Full | GSVIVT01011408001 | CBI22212.3 | 14 | - | 29409938 to 29423532 | 13595 | 28 | 27 | 3762 | 1253 | 6,54 | 142999.44 |
| VvSMC2 | - | NBD | Full | GSVIVT01018715001 | CBI24628.3 | 16 | - | 10691873 to 10719210 | 27338 | 23 | 22 | 3516 | 1171 | 7,5 | 132531.45 |
| VvSMC3 | - | NBD | Full | GSVIVT01028343001 | CBI37123.3 | 7 | - | 6291110 to 6348702 | 57593 | 23 | 22 | 6888 | 2295 | n/a | n/a |

* *Vitis vinifera* ABC proteins have been named according to HGNC (Human Gene Nomenclature Committee) (Verrier et al., 2008).
