# Supplementary material for: Whole-Genome Survey of the Putative ATP-Binding Cassette Transporter Family Genes in Vitis vinifera
Source: PLoS One. 2013 Nov 11;8(11):e78860. doi: 10.1371/journal.pone.0078860 (PMC3823996; doi:10.1371/journal.pone.0078860)
Supplement: Table S2 — Detailed inventory of Vitis ABC proteins and their genes. Columns 1–16 contain the protein acronym (Name), topology (number and orientation of nucleotide-binding folds [NBFs] and transmembrane domains [TMDs]), coding sequence (CDS), Vitis proteome 12× ID, GenBank ID, chromosome location (Chr), gene length, number of introns and exons, open reading frame (ORF) length, protein length, estimates of molecular weight, and pI of the protein for each gene are given. (DOC) [file pone.0078860.s002.doc]

**Table S2.** **Summary of the *Vitis* ABC proteins.** The identified open reading frames (ORFs) are classified into 13 subfamilies, whose nomenclature is represented according to both Sanchez-Fernandez et al. (2001) and Verrier et al. (2008). The chromosomal (C) locations of the ORFs, the total number of ORFs for each category on each chromosome and in the whole genome, and the total numbers of full-size molecule and half-size molecule transporters and proteins lacking contiguous transmembrane domains (TMDs) (“soluble” proteins) are shown.

|  | **C1** | **C2** | **C3** | **C4** | **C5** | **C6** | **C7** | **C8** | **C9** | **C10** | **C11** | **C12** | **C13** | **C14** | **C15** | **C16** | **C17** | **C18** | **C19** | **CUn** | **Total** |
| --- | --- | --- | --- | --- | --- | --- | --- | --- | --- | --- | --- | --- | --- | --- | --- | --- | --- | --- | --- | --- | --- |
| **Whole Molecule Transporters** |  |  |  |  |  |  |  |  |  |  |  |  |  |  |  |  |  |  |  |  |  |
| VvMDRs (ABCB) |  | 1 | 1 | 1 | 2 | 1 | 1 | 1 | 1 | 2 | 1 |  |  | 2 |  | 1 | 1 | 1 | 2 |  | **19** |
| VvMRPs (ABCC) |  | 7 |  |  |  |  | 3 |  | 3 | 4 |  |  |  |  | 1 | 2 |  |  | 6 |  | **26** |
| VVPDRs (ABCG) |  |  |  | 5 | 1 | 5 |  | 1 | 14 |  | 2 |  | 4 | 1 |  |  |  |  |  |  | **33** |
| VvAOH (ABCA) |  |  |  |  |  |  |  | 1 |  |  |  |  |  |  |  |  |  |  |  |  | **1** |
|  |  |  |  |  |  |  |  |  |  |  |  |  |  |  |  |  |  |  |  |  |  |
| **Half Molecule Transporters** |  |  |  |  |  |  |  |  |  |  |  |  |  |  |  |  |  |  |  |  |  |
| VvWBCs (ABCG) | 2 |  | 3 |  |  | 5 | 2 | 3 |  |  |  | 1 | 2 |  |  | 2 | 1 | 2 | 4 | 3 | **30** |
| VvATHs (ABCA) |  |  |  |  |  |  |  | 1 |  |  |  |  |  |  |  |  | 2 | 1 |  |  | **4** |
| VvATM (ABCB) |  |  |  |  |  | 1 |  |  |  |  |  |  |  |  |  |  |  |  |  |  | **1** |
| VvTAPs (ABCB) |  |  |  |  |  |  |  |  |  |  |  |  |  | 2 |  | 1 | 1 |  |  | 1 | **5** |
| VvPMP (ABCD) |  |  |  |  |  |  |  |  |  |  |  |  |  |  |  |  |  |  | 1 |  | **1** |
|  |  |  |  |  |  |  |  |  |  |  |  |  |  |  |  |  |  |  |  |  |  |
| **Soluble Transporters** |  |  |  |  |  |  |  |  |  |  |  |  |  |  |  |  |  |  |  |  |  |
| VvRLI (ABCE) |  | 1 |  |  |  |  |  |  |  |  |  |  |  |  |  |  |  |  |  |  | **1** |
| VvSMCs |  |  |  |  |  |  | 1 |  |  |  |  |  |  | 1 |  | 1 |  |  |  |  | **3** |
| VvGCNs (ABCF) |  | 1 |  |  |  | 1 | 1 |  |  |  |  |  |  |  |  |  |  | 2 |  |  | **5** |
| VvNAPs (ABCI) |  | 1 |  | 1 | 2 |  |  |  |  | 1 |  |  |  | 1 |  |  |  |  |  |  | **6** |
|  |  |  |  |  |  |  |  |  |  |  |  |  |  |  |  |  |  |  |  |  |  |
|  |  |  |  |  |  |  |  |  |  |  |  |  |  |  |  |  |  |  |  |  |  |
| **Total** | **2** | **11** | **4** | **7** | **5** | **13** | **8** | **7** | **18** | **7** | **3** | **1** | **6** | **7** | **1** | **7** | **5** | **6** | **13** | **4** | **135** |
