# Supplementary material for: Whole-Genome Survey of the Putative ATP-Binding Cassette Transporter Family Genes in Vitis vinifera
Source: PLoS One. 2013 Nov 11;8(11):e78860. doi: 10.1371/journal.pone.0078860 (PMC3823996; doi:10.1371/journal.pone.0078860)
Supplement: Table S3 — Orthologs of Vitis ABC proteins identified in diverse plant species. Columns 1–6 contain the protein name represented according to both Sanchez-Fernandez et al. (2001) and Verrier et al. (2008), Vitis proteome 12× ID, GenBank ID, species, percentage identity (%ID), UniprotKB ID. (DOC) [file pone.0078860.s003.doc]

**Table S3.** **Orthologs of *Vitis* ABC proteins identified in diverse plant species.**

Columns 1–6 contain the protein name represented according to both Sanchez-Fernandez et al. (2001) and Verrier et al. (2008), *Vitis* proteome 12× ID, GenBank ID, species, percentage identity (%ID), UniprotKB ID.

| **Sanchez-Fernandez**  **subfamily name** | **HGNC subfamily name** | **12X Vitis vinifera ID** | **Species** | **%ID** | **UniprotKB ID** |
| --- | --- | --- | --- | --- | --- |
| *VvAOH1* | *VvABCA1* | GSVIVT01033258001 | *Populus trichocarpa* | 91.3 | B9H9T2 |
|  |  |  | *Arabidopsis thaliana* | 84.0 | Q84M24 |
|  |  |  | *Ricinus communis* | 89.1 | B9SU42 |
| *VvATH1* | *VvABCA2* | GSVIVT01008040001 | *Populus trichocarpa* | 90.7 | B9N4B2 |
|  |  |  | *Ricinus communis* | 90.2 | B9R969 |
|  |  |  | *Oryza sativa ssp indica* | 87.4 | B8BAI4 |
|  |  |  | *Arabidopsis thaliana* | 88.5 | Q9STT5 |
| *VvATH2* | *VvABCA3* | GSVIVT01008042001 | *Populus trichocarpa* | 84.7 | B9I300 |
|  |  |  | *Oryza sativa ssp japonica* | 83.6 | Q6ZIW1 |
|  |  |  | *Arabidopsis thaliana* | 84.7 | Q84K47 |
| *VvATH4* | *VvABCA5* | GSVIVT01033860001 | *Populus trichocarpa* | 86.1 | B9HC81 |
| *VvMDR5* | *VvABCB5* | GSVIVT01013125001 | *Gossypium hirsutum* | 87.5 | Q9SDM5 |
|  |  |  | *Arabidopsis thaliana* | 86.8 | Q8LPK2 |
|  |  |  | *Ricinus communis* | 85.0 | B9SNT8 |
|  |  |  | *Populus trichocarpa* | 83.0 | B9MU48 |
|  |  |  | *Oryza sativa ssp japonica* | 84.0 | Q8GU75 |
|  |  |  | *Zea mays* | 83.3 | C0PDJ8 |
| *VvMDR8* | *VvABCB8* | GSVIVT01016617001 | *Ricinus communis* | 84.8 | B9T158 |
|  |  |  | *Populus trichocarpa* | 83.3 | B9HTN2 |
| *VvMDR10* | *VvABCB10* | GSVIVT01017696001 | *Ricinus communis* | 63.0 | B9S018 |
| *VvMDR11* | *VvABCB11* | GSVIVT01021365001 | *Arabidopsis thaliana* | 85.5 | Q9FHF1 |
|  |  |  | *Catharantus roseus* | 79.9 | A5X7X7 |
|  |  |  | *Populus trichocarpa* | 71.4 | B9N9D8 |
|  |  |  | *Ricinus communis* | 77.9 | B9S188 |
| *VvMDR12* | *VvABCB12* | GSVIVT01021366001 | *Ricinus communis* | 85.2 | B9S190 |
|  |  |  | *Populus trichocarpa* | 87.0 | B9N9D8 |
|  |  |  | *Catharantus roseus* | 82.3 | A5X7X7 |
|  |  |  | *Oryza sativa ssp japonica* | 83.2 | A2ZS26 |
|  |  |  | *Arabidopsis thaliana* | 84.4 | Q9M0M2 |
| *VvMDR13* | *VvABCB13* | GSVIVT01025040001 | *Arabidopsis thaliana* | 81.4 | Q9M3B9 |
|  |  |  | *Oryza sativa ssp japonica* | 76.7 | B9EX09 |
|  |  |  | *Populus trichocarpa* | 84.7 | B9HM46 |
|  |  |  | *Ricinus communis* | 87.3 | B9T158 |
| *VvMDr14* | *VvABCB14* | GSVIVT01028256001 | *Arabidopsis thaliana* | 87.0 | Q9SYI3 |
|  |  |  | *Coptis japonica* | 74.5 | Q94IH6 |
|  |  |  | *Populus trichocarpa* | 81.1 | B9GT94 |
|  |  |  | *Ricinus communis* | 77.6 | B9RN47 |
| *VvMDR15* | *VvABCB15* | GSVIVT01032578001 | *Arabidopsis thaliana* | 82.2 | Q9LSJ2 |
|  |  |  | *Oryza sativa ssp indica* | 67.0 | B8AJ92 |
|  |  |  | *Populus trichocarpa* | 76.3 | B9IJZ6 |
|  |  |  | *Ricinus communis* | 83.7 | B9RUH8 |
| *VvMDR17* | *VvABCB17* | GSVIVT01033645001 | *Ricinus communis* | 90.4 | B9S0G9 |
|  |  |  | *Populus trichocarpa* | 90.0 | B9IFR5 |
|  |  |  | *Solanum tuberosum* | 86.8 | Q9ZRG2 |
|  |  |  | *Oryza sativa ssp japonica* | 84.2 | Q7EZL1 |
|  |  |  | *Arabidopsis thaliana* | 88.9 | Q0WL75 |
|  |  |  | *Oryza sativa ssp japonica* | 83.6 | Q7EZL2 |
|  |  |  | *Sorghum bicolor* | 81.7 | Q6UC91 |
|  |  |  | *Zea mays* | 81.0 | Q6UNK5 |
| *VvATM1* | *VvABCB20* | GSVIVT01024527001 | *Ricinus communis* | 90.0 | B9T3V9 |
|  |  |  | *Populus trichocarpa* | 88.3 | B9I784 |
|  |  |  | *Arabidopsis thaliana* | 84.2 | Q9LVM1 |
| *VvTAP1* | *VvABCB21* | GSVIVT01005757001 | *Populus trichocarpa* | 83.9 | B9HTT8 |
|  |  |  | *Arabidopsis thaliana* | 81.6 | Q8RY46 |
|  |  |  | *Ricinus communis* | 81.9 | B9SPK8 |
| *VvTAP2* | *VvABCB22* | GSVIVT01008121001 | *Populus trichocarpa* | 82.8 | B9N4E9 |
| *VvTAP3* | *VvABCB23* | GSVIVT01010634001 | *Ricinus communis* | 82.1 | B9R9H4 |
| *VvMRP1* | *VvABCC1* | GSVIVT01010326001 | *Ricinus communis* | 81.8 | B9RMN4 |
| *VvMRP7* | *VvABCC7* | GSVIVT01015960001 | *Populus trichocarpa* | 82.5 | B9GVL2 |
|  |  |  | *Arabidopsis thaliana* | 81.9 | Q9SKX0 |
| *VvMRP8* | *VvABCC8* | GSVIVT01016879001 | *Populus trichocarpa* | 94.0 | B9MWQ7 |
|  |  |  | *Arabidopsis thaliana* | 87.0 | Q8LGU1 |
| *VvMRP17* | *VvABCC17* | GSVIVT01021589001 | *Ricinus communis* | 87.0 | B9SKU0 |
|  |  |  | *Arabidopsis thaliana* | 85.4 | Q42093 |
|  |  |  | *Populus trichocarpa* | 87.8 | B9I191 |
|  |  |  | *Oryza sativa* | 85.7 | Q01JU7 |
|  |  |  | *Arabidopsis thaliana* | 80.0 | Q9C8G9 |
| *VvMRP22* | *VvABCC22* | GSVIVT01028440001 | *Ricinus communis* | 94.5 | B9SKL4 |
|  |  |  | *Populus trichocarpa* | 93.2 | B9IBC8 |
|  |  |  | *Arabidopsis thaliana* | 87.4 | Q7GB25 |
|  |  |  | *Populus trichocarpa* | 87.7 | B9GRC2 |
|  |  |  | *Oryza sativa ssp japonica* | 83.3 | B9FB03 |
|  |  |  | *Zea mays* | 83.3 | A7KVC2 |
| *VvMRP24* | *VvABCC24* | GSVIVT01028722001 | *Arabidopsis thaliana* | 71.1 | Q9LYS2 |
|  |  |  | *Oryza sativa ssp japonica* | 72.1 | Q5VMX7 |
|  |  |  | *Populus trichocarpa* | 82.6 | B9N9A1 |
|  |  |  | *Ricinus communis* | 84.3 | B9RIN7 |
|  |  |  | *Zea mays* | 72.1 | Q6J0P5 |
| *VvMRP26* | *VvABCC26* | GSVIVT01037789001 | *Arabidopsis thaliana* | 85.9 | Q8VZZ4 |
|  |  |  | *Gossypium hirsutum* | 76.5 | B0KYV2 |
|  |  |  | *Oryza sativa ssp japonica* | 74.4 | Q0JQA5 |
|  |  |  | *Populus trichocarpa* | 80.2 | B9GJX7 |
|  |  |  | *Ricinus communis* | 71.0 | B9T464 |
|  |  |  | *Triticum aestivum* | 62.0 | Q71CZ3 |
| *VvPMP1* | *VvABCD1* | GSVIVT01036685001 | *Populus trichocarpa* | 93.8 | B9HYE2 |
|  |  |  | *Ricinus communis* | 92.1 | B9RKV1 |
|  |  |  | *Oryza sativa ssp japonica* | 87.2 | Q5QNE1 |
|  |  |  | *Arabidopsis thaliana* | 86.9 | Q6NLC1 |
| *VvGCN2* | *VvABCF2* | GSVIVT01019609001 | *Populus trichocarpa* | 84.2 | B9GL16 |
| *VvGCN3* | *VvABCF3* | GSVIVT01022235001 | *Ricinus communis* | 92.8 | B9STB1 |
|  |  |  | *Populus trichocarpa* | 91.0 | B9HG24 |
|  |  |  | *Arabidopsis thaliana* | 89.2 | Q9FIB4 |
|  |  |  | *Zea mays* | 86.7 | Q6GYA9 |
|  |  |  | *Oryza sativa ssp japonica* | 86.7 | Q2R1J1 |
| *VvGCN4* | *VvABCF4* | GSVIVT01031505001 | *Populus trichocarpa* | 94.4 | B9N856 |
|  |  |  | *Ricinus communis* | 92.6 | B9SCH6 |
|  |  |  | *Zea mays* | 92.4 | B4FZI2 |
|  |  |  | *Oryza sativa* | 92.3 | B8B9L7 |
|  |  |  | *Physcomitrella patens ssp patens* | 86.0 | A9TM80 |
|  |  |  | *Capsicum chinense* | 88.8 | Q5GMM7 |
| *VvWBC5* | *VvABCG5* | GSVIVT01003413001 | *Populus trichocarpa* | 93.0 | B9H4R6 |
| *VvWBC6* | *VvABCG6* | GSVIVT01008456001 | *Ricinus communis* | 85.6 | B9SXR8 |
|  |  |  | *Populus trichocarpa* | 85.2 | B9I2C8 |
| *VvWBC7* | *VvABCG7* | GSVIVT01011781001 | *Populus trichocarpa* | 92.9 | B9HW88 |
|  |  |  | *Ricinus communis* | 92.5 | B9SKT4 |
|  |  |  | *Arabidopsis thaliana* | 91.0 | Q9ZU35 |
|  |  |  | *Oryza sativa ssp indica* | 82.8 | B8ACZ8 |
|  |  |  | *Zea mays* | 88.2 | B6U7G4 |
| *VvWBC8* | *VvABCG8* | GSVIVT01011981001 | *Arabidopsis thaliana* | 84.1 | Q7XA72 |
|  |  |  | *Populus trichocarpa* | 94.2 | B9HWN9 |
|  |  |  | *Ricinus communis* | 93.2 | B9SEG2 |
| *VvWBC9* | *VvABCG9* | GSVIVT01014222001 | *Arabidopsis thaliana* | 78.4 | Q9MAH4 |
|  |  |  | *Populus trichocarpa* | 86.3 | B9I1V7 |
|  |  |  | *Ricinus communis* | 85.9 | B9SE55 |
| *VvWBC11* | *VvABCG11* | GSVIVT01014733001 | *Arabidopsis thaliana* | 84.8 | Q9LK50 |
|  |  |  | *Oryza sativa ssp japonica* | 74.8 | Q69Q33 |
|  |  |  | *Populus trichocarpa* | 87.6 | B9GG01 |
|  |  |  | *Ricinus communis* | 87.6 | B9S9X8 |
|  |  |  | *Zea mays* | 76.7 | B6U195 |
| *VvWBC14* | *VvABCG14* | GSVIVT01015771001 | *Populus trichocarpa* | 77.7 | B9IJD7 |
|  |  |  | *Ricinus communis* | 77.0 | B9SIL5 |
| *VvWBC15* | *VvABCG15* | GSVIVT01016240001 | *Ricinus communis* | 91.1 | B9SY65 |
|  |  |  | *Populus trichocarpa* | 90.6 | B9HUH9 |
| *VvWBC16* | *VvABCG16* | GSVIVT01020687001 | *Populus trichocarpa* | 81.2 | B9MW36 |
| *VvWBC17* | *VvABCG17* | GSVIVT01022346001 | *Populus trichocarpa* | 81.0 | B9HET0 |
|  |  |  | *Populus trichocarpa* | 80.1 | B9H498 |
| *VvWBC18* | *VvABCG18* | GSVIVT01022526001 | *Populus trichocarpa* | 90.6 | B9IIG9 |
|  |  |  | *Ricinus communis* | 87.7 | B9SDN1 |
|  |  |  | *Arabidopsis thaliana* | 85.7 | Q93YS4 |
|  |  |  | *Oryza sativa ssp indica* | 81.4 | B8ANU1 |
| *VvWBC19* | *VvABCG19* | GSVIVT01024228001 | *Arabidopsis thaliana* | 90.6 | Q8RXN0 |
|  |  |  | *Gossypium hirsutum* | 91.0 | Q6X4V5 |
|  |  |  | *Populus trichocarpa* | 94.8 | B9N7Z1 |
|  |  |  | *Ricinus communis* | 95.3 | B9SI51 |
| *VvWBC22* | *VvABCG22* | GSVIVT01025712001 | *Ricinus communis* | 83.3 | B9S0J4 |
|  |  |  | *Populus trichocarpa* | 82.8 | B9HUH9 |
|  |  |  | *Arabidopsis thaliana* | 84.3 | Q9FF46 |
| *VvWBC23* | *VvABCG23* | GSVIVT01028809001 | *Populus trichocarpa* | 89.6 | B9N349 |
|  |  |  | *Arabidopsis thaliana* | 83.8 | Q9FLX5 |
|  |  |  | *Arabidopsis thaliana* | 82.4 | Q9SW08 |
|  |  |  | *Ricinus communis* | 83.0 | B9RIQ8 |
| *VvWBC24* | *VvABCG24* | GSVIVT01031516001 | *Ricinus communis* | 85.8 | B9SCH2 |
|  |  |  | *Populus trichocarpa* | 83.3 | B9HQM2 |
|  |  |  | *Arabidopsis thaliana* | 92.1 | Q9ZUU9 |
| *VvWBC26* | *VvABCG26* | GSVIVT01031529001 | *Arabidopsis thaliana* | 84.8 | C0Z2H3 |
| *VvWBC27* | *VvABCG27* | GSVIVT01032625001 | *Ricinus communis* | 90.5 | B9SGG5 |
|  |  |  | *Populus trichocarpa* | 89.5 | B9HTY6 |
|  |  |  | *Arabidopsis thaliana* | 86.2 | Q9FNB5 |
|  |  |  | *Nicotina tabacum* | 82.9 | Q69FB5 |
| *VvWBC28* | *VvABCG28* | GSVIVT01034463001 | *Arabidopsis thaliana* | 72.9 | Q84TH5 |
|  |  |  | *Populus trichocarpa* | 87.0 | B9MW88 |
|  |  |  | *Ricinus communis* | 87.4 | B9T8C4 |
| *VvWBC29* | *VABCG29* | GSVIVT01036869001 | *Arabidopsis thaliana* | 89.8 | Q9C6W5 |
|  |  |  | *Oryza sativa ssp japonica* | 76.7 | Q6YYM2 |
|  |  |  | *Populus trichocarpa* | 94.7 | B9GVQ0 |
|  |  |  | *Ricinus communis* | 92.3 | B9S1S7 |
|  |  |  | *Zea mays* | 74.3 | C4J0R6 |
| *VvWBC30* | *VvABCG30* | GSVIVT01037274001 | *Arabidopsis thaliana* | 81.4 | Q3E9B8 |
|  |  |  | *Oryza sativa ssp indica* | 54.0 | B8AXS8 |
|  |  |  | *Populus trichocarpa* | 90.5 | B9HNG6 |
|  |  |  | *Ricinus communis* | 89.6 | B9SY40 |
|  |  |  | *Zea mays* | 55.0 | B6SXQ6 |
| *VvPDR1* | *VvABCG31* | GSVIVT01015456001 | *Populus trichocarpa* | 87.4 | B9ILH5 |
|  |  |  | *Arabidopsis thaliana* | 83.9 | O81016 |
|  |  |  | *Ricinus communis* | 90.1 | B9RP91 |
|  |  |  | *Oryza sativa ssp japonica* | 82.9 | B7E2S5 |
|  |  |  | *Zea mays* | 81.5 | C0PKY7 |
|  |  |  | *Catharantus roseus* | 84.6 | Q5DQU0 |
| *VvPDR7* | *VvABCG37* | GSVIVT01016999001 | *Arabidopsis thaliana* | 81.9 | Q0WM82 |
|  |  |  | *Nicotina tabacum* | 55.0 | Q5W273 |
|  |  |  | *Oryza sativa ssp indica* | 51.0 | B8AJ01 |
|  |  |  | *Populus trichocarpa* | 61.0 | B9H0A6 |
| *VvPDR9* | *VvABCG39* | GSVIVT01017185001 | *Arabidopsis thaliana* | 72.0 | Q9M9E1 |
|  |  |  | *Populus trichocarpa* | 66.0 | B9GWQ9 |
| *VvPDR16* | *VvABCG46* | GSVIVT01017204001 | *Arabidopsis thaliana* | 72.0 | Q9M9E1 |
|  |  |  | *Populus trichocarpa* | 67.0 | B9GWQ9 |
| *VvPDR17* | *VvABCG47* | GSVIVT01017676001 | *Populus trichocarpa* | 85.2 | B9HXH2 |
| *VvPDR19* | *VvABCG49* | GSVIVT01031314001 | *Arabidopsis thaliana* | 77.7 | Q9LFH0 |
|  |  |  | *Nicotina tabacum* | 86.6 | Q5W274 |
|  |  |  | *Populus trichocarpa* | 85.1 | B9IDJ1 |
|  |  |  | *Ricinus communis* | 86.1 | B9RZZ4 |
| *VvPDR20* | *VvABCG50* | GSVIVT01031377001 | *Nicotiana plumbaginifolia* | 87.9 | Q2PCF1 |
|  |  |  | *Ricinus communis* | 87.4 | B9SSW0 |
| *VvPDR21* | *VVABCG51* | GSVIVT01031378001 | *Ricinus communis* | 85.2 | B9SSW1 |
| *VvPDR23* | *VvABCG53* | GSVIVT01033804001 | *Populus trichocarpa* | 87.9 | B9MTQ1 |
|  |  |  | *Ricinus communis* | 86.9 | B9T195 |
| *VvPDR28* | *VvABCG58* | GSVIVT01035715001 | *Arabidopsis thaliana* | 80.6 | O81016 |
|  |  |  | *Oryza sativa ssp indica* | 80.2 | B8ADJ4 |
|  |  |  | *Populus trichocarpa* | 82.7 | B9ILH5 |
|  |  |  | *Ricinus communis* | 82.1 | B9RP91 |
| *VvPDR29* | *VvABCG59* | GSVIVT01035780001 | *Populus trichocarpa* | 84.8 | B9IKS8 |
|  |  |  | *Ricinus communis* | 82.9 | B9RJZ6 |
|  |  |  | *Nicotina tabacum* | 83.9 | Q5W273 |
| *VvNAP1* | *VvABCI1* | GSVIVT01010853001 | *Arabidopsis thaliana* | 71.0 | Q9LTR2 |
|  |  |  | *Oryza sativa ssp japonica* | 62.0 | Q5JL23 |
|  |  |  | *Populus trichocarpa* | 77.0 | B9HGZ1 |
|  |  |  | *Ricinus communis* | 76.0 | B9T7S8 |
|  |  |  | *Zea mays* | 63.0 | B6TXI1 |
| *VvNAP2* | *VvABCI2* | GSVIVT01012742001 | *Ricinus communis* | 96.9 | B9RXM8 |
|  |  |  | *Populus trichocarpa* | 95.6 | B9H1C6 |
|  |  |  | *Nicotina tabacum* | 91.6 | Q58IU5 |
|  |  |  | *Arabidopsis thaliana* | 88.9 | Q9ZS97 |
|  |  |  | *Oryza sativa ssp indica* | 84.1 | A2WWL8 |
|  |  |  | *Zea mays* | 82.7 | C0P9D7 |
| *VvNAP3* | *VvABCI3* | GSVIVT01013180001 | *Arabidopsis thaliana* | 63.0 | Q058Q9 |
|  |  |  | *Oryza sativa subsp. indica* | 53.0 | B8AD17 |
|  |  |  | *Populus trichocarpa* | 79.0 | B9N2P8 |
|  |  |  | *Ricinus communis* | 68.0 | B9T968 |
| *VvNAP4* | *VvABCI4* | GSVIVT01017866001 | *Populus trichocarpa* | 72.0 | B9HMA8 |
| *VvSMC1* |  | GSVIVT01011408001 | *Populus trichocarpa* | 81.2 | B9IJY0 |
|  |  |  | *Ricinus communis* | 80.2 | B9T849 |
| *VvSMC2* |  | GSVIVT01018715001 | *Populus trichocarpa* | 79.0 | B9H079 |
|  |  |  | *Ricinus communis* | 79.0 | B9R9T8 |
| *VvSMC3* |  | GSVIVT01028343001 | *Ricinus communis* | 80.3 | B9T1A8 |
