# Supplementary material for: Whole-Genome Survey of the Putative ATP-Binding Cassette Transporter Family Genes in Vitis vinifera
Source: PLoS One. 2013 Nov 11;8(11):e78860. doi: 10.1371/journal.pone.0078860 (PMC3823996; doi:10.1371/journal.pone.0078860)
Supplement: Table S4 — Expressed sequence tags (ESTs) identified for the ABCA (ATH and AOH) subfamily in Vitis vinifera . The protein name, Vitis proteome 12× ID, GenBank ID, EST name, cultivar/tissue type, and development stage are given for each gene. (DOC) [file pone.0078860.s004.doc]

**Table S4.** Expressed sequence tags (ESTs) identified for the ABCA (ATH and AOH) subfamily in *Vitis vinifera*. The protein name, *Vitis* proteome 12× ID, GenBank ID, EST name, cultivar/tissue type, and development stage are given for each gene.

| **Name** | | ***Vitis* 12X ID** | **EST Name** | **GenBank ID** | **Species/Cultivar** | **Tissue Type** | **Development Stage** |
| --- | --- | --- | --- | --- | --- | --- | --- |
| *VvAOH1* | *VvABCA1* | GSVIVT01033258001 | VVD067A05_351999 | CB920683 | Chardonnay | Berries | Mixed; 8, 9, 11, 13, 15, 16 weeks daf |
|  |  |  | WIN0415.C21_F11 | EC932100 | Cabernet Sauvignon | Pericarp | Fruit set to maturity |
|  |  |  | VVA017D07_392405 | CD004406 | Chardonnay | Leaf | Juvenile and adult |
|  |  |  | RR890915N0006_IIa_Ra_C08 | 33397092 | Vitis hybrid cultivar | Leaf | 17-week old greenhouse grown plants |
|  |  |  | VVA017D07_54095 | 18459022 | Chardonnay | Leaf | Juvenile and adult |
|  |  |  | WIN021.C21_F20 | 110364174 | Cabernet Sauvignon | Flower, leaf and root | Flower, pre-anthesis; leaf, fully expanded; root, produced by air-layering |
| *VvATH1* | *VvABCA2* | GSVIVT01008040001 | FAMU_USDA_FP_3382 | 51577523 | Vitis shuttleworthii | Entire tendril, leaves, bud, flowers | At blooming |
|  |  |  | BACCA01_000750 | 37184113 | Pinot Noir | Berry | Veraison |
|  |  |  | WIN1018.C21_M20 | 110408731 | Muscat Hamburg | Pericarp | Fruit set to maturity |
| *VvATH2* | *VvABCA3* | GSVIVT01008042001 | WIN0813.C21_P22 | 110396572 | Cabernet Sauvignon | Seed | Fruit set to maturity |
|  |  |  | BACCA01_001574 | 37184882 | Pinot Noir | Berry | Veraison |
|  |  |  | WIN0216.TB24_L18 | 110364066 | Cabernet Sauvignon | Flower, leaf and root | Flower, pre-anthesis; leaf, fully expanded; root, producedby air-layering |
|  |  |  | BQ797081 | 22012047 | Shiraz | Fruit | Ripening stage |
|  |  |  | WIN0537.C21_L20 | 110380814 | Cabernet Sauvignon | Flower, leaf and root | Flower, pre-anthesis; leaf, fully expanded; root, producedby air-layering |
|  |  |  | WIN103.C21_K21 | 110405547 | Muscat Hamburg | Pericarp | Fruit set to maturity |
|  |  |  | BQ798306 | 22013272 | Shiraz | Fruit | Green stage |
|  |  |  | WIN0559.C21_D22 | 110388818 | Cabernet Sauvignon | Flower, leaf and root | Flower, pre-anthesis; leaf, fully expanded; root, producedby air-layering |
|  |  |  | CSECS022G06_PREu0032 | 34362594 | Cabernet Sauvignon | Fruit with seeds removed | 32 - modified E-L system |
|  |  |  | CN546953 | 46917624 | Shiraz | Fruit without seeds | Green stage |
|  |  |  | sT7aVVM_AER7G03 | 161709915 | Cabernet Sauvignon | Roots | 10 cm high plants grown in Magenta boxes |
|  |  |  | CA32EN0003_IVaR_F05 | 28963628 | Cabernet Sauvignon | Leaf | Mid-season leaf material, collected July 25, 2001 |
|  |  |  | CA32EN0003_IVaF_F05 | 28963553 | Cabernet Sauvignon | Leaf | Mid-season leaf material, collected July 25, 2001 |
|  |  |  | BQ795846 | 22010812 | Shiraz | Fruit | Ripening stage |
| VvATH3 | VvABCA3 | GSVIVT01025902001 | WIN058.C21_L18 | 110374411 | Cabernet Sauvignon | Flower, leaf and root | Flower, pre-anthesis; leaf, fully expanded; root, producedby air-layering |
|  |  |  | FAMU_USDA_FP_3382 | 51577523 | Vitis shuttleworthii | Entire tendril, leaves, bud, flowers | At blooming |
| VvATH4 | VvABCA4 | GSVIVT01033860001 | CAbud0002_IIIR_D07 | 34543280 | Cabernet Sauvignon | Bud | Pre-bloom (10-11 days before bloom) |
|  |  |  | WIN1133.C21_D19 | 122692027 | Muscat Hamburg | Berry | Anthesis flower to prior to veraison |
