# Supplementary material for: Whole-Genome Survey of the Putative ATP-Binding Cassette Transporter Family Genes in Vitis vinifera
Source: PLoS One. 2013 Nov 11;8(11):e78860. doi: 10.1371/journal.pone.0078860 (PMC3823996; doi:10.1371/journal.pone.0078860)
Supplement: Table S5 — Expressed sequence taqs (ESTs) identified for ABCB (MDR, TAP and ATM) subfamily in Vitis vinifera . The protein name, Vitis proteome 12x ID, GenBank ID, EST name, cultivar/tissue type, and development stage are given for each gene. (DOC) [file pone.0078860.s005.doc]

**Table S5.** Expressed sequence taqs (ESTs) identified for ABCB (MDR, ATM and TAP) subfamily in *Vitis vinifera*.The protein name, *Vitis* proteome 12x ID, GenBank ID, EST name, cultivar/tissue type, and development stage are given for each gene.

| **Name** | | ***Vitis* 12X ID** | **EST Name** | **GenBank ID** | **Species/Cultivar** | **Tissue Type** | **Development Stage** |
| --- | --- | --- | --- | --- | --- | --- | --- |
| *VvMDR1* | *VvABCB1* | GSVIVT01000580001 | WIN0525.C21_B19 | 110377155 | Cabernet Sauvignon | Flower, leaf and root | Flower, pre-anthesis; leaf, fully expanded; root, produced by air-layering |
|  |  |  | WIN114.C21_B11 | 122691548 | Muscat Hamburg | Berry | Anthesis flower to prior to veraison |
|  |  |  | S5B03129 | 110709399 | Thompson-seedless | Fruit | Fruits 7-9 mm |
| *VvMDR2* | *VvABCB2* | GSVIVT01007586001 | VVD024F01_346371 | 30132530 | Chardonnay | Berries | Mixed; 8, 9, 11, 13, 15, 16 weeks daf |
| *VvMDR3* | *VvABCB3* | GSVIVT01009946001 | sT7aVV01003X1G04 | 161721704 | Cabernet Sauvignon | Roots | 10 cm high plants grown in Magenta boxes |
|  |  |  | WIN022.C21_O24 | 122688702 | Cabernet Sauvignon | Flower, leaf and root | Flower, pre-anthesis; leaf, fully expanded; root, produced by air-layering |
|  |  |  | WIN114.C21_B11 | 122691548 | Muscat Hamburg | Berry | Anthesis flower to prior to veraison |
|  |  |  | S2B22295 | 110703497 | Thompson-seedless | Bud |  |
|  |  |  | FAMU_USDA_FP_5929 | 51580070 | Vitis shuttleworthii | Entire tendril, leaves, bud, flowers | At blooming |
| *VvMDR4* | *VvABCB4* | GSVIVT01011381001 | SBB06249 | 110728671 | Thompson-seedless | Inflorescence |  |
|  |  |  | WIN029.TB24_M04 | 110362189 | Cabernet Sauvignon | Flower, leaf and root | Flower, pre-anthesis; leaf, fully expanded; root, produced by air-layering |
|  |  |  | sT7aVVM015B18079 | 161716508 | Cabernet Sauvignon | Roots | 10 cm high plants grown in Magenta boxes |
|  |  |  | sT7aVV01003X1G04 | 161721704 | Cabernet Sauvignon | Roots | 10 cm high plants grown in Magenta boxes |
|  |  |  | WIN0515.C21_C16 | 110373115 | Cabernet Sauvignon | Hermaphrodite | Flower, leaf and root |
|  |  |  | FAMU_USDA_FP_4751 | 51578892 | Vitis shuttleworthii | Entire tendril, leaves, bud, flowers | At blooming |
|  |  |  | FAMU_USDA_FP_625 | 51574766 | Vitis shuttleworthii | Entire tendril, leaves, bud, flowers | At blooming |
|  |  |  | CAB40007_Ib_Rb_C12 | 30302789 | Cabernet Sauvignon | Berry | Berry on stage II, 9 mm |
|  |  |  | CAB20003_IIIa_Fa_H11 | 33402646 | Cabernet Sauvignon | Flower - bloom | Bloom |
|  |  |  | WIN0558.C21_K05 | 110388638 | Cabernet Sauvignon | Flower, leaf and root | Flower, pre-anthesis; leaf, fully expanded; root, produced by air-layering |
|  |  |  | CSECS183H08_INFu0031 | 83274705 | Cabernet Sauvignon | Inflorescence | 31 - modified E-L system |
|  |  |  | GEMMA01_001778 | 37188883 | Pinot Noir | Bud | Bud swelling |
|  |  |  | WIN094.C21_G22 | 110404080 | Muscat Hamburg | Pericarp | Fruit set to maturity |
|  |  |  | CAP0007_IF_A04 | 34550112 | Cabernet Sauvignon | Petiole | Onset of Veraison (berry softening) |
|  |  |  | C2B04987 | 110689657 | Carmenere | Bud - cluster |  |
|  |  |  | WIN102.C21_I14 | 110405209 | Muscat Hamburg | Pericarp | Fruit set to maturity |
|  |  |  | S2B22295 | 110703497 | Thompson-seedless | Bud |  |
|  |  |  | VVI014A11_585010 | 71868530 | Cabernet Sauvignon | Inflorescence including flower | 12 - modified E-L system |
|  |  |  | VVD090G04_356633 | 30137662 | Chardonnay | Berries | Mixed; 8, 9, 11, 13, 15, 16 weeks daf |
| *VvMDR5* | *VvABCB5* | GSVIVT01013125001 | CAB40007_Ib_Rb_C12 | 30302789 | Cabernet Sauvignon | Berry | Berry on stage II, 9 mm |
|  |  |  | VVD090G04_356633 | 30137662 | Chardonnay | Berries | Mixed; 8, 9, 11, 13, 15, 16 weeks daf |
|  |  |  | CAbud0005_IIF_A02 | 34546320 | Cabernet Sauvignon | Bud | Pre-bloom (10-11 days before bloom) |
|  |  |  | VVA019F01_402667 | 32245250 | Chardonnay | Leaf | Juvenile and adult |
|  |  |  | CAB30005_Iia_Ra_B09 | 30297813 | Cabernet Sauvignon | Berry | Berry stage I |
|  |  |  | VVC008B06_126230 | 27582405 | Chardonnay | Berries | Mixed; 8, 9, 11, 13, 15, 16 weeks daf |
|  |  |  | VVA019F01_54375 | 18459162 | Chardonnay | Leaf | Juvenile and adult |
|  |  |  | Cabud0005_IIR_A02 | 34546404 | Cabernet Sauvignon | Bud | Pre-bloom (10-11 days before bloom) |
|  |  |  | S6B05186 | 110713536 | Thompson-seedless | Fruit |  |
| *VvMDR6* | *VvABCB6* | GSVIVT01014625001 | S2B22295 | 110703497 | Thompson-seedless | Bud |  |
|  |  |  | VVD024F01_346371 | 30132530 | Chardonnay | Berries | Mixed; 8, 9, 11, 13, 15, 16 weeks daf |
|  |  |  | sT7aVV01003X1G04 | 161721704 | Cabernet Sauvignon | Roots | 10 cm high plants grown in Magenta boxes |
| *VvMDR7* | *VvABCB7* | GSVIVT01015306001 | sT7aVVM015B18079 | 161716508 | Cabernet Sauvignon | Roots | 10 cm high plants grown in Magenta boxes |
|  |  |  | WIN0525.C21_B19 | 110377155 | Cabernet Sauvignon | Flower, leaf and root | Flower, pre-anthesis; leaf, fully expanded; root, produced by air-layering |
| *VvMDR8* | *VvABCB8* | GSVIVT01016617001 | VVTOV304 | 160482015 | Thompson-seedless | Ovule | Mixed stages 27-48d after bloom |
|  |  |  | WIN1116.C21_M07 | 110416718 | Muscat Hamburg | Berry | Anthesis flower to prior to veraison |
|  |  |  | VV_PEb02c06.b1 | 156728371 | Perlette | Bud | Mature |
|  |  |  | VV_PEb02c06.g1 | 156728372 | Perlette | Bud | Mature |
|  |  |  | CAB20003_Ia_Fa_D03 | 33403121 | Cabernet Sauvignon | Flower | Bloom |
|  |  |  | WIN1033.C21_C18 | 110411276 | Muscat Hamburg | Pericarp | Fruit set to maturity |
|  |  |  | S2B12108 | 110703343 | Thompson-seedless | Bud |  |
|  |  |  | FAMU_USDA_FP_833 | 51574974 | Vitis shuttleworthii | Entire tendril, leaves, bud, flowers | At blooming |
|  |  |  | VVB092G11_339081 | 30325519 | Chardonnay | Leaf | Juvenile and adult |
|  |  |  | VVB096E04_341129 | 30326543 | Chardonnay | Leaf | Juvenile and adult |
|  |  |  | VVB193B12_430727 | 32247911 | Chardonnay | Leaf | Juvenile and adult |
| *VvMDR9* | *VvABCB9* | GSVIVT01016706001 | sT7aVV01003X1G04 | 161721704 | Cabernet Sauvignon | Roots | 10 cm high plants grown in Magenta boxes |
| *VvMDR10* | *VvABCB10* | GSVIVT01017696001 | C2B04987 | 110689657 | Carmenere | Bud - cluster |  |
|  |  |  | sT7aVVM_AER24E03 | 161711125 | Cabernet Sauvignon | Roots | 10 cm high plants grown in Magenta boxes |
|  |  |  | WIN0532.C21_G05 | 110379431 | Cabernet Sauvignon | Flower, leaf and root | Flower, pre-anthesis; leaf, fully expanded; root, produced by air-layering |
|  |  |  | sT7aVV01003X1G04 | 161721704 | Cabernet Sauvignon | Roots | 10 cm high plants grown in Magenta boxes |
|  |  |  | CSECS017H05_PREu0032 | 34362391 | Cabernet Sauvignon | Fruit with seeds removed | 32 - modified E-L system |
|  |  |  | WIN0532.C21_G05 | 110379431 | Cabernet Sauvignon | Flower, leaf and root | Flower, pre-anthesis; leaf, fully expanded; root, produced by air-layering |
|  |  |  | GEMMA01_001778 | 37188883 | Pinot Noir | Bud | Bud swelling |
|  |  |  | sT7aVVM_AER24E03 | 161711125 | Cabernet Sauvignon | Roots | 10 cm high plants grown in Magenta boxes |
| *VvMDR11* | *VvABCB11* | GSVIVT01021365001 | sT7aVV01003X1G04 | 161721704 | Cabernet Sauvignon | Roots | 10 cm high plants grown in Magenta boxes |
|  |  |  | sT7aVVM_AER24E03 | 161711125 | Cabernet Sauvignon | Roots | 10 cm high plants grown in Magenta boxes |
|  |  |  | WIN0525.C21_B19 | 110377155 | Cabernet Sauvignon | Flower, leaf and root | Flower, pre-anthesis; leaf, fully expanded; root, produced by air-layering |
| *VvMDR12* | *VvABCB12* | GSVIVT01021366001 | sT7aVV01003X1G04 | 161721704 | Cabernet Sauvignon | Roots | 10 cm high plants grown in Magenta boxes |
|  |  |  | sT7aVVM_AER24E03 | 161711125 | Cabernet Sauvignon | Roots | 10 cm high plants grown in Magenta boxes |
|  |  |  | WIN0525.C21_B19 | 110377155 | Cabernet Sauvignon | Flower, leaf and root | Flower, pre-anthesis; leaf, fully expanded; root, produced by air-layering |
|  |  |  | C2B04987 | 110689657 | Carmenere | Bud - cluster |  |
|  |  |  | sT7aVV01003X1G04 | 161721704 | Cabernet Sauvignon | Roots | 10 cm high plants grown in Magenta boxes |
|  |  |  | WIN0525.C21_B19 | 110377155 | Cabernet Sauvignon | Flower, leaf and root | Flower, pre-anthesis; leaf, fully expanded; root, produced by air-layering |
| *VvMDR13* | *VvABCB13* | GSVIVT01025040001 | VVB096E04_341129 | 30326543 | Chardonnay | Leaf | Juvenile and adult |
|  |  |  | FAMU_USDA_FP_833 | 51574974 | Vitis shuttleworthii | Entire tendril, leaves, bud, flowers | At blooming |
|  |  |  | WIN0529.C21_H18 | 110378510 | Cabernet Sauvignon | Flower, leaf and root | Flower, pre-anthesis; leaf, fully expanded; root, produced by air-layering |
|  |  |  | S2B12108 | 110703343 | Thompson-seedless | Bud |  |
|  |  |  | VVB096E04_341129 | 30326543 | Chardonnay | Leaf | Juvenile and adult |
|  |  |  | S2B12108 | 110703343 | Thompson-seedless | Bud |  |
|  |  |  | FAMU_USDA_FP_833 | 51574974 | Vitis shuttleworthii | Entire tendril, leaves, bud, flowers | At blooming |
|  |  |  | WIN0529.C21_H18 | 110378510 | Cabernet Sauvignon | Flower, leaf and root | Flower, pre-anthesis; leaf, fully expanded; root, produced by air-layering |
| *VvMDR14* | *VvABCB14* | GSVIVT01028256001 | CSECS017H05_PREu0032 | 34362391 | Cabernet Sauvignon | Fruit with seeds removed | 32 - modified E-L system |
|  |  |  | sT7aVVM027F01011 | 161720784 | Cabernet Sauvignon | Roots | 10 cm high plants grown in Magenta boxes |
|  |  |  | WIN0532.C21_G05 | 110379431 | Cabernet Sauvignon | Flower, leaf and root | Flower, pre-anthesis; leaf, fully expanded; root, produced by air-layering |
|  |  |  | CAB40007_Ib_Rb_C12 | 30302789 | Cabernet Sauvignon | Berry | Berry on stage II, 9 mm |
|  |  |  | VV_PEa25a07.g1 | 156728004 | Perlette | Bud | Mature |
|  |  |  | sT7aVVM_AER24E03 | 161711125 | Cabernet Sauvignon | Roots | 10 cm high plants grown in Magenta boxes |
|  |  |  | S2B22295 | 110703497 | Thompson-seedless | Bud |  |
| *VvMDR15* | *VvABCB15* | GSVIVT01032578001 | sT7aVVM_AER79D01 | 161705921 | Cabernet Sauvignon | Roots | 10 cm high plants grown in Magenta boxes |
|  |  |  | sT7aVVM019A22095 | 161717320 | Cabernet Sauvignon | Roots | 10 cm high plants grown in Magenta boxes |
|  |  |  | WIN1131.C21_E07 | 110421335 | Muscat Hamburg | Berry | Anthesis flower to prior to veraison |
|  |  |  | WIN0529.C21_L24 | 122689746 | Cabernet Sauvignon | Flower, leaf and root | Flower, pre-anthesis; leaf, fully expanded; root, produced by air-layering |
|  |  |  | WIN0540.C21_H24 | 122690047 | Cabernet Sauvignon | Flower, leaf and root | Flower, pre-anthesis; leaf, fully expanded; root, produced by air-layering |
|  |  |  | sT7aVVM026B23096 | 161720517 | Cabernet Sauvignon | Roots | 10 cm high plants grown in Magenta boxes |
|  |  |  | sT7aVVM015B18079 | 161716508 | Cabernet Sauvignon | Roots | 10 cm high plants grown in Magenta boxes |
|  |  |  | sT7aVVM019J10039 | 161719515 | Cabernet Sauvignon | Roots | 10 cm high plants grown in Magenta boxes |
|  |  |  | WIN026.TB24.1_D01 | 122688726 | Cabernet Sauvignon | Flower, leaf and root | Flower, pre-anthesis; leaf, fully expanded; root, produced by air-layering |
|  |  |  | sT7aVVM002J09039 | 161712360 | Cabernet Sauvignon | Roots | 10 cm high plants grown in Magenta boxes |
|  |  |  | sT7aVVM014P24082 | 161715755 | Cabernet Sauvignon | Roots | 10 cm high plants grown in Magenta boxes |
|  |  |  | sT7aVVM007J14055 | 161715153 | Cabernet Sauvignon | Roots | 10 cm high plants grown in Magenta boxes |
|  |  |  | sT7aVVM015G23090 | 161718387 | Cabernet Sauvignon | Roots | 10 cm high plants grown in Magenta boxes |
|  |  |  | sT7aVVM014P24082 | 161715755 | Cabernet Sauvignon | Roots | 10 cm high plants grown in Magenta boxes |
|  |  |  | sT7aVVM026B23096 | 161720517 | Cabernet Sauvignon | Roots | 10 cm high plants grown in Magenta boxes |
|  |  |  | WIN0540.C21_H24 | 122690047 | Cabernet Sauvignon | Flower, leaf and root | Flower, pre-anthesis; leaf, fully expanded; root, produced by air-layering |
|  |  |  | sT7aVVM007J14055 | 161715153 | Cabernet Sauvignon | Roots | 10 cm high plants grown in Magenta boxes |
|  |  |  | sT7aVVM015B18079 | 161716508 | Cabernet Sauvignon | Roots | 10 cm high plants grown in Magenta boxes |
|  |  |  | sT7aVVM019J10039 | 161719515 | Cabernet Sauvignon | Roots | 10 cm high plants grown in Magenta boxes |
|  |  |  | sT7aVVM015G23090 | 161718387 | Cabernet Sauvignon | Roots | 10 cm high plants grown in Magenta boxes |
|  |  |  | WIN0529.C21_L24 | 122689746 | Cabernet Sauvignon | Flower, leaf and root | Flower, pre-anthesis; leaf, fully expanded; root, produced by air-layering |
|  |  |  | WIN1131.C21_E07 | 110421335 | Muscat Hamburg | Berry | Anthesis flower to prior to veraison |
|  |  |  | sT7aVVM019A22095 | 161717320 | Cabernet Sauvignon | Roots | 10 cm high plants grown in Magenta boxes |
|  |  |  | sT7aVVM_AER79D01 | 161705921 | Cabernet Sauvignon | Roots | 10 cm high plants grown in Magenta boxes |
|  |  |  | sT7aVVM002J09039 | 161712360 | Cabernet Sauvignon | Roots | 10 cm high plants grown in Magenta boxes |
|  |  |  | WIN026.TB24.1_D01 | 122688726 | Cabernet Sauvignon | Flower, leaf and root | Flower, pre-anthesis; leaf, fully expanded; root, produced by air-layering |
| *VvMDR16* | *VvABCB16* | GSVIVT01032898001 | sT7aVVM_AER12A11 | 161708365 | Cabernet Sauvignon | Roots | 10 cm high plants grown in Magenta boxes |
|  |  |  | sT7aVVM_AER11H11 | 161710662 | Cabernet Sauvignon | Roots | 10 cm high plants grown in Magenta boxes |
|  |  |  | sT7aVVM020K18069 | 161716179 | Cabernet Sauvignon | Roots | 10 cm high plants grown in Magenta boxes |
|  |  |  | sT7aVVM_AER44H10 | 161710848 | Cabernet Sauvignon | Roots | 10 cm high plants grown in Magenta boxes |
|  |  |  | FAMU_USDA_FP_1198 | 51575339 | Vitis shuttleworthii | Entire tendril, leaves, bud, flowers | At blooming |
|  |  |  | FAMU_USDA_FP_1198 | 51575339 | Vitis shuttleworthii | Entire tendril, leaves, bud, flowers | At blooming |
|  |  |  | VVA019E07_54365 | 18459157 | Chardonnay | Leaf | Juvenile and adult |
|  |  |  | CAP0002_IIIF_F12 | 34547701 | Cabernet Sauvignon | Petiole | Onset of Veraison (berry softening) |
|  |  |  | WIN027.TB24.1_M04 | 110361618 | Cabernet Sauvignon | Flower, leaf and root | Flower, pre-anthesis; leaf, fully expanded; root, produced by air-layering |
|  |  |  | WIN0533.C21_L16 | 110379857 | Cabernet Sauvignon | Flower, leaf and root | Flower, pre-anthesis; leaf, fully expanded; root, produced by air-layering |
|  |  |  | VVL087H12_689602 | 71884933 | Cabernet Sauvignon | Fruit with seeds removed | Mixed 36-38 - modified E-L system (Brix > 15) |
|  |  |  | WIN027.TB24.1_H06 | 110361530 | Cabernet Sauvignon | Flower, leaf and root | Flower, pre-anthesis; leaf, fully expanded; root, produced by air-layering |
|  |  |  | VVL086B07_689286 | 71884775 | Cabernet Sauvignon | Fruit with seeds removed | Mixed 36-38 - modified E-L system (Brix > 15) |
|  |  |  | VVL131F01_697168 | 71888716 | Cabernet Sauvignon | Fruit with seeds removed | Mixed 36-38 - modified E-L system (Brix > 15) |
|  |  |  | VVL125H06_696204 | 71888234 | Cabernet Sauvignon | Fruit with seeds removed | Mixed 36-38 - modified E-L system (Brix > 15) |
|  |  |  | VVL124C04_695916 | 71888090 | Cabernet Sauvignon | Fruit with seeds removed | Mixed 36-38 - modified E-L system (Brix > 15) |
|  |  |  | VVL086B07_689286 | 71884775 | Cabernet Sauvignon | Fruit with seeds removed | Mixed 36-38 - modified E-L system (Brix > 15) |
|  |  |  | S1G03330 | 110699209 | Thompson-seedless | Fruit and flower |  |
|  |  |  | WIN074.C21_E11 | 110394417 | Cabernet Sauvignon | Pericarp | Fruit set to maturity |
|  |  |  | VVA019E07_54365 | 18459157 | Chardonnay | Leaf | Juvenile and adult |
|  |  |  | VVD064H07_351705 | 30135198 | Chardonnay | Berries | Mixed; 8, 9, 11, 13, 15, 16 weeks daf |
|  |  |  | CAP0002_IIIR_F12 | 34547772 | Cabernet Sauvignon | Petiole | Onset of Veraison (berry softening) |
| *VvMDR17* | *VvABCB17* | GSVIVT01033645001 | WIN1150.C21_J13 | 110428068 | Muscat Hamburg | Berry | Anthesis flower to prior to veraison |
|  |  |  | WIN0541.C21_E07 | 110382969 | Cabernet Sauvignon | Flower, leaf and root | Flower, pre-anthesis; leaf, fully expanded; root, produced by air-layering |
|  |  |  | VVI174D05_610914 | 77581392 | Cabernet Sauvignon | Inflorescence including flowers | 12 - modified E-L system |
|  |  |  | CAST0001_IIIF_G12 | 33409407 | Cabernet Sauvignon | Stem | Pre-bloom (10-11 days before bloom) |
|  |  |  | S5B03129 | 110709399 | Thompson-seedless | Fruit |  |
|  |  |  | CAST0001_IIIR_G12 | 33409492 | Cabernet Sauvignon | Stem | Pre-bloom (10-11 days before bloom) |
|  |  |  | SBB04122 | 110728199 | Thompson-seedless | Inflorescence |  |
|  |  |  | VVI099G01_598234 | 71866328 | Cabernet Sauvignon | Inflorescence including flowers | 12 - modified E-L system |
|  |  |  | SCB05621 | 110730637 | Thompson-seedless | Inflorescence |  |
|  |  |  | FAMU_USDA_FP_5447 | 51579588 | Vitis shuttleworthii | Entire tendril, leaves, bud, flowers | At blooming |
|  |  |  | Fag-B-IES1,5h-K1-93 | 118498750 | Vitis cinerea x Vitis riparia | Roots | Adult plants |
|  |  |  | FAMU_USDA_FP_5804 | 51579945 | Vitis shuttleworthii | Entire tendril, leaves, bud, flowers | At blooming |
|  |  |  | CAB10004_IIa_Ra_F05 | 30253324 | Cabernet Sauvignon | Flower | Pre-bloom |
|  |  |  | sT7aVVM_AER50E04 | 161708259 | Cabernet Sauvignon | Roots | 10 cm high plants grown in Magenta boxes |
|  |  |  | SCB05705 | 110732521 | Thompson-seedless | Inflorescence |  |
|  |  |  | VVI141B03_605236 | 71876999 | Cabernet Sauvignon | Inflorescence including flowers | 12 - modified E-L system |
|  |  |  | FAMU_USDA_FP_7275 | 51581416 | Vitis shuttleworthii | Entire tendril, leaves, bud, flowers | At blooming |
|  |  |  | CAB10004_IIa_Fa_F05 | 30253246 | Cabernet Sauvignon | Flower | Pre-bloom |
|  |  |  | VVI014A11_585010 | 71868530 | Cabernet Sauvignon | Inflorescence including flowers | 12 - modified E-L system |
|  |  |  | S2B10444 | 110699988 | Thompson-seedless | Bud |  |
|  |  |  | sT7aVVM026C16062 | 161720548 | Cabernet Sauvignon | Roots | 10 cm high plants grown in Magenta boxes |
|  |  |  | WIN029.TB24_M04 | 110362189 | Cabernet Sauvignon | Flower, leaf and root | Flower, pre-anthesis; leaf, fully expanded; root, produced by air-layering |
|  |  |  | EST 1103 | 22013900 | Shiraz | Fruit | Green stage |
|  |  |  | SBB06211 | 110727539 | Thompson-seedless | Inflorescence |  |
|  |  |  | CAB20003_IIIa_Fa_H11 | 33402646 | Cabernet Sauvignon | Flower | Bloom |
|  |  |  | C1G02747 | 110685628 | Carmenere | Fruit - bud - clusters |  |
|  |  |  | CAB30006_Id_Fe_D12 | 30298553 | Cabernet Sauvignon | Berry | Berry stage 1 |
|  |  |  | CAB30006_Ib_Rb_D12 | 30298495 | Cabernet Sauvignon | Berry | Berry stage 1 |
|  |  |  | WIN0535.C21_H15 | 110380413 | Cabernet Sauvignon | Flower, leaf and root | Flower, pre-anthesis; leaf, fully expanded; root, produced by air-layering |
|  |  |  | WIN1118.C21_M02 | 110417344 | Muscat Hamburg | Berry | Anthesis flower to prior to veraison |
|  |  |  | WIN1136.C21_C22 | 110422956 | Muscat Hamburg | Berry | Anthesis flower to prior to veraison |
|  |  |  | VVI134A01_604002 | 71876382 | Cabernet Sauvignon | Inflorescence including flowers | 12 - modified E-L system |
|  |  |  | CAB20003_IIIa_Ra_H11 | 33402736 | Cabernet Sauvignon | Flower | Bloom |
|  |  |  | SCB01403 | 110731533 | Thompson-seedless | Inflorescence |  |
| *VvMDR18* | *VvMDR18* | GSVIVT01036801001 | sT7aVVM_AER79D01 | 161705921 | Cabernet Sauvignon | Roots | 10 cm high plants grown in Magenta boxes |
|  |  |  | sT7aVVM026B23096 | 161720517 | Cabernet Sauvignon | Roots | 10 cm high plants grown in Magenta boxes |
|  |  |  | sT7aVVM015G23090 | 161718387 | Cabernet Sauvignon | Roots | 10 cm high plants grown in Magenta boxes |
|  |  |  | sT7aVVM014P24082 | 161715755 | Cabernet Sauvignon | Roots | 10 cm high plants grown in Magenta boxes |
|  |  |  | sT7aVVM002J09039 | 161712360 | Cabernet Sauvignon | Roots | 10 cm high plants grown in Magenta boxes |
|  |  |  | WIN026.TB24.1_D01 | 122688726 | Cabernet Sauvignon | Flower, leaf and root | Flower, pre-anthesis; leaf, fully expanded; root, produced by air-layering |
|  |  |  | WIN0540.C21_H24 | 122690047 | Cabernet Sauvignon | Flower, leaf and root | Flower, pre-anthesis; leaf, fully expanded; root, produced by air-layering |
|  |  |  | S2B22295 | 110703497 | Thompson-seedless | Bud |  |
| *VvMDR19* | *VvABCB19* | GSVIVT01038687001 | WIN114.C21_B11 | 122691548 | Muscat Hamburg | Berry | Anthesis flower to prior to veraison |
|  |  |  | WIN1150.C21_J13 | 110428068 | Muscat Hamburg | Berry | Anthesis flower to prior to veraison |
| *VvATM1* | *VvABCB20* | GSVIVT01024527001 | CAB10006_IIa_ra_C12 | 30256194 | Cabernet Sauvignon | Flower | Pre-Bloom |
|  |  |  | CAB20002_IIIa_Fa_H06 | 33402054 | Cabernet Sauvignon | Flower | Bloom |
|  |  |  | WIN0419.C21_D07 | 110369586 | Cabernet Sauvignon | Pericarp | Fruit set to maturity |
|  |  |  | VVA006F06_390853 | 30320368 | Chardonnay | Leaf | Juvenile and Adult |
|  |  |  | VVI111F02_600214 | 71874488 | Cabernet Sauvignon | Inflorescence including flowers | 12 - modified E-L system |
|  |  |  | EE076310 | 110698612 | Thompson-seedless | Fruit and flower |  |
|  |  |  | FAMU_USDA_FP_231 | 51574372 | Vitis shuttleworthii | Entire tendril, leaves, bud, flowers | At blooming |
|  |  |  | VVA006F06_52757 | 18458309 | Chardonnay | Leaf | Juvenile and Adult |
|  |  |  | CAB10006_IIa_Fa_C12 | 30256126 | Cabernet Sauvignon | Flower | Pre-Bloom |
|  |  |  | CAB70005_IIIaF_A01 | 30304818 | Cabernet Sauvignon | Berry | Post-Veraison, 18-19 brix |
|  |  |  | CAB70005_IIIaR_A01 | 30304891 | Cabernet Sauvignon | Berry | Post-Veraison, 18-19 brix |
| *VvTAP1* | *VvABCB21* | GSVIVT01005757001 | WIN1144.C21_F03 | 110425659 | Muscat Hamburg | Berry | Anthesis flower to prior to veraison |
|  |  |  | VVB201H07_432305 | 32248700 | Chardonnay | Leaf | Juvenile and adult |
|  |  |  | VVB080H07_334974 | 30324661 | Chardonnay | Leaf | Juvenile and adult |
|  |  |  | VVB202G08_432465 | 32248780 | Chardonnay | Leaf | Juvenile and adult |
|  |  |  | VVB078E01_334544 | 30324446 | Chardonnay | Leaf | Juvenile and adult |
|  |  |  | WIN0513.C21_I06 | 110372609 | Cabernet Sauvignon | Flower, leaf and root | Flower, pre-anthesis; leaf, fully expanded; root, produced by air-layering |
|  |  |  | WIN0574.C21_M15 | 110391791 | Cabernet Sauvignon | Flower, leaf and root | Flower, pre-anthesis; leaf, fully expanded; root, produced by air-layering |
|  |  |  | BACCA01_000217 | 37183650 | Pinot Noir | Berry | Veraison |
|  |  |  | WIN0546.C21_B18 | 122690190 | Cabernet Sauvignon | Flower, leaf and root | Flower, pre-anthesis; leaf, fully expanded; root, produced by air-layering |
|  |  |  | CAB70005_IVaF_C09 | 30305095 | Cabernet Sauvignon | Berry | Post-Veraison, 18-19 brix |
|  |  |  | WIN0544.C21_E19 | 110383907 | Cabernet Sauvignon | Flower, leaf and root | Flower, pre-anthesis; leaf, fully expanded; root, produced by air-layering |
|  |  |  | CAB70005_IVaR_C09 | 30305157 | Cabernet Sauvignon | Berry | Post-Veraison, 18-19 brix |
|  |  |  | VVD130F10_372689 | 30126060 | Chardonnay | Berries | Mixed; 8, 9, 11, 13, 15, 16 weeks daf |
| *VvTAP2* | *VvABCB22* | GSVIVT01008121001 | CAP0002_IIIR_F12 | 34547772 | Cabernet Sauvignon | Petiole | Onset of Veraison (berry softening) |
|  |  |  | WIN0543.C21_C09 | 110383549 | Cabernet Sauvignon | Flower, leaf and root | Flower, pre-anthesis; leaf, fully expanded; root, produced by air-layering |
|  |  |  | VVD064H07_351705 | 30135198 | Chardonnay | Berries | Mixed; 8, 9, 11, 13, 15, 16 weeks daf |
|  |  |  | VVA019E07_391431 | 30320657 | Chardonnay | Leaf | Juvenile and adult |
|  |  |  | CAP0002_IIIF_F12 | 34547701 | Cabernet Sauvignon | Petiole | Onset of Veraison (berry softening) |
|  |  |  | CAB20007_Ia_Ra_B11 | 33405807 | Cabernet Sauvignon | Flower | Bloom |
|  |  |  | WIN074.C21_E11 | 110394417 | Cabernet Sauvignon | Pericarp | Fruit set to maturity |
|  |  |  | CAbud0007_IIIR_G03 | 34545722 | Cabernet Sauvignon | Bud | Pre-bloom (10-11 days before bloom) |
|  |  |  | CAB20007_IVa_Fa_C02 | 33405559 | Cabernet Sauvignon | Flower | Bloom |
|  |  |  | CAbud0007_IIIF_G03 | 34545637 | Cabernet Sauvignon | Bud | Pre-bloom (10-11 days before bloom) |
|  |  |  | CAB20007_IVa_Ra_C02 | 33405643 | Cabernet Sauvignon | Flower | Bloom |
|  |  |  | WIN027.TB24.1_M04 | 110361618 | Cabernet Sauvignon | Flower, leaf and root | Flower, pre-anthesis; leaf, fully expanded; root, produced by air-layering |
|  |  |  | FAMU_USDA_FP_1198 | 51575339 | Vitis shuttleworthii | Entire tendril, leaves, bud, flowers | At blooming |
|  |  |  | VVA019E07_54365 | 18459157 | Chardonnay | Leaf | Juvenile and adult |
|  |  |  | FAMU_USDA_FP_6959 | 51581100 | Vitis shuttleworthii | Entire tendril, leaves, bud, flowers | At blooming |
|  |  |  | CN547301 | 46917972 | Shiraz | Fruit without seeds | Veraison stage |
|  |  |  | S1G03330 | 110699209 | Thompson-seedless | Fruit and flower |  |
|  |  |  | sT7aVVM_AER44H10 | 161710848 | Cabernet Sauvignon | Roots | 10 cm high plants grown in Magenta boxes |
|  |  |  | sT7aVVM_AER12A11 | 161708365 | Cabernet Sauvignon | Roots | 10 cm high plants grown in Magenta boxes |
|  |  |  | sT7aVVM_AER11H11 | 161710662 | Cabernet Sauvignon | Roots | 10 cm high plants grown in Magenta boxes |
|  |  |  | WIN0533.C21_L16 | 110379857 | Cabernet Sauvignon | Flower, leaf and root | Flower, pre-anthesis; leaf, fully expanded; root, produced by air-layering |
|  |  |  | S6B02184 | 110712851 | Thompson-seedless | Fruit |  |
|  |  |  | CAB20007_Ia_Fa_B11 | 33405722 | Cabernet Sauvignon | Flower | Bloom |
|  |  |  | S8B01355 | 110718335 | Thompson-seedless | Fruit | Veraison |
|  |  |  | VVA019E07_54365 | 18459157 | Chardonnay | Leaf | Juvenile and adult |
|  |  |  | FAMU_USDA_FP_1198 | 51575339 | Vitis shuttleworthii | Entire tendril, leaves, bud, flowers | At blooming |
| *VvTAP3* | *VvABCB23* | GSVIVT01010634001 |  |  |  |  |  |
| *VvTAP4* | *VvABCB24* | GSVIVT01032404001 | WIN0551.C21_A01 | 122690312 | Cabernet Sauvignon | Flower, leaf and root | Flower, pre-anthesis; leaf, fully expanded; root, produced by air-layering |
|  |  |  | WIN1144.C21_F03 | 110425659 | Muscat Hamburg | Berry | Anthesis flower to prior to veraison |
|  |  |  | WIN0530.C21_D12 | 110378746 | Cabernet Sauvignon | Flower, leaf and root | Flower, pre-anthesis; leaf, fully expanded; root, produced by air-layering |
| *VvTAP5* | *VvABCB25* | GSVIVT01032898001 | VVD064H07_351705 | 30135198 | Chardonnay | Berries | Mixed; 8, 9, 11, 13, 15, 16 weeks daf |
|  |  |  | VVL086B07_689286 | 71884775 | Cabernet Sauvignon | Fruit with seeds removed | Mixed 36-38 - modified E-L system (Brix > 15) |
|  |  |  | VVA019E07_54365 | 18459157 | Chardonnay | Leaf | Juvenile and adult |
|  |  |  | WIN074.C21_E11 | 110394417 | Cabernet Sauvignon | Pericarp | Fruit set to maturity |
|  |  |  | S1G03330 | 110699209 | Thompson-seedless | Fruit and flower |  |
|  |  |  | sT7aVVM_AER44H10 | 161710848 | Cabernet Sauvignon | Roots | 10 cm high plants grown in Magenta boxes |
|  |  |  | FAMU_USDA_FP_1198 | 51575339 | Vitis shuttleworthii | Entire tendril, leaves, bud, flowers | At blooming |
|  |  |  | WIN027.TB24.1_H06 | 110361530 | Cabernet Sauvignon | Flower, leaf and root | Flower, pre-anthesis; leaf, fully expanded; root, produced by air-layering |
|  |  |  | sT7aVVM020K18069 | 161716179 | Cabernet Sauvignon | Roots | 10 cm high plants grown in Magenta boxes |
|  |  |  | WIN027.TB24.1_M04 | 110361618 | Cabernet Sauvignon | Flower, leaf and root | Flower, pre-anthesis; leaf, fully expanded; root, produced by air-layering |
|  |  |  | CAP0002_IIIF_F12 | 34547701 | Cabernet Sauvignon | Petiole | Onset of Veraison (berry softening) |
|  |  |  | CAP0002_IIIR_F12 | 34547772 | Cabernet Sauvignon | Petiole | Onset of Veraison (berry softening) |
|  |  |  | VVD064H07_351705 | 30135198 | Chardonnay | Berries | Mixed; 8, 9, 11, 13, 15, 16 weeks daf |
|  |  |  | VVL124C04_695916 | 71888090 | Cabernet Sauvignon | Fruit with seeds removed | Mixed 36-38 - modified E-L system (Brix > 15) |
|  |  |  | VVL125H06_696204 | 71888234 | Cabernet Sauvignon | Fruit with seeds removed | Mixed 36-38 - modified E-L system (Brix > 15) |
|  |  |  | VVL131F01_697168 | 71888716 | Cabernet Sauvignon | Fruit with seeds removed | Mixed 36-38 - modified E-L system (Brix > 15) |
|  |  |  | VVL086B07_689286 | 71884775 | Cabernet Sauvignon | Fruit with seeds removed | Mixed 36-38 - modified E-L system (Brix > 15) |
|  |  |  | VVL087H12_689602 | 71884933 | Cabernet Sauvignon | Fruit with seeds removed | Mixed 36-38 - modified E-L system (Brix > 15) |
|  |  |  | WIN027.TB24.1_M04 | 110361618 | Cabernet Sauvignon | Flower, leaf and root | Flower, pre-anthesis; leaf, fully expanded; root, produced by air-layering |
|  |  |  | CAP0002_IIIF_F12 | 34547701 | Cabernet Sauvignon | Petiole | Onset of Veraison (berry softening) |
|  |  |  | VVA019E07_54365 | 18459157 | Chardonnay | Leaf | Juvenile and adult |
|  |  |  | WIN0533.C21_L16 | 110379857 | Cabernet Sauvignon | Flower, leaf and root | Flower, pre-anthesis; leaf, fully expanded; root, produced by air-layering |
|  |  |  | sT7aVVM_AER11H11 | 161710662 | Cabernet Sauvignon | Roots | 10 cm high plants grown in Magenta boxes |
|  |  |  | sT7aVVM_AER12A11 | 161708365 | Cabernet Sauvignon | Roots | 10 cm high plants grown in Magenta boxes |
|  |  |  | FAMU_USDA_FP_1198 | 51575339 | Vitis shuttleworthii | Entire tendril, leaves, bud, flowers | At blooming |
