# Supplementary material for: Whole-Genome Survey of the Putative ATP-Binding Cassette Transporter Family Genes in Vitis vinifera
Source: PLoS One. 2013 Nov 11;8(11):e78860. doi: 10.1371/journal.pone.0078860 (PMC3823996; doi:10.1371/journal.pone.0078860)
Supplement: Table S6 — Expressed sequence taqs (ESTs) identified for ABCC (MRP) subfamily in Vitis vinifera . The protein name, Vitis proteome 12x ID, GenBank ID, EST name, cultivar/tissue type, and development stage are given for each gene. (DOC) [file pone.0078860.s006.doc]

**Table S6.** Expressed sequence taqs (ESTs) identified for ABCC (MRP) subfamily in *Vitis vinifera*.The protein name, *Vitis* proteome 12x ID, GenBank ID, EST name, cultivar/tissue type, and development stage are given for each gene.

| **Name** | | **12X ID** | **EST Name** | **GenBank ID** | **Species/Cultivar** | **Tissue Type** | **Development Stage** |
| --- | --- | --- | --- | --- | --- | --- | --- |
| *VvMRP1* | *VvABCC1* | GSVIVT01010326001 | VVB176A01_413457 | 32271616 | Chardonnay | Leaf | Juvenile and adult |
|  |  |  | VVB071E10_333456 | 30323902 | Chardonnay | Leaf | Juvenile and adult |
|  |  |  | VVB178D12_413897 | 32271836 | Chardonnay | Leaf | Juvenile and adult |
|  |  |  | S7B04488 | 110715223 | Thompson-seedless | Berry | Berries 14mm |
|  |  |  | VVI021F04_586288 | 71869169 | Cabernet Sauvignon | Inflorescence including flowers | 12 - modified E-L system |
|  |  |  | EST 15428 | 46918110 | Shiraz | Fruit without seeds | Veraison stage |
|  |  |  | CAB40003_Ia_Fa_H04 | 30300511 | Cabernet Sauvignon | Berry | Berry on stage II, 9 mm |
|  |  |  | FAMU_USDA_FP_6869 | 51581010 | Vitis shuttleworthii | Entire tendril, leaves, bud, flowers | At blooming |
| *VvMRP2* | *VvABCC2* | GSVIVT01014628001 | VV_PEa016e01.b1 | 156723994 | Perlette | Bud | Mature |
|  |  |  | VV_PEc03C05.r.ab1 | 156733370 | Perlette | Bud | Mature |
|  |  |  | VVC065F06_230914 | 27586561 | Chardonnay | Berries | Mixed; 8, 9, 11, 13, 15, 16 weeks daf |
|  |  |  | VVL004A01_675224 | 71877744 | Cabernet Sauvignon | Fruit with seed removed | Mixed 36-38 - modified E-L system (Brix > 15) |
|  |  |  | WIN0821.C21_M23 | 110400165 | Cabernet Sauvignon | Seed | Fruit set to maturity |
|  |  |  | VRJ387 | 39952408 | Vitis riparia | Bud | Dormant |
|  |  |  | VVB186B12_429463 | 32247279 | Chardonnay | Leaf | Juvenile and adult |
|  |  |  | RR890915N0006_IIa_Ra_H12 | 33397121 | Vitis hybrid cultivar | Leaf | 17-week old greenhouse grown plants |
|  |  |  | RR890915N0006_IIa_Fa_H12 | 33397075 | Vitis hybrid cultivar | Leaf | 17-week old greenhouse grown plants |
| *VvMRP3* | *VvABCC3* | GSVIVT01014629001 | VV_PEa16e01.g1 | 156726808 | Perlette | Bud | Mature |
|  |  |  | VV_PEa016e01.b1 | 156723994 | Perlette | Bud | Mature |
|  |  |  | VV_PEc03C05.f.ab1 | 156733369 | Perlette | Bud | Mature |
|  |  |  | VV_PEc03C05.r.ab1 | 156733370 | Perlette | Bud | Mature |
|  |  |  | RR890915N0006_IIa_Ra_H12 | 33397121 | Vitis hybrid cultivar | Leaf | 17-week old greenhouse grown plants |
|  |  |  | RR890915N0006_IIa_Fa_H12 | 33397075 | Vitis hybrid cultivar | Leaf | 17-week old greenhouse grown plants |
|  |  |  | VVL004A01_675224 | 71877744 | Cabernet Sauvignon | Fruit with seed removed | Mixed 36-38 - modified E-L system (Brix > 15) |
|  |  |  | VVL011A08_676432 | 71878348 | Cabernet Sauvignon | Fruit with seed removed | Mixed 36-38 - modified E-L system (Brix > 15) |
|  |  |  | VVC065F06_230914 | 27586561 | Chardonnay | Berries | Mixed; 8, 9, 11, 13, 15, 16 weeks daf |
|  |  |  | VVB186B12_429463 | 32247279 | Chardonnay | Berries | Mixed; 8, 9, 11, 13, 15, 16 weeks daf |
|  |  |  | WIN0821.C21_M23 | 110400165 | Cabernet Sauvignon | Seed | Fruit set to maturity |
|  |  |  | CAbud0005_IVF_H12 | 34544835 | Cabernet Sauvignon | Bud | Pre-bloom (10-11 days before bloom) |
|  |  |  | VRJ387 | 39952408 | Vitis riparia | Bud | Dormant |
| *VvMRP4* | *VvABCC4* | GSVIVT01014631001 | VV_PEa16e01.g1 | 156726808 | Perlette | Bud | Mature |
|  |  |  | VV_PEa016e01.b1 | 156723994 | Perlette | Bud | Mature |
|  |  |  | VV_PEc03C05.f.ab1 | 156733369 | Perlette | Bud | Mature |
|  |  |  | VV_PEc03C05.r.ab1 | 156733370 | Perlette | Bud | Mature |
|  |  |  | RR890915N0006_IIa_Ra_H12 | 33397121 | Vitis hybrid cultivar | Leaf | 17-week old greenhouse grown plants |
|  |  |  | RR890915N0006_IIa_Fa_H12 | 33397075 | Vitis hybrid cultivar | Leaf | 17-week old greenhouse grown plants |
|  |  |  | VVL004A01_675224 | 71877744 | Cabernet Sauvignon | Fruit with seed removed | Mixed 36-38 - modified E-L system (Brix > 15) |
|  |  |  | VVL011A08_676432 | 71878348 | Cabernet Sauvignon | Fruit with seed removed | Mixed 36-38 - modified E-L system (Brix > 15) |
|  |  |  | VVD106E08_369847 | 30130754 | Chardonnay | Berries | Mixed; 8, 9, 11, 13, 15, 16 weeks daf |
|  |  |  | WIN0821.C21_M23 | 110400165 | Cabernet Sauvignon | Seed | Fruit set to maturity |
| *VvMRP5* | *VvABCC5* | GSVIVT01014632001 | VV_PEa16e01.g1 | 156726808 | Perlette | Bud | Mature |
|  |  |  | VV_PEa016e01.b1 | 156723994 | Perlette | Bud | Mature |
|  |  |  | VV_PEc03C05.r.ab1 | 156733370 | Perlette | Bud | Mature |
|  |  |  | VV_PEc03C05.f.ab1 | 156733369 | Perlette | Bud | Mature |
|  |  |  | VVL004A01_675224 | 71877744 | Cabernet Sauvignon | Fruit with seed removed | Mixed 36-38 - modified E-L system (Brix > 15) |
|  |  |  | VVL011A08_676432 | 71878348 | Cabernet Sauvignon | Fruit with seed removed | Mixed 36-38 - modified E-L system (Brix > 15) |
|  |  |  | VVB186B12_429463 | 32247279 | Chardonnay | Leaf | Juvenile and adult |
|  |  |  | VVD106E08_369847 | 30130754 | Chardonnay | Berries | Mixed; 8, 9, 11, 13, 15, 16 weeks daf |
|  |  |  | RR890915N0006_IIa_Fa_H12 | 33397075 | Vitis hybrid cultivar | Leaf | 17-week old greenhouse grown plants |
|  |  |  | RR890915N0006_IIa_Ra_H12 | 33397121 | Vitis hybrid cultivar | Leaf | 17-week old greenhouse grown plants |
|  |  |  | WIN0821.C21_M23 | 110400165 | Cabernet Sauvignon | Seed | Fruit set to maturity |
|  |  |  | CAB40003_Ia_Fa_H04 | 30300511 | Cabernet Sauvignon | Berry | Berry on stage II, 9 mm |
|  |  |  | FAMU_USDA_FP_6869 | 51581010 | Vitis shuttleworthii | Entire tendril, leaves, bud, flowers | At blooming |
| *VvMRP6* | *VvABCC6* | GSVIVT01014633001 | VV_PEa016e01.b1 | 156723994 | Perlette | Bud | Mature |
|  |  |  | VV_PEc03C05.r.ab1 | 156733370 | Perlette | Bud | Mature |
|  |  |  | VV_PEc03C05.f.ab1 | 156733369 | Perlette | Bud | Mature |
|  |  |  | WIN0821.C21_M23 | 110400165 | Cabernet Sauvignon | Seed | Fruit set to maturity |
|  |  |  | RR890915N0006_IIa_Fa_H12 | 33397075 | Vitis hybrid cultivar | Leaf | 17-week old greenhouse grown plants |
|  |  |  | RR890915N0006_IIa_Ra_H12 | 33397121 | Vitis hybrid cultivar | Leaf | 17-week old greenhouse grown plants |
|  |  |  | VVB186B12_429463 | 32247279 | Chardonnay | Leaf | Juvenile and adult |
|  |  |  | VVD106E08_369847 | 30130754 | Chardonnay | Berries | Mixed; 8, 9, 11, 13, 15, 16 weeks daf |
|  |  |  | VVL004A01_675224 | 71877744 | Cabernet Sauvignon | Fruit with seed removed | Mixed 36-38 - modified E-L system (Brix > 15) |
|  |  |  | VVL011A08_676432 | 71878348 | Cabernet Sauvignon | Fruit with seed removed | Mixed 36-38 - modified E-L system (Brix > 15) |
| *VvMRP7* | *VvABCC7* | GSVIVT01015960001 |  |  |  |  |  |
| *VvMPR8* | *VvABCC8* | GSVIVT01016879001 | GEMMA01_000344 | 37187581 | Pinot Noir | Bud | Bud swelling |
|  |  |  | WIN105.C21_C10 | 110411874 | Muscat Hamburg | Pericarp | Fruit set to maturity |
|  |  |  | CA12LIO2IIIF_C07 | 26257141 | Cabernet Sauvignon | Leaf | Late season sample |
|  |  |  | CA12LIO2IIIR_C07 | 26257978 | Cabernet Sauvignon | Leaf | Late season sample |
|  |  |  | WIN105.C21_H21 | 110411984 | Muscat Hamburg | Pericarp | Fruit set to maturity |
|  |  |  | CAB10001_IIIa_Ra_H03 | 30251230 | Cabernet Sauvignon | Flower | Pre-bloom |
|  |  |  | CA32EN0002_IIaR_H07 | 29785201 | Cabernet Sauvignon | Leaf | Mid-season leaf material, collected July 25, 2001 |
|  |  |  | EST 4925 | 22010953 | Shiraz | Fruit | Ripening stage |
|  |  |  | INFIO01_000633 | 37190464 | Regent | Inflorescence | young inflorescence before flowering |
|  |  |  | BQ796635 | 110362756 | Cabernet Sauvignon | Fruit | Ripening stage |
|  |  |  | WIN0211.TB24_N14 | 110362756 | Cabernet Sauvignon | Flower, leaf and root | Flower, pre-anthesis; leaf, fully expanded; root, produced by air-layering |
|  |  |  | WIN0821.C21_B11 | 110399935 | Cabernet Sauvignon | Seed | Fruit set to maturity |
|  |  |  | C4B02624 | 110696847 | Carmenere | Cluster | Veraison |
|  |  |  | WIN052.C21_C11 | 110381535 | Cabernet Sauvignon | Flower, leaf and root | Flower, pre-anthesis; leaf, fully expanded; root, produced by air-layering |
|  |  |  | CAB70003_IIIaR_G05 | 30303963 | Cabernet Sauvignon | Berry | Post-Veraison, 18-19 brix |
|  |  |  | C4B03505 | 110695449 | Carmenere | Cluster | Veraison |
|  |  |  | WIN013.BR_P21 | 110359216 | Cabernet Sauvignon | Pericarp | Fruit set to maturity |
|  |  |  | CAB40003_IIa_Fa_E07 | 30300210 | Cabernet Sauvignon | Berry | Berry on stage II, 9 mm |
|  |  |  | sT7aVVM012E12044 | 161716554 | Cabernet Sauvignon | Roots | 10 cm high plants grown in Magenta boxes |
|  |  |  | WIN086.C21_E16 | 110402909 | Cabernet Sauvignon | Seed | Fruit set to maturity |
|  |  |  | WIN0810.C21_G18 | 110395380 | Cabernet Sauvignon | Seed | Fruit set to maturity |
|  |  |  | GEMMA01_000344 | 37187581 | Pinot Noir | Bud | Bud swelling |
|  |  |  | WIN105.C21_C10 | 110411874 | Muscat Hamburg | Pericarp | Fruit set to maturity |
|  |  |  | CA12LIO2IIIF_C07 | 26257141 | Cabernet Sauvignon | Leaf | Late season sample |
|  |  |  | CA12LIO2IIIR_C07 | 26257978 | Cabernet Sauvignon | Leaf | Late season sample |
|  |  |  | WIN105.C21_H21 | 110411984 | Muscat Hamburg | Pericarp | Fruit set to maturity |
|  |  |  | VVB074H04_335992 | 30325170 | Chardonnay | Leaf | Juvenile and adult |
|  |  |  | VVB064B11_326946 | 30322934 | Chardonnay | Leaf | Juvenile and adult |
|  |  |  | EST 4925 | 22010953 | Shiraz | Fruit | Ripening stage |
|  |  |  | VVB083D09_335402 | 30324875 | Chardonnay | Leaf | Juvenile and adult |
|  |  |  | VVB137D03_406435 | 32268105 | Chardonnay | Leaf | Juvenile and adult |
|  |  |  | VVB100G08_339799 | 30325878 | Chardonnay | Leaf | Juvenile and adult |
|  |  |  | VVB137D03_406435 | 32268105 | Chardonnay | Leaf | Juvenile and adult |
|  |  |  | EST 5574 | 22011602 | Shiraz | Fruit | Ripening stage |
|  |  |  | CAB10001_IIIa_Ra_H03 | 30251230 | Cabernet Sauvignon | Flower | Pre-bloom |
|  |  |  | CA32EN0002_IIaR_H07 | 29785201 | Cabernet Sauvignon | Leaf | Mid-season leaf material, collected July 25, 2001 |
|  |  |  | INFIO01_000633 | 37190464 | Regent | Inflorescence | young inflorescence before flowering |
|  |  |  | CSECS060B02_VERu0035 | 34364268 | Cabernet Sauvignon | Fruit with seeds remove | 35 - modified E-L system |
|  |  |  | WIN0821.C21_B11 | 110399935 | Cabernet Sauvignon | Seed | Fruit set to maturity |
|  |  |  | C4B02624 | 110696847 | Carmenere | Cluster | Veraison |
|  |  |  | S7B04488 | 110715223 | Thompson-seedless | Berry |  |
|  |  |  | SCB03932 | 110732027 | Thompson-seedless | Inflorescence |  |
|  |  |  | WIN052.C21_C11 | 110381535 | Cabernet Sauvignon | Flower, leaf and root | Flower, pre-anthesis; leaf, fully expanded; root, produced by air-layering |
|  |  |  | CAB70003_IIIaF_G05 | 30303898 | Cabernet Sauvignon | Berry | Post-Veraison, 18-19 brix |
|  |  |  | CAB70003_IIIaR_G05 | 30303963 | Cabernet Sauvignon | Berry | Post-Veraison, 18-19 brix |
|  |  |  | C4B03505 | 110695449 | Carmenere | Cluster | Verasion |
|  |  |  | WIN061.C21_A03 | 110391856 | Cabernet Sauvignon | Seed | Fruit set to maturity |
|  |  |  | WIN013.BR_P21 | 110359216 | Cabernet Sauvignon | Pericarp | Fruit set to maturity |
|  |  |  | WIN048.C21_J18 | 110370621 | Cabernet Sauvignon | Pericarp | Fruit set to maturity |
|  |  |  | CAB40003_IIa_Ra_E07 | 30300267 | Cabernet Sauvignon | Berry | Berry on stage II, 9 mm |
|  |  |  | CAB40003_IIa_Fa_E07 | 30300210 | Cabernet Sauvignon | Berry | Berry on stage II, 9 mm |
|  |  |  | FAMU_USDA_FP_5379 | 51579520 | Vitis shuttleworthii | Entire tendril, leaves, bud, flowers | At blooming |
|  |  |  | VVB185D05_415319 | 32272547 | Chardonnay | Leaf | Juvenile and adult |
|  |  |  | C4B00786 | 110694968 | Carmenere | Cluster | Veraison |
|  |  |  | VVB160G11_410715 | 32270245 | Chardonnay | Leaf | Juvenile and adult |
|  |  |  | VVC036D03_139486 | 27583963 | Chardonnay | Berries | Mixed; 8, 9, 11, 13, 15, 16 weeks daf |
|  |  |  | CA32EN0002_IIaF_H07 | 29785172 | Cabernet Sauvignon | Leaf | Mid-season leaf material, collected July 25, 2001 |
|  |  |  | VVC036D03_395149 | 30329508 | Chardonnay | Berries | Mixed; 8, 9, 11, 13, 15, 16 weeks daf |
|  |  |  | VVB186A06_429427 | 32247261 | Chardonnay | Leaf | Juvenile and adult |
|  |  |  | CAB10001_IIIa_Fa_H03 | 30251150 | Cabernet Sauvignon | Flower | Pre-bloom |
|  |  |  | EST 5573 | 22011601 | Shiraz | Fruit | Ripening stage |
|  |  |  | FAMU_USDA_FP_1175 | 51575316 | Vitis shuttleworthii | Entire tendril, leaves, bud, flowers | At blooming |
|  |  |  | WIN0211.TB24_N14 | 110362756 | Cabernet Sauvignon | Flower, leaf and root | Flower, pre-anthesis; leaf, fully expanded; root, produced by air-layering |
|  |  |  | WIN088.C21_A08 | 110398184 | Cabernet Sauvignon | Seed | Fruit set to maturity |
|  |  |  | EST 7160 | 22015091 | Ugni Blanc | Fruit | Veraison stage |
| *VvMRP9* | *VvABCC9* | GSVIVT01016880001 | sT7aVVM012E12044 | 161716554 | Cabernet Sauvignon | Roots | 10 cm high plants grown in Magenta boxes |
|  |  |  | GEMMA01_000344 | 37187581 | Pinot Noir | Bud | Bud swelling |
|  |  |  | CA12LIO2IIIR_C07 | 26257978 | Cabernet Sauvignon | Leaf | Leaf season sample |
|  |  |  | WIN105.C21_H21 | 110411984 | Muscat Hamburg | Pericarp | Fruit set to maturity |
|  |  |  | VVB074H04_335992 | 30325170 | Chardonnay | Leaf | Juvenile and adult |
|  |  |  | VVB064B11_326946 | 30322934 | Chardonnay | Leaf | Juvenile and adult |
|  |  |  | EST 4925 | 22010953 | Shiraz | Fruit | Ripening stage |
|  |  |  | VVB083D09_335402 | 30324875 | Chardonnay | Leaf | Juvenile and adult |
|  |  |  | VVB137D03_406435 | 32268105 | Chardonnay | Leaf | Juvenile and adult |
|  |  |  | VVB100G08_339799 | 30325878 | Chardonnay | Leaf | Juvenile and adult |
|  |  |  | VVB185D05_415319 | 32272547 | Chardonnay | Leaf | Juvenile and adult |
|  |  |  | VVB160G11_410715 | 32270245 | Chardonnay | Leaf | Juvenile and adult |
|  |  |  | VVB154C08_409509 | 32269642 | Chardonnay | Leaf | Juvenile and adult |
|  |  |  | VVB153A08_409277 | 32269526 | Chardonnay | Leaf | Juvenile and adult |
|  |  |  | VVB172C11_412815 | 32271295 | Chardonnay | Leaf | Juvenile and adult |
|  |  |  | VVB186A06_429427 | 32247261 | Chardonnay | Leaf | Juvenile and adult |
|  |  |  | CAB10001_IIIa_Ra_H03 | 30251230 | Cabernet Sauvignon | Flower | Pre-bloom |
|  |  |  | CA32EN0002_IIaR_H07 | 29785201 | Cabernet Sauvignon | Leaf | Mid-season leaf material, collected July 25, 2001 |
|  |  |  | VVB142E11_407395 | 32268585 | Chardonnay | Leaf | Juvenile and adult |
|  |  |  | INFIO01_000633 | 37190464 | Regent | Inflorescence | young inflorescence before flowering |
|  |  |  | WIN0821.C21_B11 | 110399935 | Cabernet Sauvignon | seed | Fruit set to maturity |
|  |  |  | C4B02624 | 110696847 | Carmenere | Cluster | Veraison |
|  |  |  | S7B04488 | 110715223 | Thompson-seedless | Berry |  |
|  |  |  | SCB03932 | 110732027 | Thompson-seedless | Inflorescence |  |
|  |  |  | WIN052.C21_C11 | 110381535 | Cabernet Sauvignon | Flower leaf and root | Flower, pre-anthesis; leaf, fully expanded; root, produced by air-layering |
|  |  |  | CAB70003_IIIaF_G05 | 30303898 | Cabernet Sauvignon | Berry | Post-Veraison, 18-19 brix |
|  |  |  | CAB70003_IIIaR_G05 | 30303963 | Cabernet Sauvignon | Berry | Post-Veraison, 18-19 brix |
|  |  |  | C4B03505 | 110695449 | Carmenere | Cluster | Veraison |
|  |  |  | WIN061.C21_A03 | 110391856 | Cabernet Sauvignon | Seed | Fruit set to maturity |
|  |  |  | CAB40003_IIa_Fa_E07 | 30300210 | Cabernet Sauvignon | Berry | Berry on stage II, 9 mm |
|  |  |  | CAB40003_IIa_Ra_E07 | 30300267 | Cabernet Sauvignon | Berry | Berry on stage II, 9 mm |
|  |  |  | FAMU_USDA_FP_5379 | 51579520 | Vitis shuttleworthii | Entire tendril, leaves, bud, flowers | At blooming |
|  |  |  | VVB146E06_408125 | 32268950 | Chardonnay | Leaf | Juvenile and adult |
|  |  |  | CA32EN0002_IIaF_H07 | 29785172 | Cabernet Sauvignon | Leaf | Mid-season leaf material, collected July 25, 2001 |
|  |  |  | VVB173H01_413091 | 32271433 | Chardonnay | Leaf | Juvenile and adult |
|  |  |  | C4B00786 | 110694968 | Carmenere | Cluster | Veraison |
|  |  |  | EST 5574 | 22011602 | Shiraz | Fruit | Ripening stage |
|  |  |  | CAB10001_IIIa_Fa_H03 | 30251150 | Cabernet Sauvignon | Flower | Pre-bloom |
|  |  |  | EST 5573 | 22011601 | Shiraz | Fruit | Ripening stage |
|  |  |  | FAMU_USDA_FP_1175 | 51575316 | Vitis shuttleworthii | Entire tendril, leaves, bud, flowers | At blooming |
|  |  |  | WIN0211.TB24_N14 | 110362756 | Cabernet Sauvignon | Flower, leaf and root | Flower, pre-anthesis; leaf, fully expanded; root, produced by air-layering |
| *VvMRP10* | *VvABCC10* | GSVIVT01018403001 | EST 15428 | 46918110 | Shiraz | Fruit without seed | Veraison stage |
|  |  |  | VVC030G04_138530 | 27583485 | Chardonnay | Berries | Mixed; 8, 9, 11, 13, 15, 16 weeks daf |
|  |  |  | CAB40003_Ia_Fa_H04 | 30300511 | Cabernet Sauvignon | Berry | Berry on stage II, 9 mm |
|  |  |  | WIN045.C21_F06 | 110370213 | Cabernet Sauvignon | Pericarp | Fruit set to maturity |
|  |  |  | S8B04238 | 110720413 | Thompson-seedless | Fruit | Veraison |
|  |  |  | EST 18090 | 46910763 | Cabernet Sauvignon | Fruit skin | Ripening stage |
|  |  |  | FAMU_USDA_FP_1781 | 51575922 | Vitis shuttleworthii | Entire tendril, leaves, bud, flowers | At bloomng |
|  |  |  | EST 12462 | 32456084 | Ugni Blanc | Fruit without seed | Veraison stage |
|  |  |  | VVC065F06_230914 | 27586561 | Chardonnay | Berries | Mixed; 8, 9, 11, 13, 15, 16 weeks daf |
|  |  |  | VVD101E01_368965 | 30130313 | Chardonnay | Berries | Mixed; 8, 9, 11, 13, 15, 16 weeks daf |
|  |  |  | EST 231 | 14580405 | Shiraz | Fruit | Veraison stage |
|  |  |  | S7B03890 | 110714877 | Thompson-seedless | Berry |  |
|  |  |  | sT7aVVM_AER77C09 | 161710658 | Cabernet Sauvignon | Roots | 10 cm high plants grown in Magenta boxes |
|  |  |  | VVTOV303 | 160482014 | Thompson-seedless | Ovule | Mixed stages 27-48d after bloom |
|  |  |  | EST 18155 | 46910828 | Cabernet Sauvignon | Fruit skin | Ripening stage |
|  |  |  | CAB40003_Ia_Ra_H04 | 30300584 | Cabernet Sauvignon | Berry | Berry on stage II, 9 mm |
|  |  |  | VVC050D04_147218 | 27585184 | Chardonnay | Berries | Mixed; 8, 9, 11, 13, 15, 16 weeks daf |
|  |  |  | VVD015A02_344843 | 30131766 | Chardonnay | Berries | Mixed; 8, 9, 11, 13, 15, 16 weeks daf |
|  |  |  | WIN1132.C21_C03 | 110421614 | Muscat Hamburg | Berry | Anthesis flower to prior to veraison |
|  |  |  | VVC005D12_119248 | 27582081 | Chardonnay | Berries | Mixed; 8, 9, 11, 13, 15, 16 weeks daf |
|  |  |  | VVC065F06_416223 | 32245759 | Chardonnay | Berries | Mixed; 8, 9, 11, 13, 15, 16 weeks daf |
|  |  |  | S1G02594 | 110699098 | Thompson-seedless | Fruit and flower |  |
|  |  |  | FAMU_USDA_FP_7591 | 51581732 | Vitis shuttleworthii | Entire tendril, leaves, bud, flowers | At blooming |
| *VvMRP11* | *VvABCC11* | GSVIVT01019467001 | EST 11714 | 32457594 | Chardonnay | Fruit pedicle | Ripe stage |
|  |  |  | S1G05217 | 110699494 | Thompson-seedless | Fruit and flower |  |
|  |  |  | WIN0548.C21_D14 | 110385427 | Cabernet Sauvignon | Flower, leaf and root | Flower, pre-anthesis; leaf, fully expanded; root, produced by air-layering |
|  |  |  | VVH051A09_747293 | 71860768 | Cabernet Sauvignon | Nectary of flowers | 25 - modified E-L system |
|  |  |  | sT7aVVM_AER20D06 | 161706059 | Cabernet Sauvignon | Roots | 10 cm high plants grown in Magenta boxes |
|  |  |  | S6B03013 | 110713048 | Thompson-seedless | Fruit | Fruits 7-9 mm |
| *VvMRP12* | *VvABCC12* | GSVIVT01019469001 | sT7aVVM_AER20D06 | 161706059 | Cabernet Sauvignon | Roots | 10 cm high plants grown in Magenta boxes |
|  |  |  | sT7aVVM_AER28F09 | 161711103 | Cabernet Sauvignon | Roots | 10 cm high plants grown in Magenta boxes |
|  |  |  | sT7aVVM_AER28F08 | 161708945 | Cabernet Sauvignon | Roots | 10 cm high plants grown in Magenta boxes |
|  |  |  | VVL154B12_701102 | 77589227 | Cabernet Sauvignon | Fruit with seeds remove | Mixed 36-38 - modified E-L system (Brix > 15) |
|  |  |  | VVL151G10_700688 | 77588858 | Cabernet Sauvignon | Fruit with seeds remove | Mixed 36-38 - modified E-L system (Brix > 15) |
|  |  |  | WIN0548.C21_J12 | 122690262 | Cabernet Sauvignon | Flower leaf and root | Flower, pre-anthesis; leaf, fully expanded; root, produced by air-layering |
|  |  |  | EST 11714 | 32457594 | Chardonnay | Fruit pedicle | Ripe stage |
|  |  |  | WIN013.BR_P21 | 110359216 | Chardonnay | Pericarp | Fruit set to maturity |
|  |  |  | S1G05217 | 110699494 | Thompson-seedless | Fruit and flower |  |
|  |  |  | BACCA01_000742 | 37184105 | Pinot Noir | Berrry | Veraison |
|  |  |  | VVH051A09_747293 | 71860768 | Cabernet Sauvignon | Nectary of flowers | 25 - modified E-L system |
| *VvMRP13* | *VvABCC13* | GSVIVT01019471001 | BACCA01_000742 | 37184105 | Pinot Noir | Berrry | Veraison |
|  |  |  | S6B05345 | 110713613 | Thompson-seedless | Fruit | Fruits 7-9 mm |
|  |  |  | S1G05217 | 110699494 | Thompson-seedless | Fruit and flower |  |
|  |  |  | EST 11714 | 32457594 | Chardonnay | Fruit pedicle | Ripe stage |
|  |  |  | WIN022.C21_D02 | 110360506 | Cabernet Sauvignon | Flower, leaf and root | Flower, pre-anthesis; leaf, fully expanded; root, produced by air-layering |
|  |  |  | WIN1124.C21_N21 | 110419234 | Muscat Hamburg | Berry | Anthesis flower to prior to veraison |
|  |  |  | FAMU_USDA_FP_5379 | 51579520 | Vitis shuttleworthii | Entire tendril, leaves, bud, flowers | At blooming |
|  |  |  | VVH051A09_747293 | 71860768 | Cabernet Sauvignon | Nectary of flowers | 25 - modified E-L system |
|  |  |  | sT7aVVM_AER28F08 | 161708945 | Cabernet Sauvignon | Roots | 10 cm high plants grown in Magenta boxes |
|  |  |  | VVL154B12_701102 | 77589227 | Cabernet Sauvignon | Fruit with seed removed | Mixed 36-38 - modified E-L system (Brix > 15) |
|  |  |  | VVL151G10_700688 | 77588858 | Cabernet Sauvignon | Fruit with seeds remove | Mixed 36-38 - modified E-L system (Brix > 15) |
|  |  |  | sT7aVVM_AER28F09 | 161711103 | Cabernet Sauvignon | Roots | 10 cm high plants grown in Magenta boxes |
|  |  |  | sT7aVVM_AER20D06 | 161706059 | Cabernet Sauvignon | Roots | 10 cm high plants grown in Magenta boxes |
| *VvMRP14* | *VvABCC14* | GSVIVT01019473001 | INFIO01_000458 | 37190300 | Regent | inflorescence | young inflorescence before flowering |
|  |  |  | WIN022.C21_D02 | 110360506 | Cabernet Sauvignon | Flower, leaf and root | Flower, pre-anthesis; leaf, fully expanded; root, produced by air-layering |
|  |  |  | WIN1124.C21_N21 | 110419234 | Muscat Hamburg | Berry | Anthesis flower to prior to veraison |
|  |  |  | WIN0548.C21_D14 | 110385427 | Cabernet Sauvignon | Flower, leaf and root | Flower, pre-anthesis; leaf, fully expanded; root, produced by air-layering |
|  |  |  | BACCA01_000742 | 37184105 | Pinot Noir | Berry | Verasion |
|  |  |  | WIN013.BR_P21 | 110359216 | Cabernet Sauvignon | Pericarp | Fruit set to maturity |
|  |  |  | CAB40007_IIa_Fa_E08 | 30302459 | Cabernet Sauvignon | Berry | Berry on stage II, 9 mm |
|  |  |  | WIN0548.C21_J12 | 122690262 | Cabernet Sauvignon | Flower, leaf and root | Flower, pre-anthesis; leaf, fully expanded; root, produced by air-layering |
|  |  |  | EST0088 | 10283302 | Pinot Noir | Fruit | Berries harvested at veraison (onset of ripening) |
|  |  |  | sT7aVVM_AER28F09 | 161711103 | Cabernet Sauvignon | Roots | 10 cm high plants grown in Magenta boxes |
|  |  |  | VVL154B12_701102 | 77589227 | Cabernet Sauvignon | Fruit with seeds removed | Mixed 36-38 - modified E-L system (Brix > 15) |
|  |  |  | WIN1120.C21_G20 | 110417867 | Muscat Hamburg | Berry | Anthesis flower to prior to veraison |
|  |  |  | VVL151G10_700688 | 77588858 | Cabernet Sauvignon | Fruit with seeds removed | Mixed 36-38 - modified E-L system (Brix > 15) |
|  |  |  | sT7aVVM_AER28F08 | 161708945 | Cabernet Sauvignon | Roots | 10 cm high plants grown in Magenta boxes |
|  |  |  | sT7aVVM_AER20D06 | 161706059 | Cabernet Sauvignon | Roots | 10 cm high plants grown in Magenta boxes |
|  |  |  | VVH051A09_747293 | 71860768 | Cabernet Sauvignon | Nectary of flowers | 25 - modified E-L system |
| *VvMRP15* | *VvABCC15* | GSVIVT01019475001 | S6B05345 | 110713613 | Thompson-seedless | Fruit | Fruits 7-9 mm |
|  |  |  | S1G05217 | 110699494 | Thompson-seedless | Fruit and Flower | Fruits 7-9 mm |
|  |  |  | WIN0548.C21_D14 | 110385427 | Cabernet Sauvignon | Flower, leaf and root | Flower, pre-anthesis; leaf, fully expanded; root, produced by air-layering |
|  |  |  | EST 7595 | 22015526 | Ugni Blanc | Fruit | Veraison stage |
|  |  |  | sT7aVVM_AER28F08 | 161708945 | Cabernet Sauvignon | Roots | 10 cm high plants grown in Magenta boxes |
|  |  |  | VVL154B12_701102 | 77589227 | Cabernet Sauvignon | Fruit with seed removed | mixed 36-38 - modified E-L system (Brix > 15) |
|  |  |  | VVL151G10_700688 | 77588858 | Cabernet Sauvignon | Fruit with seed removed | mixed 36-38 - modified E-L system (Brix > 15) |
|  |  |  | sT7aVVM_AER28F09 | 161711103 | Cabernet Sauvignon | Roots | 10 cm high plants grown in Magenta boxes |
|  |  |  | sT7aVVM_AER20D06 | 161706059 | Cabernet Sauvignon | Roots | 10 cm high plants grown in Magenta boxes |
|  |  |  | WIN0548.C21_J12 | 122690262 | Cabernet Sauvignon | Flower, leaf and root | Flower, pre-anthesis; leaf, fully expanded; root, produced by air-layering |
|  |  |  | EST0088 | 10283302 | Pinot Noir | Fruit | Berries harvested at veraison (onset of ripening) |
|  |  |  | FAMU_USDA_FP_5379 | 51579520 | Vitis shuttleworthii | Entire tendril, leaves, bud, flowers | At blooming |
|  |  |  | EST 11714 | 32457594 | Chardonnay | Fruit pedicle | Ripe stage |
| *VvMRP16* | *VvABCC16* | GSVIVT01019476001 | S6B05345 | 110713613 | Thompson-seedless | Fruit | Fruits 7-9 mm |
|  |  |  | WIN0548.C21_J12 | 122690262 | Cabernet Sauvignon | Flower, leaf and root | Flower, pre-anthesis; leaf, fully expanded; root, produced by air-layering |
|  |  |  | SBB02819 | 110726701 | Thompson-seedless | Inflorescence |  |
|  |  |  | sT7aVVM_AER28F09 | 161711103 | Cabernet Sauvignon | Roots | 10 cm high plants grown in Magenta boxes |
|  |  |  | VVL154B12_701102 | 77589227 | Cabernet Sauvignon | Fruit with seeds removed | mixed 36-38 - modified E-L system (Brix > 15) |
|  |  |  | S1G05217 | 110699494 | Thompson-seedless | Fruit and Flower |  |
|  |  |  | WIN0548.C21_D14 | 110385427 | Cabernet Sauvignon | Flower, leaf and root | Flower, pre-anthesis; leaf, fully expanded; root, produced by air-layering |
|  |  |  | EST 7595 | 22015526 | Ugni Blanc | Fruit | Veraison stage |
|  |  |  | BACCA01_000742 | 37184105 | Pinot Noir | berry | veraison |
|  |  |  | EST 11714 | 32457594 | Chardonnay | Fruit pedicle | Ripe stage |
|  |  |  | FAMU_USDA_FP_5379 | 51579520 | Vitis shuttleworthii | Entire tendril, leaves, bud, flowers | At blooming |
|  |  |  | VVH051A09_747293 | 71860768 | Cabernet Sauvignon | Nectary of flowers | 25 - modified E-L system |
|  |  |  | sT7aVVM_AER28F08 | 161708945 | Cabernet Sauvignon | Roots | 10 cm high plants grown in Magenta boxes |
|  |  |  | VVL151G10_700688 | 77588858 | Cabernet Sauvignon | Fruit with seeds removed | mixed 36-38 - modified E-L system (Brix > 15) |
| *VvMRP17* | *VvABCC17* | GSVIVT01021589001 | FAMU_USDA_FP_2923 | 51577064 | Vitis shuttleworthii | Entire tendril, leaves, bud, flowers | At blooming |
|  |  |  | CAbud0007_IVF_D06 | 34545775 | Cabernet Sauvignon | Bud | Pre-bloom (10-11 days before bloom) |
|  |  |  | sT7aVVM024N06019 | 161715498 | Cabernet Sauvignon | Roots | 10 cm high plants grown in Magenta boxes |
|  |  |  | CAB70003_IIaF_H06 | 30304042 | Cabernet Sauvignon | Berry | Post-Veraison, 18-19 brix |
|  |  |  | CAP0006_IIF_H07 | 34549912 | Cabernet Sauvignon | Petiole | Onset of Veraison (berry softening) |
|  |  |  | Fag-B-IES1,5h-K1-95 | 118498742 | Vitis riparia | Root tip tissue | Adult plants |
|  |  |  | CAP0006_IIF_H07 | 34549912 | Cabernet Sauvignon | Petiole | Onset of Veraison (berry softening) |
|  |  |  | EST 18067 | 46910740 | Cabernet Sauvignon | Fruit skin | Ripening stage |
|  |  |  | VVL057A05_684352 | 71882308 | Cabernet Sauvignon | Fruit with seed removed | mixed 36-38 -modified E-L system (Brix > 15) |
|  |  |  | CA22LI02IVF-H7 | 26259023 | Cabernet Sauvignon | Leaf | Late season sample |
|  |  |  | EST 11769 | 32457649 | Chardonnay | Fruit pedicle | Ripening stage |
|  |  |  | CA23EI03IVFb_B10 | 28958677 | Cabernet Sauvignon | Leaf | Mid-season leaf material, collected July 25, 2001 |
|  |  |  | CA12EI203IIF_A05 | 26264206 | Cabernet Sauvignon | Leaf | Mid-season leaf material |
|  |  |  | CA23EI03IVRb_B10 | 28958514 | Cabernet Sauvignon | Leaf | Mid-season leaf material, collected July 25, 2001 |
|  |  |  | VVB187F06_429727 | 32247411 | Chardonnay | Leaf | Juvenile and adult |
|  |  |  | VVL127C08_696432 | 71888348 | Cabernet Sauvignon | Fruit with seeds removed | mixed 36-38 - modified E-L system (Brix > 15) |
|  |  |  | CAB10006_IVa_Fa_D03 | 30256268 | Cabernet Sauvignon | Flower | Pre-bloom |
|  |  |  | VVA019B03_402177 | 32245005 | Cabernet Sauvignon | Leaf | Juvenile and adult |
|  |  |  | WIN053.C21_P24 | 122689215 | Cabernet Sauvignon | Flower leaf and root | Flower, pre-anthesis; leaf, fully expanded; root, produced by air-layering |
|  |  |  | EE072120 | 110692642 | Carmenere | Cluster | Clusters 4 cm |
|  |  |  | sT7aVVM002B07032 | 161712466 | Cabernet Sauvignon | Roots | 10 cm high plants grown in Magenta boxes |
|  |  |  | CA32EN0004_IIaR_H10 | 29785770 | Cabernet Sauvignon | Leaf | Mid-season leaf material, collected July 25, 2001 |
|  |  |  | CA32EN0004_IVaR_H10 | 28964140 | Cabernet Sauvignon | Leaf | Mid-season leaf material, collected July 25, 2001 |
|  |  |  | CA32EN0004_IaR_H10 | 28964293 | Cabernet Sauvignon | Leaf | Mid-season leaf material, collected July 25, 2001 |
|  |  |  | CA32EN0004_IaF_H10 | 29785910 | Cabernet Sauvignon | Leaf | Mid-season leaf material, collected July 25, 2001 |
|  |  |  | WIN0562.C21_A22 | 110389690 | Cabernet Sauvignon | Flower leaf and root | Flower, pre-anthesis; leaf, fully expanded; root, produced by air-layering |
|  |  |  | VV_PEb21b08.g1 | 156731464 | Perlette | Bud | Mature |
|  |  |  | CSECS202E08_5_PREn0028 | 87584851 | Cabernet Sauvignon | Fruit | 28 - modified E-L system |
|  |  |  | BACCA01_001132 | 37184469 | Pinot Noir | Berry | Veraison |
|  |  |  | WIN1110.C21_C14 | 110414716 | Muscat Hamburg | Berry | Anthesis flower to prior to veraison |
|  |  |  | WIN051.C21_B23 | 110381415 | Cabernet Sauvignon | Flower leaf and root | Flower, pre-anthesis; leaf, fully expanded; root, produced by air-layering |
|  |  |  | WIN0544.C21_C22 | 110383867 | Cabernet Sauvignon | Flower leaf and root | Flower, pre-anthesis; leaf, fully expanded; root, produced by air-layering |
|  |  |  | WIN0518.C21_M04 | 110375179 | Cabernet Sauvignon | Flower leaf and root | Flower, pre-anthesis; leaf, fully expanded; root, produced by air-layering |
| *VvMRP18* | *VvABCC18* | GSVIVT01021593001 | FAMU_USDA_FP_2923 | 51577064 | Vitis shuttleworthii | Entire tendril, leaves, bud, flowers | At blooming |
|  |  |  | CAP0006_IIF_H07 | 34549912 | Cabernet Sauvignon | Petiole | Onset of Veraison (berry softening) |
| *VvMRP19* | *VvABC19* | GSVIVT01021594001 | CAP0006_IIF_H07 | 34549912 | Cabernet Sauvignon | Petiole | Onset of Veraison (berry softening) |
|  |  |  | sT7aVVM024N06019 | 161715498 | Cabernet Sauvignon | Roots | 10 cm high plants grown in Magenta boxes |
|  |  |  | S6B03611 | 110711013 | Thompson-seedless | Fruit | Fruits 7-9 mm |
|  |  |  | WIN053.C21_P24 | 122689215 | Cabernet Sauvignon | Flower leaf and root | Flower, pre-anthesis; leaf, fully expanded; root, produced by air-layering |
|  |  |  | WIN056.C21_D12 | 110373883 | Cabernet Sauvignon | Flower leaf and root | Flower, pre-anthesis; leaf, fully expanded; root, produced by air-layering |
|  |  |  | VV_PEa10h09.b1 | 156725510 | Perlette | Bud | Mature |
| *VvMRP20* | *VvABCC20* | GSVIVT01021595001 | EE068925 | 110691151 | Carmenere | Cluster | Clusters 4 cm |
|  |  |  | CAP0006_IIF_H07 | 34549912 | Cabernet Sauvignon | Petiole | Onset of Veraison (berry softening) |
|  |  |  | EE087726 | 110711013 | Thompson-seedless | Fruit | Fruits 7-9 mm |
|  |  |  | sT7aVVM024N06019 | 161715498 | Cabernet Sauvignon | Roots | 10 cm high plants grown in Magenta boxes |
|  |  |  | EE087531 | 110710922 | Thompson-seedless | Fruit | Fruits 7-9 mm |
|  |  |  | VVD174E02_378191 | 30124801 | Chardonnay | Berries | Mixed; 8, 9, 11, 13, 15, 16 weeks daf lk |
|  |  |  | VV_PEa20h06.b1 | 156727325 | Perlette | Bud | Mature |
|  |  |  | VV_PEa20h06.g1 | 156727326 | Perlette | Bud | Mature |
|  |  |  | C4B04254 | 110695705 | Carmenere | Cluster | Veraison |
|  |  |  | VV_PEa10h09.g1 | 156725511 | Perlette | Bud | Mature |
|  |  |  | VV_PEa10h09.b1 | 156725510 | Perlette | Bud | Mature |
|  |  |  | WIN056.C21_D12 | 110373883 | Cabernet Sauvignon | Flower leaf and root | Flower, pre-anthesis; leaf, fully expanded; root, produced by air-layering |
| *VvMRP21* | *VvABCC21* | GSVIVT01027990001 | sT7aVVM026F04012 | 161720711 | Cabernet Sauvignon | Roots | 10 cm high plants grown in Magenta boxes |
|  |  |  | sT7aVVM012N21083 | 161719171 | Cabernet Sauvignon | Roots | 10 cm high plants grown in Magenta boxes |
|  |  |  | sT7aVVM006L18069 | 161713323 | Cabernet Sauvignon | Roots | 10 cm high plants grown in Magenta boxes |
|  |  |  | sT7aVVM_AER70A05 | 161706119 | Cabernet Sauvignon | Roots | 10 cm high plants grown in Magenta boxes |
|  |  |  | CA32EN0002_IIaR_G04 | 28962947 | Cabernet Sauvignon | Leaf | Mid-season leaf material, collected July 25, 2001 |
|  |  |  | VVB017D03_393749 | 30328809 | Chardonnay | Leaf | Juvenile and adult |
|  |  |  | CAP0004_IF_F05 | 34548505 | Cabernet Sauvignon | Petiole | Onset of Veraison (berry softening) |
|  |  |  | CAP0004_IR_F05 | 34548593 | Cabernet Sauvignon | Petiole | Onset of Veraison (berry softening) |
|  |  |  | CAB40007_IIa_Ra_E08 | 30302530 | Cabernet Sauvignon | Berry | Berry on stage II, 9 mm |
|  |  |  | VVB017D03_393103 | 30328486 | Chardonnay | Leaf | Juvenile and adult |
|  |  |  | WIN047.C21_A05 | 110366053 | Cabernet Sauvignon | Pericarp | Fruit set to maturity |
|  |  |  | VV_PEb07f01.g1 | 156729238 | Perlette | Bud | Mature |
|  |  |  | VV_PEb07f01.b1 | 156729245 | Perlette | Bud | Mature |
|  |  |  | VV_PEa016c03.b1 | 156724247 | Perlette | Bud | Mature |
|  |  |  | VV_PEa16c03.g1 | 156726789 | Perlette | Bud | Mature |
|  |  |  | WIN057.C21_N01 | 110374208 | Cabernet Sauvignon | Flower leaf and root | Flower, pre-anthesis; leaf, fully expanded; root, produced by air-layering |
|  |  |  | VVL069F02_686534 | 71883399 | Cabernet Sauvignon | ruit with seeds removed | mixed 36-38 - modified E-L system (Brix > 15) |
|  |  |  | VVB017D03_131828 | 27579790 | Chardonnay | Leaf | Juvenile and adult |
|  |  |  | VVB135B11_406025 | 32267900 | Chardonnay | Leaf | Juvenile and adult |
|  |  |  | CAB40007_IIa_Fa_E08 | 30302459 | Cabernet Sauvignon | Berry | Berry on stage II, 9 mm |
|  |  |  | VVA017E11_119962 | 27755215 | Chardonnay | Leaf | Juvenile and adult |
|  |  |  | WIN053.C21_I07 | 110371284 | Cabernet Sauvignon | Flower leaf and root | Flower, pre-anthesis; leaf, fully expanded; root, produced by air-layering |
|  |  |  | VVA017E11_391329 | 30320606 | Chardonnay | Leaf | Juvenile and adult |
| *VvMRP22* | *VvABCC22* | GSVIVT01028440001 | VVI068A01_593066 | 71872558 | Cabernet Sauvignon | Inflorescence including flowers | 12 - modified E-L system |
|  |  |  | VVA028C09_55407 | 18459678 | Chardonnay | leaf | juvenile and adult |
|  |  |  | VVI169G04_610126 | 77580656 | Cabernet Sauvignon | Inflorescence including flowers | 12 - modified E-L system |
|  |  |  | VVI155G11_607728 | 77578484 | Cabernet Sauvignon | Inflorescence including flowers | 12 - modified E-L system |
|  |  |  | VVA028C09_402825 | 32245329 | Chardonnay | leaf | juvenile and adult |
|  |  |  | VRK130 | 47495013 | Vitis riparia | bud | paradormant |
|  |  |  | FAMU_USDA_FP_6869 | 51581010 | Vitis shuttleworthii | Entire tendril, leaves, bud, flowers | At blooming |
|  |  |  | C3B02196 | 110694280 | Carmenere | Cluster | Clusters 4 cm |
|  |  |  | WIN081.C21_C18 | 110394519 | Cabernet Sauvignon | seed | Fruit set to maturity |
|  |  |  | VV_PEa016e01.b1 | 156723994 | Perlette | Bud | Mature |
|  |  |  | C2B02093 | 110687165 | Carmenere | Bud - cluster |  |
|  |  |  | VVC001D04_125916 | 27582248 | Chardonnay | berries | mixed; 8, 9, 11, 13, 15, 16 weeks daf |
|  |  |  | VRK130T7 | 47495014 | Vitis riparia | bud | paradormant |
|  |  |  | VV_PEa25b03.b1 | 156728016 | Perlette | bud | Mature |
|  |  |  | VVI021F04_586288 | 71869169 | Cabernet Sauvignon | Inflorescence including flowers | 12 - modified E-L system |
|  |  |  | INFIO01_000261 |  |  | inflorescence | young inflorescence before flowering |
|  |  |  | VVL078D11_688032 |  |  | Fruit with seeds removed | mixed 36-38 - modified E-L system (Brix > 15) |
|  |  |  | BACCA01_001710 |  |  | Berry | Verasion |
| *VvMRP23* | *VvABCC223* | GSVIVT01028459001 | VVA028C09_55407 | 18459678 | Chardonnay | Leaf | Juvenile and adult |
|  |  |  | VVI169G04_610126 | 77580656 | Cabernet Sauvignon | Inflorescence including flowers | 12 - modified E-L system |
|  |  |  | VVI155G11_607728 | 77578484 | Cabernet Sauvignon | Inflorescence including flowers | 13 - modified E-L system |
|  |  |  | VVA028C09_402825 | 32245329 | Chardonnay | Leaf | Juvenile and adult |
|  |  |  | WIN0559.C21_E01 | 110388820 | Cabernet Sauvignon | Flower leaf and root | Flower, pre-anthesis; leaf, fully expanded; root, produced by air-layering |
|  |  |  | FAMU_USDA_FP_6869 | 51581010 | Vitis shuttleworthii | Entire tendril, leaves, bud, flowers | At blooming |
|  |  |  | WIN0559.C21_E01 | 110388820 | Cabernet Sauvignon | Flower leaf and root | Flower, pre-anthesis; leaf, fully expanded; root, produced by air-layering |
|  |  |  | Fag-B-IES1,5h-K1-100 | 118498743 | Vitis cinerea x Vitis riparia | Root | Root tip tissue |
|  |  |  | VVI021F04_586288 | 71869169 | Cabernet Sauvignon | Inflorescence including flowers | 12 - modified E-L system |
|  |  |  | CA48EN0001_IIIbF_B01 | 29783877 | Cabernet Sauvignon | Berry | Berry stage I |
|  |  |  | VVI068A01_593066 | 71872558 | Cabernet Sauvignon | Inflorescence including flowers | 12 - modified E-L system |
|  |  |  | VRK130 | 47495013 | Vitis riparia | Bud | Paradormant |
|  |  |  | VRK130T7 | 47495014 | Vitis riparia | Bud | Paradormant |
|  |  |  | INFIO01_000261 | 37190122 | Regent | Inflorescence | Young inflorescence before flowering |
|  |  |  | VVA028C09_55407 | 18459678 | Chardonnay | Leaf | Juvenile and adult |
|  |  |  | VVI155G11_607728 | 77578484 | Cabernet Sauvignon | Inflorescence | 12 - modified E-L system |
|  |  |  | C2B02093 | 110687165 | Carmenere | Bud - cluster |  |
|  |  |  | VVA028C09_402825 | 32245329 | Chardonnay | Leaf | Juvenile and adult |
|  |  |  | WIN081.C21_C18 | 110394519 | Cabernet Sauvignon | Seed | Fruit set to maturity |
| *VvMRP24* | *VvABCC24* | GSVIVT01028722001 | VVL151G10_700688 | 77588858 | Cabernet Sauvignon | Fruit with seeds removed | mixed 36-38 - modified E-L system (Brix > 15) |
|  |  |  | S6B03013 | 110713048 | Thompson-seedless | Fruit | Fruits 7-9 mm |
|  |  |  | C4B05477 | 110695979 | Carmenere | Cluster | Verasion |
|  |  |  | VVH051A09_747293 | 71860768 | Cabernet Sauvignon | Nectary of flowers | 25 - modified E-L system |
|  |  |  | WIN0416.C21_A17 | 110368362 | Cabernet Sauvignon | Pericarp | Fruit set to maturity |
|  |  |  | CAB40003_IVa_Fa_G07 | 30300354 | Cabernet Sauvignon | Berry | Berry on stage II, 9 mm |
|  |  |  | CAB40001_Ia_Fa_F08 | 30299486 | Cabernet Sauvignon | Berry | Berry on stage II, 9 mm |
|  |  |  | SBB02819 | 110726701 | Thompson-seedless | Inflorescence |  |
|  |  |  | CSECS141C03_CELu0001 | 45771084 | Cabernet Sauvignon | Cell Suspension Culture | |
|  |  |  | EST0088 | 10283302 | Pinot Noir | Fruit | berries harvested at veraison (onset of ripening) |
|  |  |  | CAbud0005_IVF_H12 | 34544835 | Cabernet Sauvignon | Bud | re-bloom (10-11 days before bloom) |
|  |  |  | sT7aVVM_AER20D06 | 161706059 | Cabernet Sauvignon | Roots | 10 cm high plants grown in Magenta boxes |
|  |  |  | CAB40003_IVa_Ra_G07 | 34544835 | Cabernet Sauvignon | Berry | Berry on stage II, 9 mm |
|  |  |  | WIN0548.C21_D14 | 110385427 | Cabernet Sauvignon | Flower, leaf and root | Flower, pre-anthesis; leaf, fully expanded; root, produced by air-layering |
|  |  |  | VVB058C10_327276 | 30323099 | Chardonnay | Leaf | Juvenile and Adult |
|  |  |  | S6B05345 | 110713613 | Thompson-seedless | Fruit | Fruits 7-9 mm |
|  |  |  | C4B03066 | 110695427 | Carmenere | Cluster | Veraison |
|  |  |  | S1G05217 | 110699494 | Thompson-seedless | Fruit and Flower |  |
|  |  |  | CSECS065C03_PREu0032 | 34362924 | Cabernet Sauvignon | Fruit with seeds removed | 32 - modified E-L system |
|  |  |  | CAbud0001_IVR_A03 | 34542820 | Cabernet Sauvignon | Bud | Pre-bloom (10-11 days before bloom) |
|  |  |  | CAbud0001_IVF_A03 | 34542733 | Cabernet Sauvignon | Bud | Pre-bloom (10-11 days before bloom) |
|  |  |  | WIN0543.C21_I03 | 110383665 | Cabernet Sauvignon | Flower, leaf and root | Flower, pre-anthesis; leaf, fully expanded; root, produced by air-layering |
|  |  |  | CAbud0005_IVR_H12 | 34544912 | Cabernet Sauvignon | Bud | Pre-bloom (10-11 days before bloom) |
|  |  |  | CAB2SG0003_IaR_E06 | 28968740 | Cabernet Sauvignon | Berry | Veraison |
|  |  |  | CSECS006H09_PREu0032 | 34361911 | Cabernet Sauvignon | Fruit with seeds removed | 32 - modified E-L system |
|  |  |  | WIN1150.C21_G02 | 110427992 | Muscat Hamburg | Berry | Anthesis flower to prior to veraison |
|  |  |  | CAB2SG0003_IaF_E06 | 28968671 | Cabernet Sauvignon | Berry | Veraison |
|  |  |  | EST 7595 | 22015526 | Ugni Blanc | Fruit | Veraison stage |
| *VvMRP25* | *VvABCC25* | GSVIVT01028744001 | VVL005H05_675556 | 71877910 | Cabernet Sauvignon | Fruit with seeds removed | Mixed 36-38 - modified E-L system (Brix > 15) |
|  |  |  | VVL004H05_675378 | 71877821 | Cabernet Sauvignon | Fruit with seeds removed | Mixed 36-38 - modified E-L system (Brix > 15) |
|  |  |  | VVL006F07_675692 | 71877978 | Cabernet Sauvignon | Fruit with seeds removed | Mixed 36-38 - modified E-L system (Brix > 15) |
|  |  |  | VVL023H01_678624 | 71879444 | Cabernet Sauvignon | Fruit with seeds removed | Mixed 36-38 - modified E-L system (Brix > 15) |
|  |  |  | VVL001D03_674792 | 71877528 | Cabernet Sauvignon | Fruit with seeds removed | Mixed 36-38 - modified E-L system (Brix > 15) |
|  |  |  | VVL015F03_677220 | 71878742 | Cabernet Sauvignon | Fruit with seeds removed | Mixed 36-38 - modified E-L system (Brix > 15) |
|  |  |  | VVL007G05_675884 | 71878074 | Cabernet Sauvignon | Fruit with seeds removed | Mixed 36-38 - modified E-L system (Brix > 15) |
|  |  |  | VVL034E09_680504 | 71880384 | Cabernet Sauvignon | Fruit with seeds removed | Mixed 36-38 - modified E-L system (Brix > 15) |
|  |  |  | VVL034E09_680504 | 71880384 | Cabernet Sauvignon | Fruit with seeds removed | Mixed 36-38 - modified E-L system (Brix > 15) |
|  |  |  | VVL025E08_678930 | 71879597 | Cabernet Sauvignon | Fruit with seeds removed | Mixed 36-38 - modified E-L system (Brix > 15) |
|  |  |  | VVL021C05_678170 | 71879217 | Cabernet Sauvignon | Fruit with seeds removed | Mixed 36-38 - modified E-L system (Brix > 15) |
|  |  |  | VVL015D09_677186 | 71878725 | Cabernet Sauvignon | Fruit with seeds removed | Mixed 36-38 - modified E-L system (Brix > 15) |
|  |  |  | VVL036C07_680782 | 71880523 | Cabernet Sauvignon | Fruit with seeds removed | Mixed 36-38 - modified E-L system (Brix > 15) |
|  |  |  | VVL011B10_676456 | 71878360 | Cabernet Sauvignon | Fruit with seeds removed | Mixed 36-38 - modified E-L system (Brix > 15) |
|  |  |  | VVL009D06_676154 | 71878209 | Cabernet Sauvignon | Fruit with seeds removed | Mixed 36-38 - modified E-L system (Brix > 15) |
|  |  |  | VVL012A05_676598 | 71878431 | Cabernet Sauvignon | Fruit with seeds removed | Mixed 36-38 - modified E-L system (Brix > 15) |
|  |  |  | VVL154B12_701102 | 77589227 | Cabernet Sauvignon | Fruit with seeds removed | Mixed 36-38 - modified E-L system (Brix > 15) |
|  |  |  | WIN0548.C21_D14 | 110385427 | Cabernet Sauvignon | Flower leaf and root | Flower, pre-anthesis; leaf, fully expanded; root, produced by air-layering |
|  |  |  | S1G05217 | 110699494 | Thompson-seedless | Fruit and Flower |  |
|  |  |  | CAB2SG0003_IaR_E06 | 28968740 | Cabernet Sauvignon | Berry | Veraison |
|  |  |  | S6B03013 | 110713048 | Thompson-seedless | Fruit | Fruits 7-9 mm |
| *VvMRP26* | *VvABCC26* | GSVIVT01037789001 | VVL004A01_675224 | 71877744 | Cabernet Sauvignon | Fruit with seed removed | Mixed 36-38 -modified E-L system (Brix > 15) |
|  |  |  | VVD106E08_369847 | 30130754 | Chardonnay | Berries | Mixed; 8, 9, 11, 13, 15, 16 weeks daf |
|  |  |  | VV_PEa16e01.g1 | 156726808 | Perlette | Bud | Mature |
|  |  |  | VV_PEa016e01.b1 | 156723994 | Perlette | Bud | Mature |
|  |  |  | VV_PEa25b03.g1 | 156728017 | Perlette | Bud | Mature |
|  |  |  | VV_PEa25b03.b1 | 156728016 | Perlette | Bud | Mature |
|  |  |  | VVI021F04_586288 | 71869169 | Cabernet Sauvignon | Inflorescence including flower | 12 - modified E-L system |
