# Supplementary material for: Whole-Genome Survey of the Putative ATP-Binding Cassette Transporter Family Genes in Vitis vinifera
Source: PLoS One. 2013 Nov 11;8(11):e78860. doi: 10.1371/journal.pone.0078860 (PMC3823996; doi:10.1371/journal.pone.0078860)
Supplement: Table S7 — Expressed sequence taqs (ESTs) identified for ABCD (PMP) and ABCE (RLI) subfamilies in Vitis vinifera . The protein name, Vitis proteome 12x ID, GenBank ID, EST name, cultivar/tissue type, and development stage are given for each gene. (DOC) [file pone.0078860.s007.doc]

**Table S7.** Expressed sequence taqs (ESTs) identified for ABCD (PMP) and ABCE (RLI) subfamilies in *Vitis vinifera*.The protein name, *Vitis* proteome 12x ID, GenBank ID, EST name, cultivar/tissue type, and development stage are given for each gene.

| **Name** | | ***Vitis* 12X ID** | **EST Name** | **GenBank ID** | **Species/Cultivar** | **Tissue Type** | **Development Stage** |
| --- | --- | --- | --- | --- | --- | --- | --- |
| *VvPMP1* | *VvABCD1* | GSVIVT01036685001 | RR890915I0002_IIIa_Fa_H09 | 33398466 | Vitis hybrid cultivar | Leaf | 17-week old greenhouse grown plants |
|  |  |  | RR890915I0002_IIIa_Ra_H09 | 33398526 | Vitis hybrid cultivar | Leaf | 17-week old greenhouse grown plants |
|  |  |  | RR890915I0007_IVc_rc_A01 | 33401211 | Vitis hybrid cultivar | Leaf | 17-week old greenhouse grown plants |
|  |  |  | VVB176B02_413481 | 32271628 | Chardonnay | Leaf | Juvenile and adult |
|  |  |  | VVB070E01_333252 | 30323800 | Chardonnay | Leaf | Juvenile and adult |
|  |  |  | CA32EN0004_IIIbR_H07 | 28963935 | Cabernet Sauvignon | Leaf | Mid-season leaf material, collected July 25, 2001 |
|  |  |  | CA32EN0004_IIIaF_H07 | 29785666 | Cabernet Sauvignon | Leaf | Mid-season leaf material, collected July 25, 2001 |
|  |  |  | WIN023.C21_E20 | 110364349 | Cabernet Sauvignon | Flower, leaf and root | Flower, pre-anthesis; leaf, fully expanded; root, produced by air-layering |
| *VvRLI1* | *VvABCE1* | GSVIVT01036876001 | WIN113.C21_C10 | 110414049 | Muscat Hamburg | Berry | Anthesis flower to prior to veraison |
|  |  |  | WIN0411.C21_E04 | 110366800 | Cabernet Sauvignon | Pericarp | Fruit set to maturity |
|  |  |  | WIN018.C21_N07 | 110360217 | Cabernet Sauvignon | Pericarp | Fruit set to maturity |
|  |  |  | WIN0527.C21_G24 | 110377886 | Cabernet Sauvignon | Flower, leaf and root | Flower, pre-anthesis; leaf, fully expanded; root, produced by air-layering |
|  |  |  | WIN029.TB24_G17 | 110362091 | Cabernet Sauvignon | Flower, leaf and root | Flower, pre-anthesis; leaf, fully expanded; root, produced by air-layering |
|  |  |  | VVH019B09_741593 | 71862412 | Cabernet Sauvignon | Nectary of flowers | 25 - modified E-L system |
|  |  |  | CA12EI301IIF_C02 | 26264772 | Cabernet Sauvignon | Leaf | Mid-season leaf material |
|  |  |  | EE087785 | 110711040 | Thompson-seedless | Fruit |  |
|  |  |  | CA12EI301IIR_C02 | 26266843 | Cabernet Sauvignon | Leaf | Mid-season leaf material |
