# Supplementary material for: Whole-Genome Survey of the Putative ATP-Binding Cassette Transporter Family Genes in Vitis vinifera
Source: PLoS One. 2013 Nov 11;8(11):e78860. doi: 10.1371/journal.pone.0078860 (PMC3823996; doi:10.1371/journal.pone.0078860)
Supplement: Table S8 — Expressed sequence taqs (ESTs) identified for ABCF (GCN) subfamily in Vitis vinifera . The protein name, Vitis proteome 12x ID, GenBank ID, EST name, cultivar/tissue type, and development stage are given for each gene. (DOC) [file pone.0078860.s008.doc]

**Table S8.** Expressed sequence taqs (ESTs) identified for ABCF (GCN) subfamily in *Vitis vinifera*.The protein name, *Vitis* proteome 12x ID, GenBank ID, EST name, cultivar/tissue type, and development stage are given for each gene.

| **Name** | | ***Vitis* 12X ID** | **EST Name** | **GenBank ID** | **Species/Cultivar** | **Tissue Type** | **Development Stage** |
| --- | --- | --- | --- | --- | --- | --- | --- |
| *VvGCN1* | *VvABCF1* | GSVIVT01001694001 | sT7aVVM027B18079 | 161720613 | Cabernet Sauvignon | Roots | 10 cm high plants grown in Magenta boxes |
|  |  |  | sT7aVVM_AER64A11 | 161705785 | Cabernet Sauvignon | Roots | 10 cm high plants grown in Magenta boxes |
|  |  |  | VV_PEa013d08.b1 | 156724056 | Perlette | Bud | Mature |
|  |  |  | VV_PEa13d08.g1 | 156726557 | Perlette | Bud | Mature |
|  |  |  | VVG035H12_758121 | 71857548 | Cabernet Sauvignon | Cell suspension culture | |
|  |  |  | VVB045G02_324618 | 30321770 | Chardonnay | Leaf | Juvenile and adult |
|  |  |  | S8B02158 | 110718574 | Thompson-seedless | Fruit | Veraison |
|  |  |  | VVL111C02_693612 | 71886938 | Cabernet Sauvignon | Fruit with seeds removed | Mixed 36-38 - modified E-L system (Brix > 15) |
|  |  |  | VVB013H10_125840 | 27579494 | Chardonnay | Leaf | Juvenile and adult |
|  |  |  | VVB218F02_435113 | 32250104 | Chardonnay | Leaf | Juvenile and adult |
|  |  |  | VVB020E06_132380 | 27580066 | Chardonnay | Leaf | Juvenile and adult |
|  |  |  | VVB038H03_229354 | 27581684 | Chardonnay | Leaf | Juvenile and adult |
|  |  |  | VVB013H10_403995 | 32247005 | Chardonnay | Leaf | Juvenile and adult |
|  |  |  | VVB013H10_403307 | 32246661 | Chardonnay | Leaf | Juvenile and adult |
|  |  |  | VVL014G03_677064 | 71878664 | Cabernet Sauvignon | Fruit with seeds removed | Mixed 36-38 - modified E-L system (Brix > 15) |
|  |  |  | VVL041B09_681604 | 71880934 | Cabernet Sauvignon | Fruit with seeds removed | Mixed 36-38 - modified E-L system (Brix > 15) |
|  |  |  | VVL059B12_684736 | 71882500 | Cabernet Sauvignon | Fruit with seeds removed | Mixed 36-38 - modified E-L system (Brix > 15) |
|  |  |  | VVL069F01_686532 | 71883398 | Cabernet Sauvignon | Fruit with seeds removed | Mixed 36-38 - modified E-L system (Brix > 15) |
|  |  |  | VVG056E01_761925 | 71859450 | Cabernet Sauvignon | Cell suspension culture | |
|  |  |  | VVG056E01_761925 | 71859450 | Cabernet Sauvignon | Cell suspension culture | |
|  |  |  | WIN0415.C21_E12 | 110368115 | Cabernet Sauvignon | Pericarp | Fruit set to maturity |
|  |  |  | WIN071.C21_D01 | 110393218 | Cabernet Sauvignon | Pericarp | Fruit set to maturity |
|  |  |  | CAB70002_IVaR_E04 | 30303686 | Cabernet Sauvignon | Berry | Post-Veraison, 18-19 brix |
|  |  |  | S6B01870 | 110711450 | Thompson-seedless | Fruit | Fruits 7-9 mm |
|  |  |  | WIN1110.C21_K04 | 110414838 | Muscat Hamburg | Berry | Anthesis flower to prior to veraison |
|  |  |  | EST 5461 | 22011489 | Shiraz | Fruit | Ripening stage |
|  |  |  | WIN0555.C21_L24 | 110387766 | Cabernet Sauvignon | Flower, leaf and root | Flower, pre-anthesis; leaf, fully expanded; root, produced |
|  |  |  | CSECS068A11_PREu0032 | 34363175 | Cabernet Sauvignon | Fruit with seeds removed | 32 - modified E-L system |
|  |  |  | WIN1133.C21_P12 | 110422214 | Muscat Hamburg | Berry | Anthesis flower to prior to veraison |
| *VvGCN2* | *VvABCF2* | GSVIVT01019609001 | VVL007A12_675772 | 71878018 | Cabernet Sauvignon | Fruit with seeds removed | mixed 36-38 - modified E-L system (Brix > 15) |
|  |  |  | VRJ627T7 | 41021529 | Vitis riparia | Bud | Dormant |
|  |  |  | VRJ627 | 41021528 | Vitis riparia | Bud | Dormant |
|  |  |  | WIN0810.C21_O09 | 110395537 | Cabernet Sauvignon | Seed | Fruit set to maturity |
|  |  |  | WIN072.C21_H15 | 110394159 | Cabernet Sauvignon | Pericarp | Fruit set to maturity |
|  |  |  | VVG032C02_757429 | 71857202 | Cabernet Sauvignon | Cell suspension culture | |
|  |  |  | VVG001A08_752243 | 71854609 | Cabernet Sauvignon | Cell suspension culture | |
|  |  |  | VVH016E12_741115 | 71862173 | Cabernet Sauvignon | Nectary of flowers | 25 - modified E-L system |
|  |  |  | sT7aVVM020L16054 | 161716294 | Cabernet Sauvignon | Roots | 10 cm high plants grown in Magenta boxes |
|  |  |  | sT7aVVM005E03012 | 161713700 | Cabernet Sauvignon | Roots | 10 cm high plants grown in Magenta boxes |
|  |  |  | FAMU_USDA_FP_227 | 51574368 | Vitis shuttleworthii | Entire tendril, leaves, bud, flowers | At blooming |
|  |  |  | FAMU_USDA_FP_2342 | 51576483 | Vitis shuttleworthii | Entire tendril, leaves, bud, flowers | At blooming |
|  |  |  | VVH008B07_739581 | 71865014 | Cabernet Sauvignon | Nectary of flowers | 25 - modified E-L system |
|  |  |  | VVL049C11_683022 | 71881643 | Cabernet Sauvignon | Fruit with seeds removed | Mixed 36-38 - modified E-L system (Brix > 15) |
| *VvGCN3* | *VvABCF3* | GSVIVT01022235001 | CAB40006_IIa_Fa_F05 | 30301899 | Cabernet Sauvignon | Berry | Berry on stage II, 9 mm |
|  |  |  | VVB150E11_408867 | 32269321 | Chardonnay | Leaf | Juvenile and adult |
|  |  |  | VVB144B05_407691 | 32268733 | Chardonnay | Leaf | Juvenile and adult |
|  |  |  | VVB066C02_332526 | 30323437 | Chardonnay | Leaf | Juvenile and adult |
|  |  |  | EST 10526 | 32456242 | Chardonnay | Fruit without seeds | Veraison stage |
|  |  |  | EST 10502 | 32456218 | Chardonnay | Fruit without seeds | Veraison stage |
|  |  |  | EST 10494 | 32456210 | Chardonnay | Fruit without seeds | Veraison stage |
|  |  |  | EST 10534 | 32456250 | Chardonnay | Fruit without seeds | Veraison stage |
|  |  |  | EST 10489 | 32456205 | Chardonnay | Fruit without seeds | Veraison stage |
|  |  |  | EST 10536 | 32456252 | Chardonnay | Fruit without seeds | Veraison stage |
|  |  |  | EST 10559 | 32456275 | Chardonnay | Fruit without seeds | Veraison stage |
|  |  |  | EST 10513 | 32456229 | Chardonnay | Fruit without seeds | Veraison stage |
|  |  |  | EST 10495 | 32456211 | Chardonnay | Fruit without seeds | Veraison stage |
|  |  |  | EST 10505 | 32456221 | Chardonnay | Fruit without seeds | Veraison stage |
|  |  |  | EST 10490 | 32456206 | Chardonnay | Fruit without seeds | Veraison stage |
|  |  |  | EST 10504 | 32456220 | Chardonnay | Fruit without seeds | Veraison stage |
|  |  |  | VVD143B02_374515 | 30126973 | Chardonnay | Berries | Mixed; 8, 9, 11, 13, 15, 16 weeks daf |
|  |  |  | EST 10500 | 32456216 | Chardonnay | Fruit without seeds | Veraison stage |
|  |  |  | EST 10514 | 32456230 | Chardonnay | Fruit without seeds | Veraison stage |
|  |  |  | EST 10461 | 32456177 | Chardonnay | Fruit without seeds | Veraison stage |
|  |  |  | EST 10470 | 32456186 | Chardonnay | Fruit without seeds | Veraison stage |
|  |  |  | EST 10551 | 32456267 | Chardonnay | Fruit without seeds | Veraison stage |
|  |  |  | EST 10550 | 32456266 | Chardonnay | Fruit without seeds | Veraison stage |
|  |  |  | EST 10491 | 32456207 | Chardonnay | Fruit without seeds | Veraison stage |
|  |  |  | VVB059C01_326168 | 30322545 | Chardonnay | Leaf | Juvenile and adult |
|  |  |  | INFIO01_000116 | 37189985 | Regent | Inflorescence | Young inflorescence before flowering |
|  |  |  | VVB153A02_409265 | 32269520 | Chardonnay | Leaf | Juvenile and adult |
|  |  |  | CAB40006_IIa_Ra_F05 | 30301970 | Cabernet Sauvignon | Berry | Berry on stage II, 9 mm |
|  |  |  | VVB071A11_333364 | 30323856 | Chardonnay | Leaf | Juvenile and adult |
|  |  |  | CA32EN0003_IIaF_C06 | 29785439 | Cabernet Sauvignon | Leaf | Mid-season leaf material, collected July 25, 2001 |
|  |  |  | WIN1117.C21_O08 | 110417069 | Muscat Hamburg | Berry | Anthesis flower to prior to veraison |
|  |  |  | CA48LN07IIIF-D3 | 26261763 | Cabernet Sauvignon | Leaf | Leaf season sample |
|  |  |  | CA12EI302IIIR_F08 | 26267092 | Cabernet Sauvignon | Leaf | Mid-season leaf material |
| *VvGCN4* | *VvABCF4* | GSVIVT01031505001 | WIN054.C21_O05 | 110371526 | Cabernet Sauvignon | Flower, leaf and root | Flower, pre-anthesis; leaf, fully expanded; root, producedby air-layering |
|  |  |  | SCB03370 | 110730109 | Thompson-seedless | Inflorescence |  |
|  |  |  | VV_PEa10a04.g1 | 156726032 | Perlette | Bud | Mature |
|  |  |  | VV_PEa10a04.b1 | 156726029 | Perlette | Bud | Mature |
|  |  |  | BACCA01_001241 | 37184573 | Pinot Noir | Berry | Veraison |
|  |  |  | C1C00369 | 110685352 | Carmenere | Fruit, bud and cluster |  |
|  |  |  | SCB02225 | 110732580 | Thompson-seedless | Inflorescence |  |
|  |  |  | S9B06941 | 110723139 | Thompson-seedless | Berry |  |
|  |  |  | VVB060C12_326336 | 30322629 | Chardonnay | Leaf | Juvenile and adult |
|  |  |  | S8B01582 | 110719826 | Thompson-seedless | Fruit | Veraison |
|  |  |  | VVB193G06_430833 | 32247964 | Chardonnay | Leaf | Juvenile and adult |
|  |  |  | VV_PEd01e11.g1 | 156737460 | Perlette | Bud | Young |
|  |  |  | S6B04530 | 110712106 | Thompson-seedless | Fruit | Fruits 7-9 mm |
|  |  |  | S9B08174 | 110723442 | Thompson-seedless | Berry | Ripening berries |
|  |  |  | S9B02841 | 110722371 | Thompson-seedless | Berry | Ripening berries |
|  |  |  | VVD163C06_376487 | 30127876 | Chardonnay | Berries | mixed; 8, 9, 11, 13, 15, 16 weeks daf |
|  |  |  | WIN054.C21_O05 | 110371526 | Cabernet Sauvignon | Flower, leaf and root | Flower, pre-anthesis; leaf, fully expanded; root, producedby air-layering |
|  |  |  | VVB060C12_326336 | 30322629 | Chardonnay | Leaf | Juvenile and adult |
|  |  |  | VVB193G06_430833 | 32247964 | Chardonnay | Leaf | Juvenile and adult |
|  |  |  | sT7aVVM_AER96H06 | 161706246 | Cabernet Sauvignon | Roots | 10 cm high plants grown in Magenta boxes |
|  |  |  | sT7aVVM004H24090 | 161713901 | Cabernet Sauvignon | Roots | 10 cm high plants grown in Magenta boxes |
|  |  |  | EST 15345 | 46918027 | Shiraz | Fruit without seeds | Veraison stage |
| *VvGCN5* | *VvABCF5* | GSVIVT01034906001 | VVB013H10_403307 | 32246661 | Chardonnay | Leaf | Juvenile and adult |
|  |  |  | VVB013H10_403995 | 32247005 | Chardonnay | Leaf | Juvenile and adult |
|  |  |  | VVL014G03_677064 | 71878664 | Cabernet Sauvignon | Fruit with seeds removed | mixed 36-38 - modified E-L system (Brix > 15) |
|  |  |  | VV_PEa13d08.g1 | 156726557 | Perlette | Bud | Mature |
|  |  |  | WIN1133.C21_P12 | 110422214 | Muscat Hamburg | Berry | Anthesis flower to prior to veraison |
|  |  |  | CSECS068A11_PREu0032 | 34363175 | Cabernet Sauvignon | Fruit with seeds removed | 32 - modified E-L system |
|  |  |  | VVL059B12_684736 | 71882500 | Cabernet Sauvignon | Fruit with seeds removed | mixed 36-38 - modified E-L system (Brix > 15) |
|  |  |  | WIN0555.C21_L24 | 110387766 | Cabernet Sauvignon | Flower, leaf and root | Flower, pre-anthesis; leaf, fully expanded; root, producedby air-layering |
|  |  |  | EST 5461 | 22011489 | Shiraz | Fruit | Ripening stage |
|  |  |  | VVG056E01_761925 | 71859450 | Cabernet Sauvignon | Cell suspension culture | |
|  |  |  | VVB038H03_229354 | 27581684 | Chardonnay | Leaf | Juvenile and adult |
|  |  |  | VVB020E06_132380 | 27580066 | Chardonnay | Leaf | Juvenile and adult |
|  |  |  | VVD074A07_353961 | 30136326 | Chardonnay | Berries | mixed; 8, 9, 11, 13, 15, 16 weeks daf |
|  |  |  | VVL069F01_686532 | 71883398 | Cabernet Sauvignon | Fruit with seeds removed | mixed 36-38 - modified E-L system (Brix > 15) |
|  |  |  | VV_PEa013d08.b1 | 156724056 | Perlette | Bud | Mature |
|  |  |  | VVB218F02_435113 | 32250104 | Chardonnay | Leaf | Juvenile and adult |
|  |  |  | sT7aVVM_AER64A11 | 161705785 | Cabernet Sauvignon | Roots | 10 cm high plants grown in Magenta boxes |
|  |  |  | WIN071.C21_D01 | 110393218 | Cabernet Sauvignon | Pericarp | Fruit set to maturity |
|  |  |  | S6B01870 | 110711450 | Thompson-seedless | Fruit |  |
|  |  |  | WIN1110.C21_K04 | 110414838 | Muscat Hamburg | Berry | Anthesis flower to prior to veraison |
|  |  |  | CAB70002_IVaR_E04 | 30303686 | Cabernet Sauvignon | Berry | Post-Veraison, 18-19 brix |
|  |  |  | VVB013H10_125840 | 27579494 | Chardonnay | Leaf | Juvenile and adult |
|  |  |  | VVG035H12_758121 | 71857548 | Cabernet Sauvignon | Cell suspension culture | |
|  |  |  | VVL111C02_693612 | 71886938 | Cabernet Sauvignon | Fruit with seeds removed | mixed 36-38 - modified E-L system (Brix > 15) |
|  |  |  | S8B02158 | 110718574 | Thompson-seedless | Fruit | Veraison |
|  |  |  | VVB045G02_324618 | 30321770 | Chardonnay | Leaf | Juvenile and adult |
|  |  |  | sT7aVVM027B18079 | 161720613 | Cabernet Sauvignon | Roots | 10 cm high plants grown in Magenta boxes |
