# Supplementary material for: Whole-Genome Survey of the Putative ATP-Binding Cassette Transporter Family Genes in Vitis vinifera
Source: PLoS One. 2013 Nov 11;8(11):e78860. doi: 10.1371/journal.pone.0078860 (PMC3823996; doi:10.1371/journal.pone.0078860)
Supplement: Table S9 — Expressed sequence taqs (ESTs) identified for ABCG (WBC and PDR) subfamily in Vitis vinifera . The protein name, Vitis proteome 12x ID, GenBank ID, EST name, cultivar/tissue type, and development stage are given for each gene. (DOC) [file pone.0078860.s009.doc]

**Table S9.** Expressed sequence taqs (ESTs) identified for ABCG (WBC and PDR) subfamily in *Vitis vinifera*.The protein name, *Vitis* proteome 12x ID, GenBank ID, EST name, cultivar/tissue type, and development stage are given for each gene.

| **Name** | | ***Vitis* 12X ID** | **EST Name** | **GenBank ID** | **Species/Cultivar** | **Tissue Type** | **Development Stage** |
| --- | --- | --- | --- | --- | --- | --- | --- |
| *VvWBC1* | *VvABCG1* | GSVIVT01001660001 | VRJ636T7 | 41021546 | *Vitis riparia* | Bud | Dormant |
| *VvWBC2* | *VvABCG2* | GSVIVT01002136001 | CSECS093D11_POSn0036 | 34416443 | Cabernet Sauvignon | Fruit with seeds removed | 36 - modified E-L system |
|  |  |  | VVL143F02_699284 | 71889774 | Cabernet Sauvignon | Fruit with seeds removed | Mixed 36-38 - modified E-L system (Brix > 15) |
|  |  |  | VVD002A09_127108 | 27586776 | Chardonnay | Berries | Mixed; 8, 9, 11, 13, 15, 16 weeks daf |
|  |  |  | S1G01109 | 110697990 | Thompson-seedless | Fruit and flower |  |
|  |  |  | sT7aVVM020J07024 | 161715343 | Cabernet Sauvignon | Roots | 10 cm high plants grown in Magenta boxes |
| *VvWBC3* | *VvABCG3* | GSVIVT01002139001 | VVD002A09_127108 | 27586776 | Chardonnay | Berries | Mixed; 8, 9, 11, 13, 15, 16 weeks daf |
|  |  |  | VVL143F02_699284 | 71889774 | Cabernet Sauvignon | Fruit with seeds removed | Mixed 36-38 - modified E-L system (Brix > 15) |
|  |  |  | S5B04076 | 110709578 | Thompson-seedless | Fruit | Fruits 7-9 mm |
|  |  |  | VVI053C10_590746 | 71871398 | Cabernet Sauvignon | Inflorescence including flowers | 12 - modified E-L system |
|  |  |  | C4B01316 | 110696555 | Carmenere | Cluster | Veraison |
|  |  |  | S1G01109 | 110697990 | Thompson-seedless | Fruit and Flower |  |
|  |  |  | WIN0519.C21_E07 | 110375326 | Cabernet Sauvignon | Flower, leaf and root | Flower, pre-anthesis; leaf, fully expanded; root, produced by air-layering |
|  |  |  | sT7aVVM020J07024 | 161715343 | Cabernet Sauvignon | Roots | 10 cm high plants grown in Magenta boxes |
| *VvWBC4* | *VvABCG4* | GSVIVT01002949001 | sT7aVVM020J07024 | 161715343 | Cabernet Sauvignon | Roots | 10 cm high plants grown in Magenta boxes |
|  |  |  | WIN0519.C21_E07 | 110375326 | Cabernet Sauvignon | Flower, leaf and root | Flower, pre-anthesis; leaf, fully expanded; root, produced by air-layering |
|  |  |  | CAB20007_Ia_Ra_D05 | 33405820 | Cabernet Sauvignon | Flower - bloom | Bloom |
|  |  |  | S5B04076 | 110709578 | Thompson-seedless | Fruit | Fruits 7-9 mm |
|  |  |  | VVL143F02_699284 | 71889774 | Cabernet Sauvignon | Fruit with seeds removed | Mixed 36-38 - modified E-L system (Brix > 15) |
|  |  |  | VVD002A09_127108 | 27586776 | Chardonnay | Berries | Mixed; 8, 9, 11, 13, 15, 16 weeks daf |
|  |  |  | CSECS093D11_POSn0036 | 34416443 | Cabernet Sauvignon | Fruit with seeds removed | 36 - modified E-L system |
| *VvWBC5* | *VvABCG5* | GSVIVT01003413001 | CSECS093D11_POSn0036 | 34416443 | Cabernet Sauvignon | Fruit with seeds removed | 36 - modified E-L system |
|  |  |  | VVL143F02_699284 | 71889774 | Cabernet Sauvignon | Fruit with seeds removed | Mixed 36-38 - modified E-L system (Brix > 15) |
|  |  |  | C4B01316 | 110696555 | Carmenere | Cluster | Veraison |
|  |  |  | VVD002A09_127108 | 27586776 | Chardonnay | Berries | Mixed; 8, 9, 11, 13, 15, 16 weeks daf |
|  |  |  | WIN0519.C21_E07 | 110375326 | Cabernet Sauvignon | Flower, leaf and root | Flower, pre-anthesis; leaf, fully expanded; root, produced by air-layering |
|  |  |  | sT7aVVM020J07024 | 161715343 | Cabernet Sauvignon | Roots | 10 cm high plants grown in Magenta boxes |
| *VvWBC6* | *VvABCG6* | GSVIVT01008456001 | FAMU_USDA_FP_2131 | 51576272 | Vitis shuttleworthii | Entire tendril, leaves, bud, flowers | At Blooming |
| *VvWBC7* | *VvABCG7* | GSVIVT01011781001 | CGF1000655_A08 | 33406999 | Cabernet Sauvignon | Stem | Pre-bloom (10-11 days before bloom) |
|  |  |  | CAP0005_IVF_C08 | 34549698 | Cabernet Sauvignon | Petiole | Onset of Veraison (berry softening) |
|  |  |  | CAbud0005_IVF_C08 | 34544781 | Cabernet Sauvignon | Bud | Pre-bloom (10-11 days before bloom) |
|  |  |  | WIN0514.C21_E14 | 110372843 | Cabernet Sauvignon | Flower, leaf and root | Flower, pre-anthesis; leaf, fully expanded; root, produced by air-layering |
|  |  |  | WIN0553.C21_I19 | 110387100 | Cabernet Sauvignon | Flower, leaf and root | Flower, pre-anthesis; leaf, fully expanded; root, produced by air-layering |
|  |  |  | CAbud0005_IVR_C08 | 34544864 | Cabernet Sauvignon | Bud | Pre-bloom (10-11 days before bloom) |
|  |  |  | VVD147E10_375135 | 30127283 | Chardonnay | Berries | Mixed; 8, 9, 11, 13, 15, 16 weeks daf |
|  |  |  | VV_PEc08H12.r.ab1 | 156734463 | Perlette | Bud | Mature |
|  |  |  | VVI057B07_591328 | 71871689 | Cabernet Sauvignon | Inflorescence including flowers | 12 - modified E-L system |
|  |  |  | CA32EN0004_IIaF_A10 | 28963945 | Cabernet Sauvignon | Leaf | Mid-season leaf material, collected July 25, 2001 |
|  |  |  | CA32EN0004_IIaR_A10 | 28964004 | Cabernet Sauvignon | Leaf | Mid-season leaf material, collected July 25, 2001 |
|  |  |  | VVI166E03_609582 | 77580128 | Cabernet Sauvignon | Inflorescence including flowers | 12 - modified E-L system |
| *VvWBC8* | *VvABCG8* | GSVIVT01011981001 | WIN0514.C21_O07 | 110373029 | Cabernet Sauvignon | Flower, leaf and root | Flower, pre-anthesis; leaf, fully expanded; root, produced by air-layering |
|  |  |  | CAbud0004_IIIF_C08 | 34543769 | Cabernet Sauvignon | Bud | Pre-bloom (10-11 days before bloom) |
|  |  |  | FAMU_USDA_FP_5577 | 51579718 | Vitis shuttleworthii | Entire tendril, leaves, bud, flowers | At Blooming |
|  |  |  | VVI103H04_598928 | 71866675 | Cabernet Sauvignon | Inflorescence including flowers | 12 - modified E-L system |
| *VvWBC9* | *VvABCG9* | GSVIVT01014222001 | USDA_FP_131945 | 47090589 | Vitis shuttleworthii | Entire tendril, leaves, bud, flowers | At blooming |
|  |  |  | SCB04533 | 110732112 | Thompson-seedless | Inflorescence |  |
|  |  |  | sT7aVVM018I07024 | 161718902 | Cabernet Sauvignon | Roots | 10 cm high plants grown in Magenta boxes |
|  |  |  | WIN1123.C21_A12 | 110418658 | Muscat Hamburg | Berry | Anthesis flower to prior to veraison |
| *VvWBC10* | *VvABCG10* | GSVIVT01014402001 | CAbud0004_IIIF_C08 | 34543769 | Cabernet Sauvignon | Bud | Pre-bloom (10-11 days before bloom) |
|  |  |  | CAbud0005_IVF_E08 | 34544801 | Cabernet Sauvignon | Bud | Pre-bloom (10-11 days before bloom) |
|  |  |  | SCB00162 | 110729397 | Thompson-seedless | Inflorescence |  |
|  |  |  | sT7aVVM011B10047 | 161719301 | Cabernet Sauvignon | Roots | 10 cm high plants grown in Magenta boxes |
|  |  |  | VVI103H04_598928 | 71866675 | Cabernet Sauvignon | Inflorescence including flowers | 12 - modified E-L system |
| *VvWBC11* | *VvABCG11* | GSVIVT01014733001 |  |  |  |  |  |
| *VvWBC12* | *VvABCG12* | GSVIVT01015767001 | sT7aVVM020J07024 | 161715343 | Cabernet Sauvignon | Roots | 10 cm high plants grown in Magenta boxes |
|  |  |  | CAB20007_Ia_Ra_D05 | 33405820 | Cabernet Sauvignon | Flower - bloom | Bloom |
|  |  |  | V-B-13C09 | 28602040 | Vitis aestivalis | Leaf | Young leaf |
|  |  |  | VVA019D10_54351 | 18459150 | Chardonnay | Leaf | Juvenile and adult |
|  |  |  | S5B04076 | 110709578 | Thompson-seedless | Fruit | Fruits 7-9 mm |
|  |  |  | VVA019D10_392483 | 30321183 | Chardonnay | Leaf | Juvenile and adult |
|  |  |  | VVD002A09_127108 | 27586776 | Chardonnay | Berries | Mixed; 8, 9, 11, 13, 15, 16 weeks daf |
|  |  |  | VVA019D10_391427 | 30320655 | Chardonnay | Leaf | Juvenile and adult |
|  |  |  | C4B01316 | 110696555 | Carmenere | Cluster | Veraison |
|  |  |  | CSECS173D06_POSu0038 | 83275941 | Cabernet Sauvignon | Fruit with seeds removed | 38 - modified E-L system |
|  |  |  | EST 9486 | 32460403 | Chardonnay | Fruit without seeds | Green stage |
|  |  |  | WIN117.C21_G17 | 110429664 | Muscat Hamburg | Berry | Anthesis flower to prior to veraison |
|  |  |  | VVD002A09_397057 | 30330463 | Chardonnay | Berries | Mixed; 8, 9, 11, 13, 15, 16 weeks daf |
| *VvWBC13* | *VvABCG13* | GSVIVT01015768001 | VVL143F02_699284 | 71889774 | Cabernet Sauvignon | Fruit with seeds removed | mixed 36-38 - modified E-L system (Brix > 15) |
|  |  |  | C4B01316 | 110696555 | Carmenere | Cluster | Veraison |
|  |  |  | sT7aVVM020J07024 | 161715343 | Cabernet Sauvignon | Roots | 10 cm high plants grown in Magenta boxes |
|  |  |  | V-B-13C09 | 28602040 | Vitis aestivalis | Leaf | Young leaf |
|  |  |  | VVD002A09_127108 | 27586776 | Chardonnay | Berries | Mixed; 8, 9, 11, 13, 15, 16 weeks daf |
|  |  |  | WIN0211.TB24_K22 | 110362707 | Cabernet Sauvignon | Flower, leaf and root | Flower, pre-anthesis; leaf, fully expanded; root, producedby air-layering |
|  |  |  | S5B04076 | 110709578 | Thompson-seedless | Fruit | Fruits 7-9 mm |
| *VvWBC14* | *VvABCG14* | GSVIVT01015771001 | V-B-13C09 | 28602040 | Vitis aestivalis | Leaf | Young leaf |
|  |  |  | WIN0211.TB24_K22 | 110362707 | Cabernet Sauvignon | Flower, leaf and root | Flower, pre-anthesis; leaf, fully expanded; root, producedby air-layering |
|  |  |  | VVA019D10_391427 | 30320655 | Chardonnay | Leaf | Juvenile and adult |
|  |  |  | C4B01316 | 110696555 | Carmenere | Cluster | Veraison |
| *VvWBC15* | *VvABCG15* | GSVIVT01016240001 | VVH032G06_743997 | 71860031 | Cabernet Sauvignon | Nectary of flowers | 25 - modified E-L system |
|  |  |  | CGF1000655_A08 | 33406999 | Cabernet Sauvignon | Stem | Pre-bloom (10-11 days before bloom) |
|  |  |  | CGF1000656_A08 | 33406911 | Cabernet Sauvignon | Stem | Pre-bloom (10-11 days before bloom) |
| *VvWBC16* | *VvABCG16* | GSVIVT01020687001 | WIN1123.C21_A12 | 110418658 | Muscat Hamburg | Berry | Anthesis flower to prior to veraison |
|  |  |  | SCB04533 | 110732112 | Thompson-seedless | Inflorescence |  |
|  |  |  | sT7aVVM018I07024 | 161718902 | Cabernet Sauvignon | Roots | 10 cm high plants grown in Magenta boxes |
| *VvWBC17* | *VvABCG17* | GSVIVT01022346001 | CAB20004_IIa_Fa_F11 | 33403474 | Cabernet Sauvignon | Flower | Bloom |
|  |  |  | C3B04878 | 110691983 | Carmenere | Cluster | Cluster 4 cm |
|  |  |  | USDA_FP_131945 | 47090589 | Vitis shuttleworthii | Entire tendril, leaves, bud, flowers | At blooming |
|  |  |  | CAP0007_IF_F09 | 34550169 | Cabernet Sauvignon | Petiole | Onset of Veraison (berry softening) |
|  |  |  | CSECS194C02_5_PREn0028 | 87583870 | Cabernet Sauvignon | Fruit | 28 - modified E-L system |
|  |  |  | CAB30002_Ic_Fc_A05 | 30296592 | Cabernet Sauvignon | Berry | Berry stage I |
|  |  |  | CAB20004_IIa_Ra_F11 | 33403557 | Cabernet Sauvignon | Flower | Bloom |
|  |  |  | CAB30002_Ia_Ra_A05 | 30296532 | Cabernet Sauvignon | Berry | Berry stage I |
|  |  |  | CAB30005_Ia_Ra_A05 | 30298054 | Cabernet Sauvignon | Berry | Berry stage I |
|  |  |  | C3B05569 | 110691344 | Carmenere | Clusters | Cluster 4 cm |
|  |  |  | CSECS194C02_PREn0028 | 83276280 | Cabernet Sauvignon | Fruit | 28 - modified E-L system |
|  |  |  | sT7aVVM_AER4H04 | 161707529 | Cabernet Sauvignon | Roots | 10 cm high plants grown in Magenta boxes |
|  |  |  | CAB10001_IIIa_Ra_C11 | 30251189 | Cabernet Sauvignon | Flower | Pre-bloom |
|  |  |  | CAB10001_IIIa_Fa_C11 | 30251103 | Cabernet Sauvignon | Flower | Pre-bloom |
|  |  |  | SCB04533 | 110732112 | Cabernet Sauvignon | Inflorescence |  |
|  |  |  | sT7aVVM_AER96B07 | 161708056 | Cabernet Sauvignon | Roots | 10 cm high plants grown in Magenta boxes |
| *VvWBC18* | *VvABCG18* | GSVIVT01022526001 | VRJ636T7 | 41021546 | Vitis riparia | Bud | Dormant |
|  |  |  | VRJ636 | 41021545 | Vitis riparia | Bud | Dormant |
|  |  |  | CAbud0005_IVF_E08 | 34544801 | Cabernet Sauvignon | Bud | Pre-bloom (10-11 days before bloom) |
|  |  |  | VVI105A02_599110 | 71866766 | Cabernet Sauvignon | Inflorescence including flowers | 12 - modified E-L system |
|  |  |  | VVI013B02_584862 | 71868456 | Cabernet Sauvignon | Inflorescence including flowers | 12 - modified E-L system |
|  |  |  | CSECS047H06_FLOn0012 | 34319305 | Cabernet Sauvignon | Inflorescence including flowers | 12 - modified E-L system |
|  |  |  | FAMU_USDA_FP_7158 | 51581299 | Vitis shuttleworthii | Entire tendril, leaves, bud, flowers | At Blooming |
| *VvWBC19* | *VvABCG19* | GSVIVT01024228001 | CGF1000817_B04 | 33408859 | Cabernet Sauvignon | Stem | Pre-bloom (10-11 days before bloom) |
|  |  |  | RR890915N0005_IVa_Ra_A04 | 33396814 | Vitis hybrid cultivar | Leaf |  |
|  |  |  | WIN0547.C21_N18 | 110385323 | Cabernet Sauvignon | Flower, leaf and root | Flower, pre-anthesis; leaf, fully expanded; root, produced by air-layering |
|  |  |  | CGF1000815_B04 | 33408694 | Cabernet Sauvignon | Stem | Pre-bloom (10-11 days before bloom) |
|  |  |  | CSECS210D08_5_PREn0028 | 87585891 | Cabernet Sauvignon | Fruit | 28 - modified E-L system |
|  |  |  | CGF1000816_B04 | 33408776 | Cabernet Sauvignon | Stem | Pre-bloom (10-11 days before bloom) |
|  |  |  | CGF1000818_B04 | 33408943 | Cabernet Sauvignon | Stem | Pre-bloom (10-11 days before bloom) |
|  |  |  | VV_PEd01d03.g1 | 156737420 | Perlette | Bud | Young |
|  |  |  | WIN1136.C21_C06 | 110422942 | Muscat Hamburg | Berry | Anthesis flower to prior to veraison |
|  |  |  | WIN0561.C21_L14 | 110389582 | Cabernet Sauvignon | Flower, leaf and root | Flower, pre-anthesis; leaf, fully expanded; root, produced by air-layering |
|  |  |  | CAB20007_Ia_Ra_D05 | 33405820 | Cabernet Sauvignon | Flower | Bloom |
|  |  |  | CAB20007_Ia_Fa_D05 | 33405738 | Cabernet Sauvignon | Flower | Bloom |
|  |  |  | VVH013D11_740541 | 71861886 | Cabernet Sauvignon | Nectary of flowers | 25 - modified E-L system |
|  |  |  | FAMU_USDA_FP_6740 | 51580881 | Vitis shuttleworthii | Entire tendril, leaves, bud, flowers | At blooming |
|  |  |  | WIN0519.C21_E07 | 110375326 | Cabernet Sauvignon | Flower, leaf and root | Flower, pre-anthesis; leaf, fully expanded; root, produced by air-layering |
|  |  |  | S1G01109 | 110697990 | Thompson-seedless | Fruit and flower |  |
| *VvWBC20* | *VvABCG20* | GSVIVT01025230001 | VVL150B04_700400 | 71890332 | Cabernet Sauvignon | Fruit with seeds removed | Mixed 36-38 - modified E-L system (Brix > 15) |
|  |  |  | CGF1000655_A08 | 33406999 | Cabernet Sauvignon | Stem | Pre-bloom (10-11 days before bloom) |
| *VvWBC21* | *VvABCG21* | GSVIVT01025582001 | WIN0551.C21_A13 | 122690313 | Cabernet Sauvignon | Flower, leaf and root | Flower, pre-anthesis; leaf, fully expanded; root, producedby air-layering |
|  |  |  | WIN0573.C21_H09 | 122690694 | Cabernet Sauvignon | Flower, leaf and root | Flower, pre-anthesis; leaf, fully expanded; root, producedby air-layering |
|  |  |  | FAMU_USDA_FP_2131 | 51576272 | Vitis shuttleworthii | Entire tendril, leaves, bud, flowers | At blooming |
|  |  |  | sT7aVVM016F20076 | 161717891 | Cabernet Sauvignon | Roots | 10 cm high plants grown in Magenta boxes |
|  |  |  | sT7aVVM_AER3G04 | 161708350 | Cabernet Sauvignon | Roots | 10 cm high plants grown in Magenta boxes |
| *VvWBC22* | *VvABCG22* | GSVIVT01025712001 | CGF1000655_A08 | 33406999 | Cabernet Sauvignon | Stem | Pre-bloom (10-11 days before bloom) |
| *VvWBC23* | *VvABCG23* | GSVIVT01028809001 | WIN1120.C21_F10 | 110417838 | Muscat Hamburg | Berry | Anthesis flower to prior to veraison |
|  |  |  | sT7aVVM028E06027 | 161720678 | Cabernet Sauvignon | Roots | 10 cm high plants grown in Magenta boxes |
|  |  |  | WIN022.C21_C18 | 110364242 | Cabernet Sauvignon | Flower, leaf and root | Flower, pre-anthesis; leaf, fully expanded; root, producedby air-layering |
|  |  |  | sT7aVVM019C21093 | 161718003 | Cabernet Sauvignon | Roots | 10 cm high plants grown in Magenta boxes |
|  |  |  | EST 1289 | 22014086 | Shiraz | Fruit | Green stage |
|  |  |  | sT7aVVM_AER42D10 | 161711657 | Cabernet Sauvignon | Roots | 10 cm high plants grown in Magenta boxes |
|  |  |  | CSECS098B10_POSn0036 | 34416797 | Cabernet Sauvignon | Fruit with seeds removed | 32 - modified E-L system |
|  |  |  | sT7aVVM005N21083 | 161714895 | Cabernet Sauvignon | Roots | 10 cm high plants grown in Magenta boxes |
|  |  |  | sT7aVVM021G14057 | 161719990 | Cabernet Sauvignon | Roots | 10 cm high plants grown in Magenta boxes |
|  |  |  | C2B03715 | 110687392 | Carmenere | Bud cluster |  |
|  |  |  | WIN1139.C21_H09 | 110424047 | Muscat Hamburg | Berry | Anthesis flower to prior to veraison |
|  |  |  | WIN0531.C21_G14 | 110379118 | Cabernet Sauvignon | Flower, leaf and root | Flower, pre-anthesis; leaf, fully expanded; root, producedby air-layering |
|  |  |  | S4B04965 | 110706814 | Thompson-seedless | Fruit | Fruits 2-3 mm |
|  |  |  | CAB2SG0005_IIIaR_G05 | 28969543 | Cabernet Sauvignon | Berry | Varaison |
|  |  |  | CAB2SG0005_IIIaF_G05 | 28969458 | Cabernet Sauvignon | Berry | Varaison |
|  |  |  | INFIO01_000748 | 37190570 | Regent | Inflorescence | Young inflorescence before flowering |
|  |  |  | USDA_FP_131710 | 47090354 | Vitis shuttleworthii | Entire tendril, leaves, bud, flowers | At blooming |
|  |  |  | WIN0558.C21_P19 | 110388741 | Cabernet Sauvignon | Flower, leaf and root | Flower, pre-anthesis; leaf, fully expanded; root, producedby air-layering |
|  |  |  | S2B11294 | 110700954 | Thompson-seedless | Bud |  |
|  |  |  | C3B01846 | 110693867 | Carmenere | Cluster | Cluster 4 cm |
|  |  |  | WIN028.TB24.1_A23 | 110361705 | Cabernet Sauvignon | Flower, leaf and root | Flower, pre-anthesis; leaf, fully expanded; root, producedby air-layering |
|  |  |  | FAMU_USDA_FP_2927 | 51577068 | Vitis shuttleworthii | Entire tendril, leaves, bud, flowers | At blooming |
|  |  |  | sT7aVVM_AER39B01 | 161711182 | Cabernet Sauvignon | Roots | 10 cm high plants grown in Magenta boxes |
|  |  |  | sT7aVVM_AER12F12 | 161709104 | Cabernet Sauvignon | Roots | 10 cm high plants grown in Magenta boxes |
| *VvWBC24* | *VvABCG24* | GSVIVT01031516001 | sT7aVVM003H07026 | 161712662 | Cabernet Sauvignon | Roots | 10 cm high plants grown in Magenta boxes |
|  |  |  | L8_67_L_TC_P7-H03-SP6.ab1 1 255 | 134031009 | Vitis arizonica x Vitis rupestris | Leaf | Vegetative stage control |
|  |  |  | VVD035B07_347765 | 30133227 | Chardonnay | Berries | Mixed; 8, 9, 11, 13, 15, 16 weeks daf |
|  |  |  | WIN0419.C21_I04 | 122689144 | Cabernet Sauvignon | Pericarp | Fruit set to maturity |
| *VvWBC25* | *VvABCG25* | GSVIVT01031528001 | WIN114.C21_F20 | 110414463 | Muscat Hamburg | Berry | Anthesis flower to prior to veraison |
|  |  |  | WIN057.C21_D24 | 110382159 | Cabernet Sauvignon | Flower, leaf and root | Flower, pre-anthesis; leaf, fully expanded; root, producedby air-layering |
|  |  |  | WIN0523.C21_J02 | 110376665 | Cabernet Sauvignon | Flower, leaf and root | Flower, pre-anthesis; leaf, fully expanded; root, producedby air-layering |
|  |  |  | WIN052.C21_N13 | 110371153 | Cabernet Sauvignon | Flower, leaf and root | Flower, pre-anthesis; leaf, fully expanded; root, producedby air-layering |
|  |  |  | WIN0563.C21_A17 | 110389990 | Cabernet Sauvignon | Flower, leaf and root | Flower, pre-anthesis; leaf, fully expanded; root, producedby air-layering |
|  |  |  | RR890915N0004_IIa_Fa_H04 | 33396247 | Vitis hybrid cultivar | Leaf |  |
|  |  |  | WIN0519.C21_E07 | 110375326 | Cabernet Sauvignon | Flower, leaf and root | Flower, pre-anthesis; leaf, fully expanded; root, producedby air-layering |
|  |  |  | WIN0211.TB24_B10 | 110362547 | Cabernet Sauvignon | Flower, leaf and root | Flower, pre-anthesis; leaf, fully expanded; root, producedby air-layering |
|  |  |  | WIN029.TB24_A21 | 110361994 | Cabernet Sauvignon | Flower, leaf and root | Flower, pre-anthesis; leaf, fully expanded; root, producedby air-layering |
|  |  |  | FAMU_USDA_FP_4520 | 51578661 | Vitis shuttleworthii | Entire tendril, leaves, bud, flowers | At blooming |
| *VvWBC26* | *VvABCG26* | GSVIVT01031529001 | WIN0419.C21_J09 | 110369712 | Cabernet Sauvignon | Pricarp | Fruit set to maturity |
|  |  |  | WIN1140.C21_F19 | 110424354 | Muscat Hamburg | Berry | Anthesis flower to prior to veraison |
|  |  |  | CSECS067B08_PREu0032 | 34363095 | Cabernet Sauvignon | Fruit with seeds removed | 32 - modified E-L system |
|  |  |  | VVD114B10_371225 | 30131443 | Chardonnay | Berries | Mixed; 8, 9, 11, 13, 15, 16 weeks daf |
|  |  |  | VVD173D09_378017 | 30124714 | Chardonnay | Berries | Mixed; 8, 9, 11, 13, 15, 16 weeks daf |
|  |  |  | WIN1154.C21_L16 | 110428778 | Muscat Hamburg | Berry | Anthesis flower to prior to veraison |
|  |  |  | VVD123H05_368765 | 30130213 | Chardonnay | Berries | Mixed; 8, 9, 11, 13, 15, 16 weeks daf |
|  |  |  | WIN0211.TB24_B10 | 110362547 | Cabernet Sauvignon | Flower, leaf and root | Flower, pre-anthesis; leaf, fully expanded; root, producedby air-layering |
|  |  |  | VVI059F12_591728 | 71871889 | Cabernet Sauvignon | Inflorescence including flowers | 12 - modified E-L system |
|  |  |  | FAMU_USDA_FP_4520 | 51578661 | Vitis shuttleworthii | Entire tendril, leaves, bud, flowers | At blooming |
|  |  |  | WIN027.TB24.1_E02 | 110361478 | Cabernet Sauvignon | Flower, leaf and root | Flower, pre-anthesis; leaf, fully expanded; root, producedby air-layering |
|  |  |  | S1G01109 | 110697990 | Thompson-seedless | Fruit and Flower |  |
|  |  |  | CAB20007_Ia_Fa_D05 | 33405738 | Cabernet Sauvignon | Flower | Bloom |
|  |  |  | CAB20007_Ia_Ra_D05 | 33405820 | Cabernet Sauvignon | Flower | Bloom |
|  |  |  | WIN0519.C21_E07 | 110375326 | Cabernet Sauvignon | Flower, leaf and root | Flower, pre-anthesis; leaf, fully expanded; root, producedby air-layering |
| *VvWBC27* | *VvABCG27* | GSVIVT01032625001 | FAMU_USDA_FP_8461 | 51582602 | Vitis shuttleworthii | Entire tendril, leaves, bud, flowers | At blooming |
|  |  |  | FAMU_USDA_FP_3579 | 51577720 | Vitis shuttleworthii | Entire tendril, leaves, bud, flowers | At blooming |
|  |  |  | FAMU_USDA_FP_2131 | 51576272 | Vitis shuttleworthii | Entire tendril, leaves, bud, flowers | At blooming |
|  |  |  | FAMU_USDA_FP_6693 | 51580834 | Vitis shuttleworthii | Entire tendril, leaves, bud, flowers | At blooming |
|  |  |  | USDA_FP_131986 | 47090630 | Vitis shuttleworthii | Entire tendril, leaves, bud, flowers | At blooming |
| *VvWBC28* | *VvABCG28* | GSVIVT01034463001 | sT7aVVM011B10047 | 161719301 | Cabernet Sauvignon | Roots | 10 cm high plants grown in Magenta boxes |
|  |  |  | sT7aVVM017J10039 | 161719578 | Cabernet Sauvignon | Roots | 10 cm high plants grown in Magenta boxes |
| *VvWBC29* | *VvABCG29* | GSVIVT01036869001 | EST 108 | 56408147 | Ugni Blanc | Fruit | Green stage |
|  |  |  | CAbud0004_IIIF_C08 | 34543769 | Cabernet Sauvignon | Bud | Pre-bloom (10-11 days before bloom) |
|  |  |  | SCB00162 | 110729397 | Thompson-seedless | Inflorescence |  |
|  |  |  | sT7aVVM018H10041 | 161718789 | Cabernet Sauvignon | Roots | 10 cm high plants grown in Magenta boxes |
|  |  |  | CAbud0005_IVF_E08 | 34544801 | Cabernet Sauvignon | Bud | Pre-bloom (10-11 days before bloom) |
|  |  |  | CAP0005_IVF_E08 | 34549718 | Cabernet Sauvignon | Petiole | Onset of Veraison (berry softening) |
| *VvWBC30* | *VvABCG30* | GSVIVT01037274001 | WIN028.TB24.1_A23 | 110361705 | Cabernet Sauvignon | Flower, leaf and root | Flower, pre-anthesis; leaf, fully expanded; root, producedby air-layering |
| *VvPDR1* | *VvABCG31* | GSVIVT01015456001 | VVI025G06_586946 | 71869498 | Cabernet Sauvignon | Inflorescence including flowers | 12 - modified E-L system |
|  |  |  | VVI066H07_592904 | 71872477 | Cabernet Sauvignon | Inflorescence including flowers | 12 - modified E-L system |
|  |  |  | FAMU_USDA_FP_7182 | 51581323 | Vitis shuttleworthii | Entire tendril, leaves, bud, flowers | At blooming |
|  |  |  | sT7aVVM015F06027 | 161717623 | Cabernet Sauvignon | Roots | 10 cm high plants grown in Magenta boxes |
|  |  |  | WIN058.C21_K05 | 110374394 | Cabernet Sauvignon | Flower, Leaf and root | Flower, pre-anthesis; leaf, fully expanded; root, produced by air-layerin |
|  |  |  | WIN0574.C21_D24 | 110391620 | Cabernet Sauvignon | Flower, Leaf and root | Flower, pre-anthesis; leaf, fully expanded; root, produced by air-layerin |
|  |  |  | VV_PEb04e11.b1 | 156728750 | Perlette | Bud | Mature |
|  |  |  | VV_PEb04e11.g1 | 156732345 | Perlette | Bud | Mature |
|  |  |  | CAB10004_IIIa_Fa_E08 | 30253080 | Cabernet Sauvignon | Flower, pre-bloom | Pre-bloom |
|  |  |  | VV_PEb09f09.b1 | 156729588 | Perlette | Bud | Mature |
|  |  |  | WIN117.C21_O11 | 110429813 | Muscat Hamburg | Berry | Anthesis flower to prior to veraison |
|  |  |  | FAMU_USDA_FP_7993 | 51582134 | Vitis shuttleworthii | Entire tendril, leaves, bud, flowers | At blooming |
|  |  |  | WIN117.C21_H18 | 110429684 | Muscat Hamburg | Berry | Anthesis flower to prior to veraison |
|  |  |  | WIN0210.TB24.1_D15 | 110362322 | Cabernet Sauvignon | Flower, Leaf and root | Flower, pre-anthesis; leaf, fully expanded; root, produced by air-layerin |
|  |  |  | SCB08071 | 110733249 | Thompson-seedless | Inflorescence | Inflorescence with GA3 |
|  |  |  | VVI080E03_595100 | 71873575 | Cabernet Sauvignon | Inflorescence including flowers | 12 - modified E-L system |
| *VvPDR2* | *VvABCG32* | GSVIVT01015461001 | CA12EI303IIFb_A07 | 26265269 | Cabernet Sauvignon | Leaf | Mid-season leaf material |
|  |  |  | WIN1144.C21_L15 | 110425792 | Muscat Hamburg | Berry | Anthesis flower to prior to veraison |
|  |  |  | sT7aVVM015B11048 | 161716494 | Cabernet Sauvignon | Roots | 10 cm high plants grown in Magenta boxes |
|  |  |  | sT7aVVM018B11048 | 161716735 | Cabernet Sauvignon | Roots | 10 cm high plants grown in Magenta boxes |
|  |  |  | sT7aVVM015F06027 | 161717623 | Cabernet Sauvignon | Roots | 10 cm high plants grown in Magenta boxes |
|  |  |  | sT7aVVM004I19072 | 161713935 | Cabernet Sauvignon | Roots | 10 cm high plants grown in Magenta boxes |
|  |  |  | S9B03542 | 110722533 | Thompson-seedless | Berry | Ripening berries |
| *VvPDR3* | *VvABCG33* | GSVIVT01016991001 | sT7aVVM015K23086 | 161719427 | Cabernet Sauvignon | Roots | 10 cm high plants grown in Magenta boxes |
|  |  |  | sT7aVVM027K14053 | 161721191 | Cabernet Sauvignon | Roots | 10 cm high plants grown in Magenta boxes |
|  |  |  | sT7aVVM015F06027 | 161717623 | Cabernet Sauvignon | Roots | 10 cm high plants grown in Magenta boxes |
|  |  |  | EST 12656 | 32457821 | Chardonnay | Fruit pedicle | Green stage |
|  |  |  | V_PEb04e11.b1 | 156728750 | Perlette | Bud | Mature |
| *VvPDR4* | *VvABCG34* | GSVIVT01016992001 | sT7aVVM027K14053 | 161721191 | Cabernet Sauvignon | Roots | 10 cm high plants grown in Magenta boxes |
|  |  |  | sT7aVVM017D07030 | 161717628 | Cabernet Sauvignon | Roots | 10 cm high plants grown in Magenta boxes |
|  |  |  | FAMU_USDA_FP_1880 | 51576021 | Vitis shuttleworthii | Entire tendril, leaves, bud, flowers | At blooming |
|  |  |  | VV_PEb09f09.b1 | 156729588 | Perlette | Bud | Mature |
|  |  |  | VV_PEb09f09.g1 | 156732493 | Perlette | Bud | Mature |
| *VvPDR5* | *VvABCG35* | GSVIVT01016993001 | sT7aVVM015K23086 | 161719427 | Cabernet Sauvignon | Roots | 10 cm high plants grown in Magenta boxes |
|  |  |  | EST 12656 | 32457821 | Chardonnay | Fruit pedicle | Green stage |
| *VvPDR6* | *VvABCG36* | GSVIVT01016998001 | sT7aVVM027K14053 | 161721191 | Cabernet Sauvignon | Roots | 10 cm high plants grown in Magenta boxes |
|  |  |  | sT7aVVM015F06027 | 161717623 | Cabernet Sauvignon | Roots | 10 cm high plants grown in Magenta boxes |
|  |  |  | sT7aVVM015K23086 | 161719427 | Cabernet Sauvignon | Roots | 10 cm high plants grown in Magenta boxes |
|  |  |  | FAMU_USDA_FP_1880 | 51576021 | Vitis shuttleworthii | Entire tendril, leaves, bud, flowers | At blooming |
|  |  |  | CAB10004_IIIa_Fa_E08 | 30253080 | Cabernet Sauvignon | Flower, pre-bloom | Pre-bloom |
|  |  |  | CAP0004_IIIF_H12 | 34548882 | Cabernet Sauvignon | Petiole | Onset of veraison (berry softening) |
|  |  |  | EST 12656 | 32457821 | Chardonnay | Fruit pedicle | Green stage |
|  |  |  | VV_PEb14g03.g1 | 156730414 | Perlette | Bud | Mature |
| *VvPDR7* | *VvABCG37* | GSVIVT01016999001 | sT7aVVM017D07030 | 161717628 | Cabernet Sauvignon | Roots | 10 cm high plants grown in Magenta boxes |
|  |  |  | sT7aVV01002X1F10 | 161721127 | Cabernet Sauvignon | Roots | 10 cm high plants grown in Magenta boxes |
|  |  |  | sT7aVVM027K14053 | 161721191 | Cabernet Sauvignon | Roots | 10 cm high plants grown in Magenta boxes |
|  |  |  | sT7aVVM004I19072 | 161713935 | Cabernet Sauvignon | Roots | 10 cm high plants grown in Magenta boxes |
|  |  |  | sT7aVVM015K23086 | 161719427 | Cabernet Sauvignon | Roots | 10 cm high plants grown in Magenta boxes |
|  |  |  | sT7aVVM015F06027 | 161717623 | Cabernet Sauvignon | Roots | 10 cm high plants grown in Magenta boxes |
|  |  |  | FAMU_USDA_FP_1880 | 51576021 | Vitis shuttleworthii | Entire tendril, leaves, bud, flowers | At blooming |
|  |  |  | VV_PEb09f09.b1 | 156729588 | Perlette | Bud | Mature |
|  |  |  | EST 12656 | 32457821 | Chardonnay | Fruit pedicle | Green stage |
| *VvPDR8* | *VvABCG38* | GSVIVT01017184001 | VV_PEb11g06.b1 | 156729932 | Perlette | Bud | Mature |
|  |  |  | CAB10004_IIIa_Fa_E08 | 30253080 | Cabernet Sauvignon | Flower, pre-bloom | Pre-bloom |
|  |  |  | CAP0004_IIIF_H12 | 34548882 | Cabernet Sauvignon | Petiole | Onset of veraison (berry softening) |
|  |  |  | VV_PEb11g06.g1 | 156729933 | Perlette | Bud | Mature |
|  |  |  | sT7aVVM004I19072 | 161713935 | Cabernet Sauvignon | Roots | 10 cm high plants grown in Magenta boxes |
|  |  |  | VVA018A12_54179 | 18459064 | Chardonnay | Leaf | Juvenile and adult |
|  |  |  | sT7aVVM004I19072 | 161713935 | Cabernet Sauvignon | Roots | 10 cm high plants grown in Magenta boxes |
|  |  |  | VVL138C09_698350 | 71889307 | Cabernet Sauvignon | Fruit with seeds removed | Mixed 36-38 - modified E-L system (Brix > 15) |
|  |  |  | VVL116F05_694590 | 71887427 | Cabernet Sauvignon | Fruit with seeds removed | Mixed 36-38 - modified E-L system (Brix > 15) |
|  |  |  | C3B02236 | 110694286 | Carmenere | Cluster | Clusters 4 cm |
|  |  |  | WIN1149.C21_L07 | 110427444 | Muscat Hamburg | Berry | Anthesis flower to prior to veraison |
|  |  |  | S9B03542 | 110722533 | Thompson-seedless | Berry | Ripening berries |
|  |  |  | VVL118G09_694978 | 71887621 | Cabernet Sauvignon | Fruit with seeds removed | Mixed 36-38 - modified E-L system (Brix > 15) |
|  |  |  | VV_PEb14g03.b1 | 156730421 | Perlette | Bud | Mature |
|  |  |  | VVG008B07_752925 | 71854950 | Cabernet Sauvignon | Cell suspension culture | |
|  |  |  | WIN022.C21_D13 | 110360510 | Cabernet Sauvignon | Flower, leaf and root | Flower, pre-anthesis; leaf, fully expanded; root, produced by air-layerin |
|  |  |  | VVL137D11_698200 | 71889232 | Cabernet Sauvignon | Fruit with seeds removed | Mixed 36-38 - modified E-L system (Brix > 15) |
|  |  |  | S1G03478 | 110699298 | Thompson-seedless | Fruit and flower |  |
|  |  |  | VV_PEa18d05.b1 | 156726918 | Perlette | Bud | Mature |
|  |  |  | sT7aVVM015F06027 | 161717623 | Cabernet Sauvignon | Roots | 10 cm high plants grown in Magenta boxes |
|  |  |  | sT7aVV01002X1F10 | 161721127 | Cabernet Sauvignon | Roots | 10 cm high plants grown in Magenta boxes |
|  |  |  | sT7aVVM002M15052 | 161712783 | Cabernet Sauvignon | Roots | 10 cm high plants grown in Magenta boxes |
|  |  |  | sT7aVVM_AER28G09 | 161707375 | Cabernet Sauvignon | Roots | 10 cm high plants grown in Magenta boxes |
|  |  |  | sT7aVVM_AER28G08 | 161711104 | Cabernet Sauvignon | Roots | 10 cm high plants grown in Magenta boxes |
|  |  |  | VV_PEa16f04.g1 | 156726816 | Perlette | Bud | Mature |
|  |  |  | VV_PEa25b08.g1 | 156723754 | Perlette | Bud | Mature |
|  |  |  | VV_PEa015c09.b1 | 156724508 | Perlette | Bud | Mature |
|  |  |  | S1G02802 | 110699740 | Thompson-seedless | Fruit and flower |  |
|  |  |  | WIN1144.C21_L15 | 110425792 | Muscat Hamburg | Berry | Anthesis flower to prior to veraison |
|  |  |  | CGF1000665_A04 | 33406125 | Cabernet Sauvignon | Stem | Pre-bloom (10-11 days before bloom) |
| *VvPDR9* | *VvABCG39* | GSVIVT01017185001 | sT7aVVM015F06027 | 161717623 | Cabernet Sauvignon | Roots | 10 cm high plants grown in Magenta boxes |
|  |  |  | sT7aVVM004I19072 | 161713935 | Cabernet Sauvignon | Roots | 10 cm high plants grown in Magenta boxes |
|  |  |  | sT7aVVM_AER28G09 | 161707375 | Cabernet Sauvignon | Roots | 10 cm high plants grown in Magenta boxes |
|  |  |  | sT7aVVM002M15052 | 161712783 | Cabernet Sauvignon | Roots | 10 cm high plants grown in Magenta boxes |
|  |  |  | sT7aVVM_AER28G08 | 161711104 | Cabernet Sauvignon | Roots | 10 cm high plants grown in Magenta boxes |
|  |  |  | VVG008B07_752925 | 71854950 | Cabernet Sauvignon | Cell suspension culture |  |
|  |  |  | VV_PEa015c09.b1 | 156724508 | Perlette | Bud | Mature |
|  |  |  | VV_PEa18d05.g1 | 156726910 | Perlette | Bud | Mature |
|  |  |  | VV_PEb11g06.b1 | 156729932 | Perlette | Bud | Mature |
|  |  |  | VV_PEa013g05.b1 | 156728155 | Perlette | Bud | Mature |
|  |  |  | VV_PEa13g05.g1 | 156726584 | Perlette | Bud | Mature |
|  |  |  | VV_PEb11g06.g1 | 156729933 | Perlette | Bud | Mature |
|  |  |  | C2B01707 | 110686591 | Carmenere | Bud - cluster |  |
|  |  |  | WIN1144.C21_L15 | 110425792 | Muscat Hamburg | Berry | Anthesis flower to prior to veraison |
|  |  |  | CGF1000665_A04 | 33406125 | Cabernet Sauvignon | Stem | Pre-bloom (10-11 days before bloom) |
|  |  |  | EST 7326 | 22015257 | Ugni Blanc | Fruit | Veraison stage |
|  |  |  | C3B02236 | 110694286 | Carmenere | Cluster | Clusters 4 cm |
|  |  |  | VVH060G10_749061 | 71861652 | Cabernet Sauvignon | Nectary of flowers | 25 - modified E-L system |
|  |  |  | S9B03542 | 110722533 | Thompson-seedless | Berry | Ripening berries |
|  |  |  | CAB10004_IIIa_Fa_E08 | 30253080 | Cabernet Sauvignon | Flower, pre-bloom | Pre-bloom |
|  |  |  | VVL057H07_684512 | 71882388 | Cabernet Sauvignon | Fruit with seeds removed | Mixed 36-38 - modified E-L system (Brix > 15) |
|  |  |  | S1G03478 | 110699298 | Thompson-seedless | Fruit and flower |  |
|  |  |  | CAP0004_IIIF_H12 | 34548882 | Cabernet Sauvignon | Petiole | Onset of veraison (berry softening) |
| *VvPDR10* | *VvABCG40* | GSVIVT01017187001 | sT7aVVM_AER28G09 | 161707375 | Cabernet Sauvignon | Roots | 10 cm high plants grown in Magenta boxes |
|  |  |  | sT7aVVM002M15052 | 161712783 | Cabernet Sauvignon | Roots | 10 cm high plants grown in Magenta boxes |
|  |  |  | sT7aVVM_AER28G08 | 161711104 | Cabernet Sauvignon | Roots | 10 cm high plants grown in Magenta boxes |
|  |  |  | sT7aVVM004I19072 | 161713935 | Cabernet Sauvignon | Roots | 10 cm high plants grown in Magenta boxes |
|  |  |  | sT7aVV01002X1F10 | 161721127 | Cabernet Sauvignon | Roots | 10 cm high plants grown in Magenta boxes |
|  |  |  | sT7aVVM015F06027 | 161717623 | Cabernet Sauvignon | Roots | 10 cm high plants grown in Magenta boxes |
|  |  |  | VV_PEa25b08.g1 | 156723754 | Perlette | Bud | Mature |
|  |  |  | VV_PEa25b08.b1 | 156723753 | Perlette | Bud | Mature |
|  |  |  | VV_PEa013g05.b1 | 156728155 | Perlette | Bud | Mature |
|  |  |  | VV_PEa13g05.g1 | 156726584 | Perlette | Bud | Mature |
|  |  |  | CAP0004_IIIF_H12 | 34548882 | Cabernet Sauvignon | Petiole | Onset of veraison (berry softening) |
|  |  |  | S1G03478 | 110699298 | Thompson-seedless | Fruit and flower |  |
|  |  |  | WIN022.C21_D13 | 110360510 | Cabernet Sauvignon | Flower, leaf and root | Flower, pre-anthesis; leaf, fully expanded; root, produced by air-layerin |
|  |  |  | EST 7326 | 22015257 | Ugni Blanc | Fruit | Veraison stage |
|  |  |  | CAB10004_IIIa_Fa_E08 | 30253080 | Cabernet Sauvignon | Flower, pre-bloom | Pre-bloom |
|  |  |  | CAP0004_IIIF_H12 | 34548882 | Cabernet Sauvignon | Petiole | Onset of veraison (berry softening) |
|  |  |  | S1G03478 | 110699298 | Thompson-seedless | Fruit and flower |  |
|  |  |  | WIN022.C21_L18 | 110364296 | Cabernet Sauvignon | Flower, leaf and root | Flower, pre-anthesis; leaf, fully expanded; root, produced by air-layerin |
|  |  |  | WIN1144.C21_L15 | 110425792 | Muscat Hamburg | Berry | Anthesis flower to prior to veraison |
|  |  |  | VVL057H07_684512 | 71882388 | Cabernet Sauvignon | Fruit with seeds removed | Mixed 36-38 - modified E-L system (Brix > 15) |
|  |  |  | VRK350T7 | 48941441 | Vitis riparia | Bud | Paradormant |
|  |  |  | S9B03542 | 110722533 | Thompson-seedless | Berry | Ripening berries |
|  |  |  | C3B02236 | 110694286 | Carmenere | Cluster | Clusters 4 cm |
|  |  |  | S1G02802 | 110699740 | Thompson-seedless | Fruit and flower |  |
|  |  |  | VVH060G10_749061 | 71861652 | Cabernet Sauvignon | Nectary of flowers | 25 - modified E-L system |
|  |  |  | VVG008B07_752925 | 71854950 | Cabernet Sauvignon | Cell suspension culture | |
|  |  |  | C2B01707 | 110686591 | Carmenere | Bud - cluster |  |
|  |  |  | CGF1000665_A04 | 33406125 | Cabernet Sauvignon | Stem | Pre-bloom (10-11 days before bloom) |
| *VvPDR11* | *VvABCG41* | GSVIVT01017188001 | WIN022.C21_D13 | 110360510 | Cabernet Sauvignon | Flower, leaf and root | Flower, pre-anthesis; leaf, fully expanded; root, produced by air-layerin |
|  |  |  | VVG008B07_752925 | 71854950 | Cabernet Sauvignon | Cell suspension culture | |
|  |  |  | VRK350T7 | 48941441 | Vitis riparia | Bud | Paradormant |
|  |  |  | CAP0004_IIIF_H12 | 34548882 | Cabernet Sauvignon | Petiole | Onset of veraison (berry softening) |
|  |  |  | EST 7326 | 22015257 | Ugni Blanc | Fruit | Veraison stage |
|  |  |  | CAB10004_IIIa_Fa_E08 | 30253080 | Cabernet Sauvignon | Flower, pre-bloom | Pre-bloom |
|  |  |  | VVL144F01_699442 | 71889853 | Cabernet Sauvignon | Fruit with seeds removed | Mixed 36-38 - modified E-L system (Brix > 15) |
|  |  |  | CAP0004_IIIF_H12 | 34548882 | Cabernet Sauvignon | Petiole | Onset of veraison (berry softening) |
|  |  |  | VV_PEa25b08.g1 | 156723754 | Perlette | Bud | Mature |
|  |  |  | sT7aVVM015F06027 | 161717623 | Cabernet Sauvignon | Roots | 10 cm high plants grown in Magenta boxes |
|  |  |  | VV_PEa18d05.g1 | 156726910 | Perlette | Bud | Mature |
|  |  |  | VV_PEa18d05.b1 | 156726918 | Perlette | Bud | Mature |
|  |  |  | VV_PEa16f04.g1 | 156726816 | Perlette | Bud | Mature |
|  |  |  | S9B03542 | 110722533 | Thompson-seedless | Berry | Ripening berries |
|  |  |  | VVL138C09_698350 | 71889307 | Cabernet Sauvignon | Fruit with seeds removed | Mixed 36-38 - modified E-L system (Brix > 15) |
|  |  |  | VVH060G10_749061 | 71861652 | Cabernet Sauvignon | Nectary of flowers | 25 - modified E-L system |
|  |  |  | VV_PEa013g05.b1 | 156728155 | Perlette | Bud | Mature |
|  |  |  | VV_PEa016f04.b1 | 156723960 | Perlette | Bud | Mature |
|  |  |  | VV_PEa25b08.b1 | 156723753 | Perlette | Bud | Mature |
|  |  |  | VV_PEa13g05.g1 | 156726584 | Perlette | Bud | Mature |
|  |  |  | S1G03478 | 110699298 | Thompson-seedless | Fruit and flower |  |
|  |  |  | sT7aVVM004I19072 | 161713935 | Cabernet Sauvignon | Roots | 10 cm high plants grown in Magenta boxes |
|  |  |  | sT7aVV01002X1F10 | 161721127 | Cabernet Sauvignon | Roots | 10 cm high plants grown in Magenta boxes |
|  |  |  | sT7aVVM_AER28G09 | 161707375 | Cabernet Sauvignon | Roots | 10 cm high plants grown in Magenta boxes |
|  |  |  | sT7aVVM002M15052 | 161712783 | Cabernet Sauvignon | Roots | 10 cm high plants grown in Magenta boxes |
|  |  |  | sT7aVVM_AER28G08 | 161711104 | Cabernet Sauvignon | Roots | 10 cm high plants grown in Magenta boxes |
|  |  |  | WIN022.C21_D13 | 110360510 | Cabernet Sauvignon | Flower, leaf and root | Flower, pre-anthesis; leaf, fully expanded; root, produced by air-layerin |
|  |  |  | WIN022.C21_L18 | 110364296 | Cabernet Sauvignon | Flower, leaf and root | Flower, pre-anthesis; leaf, fully expanded; root, produced by air-layerin |
|  |  |  | VV_PEb11g06.g1 | 156729933 | Perlette | Bud | Mature |
|  |  |  | VV_PEb14g03.g1 | 156730414 | Perlette | Bud | Mature |
|  |  |  | VV_PEa015c09.b1 | 156724508 | Perlette | Bud | Mature |
|  |  |  | C2B01707 | 110686591 | Carmenere | Bud - cluster |  |
|  |  |  | C3B02236 | 110694286 | Carmenere | Cluster | Clusters 4 cm |
|  |  |  | WIN1144.C21_L15 | 110425792 | Muscat Hamburg | Berry | Anthesis flower to prior to veraison |
| *VvPDR12* | *VvABCG42* | GSVIVT01017196001 | FOGLI02_000434 | 37187080 | Pinot Noir | Leaf | Juvenile |
|  |  |  | VVG008B07_752925 | 71854950 | Cabernet Sauvignon | Cell suspension culture | |
|  |  |  | VRK350T7 | 48941441 | Vitis riparia | Bud | Paradormant |
|  |  |  | VVL057H07_684512 | 71882388 | Cabernet Sauvignon | Fruit with seeds removed | Mixed 36-38 - modified E-L system (Brix > 15) |
|  |  |  | VV_PEa15c09.g1 | 156726711 | Perlette | Bud | Mature |
|  |  |  | VV_PEa13g05.g1 | 156726584 | Perlette | Bud | Mature |
|  |  |  | VV_PEa013g05.b1 | 156728155 | Perlette | Bud | Mature |
|  |  |  | VV_PEa18d05.b1 | 156726918 | Perlette | Bud | Mature |
|  |  |  | VV_PEa16f04.g1 | 156726816 | Perlette | Bud | Mature |
|  |  |  | S1G03478 | 110699298 | Thompson-seedless | Fruit and flower |  |
|  |  |  | S9B03542 | 110722533 | Thompson-seedless | Berry | Ripening berries |
|  |  |  | CAB10004_IIIa_Fa_E08 | 30253080 | Cabernet Sauvignon | Flower, pre-bloom | Pre-bloom |
|  |  |  | sT7aVVM015F06027 | 161717623 | Cabernet Sauvignon | Roots | 10 cm high plants grown in Magenta boxes |
|  |  |  | sT7aVVM004I19072 | 161713935 | Cabernet Sauvignon | Roots | 10 cm high plants grown in Magenta boxes |
|  |  |  | sT7aVVM015F06027 | 161717623 | Cabernet Sauvignon | Roots | 10 cm high plants grown in Magenta boxes |
|  |  |  | sT7aVVM_AER28G08 | 161711104 | Cabernet Sauvignon | Roots | 10 cm high plants grown in Magenta boxes |
|  |  |  | sT7aVVM002M15052 | 161712783 | Cabernet Sauvignon | Roots | 10 cm high plants grown in Magenta boxes |
|  |  |  | sT7aVVM_AER28G09 | 161707375 | Cabernet Sauvignon | Roots | 10 cm high plants grown in Magenta boxes |
|  |  |  | VV_PEb11g06.b1 | 156729932 | Perlette | Bud | Mature |
|  |  |  | VV_PEa18d05.b1 | 156726918 | Perlette | Bud | Mature |
|  |  |  | VV_PEa25b08.b1 | 156723753 | Perlette | Bud | Mature |
|  |  |  | VV_PEa18d05.g1 | 156726910 | Perlette | Bud | Mature |
|  |  |  | VV_PEa25b08.g1 | 156723754 | Perlette | Bud | Mature |
|  |  |  | S1G03478 | 110699298 | Thompson-seedless | Fruit and flower |  |
|  |  |  | CAP0004_IIIR_H12 | 34548975 | Cabernet Sauvignon | Petiole | Onset of veraison (berry softening) |
|  |  |  | VVA018A12_54179 | 18459064 | Chardonnay | Leaf | Juvenile and adult |
|  |  |  | VVH060G10_749061 | 71861652 | Cabernet Sauvignon | Nectary of flowers | 25 - modified E-L system |
|  |  |  | WIN022.C21_L18 | 110364296 | Cabernet Sauvignon | Flower, leaf and root | Flower, pre-anthesis; leaf, fully expanded; root, produced by air-layerin |
|  |  |  | C3B02236 | 110694286 | Carmenere | Cluster | Clusters 4 cm |
|  |  |  | S1G02802 | 110699740 | Thompson-seedless | Fruit and flower |  |
|  |  |  | WIN1144.C21_L15 | 110425792 | Muscat Hamburg | Berry | Anthesis flower to prior to veraison |
|  |  |  | CGF1000665_A04 | 33406125 | Cabernet Sauvignon | Stem | Pre-bloom (10-11 days before bloom) |
|  |  |  | C2B01707 | 110686591 | Carmenere | Bud - cluster |  |
| *VvPDR13* | *VvABCG43* | GSVIVT01017198001 | VV_PEa13g05.g1 | 156726584 | Perlette | Bud | Mature |
|  |  |  | VV_PEa18d05.g1 | 156726910 | Perlette | Bud | Mature |
|  |  |  | CAB10004_IIIa_Fa_E08 | 30253080 | Cabernet Sauvignon | Flower, pre-bloom | Pre-bloom |
|  |  |  | CAP0004_IIIF_H12 | 34548882 | Cabernet Sauvignon | Petiole | Onset of veraison (berry softening) |
|  |  |  | VVG008B07_752925 | 71854950 | Cabernet Sauvignon | Cell suspension culture | |
|  |  |  | VRK350T7 | 48941441 | Vitis riparia | Bud | Paradormant |
|  |  |  | VVL144F01_699442 | 71889853 | Cabernet Sauvignon | Fruit with seeds removed | Mixed 36-38 - modified E-L system (Brix > 15) |
|  |  |  | sT7aVVM_AER88C07 | 161707636 | Cabernet Sauvignon | Roots | 10 cm high plants grown in Magenta boxes |
|  |  |  | sT7aVVM015F06027 | 161717623 | Cabernet Sauvignon | Roots | 10 cm high plants grown in Magenta boxes |
|  |  |  | VV_PEa18d05.b1 | 156726918 | Perlette | Bud | Mature |
|  |  |  | VV_PEa013g05.b1 | 156728155 | Perlette | Bud | Mature |
|  |  |  | VV_PEa25b08.b1 | 156723753 | Perlette | Bud | Mature |
|  |  |  | VV_PEa016f04.b1 | 156723960 | Perlette | Bud | Mature |
|  |  |  | VV_PEa16f04.g1 | 156726816 | Perlette | Bud | Mature |
|  |  |  | S1G03478 | 110699298 | Thompson-seedless | Fruit and flower |  |
|  |  |  | S9B03542 | 110722533 | Thompson-seedless | Berry | Ripening berries |
|  |  |  | VVH060G10_749061 | 71861652 | Cabernet Sauvignon | Nectary of flowers | 25 - modified E-L system |
|  |  |  | VVL138C09_698350 | 71889307 | Cabernet Sauvignon | Fruit with seeds removed | Mixed 36-38 - modified E-L system (Brix > 15) |
|  |  |  | VV_PEa25b08.g1 | 156723754 | Perlette | Bud | Mature |
|  |  |  | VV_PEb11g06.b1 | 156729932 | Perlette | Bud | Mature |
|  |  |  | VV_PEb14g03.b1 | 156730421 | Perlette | Bud | Mature |
|  |  |  | VV_PEa015c09.b1 | 156724508 | Perlette | Bud | Mature |
|  |  |  | sT7aVVM004I19072 | 161713935 | Cabernet Sauvignon | Roots | 10 cm high plants grown in Magenta boxes |
|  |  |  | sT7aVV01002X1F10 | 161721127 | Cabernet Sauvignon | Roots | 10 cm high plants grown in Magenta boxes |
|  |  |  | sT7aVVM_AER28G09 | 161707375 | Cabernet Sauvignon | Roots | 10 cm high plants grown in Magenta boxes |
|  |  |  | sT7aVVM002M15052 | 161712783 | Cabernet Sauvignon | Roots | 10 cm high plants grown in Magenta boxes |
|  |  |  | sT7aVVM_AER28G08 | 161711104 | Cabernet Sauvignon | Roots | 10 cm high plants grown in Magenta boxes |
|  |  |  | WIN1144.C21_L15 | 110425792 | Muscat Hamburg | Berry | Anthesis flower to prior to veraison |
|  |  |  | S1G02802 | 110699740 | Thompson-seedless | Fruit and flower |  |
|  |  |  | C3B02236 | 110694286 | Carmenere | Cluster | Clusters 4 cm |
|  |  |  | CGF1000665_A04 | 33406125 | Cabernet Sauvignon | Stem | Pre-bloom (10-11 days before bloom) |
|  |  |  | C2B01707 | 110686591 | Carmenere | Bud - cluster |  |
| *VvPDR14* | *VvABCG44* | GSVIVT01017201001 | S1G02802 | 110699740 | Thompson-seedless | Fruit and flower |  |
|  |  |  | CAB10004_IIIa_Fa_E08 | 30253080 | Cabernet Sauvignon | Flower, pre-bloom | Pre-bloom |
|  |  |  | CAP0004_IIIF_H12 | 34548882 | Cabernet Sauvignon | Petiole | Onset of veraison (berry softening) |
|  |  |  | WIN1144.C21_L15 | 110425792 | Muscat Hamburg | Berry | Anthesis flower to prior to veraison |
|  |  |  | WIN1149.C21_L07 | 110427444 | Muscat Hamburg | Berry | Anthesis flower to prior to veraison |
|  |  |  | CAP0004_IIIF_H12 | 34548882 | Cabernet Sauvignon | Petiole | Onset of veraison (berry softening) |
|  |  |  | S1G03478 | 110699298 | Thompson-seedless | Fruit and flower |  |
|  |  |  | sT7aVVM018B11048 | 161716735 | Cabernet Sauvignon | Roots | 10 cm high plants grown in Magenta boxes |
|  |  |  | sT7aVVM015F06027 | 161717623 | Cabernet Sauvignon | Roots | 10 cm high plants grown in Magenta boxes |
|  |  |  | VV_PEa13g05.g1 | 156726584 | Perlette | Bud | Mature |
|  |  |  | VV_PEa18d05.b1 | 156726918 | Perlette | Bud | Mature |
|  |  |  | VV_PEa18d05.g1 | 156726910 | Perlette | Bud | Mature |
|  |  |  | VV_PEa16f04.g1 | 156726816 | Perlette | Bud | Mature |
|  |  |  | VV_PEa013g05.b1 | 156728155 | Perlette | Bud | Mature |
|  |  |  | VV_PEa25b08.b1 | 156723753 | Perlette | Bud | Mature |
|  |  |  | VV_PEa15c09.g1 | 156726711 | Perlette | Bud | Mature |
|  |  |  | VV_PEb11g06.g1 | 156729933 | Perlette | Bud | Mature |
|  |  |  | VV_PEa016f04.b1 | 156723960 | Perlette | Bud | Mature |
|  |  |  | VV_PEa015c09.b1 | 156724508 | Perlette | Bud | Mature |
|  |  |  | VVH060G10_749061 | 71861652 | Cabernet Sauvignon | Nectary of flowers | 25 - modified E-L system |
|  |  |  | VVG008B07_752925 | 71854950 | Cabernet Sauvignon | Cell suspension culture | |
|  |  |  | C2B01707 | 110686591 | Carmenere | Bud - cluster |  |
|  |  |  | C3B02236 | 110694286 | Carmenere | Cluster | Clusters 4 cm |
|  |  |  | S1G02802 | 110699740 | Thompson-seedless | Fruit and flower |  |
|  |  |  | S9B03542 | 110722533 | Thompson-seedless | Berry | Ripening berries |
|  |  |  | sT7aVVM004I19072 | 161713935 | Cabernet Sauvignon | Roots | 10 cm high plants grown in Magenta boxes |
|  |  |  | sT7aVV01002X1F10 | 161721127 | Cabernet Sauvignon | Roots | 10 cm high plants grown in Magenta boxes |
|  |  |  | sT7aVVM002M15052 | 161712783 | Cabernet Sauvignon | Roots | 10 cm high plants grown in Magenta boxes |
| *VvPDR15* | *VvABCG45* | GSVIVT01017202001 | CAP0004_IIIF_H12 | 34548882 | Cabernet Sauvignon | Petiole | Onset of veraison (berry softening) |
|  |  |  | WIN022.C21_L18 | 110364296 | Cabernet Sauvignon | Flower, leaf and root | Flower, pre-anthesis; leaf, fully expanded; root, produced by air-layerin |
|  |  |  | sT7aVVM004I19072 | 161713935 | Cabernet Sauvignon | Roots | 10 cm high plants grown in Magenta boxes |
|  |  |  | sT7aVVM_AER28G08 | 161711104 | Cabernet Sauvignon | Roots | 10 cm high plants grown in Magenta boxes |
|  |  |  | sT7aVVM002M15052 | 161712783 | Cabernet Sauvignon | Roots | 10 cm high plants grown in Magenta boxes |
|  |  |  | sT7aVVM_AER28G09 | 161707375 | Cabernet Sauvignon | Roots | 10 cm high plants grown in Magenta boxes |
|  |  |  | sT7aVV01002X1F10 | 161721127 | Cabernet Sauvignon | Roots | 10 cm high plants grown in Magenta boxes |
|  |  |  | sT7aVVM015F06027 | 161717623 | Cabernet Sauvignon | Roots | 10 cm high plants grown in Magenta boxes |
|  |  |  | VV_PEa013g05.b1 | 156728155 | Perlette | Bud | Mature |
|  |  |  | VV_PEa18d05.b1 | 156726918 | Perlette | Bud | Mature |
|  |  |  | VV_PEa15c09.g1 | 156726711 | Perlette | Bud | Mature |
|  |  |  | VV_PEa25b08.g1 | 156723754 | Perlette | Bud | Mature |
|  |  |  | VV_PEa25b08.b1 | 156723753 | Perlette | Bud | Mature |
|  |  |  | VV_PEa16f04.g1 | 156726816 | Perlette | Bud | Mature |
|  |  |  | VV_PEa016f04.b1 | 156723960 | Perlette | Bud | Mature |
|  |  |  | VV_PEa015c09.b1 | 156724508 | Perlette | Bud | Mature |
|  |  |  | WIN1149.C21_L07 | 110427444 | Muscat Hamburg | Berry | Anthesis flower to prior to veraison |
|  |  |  | WIN1144.C21_L15 | 110425792 | Muscat Hamburg | Berry | Anthesis flower to prior to veraison |
|  |  |  | S1G03478 | 110699298 | Thompson-seedless | Fruit and flower |  |
|  |  |  | S1G02802 | 110699740 | Thompson-seedless | Fruit and flower |  |
|  |  |  | VVL144F01_699442 | 71889853 | Cabernet Sauvignon | Fruit with seeds removed | Mixed 36-38 - modified E-L system (Brix > 15) |
|  |  |  | VVL138C09_698350 | 71889307 | Cabernet Sauvignon | Fruit with seeds removed | Mixed 36-38 - modified E-L system (Brix > 15) |
|  |  |  | CAB10004_IIIa_Fa_E08 | 30253080 | Cabernet Sauvignon | Flower, pre-bloom | Pre-bloom |
|  |  |  | WIN022.C21_L18 | 110364296 | Cabernet Sauvignon | Flower, leaf and root | Flower, pre-anthesis; leaf, fully expanded; root, produced by air-layering |
|  |  |  | S1G03478 | 110699298 | Thompson-seedless | Fruit and flower |  |
|  |  |  | VVH060G10_749061 | 71861652 | Cabernet Sauvignon | Nectary of flowers | 25 - modified E-L system |
|  |  |  | S9B03542 | 110722533 | Thompson-seedless | Berry | Ripening berries |
|  |  |  | VVG008B07_752925 | 71854950 | Cabernet Sauvignon | Cell suspension culture | |
|  |  |  | CGF1000665_A04 | 33406125 | Cabernet Sauvignon | Stem | Pre-bloom (10-11 days before bloom) |
|  |  |  | C2B01707 | 110686591 | Carmenere | Bud - cluster |  |
| *VvPDR15* | *VvABCG45* | GSVIVT01017204001 | sT7aVVM_AER28G08 | 161711104 | Cabernet Sauvignon | Roots | 10 cm high plants grown in Magenta boxes |
|  |  |  | sT7aVVM002M15052 | 161712783 | Cabernet Sauvignon | Roots | 10 cm high plants grown in Magenta boxes |
|  |  |  | sT7aVVM_AER28G09 | 161707375 | Cabernet Sauvignon | Roots | 10 cm high plants grown in Magenta boxes |
|  |  |  | sT7aVV01002X1F10 | 161721127 | Cabernet Sauvignon | Roots | 10 cm high plants grown in Magenta boxes |
|  |  |  | sT7aVVM004I19072 | 161713935 | Cabernet Sauvignon | Roots | 10 cm high plants grown in Magenta boxes |
|  |  |  | sT7aVVM015F06027 | 161717623 | Cabernet Sauvignon | Roots | 10 cm high plants grown in Magenta boxes |
|  |  |  | sT7aVVM027K14053 | 161721191 | Cabernet Sauvignon | Roots | 10 cm high plants grown in Magenta boxes |
|  |  |  | sT7aVVM027E13059 | 161720772 | Cabernet Sauvignon | Roots | 10 cm high plants grown in Magenta boxes |
|  |  |  | VV_PEa015c09.b1 | 156724508 | Perlette | Bud | Mature |
|  |  |  | VV_PEa13g05.g1 | 156726584 | Perlette | Bud | Mature |
|  |  |  | VV_PEa18d05.g1 | 156726910 | Perlette | Bud | Mature |
|  |  |  | VV_PEa16f04.g1 | 156726816 | Perlette | Bud | Mature |
|  |  |  | VV_PEa013g05.b1 | 156728155 | Perlette | Bud | Mature |
|  |  |  | VV_PEb11g06.b1 | 156729932 | Perlette | Bud | Mature |
|  |  |  | VV_PEa25b08.g1 | 156723754 | Perlette | Bud | Mature |
|  |  |  | VV_PEa25b08.b1 | 156723753 | Perlette | Bud | Mature |
|  |  |  | VV_PEa013g05.b1 | 156728155 | Perlette | Bud | Mature |
|  |  |  | VV_PEa16f04.g1 | 156726816 | Perlette | Bud | Mature |
|  |  |  | VV_PEa18d05.g1 | 156726910 | Perlette | Bud | Mature |
|  |  |  | VV_PEa18d05.b1 | 156726918 | Perlette | Bud | Mature |
|  |  |  | C2B01707 | 110686591 | Carmenere | Bud - cluster |  |
|  |  |  | WIN1144.C21_L15 | 110425792 | Muscat Hamburg | Berry | Anthesis flower to prior to veraison |
|  |  |  | CAP0004_IIIF_H12 | 34548882 | Cabernet Sauvignon | Petiole | Onset of veraison (berry softening) |
|  |  |  | CAB10004_IIIa_Fa_E08 | 30253080 | Cabernet Sauvignon | Flower, pre-bloom | Pre-bloom |
|  |  |  | VVL144F01_699442 | 71889853 | Cabernet Sauvignon | Fruit with seeds removed | Mixed 36-38 - modified E-L system (Brix > 15) |
|  |  |  | WIN022.C21_L18 | 110364296 | Cabernet Sauvignon | Flower, leaf and root | Flower, pre-anthesis; leaf, fully expanded; root, produced by air-layering |
|  |  |  | S1G03478 | 110699298 | Thompson-seedless | Fruit and flower |  |
|  |  |  | WIN1149.C21_L07 | 110427444 | Muscat Hamburg | Berry | Anthesis flower to prior to veraison |
|  |  |  | VVL138C09_698350 | 71889307 | Cabernet Sauvignon | Fruit with seeds removed | Mixed 36-38 - modified E-L system (Brix > 15) |
| *VvPDR16* | *VvABCG46* | GSVIVT01017204001 | sT7aVVM_AER28G08 | 161711104 | Cabernet Sauvignon | Roots | 10 cm high plants grown in Magenta boxes |
|  |  |  | C2B01707 | 110686591 | Carmenere | Bud cluster |  |
|  |  |  | sT7aVVM002M15052 | 161712783 | Cabernet Sauvignon | Roots | 10 cm high plants grown in Magenta boxes |
|  |  |  | S1G02802 | 110699740 | Thompson-seedless | Fruit and flower |  |
|  |  |  | VV_PEa18d05.g1 | 156726910 | Perlette | Bud | Mature |
|  |  |  | VV_PEa013g05.b1 | 156728155 | Perlette | Bud | Mature |
|  |  |  | VV_PEa016f04.b1 | 156723960 | Perlette | Bud | Mature |
|  |  |  | VV_PEa16f04.g1 | 156726816 | Perlette | Bud | Mature |
|  |  |  | EST 1376 | 22014173 | Shiraz | Fruit | Green stage |
|  |  |  | WIN0520.C21_N07 | 110375794 | Cabernet Sauvignon | Flower leaf and root | Flower, pre-anthesis; leaf, fully expanded; root, produced by air-layering |
|  |  |  | VV_PEb11g06.b1 | 156729932 | Perlette | Bud | Mature |
|  |  |  | VV_PEa15c09.g1 | 156726711 | Perlette | Bud | Mature |
|  |  |  | VVA018A12_54179 | 18459064 | Chardonnay | Leaf | Juvenile and adult |
|  |  |  | CAB70006_IIIaR_C06 | 30305419 | Cabernet Sauvignon | Berry | Post-Veraison, 18-19 brix |
|  |  |  | VV_PEa015c09.b1 | 156724508 | Perlette | Bud | Mature |
|  |  |  | VV_PEd19c04.b1 | 156738932 | Perlette | Bud | Mature |
|  |  |  | CAP0004_IIIR_H12 | 34548975 | Cabernet Sauvignon | Petiole | Onset of Veraison (berry softening) |
|  |  |  | VV_PEd19c04.g1 | 156738941 | Perlette | Bud | Mature |
|  |  |  | CA12EI204IIIR_B10 | 26266480 | Cabernet Sauvignon | Leaf | Mid-season leaf material |
|  |  |  | VV_PEb14g03.b1 | 156730421 | Perlette | Bud | Mature |
|  |  |  | S1G03478 | 110699298 | Thompson-seedless | Fruit and flower |  |
|  |  |  | CSECS001D03_PREu0032 | 34361553 | Cabernet Sauvignon | Fruit with seeds removed | 32 - modified E-L system |
|  |  |  | EST 12745 | 32457910 | Chardonnay | Fruit pedicle | Green stage |
|  |  |  | VVG008B07_752925 | 71854950 | Cabernet Sauvignon | Cell suspension culture | |
|  |  |  | CAB70006_IIIaF_C06 | 30305347 | Cabernet Sauvignon | Berry | Post-Veraison, 18-19 brix |
|  |  |  | EST 7326 | 22015257 | Ugni Blanc | Fruit | Veraison stage |
|  |  |  | WIN0413.C21_B21 | 110367408 | Cabernet Sauvignon | Pericarp | Fruit set to maturity |
|  |  |  | CGF1000665_A04 | 33406125 | Cabernet Sauvignon | Stem | Pre-bloom (10-11 days before bloom) |
|  |  |  | WIN022.C21_D13 | 110360510 | Cabernet Sauvignon | Flower, leaf and root | Flower, pre-anthesis; leaf, fully expanded; root, produced by air-layerin |
|  |  |  | sT7aVVM004I19072 | 161713935 | Cabernet Sauvignon | Roots | 10 cm high plants grown in Magenta boxes |
|  |  |  | VVD122G08_368583 | 30130122 | Chardonnay | Berries | Mixed; 8, 9, 11, 13, 15, 16 weeks daf |
|  |  |  | C3B02236 | 110694286 | Carmenere | Cluster |  |
|  |  |  | WIN1149.C21_L07 | 110427444 | Muscat Hamburg | Berry | Anthesis flower to prior to veraison |
|  |  |  | S9B03542 | 110722533 | Thompson-seedless | Berry |  |
|  |  |  | sT7aVVM018B11048 | 161716735 | Cabernet Sauvignon | Roots | 10 cm high plants grown in Magenta boxes |
|  |  |  | VVL138C09_698350 | 71889307 | Cabernet Sauvignon | Fruit with seeds removed | Mixed 36-38 - modified E-L system (Brix > 15) |
|  |  |  | VVH060G10_749061 | 71861652 | Cabernet Sauvignon | Nectary of flowers | 25 - modified E-L system |
|  |  |  | sT7aVVM015F06027 | 161717623 | Cabernet Sauvignon | Roots | 10 cm high plants grown in Magenta boxes |
|  |  |  | VVL057H07_684512 | 71882388 | Cabernet Sauvignon | Fruit with seeds removed | Mixed 36-38 - modified E-L system (Brix > 15) |
|  |  |  | CAP0004_IIIF_H12 | 34548882 | Cabernet Sauvignon | Petiole | Onset of Veraison (berry softening) |
|  |  |  | sT7aVVM_AER28G09 | 161707375 | Cabernet Sauvignon | Roots | 10 cm high plants grown in Magenta boxes |
|  |  |  | WIN1144.C21_L15 | 110425792 | Muscat Hamburg | Berry | Anthesis flower to prior to veraison |
|  |  |  | sT7aVV01002X1F10 | 161721127 | Cabernet Sauvignon | Roots | 10 cm high plants grown in Magenta boxes |
|  |  |  | C3B02236 | 110694286 | Carmenere | Cluster | Clusters 4 cm |
|  |  |  | VV_PEa13g05.g1 | 156726584 | Perlette | Bud | Mature |
|  |  |  | VV_PEa25b08.g1 | 156723754 | Perlette | Bud | Mature |
|  |  |  | VV_PEa25b08.b1 | 156723753 | Perlette | Bud | Mature |
|  |  |  | WIN022.C21_L18 | 110364296 | Cabernet Sauvignon | Flower, leaf and root | Flower, pre-anthesis; leaf, fully expanded; root, produced by air-layering |
|  |  |  | VV_PEa16f04.g1 | 156726816 | Perlette | Bud | Mature |
|  |  |  | VV_PEa18d05.b1 | 156726918 | Perlette | Bud | Mature |
|  |  |  | CAB10004_IIIa_Fa_E08 | 30253080 | Cabernet Sauvignon | Flower | Pre-bloom |
|  |  |  | sT7aVVM027K14053 | 161721191 | Cabernet Sauvignon | Roots | 10 cm high plants grown in Magenta boxes |
| *VvPDR17* | *VvABCG47* | GSVIVT01017676001 | EST 13001 | 32458166 | Chardonnay | Fruit pedicle | Green stage |
|  |  |  | VVH060G10_749061 | 71861652 | Cabernet Sauvignon | Nectary of flowers | 25 - modified E-L system |
|  |  |  | S1G03478 | 110699298 | Thompson-seedless | Fruit and flower |  |
| *VvPDR18* | *VvABCG48* | GSVIVT01024743001 | EST 7509 | 22015440 | Ugni Blanc | Fruit | Veraison stage |
|  |  |  | WIN0514.C21_F15 | 110372865 | Cabernet Sauvignon | Flower leaf and root | Flower, pre-anthesis; leaf, fully expanded; root, produced by air-layering |
|  |  |  | VV_PEa015c09.b1 | 156724508 | Perlette | Bud | Mature |
|  |  |  | CAP0004_IIIF_H12 | 34548882 | Cabernet Sauvignon | Petiole | Onset of Veraison (berry softening) |
|  |  |  | sT7aVVM004I19072 | 161713935 | Cabernet Sauvignon | Roots | 10 cm high plants grown in Magenta boxes |
|  |  |  | VV_PEb04e11.b1 | 156728750 | Perlette | Bud | Mature |
| *VvPDR19* | *VvABCG49* | GSVIVT01031314001 | VV_PEb04e11.b1 | 156728750 | Perlette | Bud | Mature |
|  |  |  | sT7aVVM004I19072 | 161713935 | Cabernet Sauvignon | Roots | 10 cm high plants grown in Magenta boxes |
| *VvPDR20* | *VvABCG50* | GSVIVT01031377001 | VVB180D05_414233 | 32272004 | Chardonnay | Leaf | Juvenile and adult |
|  |  |  | sT7aVVM015F06027 | 161717623 | Cabernet Sauvignon | Roots | 10 cm high plants grown in Magenta boxes |
|  |  |  | VVB072B06_334116 | 30324232 | Chardonnay | Leaf | Juvenile and adult |
|  |  |  | sT7aVVM004I19072 | 161713935 | Cabernet Sauvignon | Roots | 10 cm high plants grown in Magenta boxes |
|  |  |  | VRK350 | 48941440 | Vitis riparia | Bud | Paradormant |
|  |  |  | FAMU_USDA_FP_2359 | 51576500 | Vitis shuttleworthii | Entire tendril, leaves, bud, flowers | At blooming |
|  |  |  | sT7aVVM018B11048 | 161716735 | Cabernet Sauvignon | Roots | 10 cm high plants grown in Magenta boxes |
|  |  |  | VV_PEb04e11.b1 | 156728750 | Perlette | Bud | Mature |
|  |  |  | VRK350T7 | 48941441 | Vitis riparia | Bud | Paradormant |
|  |  |  | WIN025.TB24.1_O10 | 110361131 | Cabernet Sauvignon | Flower, leaf and root | Flower, pre-anthesis; leaf, fully expanded; root, produced by air-layering |
| *VvPDR21* | *VvABCG51* | GSVIVT01031378001 | VVG008B07_752925 | 71854950 | Cabernet Sauvignon | Cell suspension culture | |
|  |  |  | sT7aVVM015F06027 | 161717623 | Cabernet Sauvignon | Roots | 10 cm high plants grown in Magenta boxes |
|  |  |  | VRK350 | 48941440 | Vitis riparia | Bud | Paradormant |
|  |  |  | FAMU_USDA_FP_2359 | 51576500 | Vitis shuttleworthii | Entire tendril, leaves, bud, flowers | At blooming |
|  |  |  | VVB180D05_414233 | 32272004 | Chardonnay | Leaf | Juvenile and adult |
|  |  |  | VVB092B05_338961 | 30325459 | Chardonnay | Leaf | Juvenile and adult |
|  |  |  | VVG058B10_762241 | 71859608 | Cabernet Sauvignon | Cell suspension culture | |
|  |  |  | WIN0517.C21_N11 | 110374889 | Cabernet Sauvignon | Flower leaf and root | Flower, pre-anthesis; leaf, fully expanded; root, produced by air-layering |
|  |  |  | WIN025.TB24.1_O10 | 110361131 | Cabernet Sauvignon | Flower, leaf and root | Flower, pre-anthesis; leaf, fully expanded; root, produced by air-layering |
|  |  |  | sT7aVV01002X1F10 | 161721127 | Cabernet Sauvignon | Roots | 10 cm high plants grown in Magenta boxes |
|  |  |  | VRK350T7 | 48941441 | Vitis riparia | Bud | Paradormant |
|  |  |  | VVB072B06_334116 | 30324232 | Chardonnay | Leaf | Juvenile and adult |
|  |  |  | WIN0813.C21_K04 | 110396451 | Cabernet Sauvignon | Seed | Fruit set to maturity |
|  |  |  | VVG058B10_762241 | 71859608 | Cabernet Sauvignon | Cell suspension culture | |
| *VvPDR22* | *VvABCG52* | GSVIVT01031380001 | sT7aVVM015F06027 | 161717623 | Cabernet Sauvignon | Roots | 10 cm high plants grown in Magenta boxes |
|  |  |  | sT7aVVM018B11048 | 161716735 | Cabernet Sauvignon | Roots | 10 cm high plants grown in Magenta boxes |
|  |  |  | FAMU_USDA_FP_2359 | 51576500 | Vitis shuttleworthii | Entire tendril, leaves, bud, flowers | At blooming |
|  |  |  | FAMU_USDA_FP_00004 | 34994867 | Vitis shuttleworthii | Entire tendril, leaves, bud, flowers | At blooming |
|  |  |  | VVB072B06_334116 | 30324232 | Chardonnay | Leaf | Juvenile and adult |
|  |  |  | VVG058B10_762241 | 71859608 | Cabernet Sauvignon | Cell suspension culture | |
|  |  |  | WIN0813.C21_K04 | 110396451 | Cabernet Sauvignon | Seed | Fruit set to maturity |
|  |  |  | VRK350T7 | 48941441 | Vitis riparia | Bud | Paradormant |
|  |  |  | WIN1149.C21_L07 | 110427444 | Muscat Hamburg | Berry | Anthesis flower to prior to veraison |
|  |  |  | VV_PEb04e11.b1 | 156728750 | Perlette | Bud | Mature |
| *VvPDR23* | *VvABCG53* | GSVIVT01033804001 | sT7aVVM004I19072 | 161713935 | Cabernet Sauvignon | Roots | 10 cm high plants grown in Magenta boxes |
|  |  |  | FAMU_USDA_FP_00004 | 34994867 | Vitis shuttleworthii | Entire tendril, leaves, bud, flowers | At blooming |
|  |  |  | VRK350T7 | 48941441 | Vitis riparia | Bud | Paradormant |
|  |  |  | WIN0517.C21_N11 | 110374889 | Cabernet Sauvignon | Flower leaf and root | Flower, pre-anthesis; leaf, fully expanded; root, produced by air-layering |
|  |  |  | VVI139G10_605008 | 71876885 | Cabernet Sauvignon | Inflorescence including flowers | 12 - modified E-L system |
|  |  |  | VVH060G10_749061 | 71861652 | Cabernet Sauvignon | Nectary of flowers | 25 - modified E-L system |
|  |  |  | WIN0210.TB24.1_P19 | 110362519 | Cabernet Sauvignon | Flower, leaf and root | Flower, pre-anthesis; leaf, fully expanded; root, produced by air-layering |
|  |  |  | WIN1149.C21_L07 | 110427444 | Muscat Hamburg | Berry | Anthesis flower to prior to veraison |
|  |  |  | sT7aVVM018B11048 | 161716735 | Cabernet Sauvignon | Roots | 10 cm high plants grown in Magenta boxes |
|  |  |  | WIN025.TB24.1_O10 | 110361131 | Cabernet Sauvignon | Flower, leaf and root | Flower, pre-anthesis; leaf, fully expanded; root, produced by air-layering |
|  |  |  | FAMU_USDA_FP_2359 | 51576500 | Vitis shuttleworthii | Entire tendril, leaves, bud, flowers | At blooming |
|  |  |  | VVB180D05_414233 | 32272004 | Chardonnay | Leaf | Juvenile and adult |
|  |  |  | VVB045H10_324652 | 30321787 | Chardonnay | Leaf | Juvenile and adult |
| *VvPDR24* | *VvABCG54* | GSVIVT01034741001 | WIN1116.C21_C07 | 110416513 | Muscat Hamburg | Berry | Anthesis flower to prior to veraison |
|  |  |  | WIN0417.C21_P17 | 110369179 | Cabernet Sauvignon | Pericarp | Fruit set to maturity |
|  |  |  | sT7aVVM015F06027 | 161717623 | Cabernet Sauvignon | Roots | 10 cm high plants grown in Magenta boxes |
|  |  |  | VVL142C04_699050 | 71889657 | Cabernet Sauvignon | Fruit with seeds removed | Mixeded 36-38 - modified E-L system (Brix > 15) |
|  |  |  | WIN1149.C21_L07 | 110427444 | Muscat Hamburg | Berry | Anthesis flower to prior to veraison |
|  |  |  | VVG058B10_762241 | 71859608 | Cabernet Sauvignon | Cell suspension culture | |
|  |  |  | VRK350 | 48941440 | Vitis riparia | Bud | Paradormant |
|  |  |  | EST 17141 | 46909652 | Cabernet Sauvignon | Fruit skin | Green stage |
|  |  |  | VVL143B04_699196 | 71889730 | Cabernet Sauvignon | Fruit with seeds removed | Mixed 36-38 - modified E-L system (Brix > 15) |
|  |  |  | CAB10004_IIIa_Fa_E08 | 30253080 | Cabernet Sauvignon | Flower | Pre-bloom |
|  |  |  | VVC012B08_394327 | 30329097 | Chardonnay | Berries | Mixed; 8, 9, 11, 13, 15, 16 weeks daf |
|  |  |  | WIN0813.C21_K04 | 110396451 | Cabernet Sauvignon | Seed | Fruit set to maturity |
|  |  |  | VVB092B05_338961 | 30325459 | Chardonnay | Leaf | Juvenile and adult |
| *VvPDR25* | *VvABCG55* | GSVIVT01034745001 | FAMU_USDA_FP_2359 | 51576500 | Vitis shuttleworthii | Entire tendril, leaves, bud, flowers | At blooming |
|  |  |  | VVG058B10_762241 | 71859608 | Cabernet Sauvignon | Cell suspension culture | |
|  |  |  | WIN1149.C21_L07 | 110427444 | Muscat Hamburg | Berry | Anthesis flower to prior to veraison |
|  |  |  | EST 17141 | 46909652 | Cabernet Sauvignon | Fruit skin | Green stage |
|  |  |  | VVL142C04_699050 | 71889657 | Cabernet Sauvignon | Fruit with seeds removed | Mixed 36-38 - modified E-L system (Brix > 15) |
|  |  |  | sT7aVVM015F06027 | 161717623 | Cabernet Sauvignon | Roots | 10 cm high plants grown in Magenta boxes |
|  |  |  | VRK350 | 48941440 | Vitis riparia | Bud | Paradormant |
|  |  |  | WIN0813.C21_K04 | 110396451 | Cabernet Sauvignon | Seed | Fruit set to maturity |
|  |  |  | VVB092B05_338961 | 30325459 | Chardonnay | Leaf | Juvenile and adult |
|  |  |  | WIN1116.C21_C07 | 110416513 | Muscat Hamburg | Berry | Anthesis flower to prior to veraison |
|  |  |  | VVL143B04_699196 | 71889730 | Cabernet Sauvignon | Fruit with seeds removed | Mixed 36-38 - modified E-L system (Brix > 15) |
|  |  |  | WIN0417.C21_P17 | 110369179 | Cabernet Sauvignon | Pericarp | Fruit set to maturity |
|  |  |  | WIN1149.C21_L07 | 110427444 | Muscat Hamburg | Berry | Anthesis flower to prior to veraison |
|  |  |  | VRK350 | 48941440 | Vitis riparia | Bud | Paradormant |
| *VvPDR26* | *VvABCG56* | GSVIVT01034746001 | sT7aVVM004I19072 | 161713935 | Cabernet Sauvignon | Roots | 10 cm high plants grown in Magenta boxes |
|  |  |  | sT7aVVM015F06027 | 161717623 | Cabernet Sauvignon | Roots | 10 cm high plants grown in Magenta boxes |
|  |  |  | VV_PEb04e11.b1 | 156728750 | Perlette | Bud | Mature |
|  |  |  | VRK350T7 | 48941441 | Vitis riparia | Bud | Paradormant |
|  |  |  | VRK350 | 48941440 | Vitis riparia | Bud | Paradormant |
|  |  |  | VVG058B10_762241 | 71859608 | Cabernet Sauvignon | Cell suspension culture | |
|  |  |  | VVB072B06_334116 | 30324232 | Chardonnay | Leaf | Juvenile and adult |
|  |  |  | WIN1149.C21_L07 | 110427444 | Muscat Hamburg | Berry | Anthesis flower to prior to veraison |
|  |  |  | WIN1116.C21_C07 | 110416513 | Muscat Hamburg | Berry | Anthesis flower to prior to veraison |
|  |  |  | VVL143B04_699196 | 71889730 | Cabernet Sauvignon | Fruit with seeds removed | Mixed 36-38 - modified E-L system (Brix > 15) |
|  |  |  | VVL142C04_699050 | 71889657 | Cabernet Sauvignon | Fruit with seeds removed | Mixed 36-38 - modified E-L system (Brix > 15) |
|  |  |  | FAMU_USDA_FP_2359 | 51576500 | Vitis shuttleworthii | Entire tendril, leaves, bud, flowers | At blooming |
|  |  |  | WIN0417.C21_P17 | 110369179 | Cabernet Sauvignon | Pericarp | Fruit set to maturity |
|  |  |  | VVC012B08_394327 | 30329097 | Chardonnay | Berries | Mixed; 8, 9, 11, 13, 15, 16 weeks daf |
|  |  |  | EST 17141 | 46909652 | Cabernet Sauvignon | Fruit skin | Green stage |
| *VvPDR27* | *VvABCG57* | GSVIVT01034748001 | VRK350 | 48941440 | Vitis riparia | Bud | Paradormant |
|  |  |  | VVG058B10_762241 | 71859608 | Cabernet Sauvignon | Cell suspension culture | |
|  |  |  | WIN1149.C21_L07 | 110427444 | Muscat Hamburg | Berry | Anthesis flower to prior to veraison |
|  |  |  | sT7aVVM015F06027 | 161717623 | Cabernet Sauvignon | Roots | 10 cm high plants grown in Magenta boxes |
|  |  |  | VRK350T7 | 48941441 | Vitis riparia | Bud | Paradormant |
|  |  |  | VVC012B08_394327 | 30329097 | Chardonnay | Berries | Mixed; 8, 9, 11, 13, 15, 16 weeks daf |
|  |  |  | WIN1116.C21_C07 | 110416513 | Muscat Hamburg | Berry | Anthesis flower to prior to veraison |
|  |  |  | VVL142C04_699050 | 71889657 | Cabernet Sauvignon | Fruit with seeds removed | ixed 36-38 - modified E-L system (Brix > 15) |
|  |  |  | WIN0417.C21_P17 | 110369179 | Cabernet Sauvignon | Pericarp | Fruit set to maturity |
|  |  |  | VV_PEb04e11.b1 | 156728750 | Perlette | Bud | Mature |
|  |  |  | VVB092B05_338961 | 30325459 | Chardonnay | Leaf | Juvenile and adult |
|  |  |  | sT7aVVM015F06027 | 161717623 | Cabernet Sauvignon | Roots | 10 cm high plants grown in Magenta boxes |
|  |  |  | VVL143B04_699196 | 71889730 | Cabernet Sauvignon | Fruit with seeds removed | Mixed 36-38 - modified E-L system (Brix > 15) |
|  |  |  | CN545197 | 110429813 | Muscat Hamburg | Fruit skin | Green stage |
| *VvPDR28* | *VvABCG58* | GSVIVT01035715001 | FAMU_USDA_FP_7993 | 51582134 | Vitis shuttleworthii | Entire tendril, leaves, bud, flowers | At blooming |
|  |  |  | VV_PEb04e11.b1 | 156728750 | Perlette | Bud | Mature |
|  |  |  | WIN117.C21_O11 | 110429813 | Muscat Hamburg | Berry | Anthesis flower to prior to veraison |
|  |  |  | sT7aVV01002X1F10 | 161721127 | Cabernet Sauvignon | Roots | 10 cm high plants grown in Magenta boxes |
|  |  |  | sT7aVVM015F06027 | 161717623 | Cabernet Sauvignon | Roots | 10 cm high plants grown in Magenta boxes |
|  |  |  | CSECS096H10_POSn0036 | 34416693 | Cabernet Sauvignon | Fruit with seeds removed | 36 - modified E-L system |
|  |  |  | sT7aVVM_AER28G08 | 161711104 | Cabernet Sauvignon | Roots | 10 cm high plants grown in Magenta boxes |
|  |  |  | VV_PEb04e11.g1 | 156732345 | Perlette | Bud | Mature |
|  |  |  | FAMU_USDA_FP_7182 | 51581323 | Vitis shuttleworthii | Entire tendril, leaves, bud, flowers | At blooming |
|  |  |  | WIN0574.C21_D24 | 110391620 | Cabernet Sauvignon | Flower leaf and root | Flower, pre-anthesis; leaf, fully expanded; root, produced by air-layering |
|  |  |  | WIN058.C21_K05 | 110374394 | Cabernet Sauvignon | Flower leaf and root | Flower, pre-anthesis; leaf, fully expanded; root, produced by air-layering |
|  |  |  | VVG058B10_762241 | 71859608 | Cabernet Sauvignon | Cell suspension culture | |
|  |  |  | sT7aVVM_AER65G03 | 161709855 | Cabernet Sauvignon | Roots | 10 cm high plants grown in Magenta boxes |
| *VvPDR29* | *VvABCG59* | GSVIVT01035780001 | sT7aVVM_AER28G08 | 161711104 | Cabernet Sauvignon | Roots | 10 cm high plants grown in Magenta boxes |
|  |  |  | sT7aVVM015B11048 | 161716494 | Cabernet Sauvignon | Roots | 10 cm high plants grown in Magenta boxes |
|  |  |  | WIN053.C21_C06 | 110371208 | Cabernet Sauvignon | Flower leaf and root | Flower, pre-anthesis; leaf, fully expanded; root, produced by air-layering |
|  |  |  | CAP0004_IIIF_H12 | 34548882 | Cabernet Sauvignon | Petiole | Onset of Veraison (berry softening) |
|  |  |  | CAB10004_IIIa_Fa_E08 | 30253080 | Cabernet Sauvignon | Flower - pre-bloom | Pre-bloom |
|  |  |  | VVH060G10_749061 | 71861652 | Cabernet Sauvignon | Nectary of flowers | 25 - modified E-L system |
| *VvPDR30* | *VvABCG60* | GSVIVT01035784001 | CAB10004_IIIa_Fa_E08 | 30253080 | Cabernet Sauvignon | Flower | Pre-bloom |
|  |  |  | sT7aVVM015B11048 | 161716494 | Cabernet Sauvignon | Roots | 10 cm high plants grown in Magenta boxes |
|  |  |  | WIN053.C21_C06 | 110371208 | Cabernet Sauvignon | Flower leaf and root | Flower, pre-anthesis; leaf, fully expanded; root, produced by air-layering |
|  |  |  | WIN025.TB24.1_O10 | 110361131 | Cabernet Sauvignon | Flower, leaf and root | Flower, pre-anthesis; leaf, fully expanded; root, produced by air-layering |
|  |  |  | sT7aVVM027K14053 | 161721191 | Cabernet Sauvignon | Roots | 10 cm high plants grown in Magenta boxes |
|  |  |  | VVH060G10_749061 | 71861652 | Cabernet Sauvignon | Nectary of flowers | 25 - modified E-L system |
|  |  |  | sT7aVVM002M15052 | 161712783 | Cabernet Sauvignon | Roots | 10 cm high plants grown in Magenta boxes |
|  |  |  | VV_PEb09f09.b1 | 156729588 | Perlette | Bud | Mature |
|  |  |  | VVG008B07_752925 | 71854950 | Cabernet Sauvignon | Cell suspension culture | |
|  |  |  | VV_PEb04e11.g1 | 156732345 | Perlette | Bud | Mature |
|  |  |  | CAP0004_IIIF_H12 | 34548882 | Cabernet Sauvignon | Petiole | Onset of Veraison (berry softening) |
| *VvPDR31* | *VvABCG61* | GSVIVT01035785001 |  |  |  |  |  |
| *VvPDR32* | *VvABCG62* | GSVIVT01035786001 | sT7aVVM004I19072 | 161713935 | Cabernet Sauvignon | Roots | 10 cm high plants grown in Magenta boxes |
|  |  |  | sT7aVVM027K14053 | 161721191 | Cabernet Sauvignon | Roots | 10 cm high plants grown in Magenta boxes |
|  |  |  | VV_PEb04e11.g1 | 156732345 | Perlette | Bud | Mature |
|  |  |  | VV_PEb11g06.b1 | 156729932 | Perlette | Bud | Mature |
|  |  |  | VV_PEb04e11.b1 | 156728750 | Perlette | Bud | Mature |
|  |  |  | S9B03542 | 110722533 | Thompson-seedless | Berry | Ripening berries |
|  |  |  | CGF1000665_A04 | 33406125 | Cabernet Sauvignon | Stem | Pre-bloom (10-11 days before bloom) |
|  |  |  | VVL144F01_699442 | 71889853 | Cabernet Sauvignon | Fruit with seeds removed | Mixed 36-38 - modified E-L system (Brix > 15) |
|  |  |  | CAB10004_IIIa_Fa_E08 | 30253080 | Cabernet Sauvignon | Flower - pre-bloom | Pre-bloom |
| *VvPDR33* | *VvABCG63* | GSVIVT01036184001 | sT7aVVM018B11048 | 161716735 | Cabernet Sauvignon | Roots | 10 cm high plants grown in Magenta boxes |
|  |  |  | sT7aVVM015F06027 | 161717623 | Cabernet Sauvignon | Roots | 10 cm high plants grown in Magenta boxes |
|  |  |  | WIN0813.C21_K04 | 110396451 | Cabernet Sauvignon | Seed | Fruit set to maturity |
|  |  |  | VVB180D05_414233 | 32272004 | Chardonnay | Leaf | Juvenile and adult |
|  |  |  | VVB092B05_338961 | 30325459 | Chardonnay | Leaf | Juvenile and adult |
|  |  |  | VVB072B06_334116 | 30324232 | Chardonnay | Leaf | Juvenile and adult |
|  |  |  | sT7aVVM004I19072 | 161713935 | Cabernet Sauvignon | Roots | 10 cm high plants grown in Magenta boxes |
|  |  |  | sT7aVVM015B11048 | 161716494 | Cabernet Sauvignon | Roots | 10 cm high plants grown in Magenta boxes |
|  |  |  | VVL143B04_699196 | 71889730 | Cabernet Sauvignon | Fruit with seeds removed | Mixed 36-38 - modified E-L system (Brix > 15) |
|  |  |  | WIN1149.C21_L07 | 110427444 | Muscat Hamburg | Berry | Anthesis flower to prior to veraison |
|  |  |  | WIN025.TB24.1_O10 | 110361131 | Cabernet Sauvignon | Flower, leaf and root | Flower, pre-anthesis; leaf, fully expanded; root, produced by air-layering |
|  |  |  | FAMU_USDA_FP_2359 | 51576500 | Vitis shuttleworthii | Entire tendril, leaves, bud, flowers | At blooming |
|  |  |  | VVB104F09_341871 | 30326914 | Chardonnay | Leaf | Juvenile and adult |
|  |  |  | VVB076D03_333788 | 30324068 | Chardonnay | Leaf | Juvenile and adult |
|  |  |  | VVB089H03_338543 | 30325250 | Chardonnay | Leaf | Juvenile and adult |
|  |  |  | VVB073B11_334314 | 30324331 | Chardonnay | Leaf | Juvenile and adult |
|  |  |  | VVB198E12_431705 | 32248400 | Chardonnay | Leaf | Juvenile and adult |
|  |  |  | VVB170H03_412545 | 32271160 | Chardonnay | Leaf | Juvenile and adult |
|  |  |  | VVB085C02_335686 | 30325017 | Chardonnay | Leaf | Juvenile and adult |
