# Supplementary material for: Whole-Genome Survey of the Putative ATP-Binding Cassette Transporter Family Genes in Vitis vinifera
Source: PLoS One. 2013 Nov 11;8(11):e78860. doi: 10.1371/journal.pone.0078860 (PMC3823996; doi:10.1371/journal.pone.0078860)
Supplement: Table S10 — Expressed sequence taqs (ESTs) identified for ABCI (NAP) subfamily in Vitis vinifera . The protein name, Vitis proteome 12x ID, GenBank ID, EST name, cultivar/tissue type, and development stage are given for each gene. (DOC) [file pone.0078860.s010.doc]

**Table S10.** Expressed sequence taqs (ESTs) identified for ABCI (NAP) subfamily in *Vitis vinifera*.The protein name, *Vitis* proteome 12x ID, GenBank ID, EST name, cultivar/tissue type, and development stage are given for each gene.

| **Name** | | ***Vitis* 12X ID** | **EST Name** | **GenBank ID** | **Species/Cultivar** | **Tissue Type** | **Development Stage** |
| --- | --- | --- | --- | --- | --- | --- | --- |
| *VvNAP1* | *VvABCI1* | GSVIVT01010853001 | CSECS168B08_POSu0038 | 83275622 | Cabernet Sauvignon | Fruit with seeds removed | 38 - modified E-L system |
|  |  |  | sT7aVVM007E13059 | 161714282 | Cabernet Sauvignon | Roots | 10 cm high plants grown in Magenta boxes |
|  |  |  | sT7aVVM006M03004 | 161713337 | Cabernet Sauvignon | Roots | 10 cm high plants grown in Magenta boxes |
|  |  |  | sT7aVVM_AER30E01 | 161708195 | Cabernet Sauvignon | Roots | 10 cm high plants grown in Magenta boxes |
|  |  |  | FAMU_USDA_FP_3982 | 51578123 | Vitis shuttleworthii | Entire tendril, leaves, bud, flowers | At blooming |
|  |  |  | FAMU_USDA_FP_4596 | 51578737 | Vitis shuttleworthii | Entire tendril, leaves, bud, flowers | At blooming |
|  |  |  | VV_PEc12E01.f.ab1 | 156735135 | Perlette | Bud | Mature |
|  |  |  | VV_PEc12E01.r.ab1 | 156735143 | Perlette | Bud | Mature |
|  |  |  | USDA_FP_132161 | 47090805 | Vitis shuttleworthii | Entire tendril, leaves, bud, flowers | At blooming |
|  |  |  | BACCA01_001594 | 37184898 | Pinot Noir | Berry | Veraison |
|  |  |  | VVG052A01_761101 | 71859038 | Cabernet Sauvignon | Cell suspension culture | |
|  |  |  | VVI082E08_595424 | 71873737 | Cabernet Sauvignon | Inflorescence including flowers | 12 - modified E-L system |
|  |  |  | VVD002B04_127120 | 27586782 | Chardonnay | Berries | Mixed; 8, 9, 11, 13, 15, 16 weeks daf |
|  |  |  | VVG057H06_762189 | 71859582 | Cabernet Sauvignon | Cell suspension culture | |
|  |  |  | VVG037A12_758331 | 71857653 | Cabernet Sauvignon | Cell suspension culture | |
|  |  |  | S2B10489 | 110699996 | Thompson-seedless | Bud |  |
|  |  |  | S8B03160 | 110718770 | Thompson-seedless | Fruit | Veraison |
|  |  |  | SBB06004 | 110727461 | Thompson-seedless | Inflorescence |  |
|  |  |  | S1G05612 | 110698764 | Thompson-seedless | Fruit and flower |  |
|  |  |  | S5B05671 | 110708403 | Thompson-seedless | Fruit | Fruits 7-9 mm |
|  |  |  | SCB06582 | 110733021 | Thompson-seedless | Inflorescence |  |
|  |  |  | INFIO01_000175 | 37190042 | Regent | Inflorescence | Young inflorescence before flowering |
|  |  |  | WIN0824.C21_F07 | 110400676 | Cabernet Sauvignon | Seed | Fruit set to maturity |
|  |  |  | CAB40005_IIIb_Fb_F07 | 30301261 | Cabernet Sauvignon | Berry | Berry on stage II, 9 mm |
| *VvNAP2* | *VvABCI2* | GSVIVT01012742001 | S9B01743 | 110721275 | Thompson-seedless | Berry |  |
|  |  |  | CA12LI01IIF-G8 | 26256882 | Cabernet Sauvignon | Leaf | Late season sample |
|  |  |  | VVB154B01_409473 | 32269624 | Chardonnay | leaf | juvenile and adult |
|  |  |  | CAP0003_IF_E10 | 34551039 | Cabernet Sauvignon | Petiole | Onset of Veraison (berry softening) |
|  |  |  | VVB192D06_430573 | 32247834 | Chardonnay | Leaf | juvenile and adult |
|  |  |  | VVI188A12_613170 | 77583428 | Cabernet Sauvignon | Inflorescence including flowers | 12 - modified E-L system |
|  |  |  | CAP0003_IR_E10 | 34548247 | Cabernet Sauvignon | Petiole | Onset of Veraison (berry softening) |
|  |  |  | WIN1140.C21_F04 | 110424341 | Muscat Hamburg | Berry | Anthesis flower to prior to veraison |
|  |  |  | WIN017.C21_A07 | 110358353 | Cabernet Sauvignon | Pericarp | Fruit set to maturity |
|  |  |  | WIN1145.C21_L05 | 110426123 | Muscat Hamburg | Berry | Anthesis flower to prior to veraison |
|  |  |  | EST 3807 | 22009835 | Shiraz | Fruit | veraison stage |
|  |  |  | WIN0538.C21_E14 | 110380985 | Cabernet Sauvignon | Flower, leaf and root | Flower, pre-anthesis; leaf, fully expanded; root, produced by air-layering |
|  |  |  | VVL101G07_691982 | 71886123 | Cabernet Sauvignon | Fruit with seeds removed | mixed 36-38 - modified E-L system (Brix > 15) |
|  |  |  | VVL048H02_682944 | 71881604 | Cabernet Sauvignon | Fruit with seeds removed | mixed 36-38 - modified E-L system (Brix > 15) |
|  |  |  | VVL067C04_686142 | 71883203 | Cabernet Sauvignon | Fruit with seeds removed | mixed 36-38 - modified E-L system (Brix > 15) |
|  |  |  | VVL125A12_696060 | 71888162 | Cabernet Sauvignon | Fruit with seeds removed | mixed 36-38 - modified E-L system (Brix > 15) |
|  |  |  | FAMU_USDA_FP_6065 | 51580206 | Vitis shuttleworthii | Entire tendril, leaves, bud, flowers | At blooming |
|  |  |  | sT7aVVM008M11036 | 161713494 | Cabernet Sauvignon | Roots | 10 cm high plants grown in Magenta boxes |
| *VvNAP3* | *VvABCI3* | GSVIVT01013180001 | S8B04128 | 110720486 | Thompson-seedless | Fruit | Veraison |
|  |  |  | EST 3275 | 22009303 | Shiraz | Fruit | Veraison stage |
|  |  |  | WIN0415.C21_E06 | 110368110 | Cabernet Sauvignon | Pericarp | Fruit set to maturity |
|  |  |  | WIN0561.C21_G02 | 110389483 | Cabernet Sauvignon | Flower, leaf and root | Flower, pre-anthesis; leaf, fully expanded; root, produced by air-layering |
| *VvNAP4* | *VvABCI4* | GSVIVT01017866001 | sT7aVVM011M08020 | 161718803 | Cabernet Sauvignon | Roots | 10 cm high plants grown in Magenta boxes |
| *VvNAP5* | *VvABCI5* | GSVIVT01019131001 | FAMU_USDA_FP_2614 | 51576755 | Vitis shuttleworthii | Entire tendril, leaves, bud, flowers | At blooming |
|  |  |  | sT7aVVM_AER52D02 | 161710939 | Cabernet Sauvignon | roots | 10 cm high plants grown in Magenta boxes |
|  |  |  | VVD075E05_354193 | 30136442 | Chardonnay | Berries | mixed; 8, 9, 11, 13, 15, 16 weeks daf |
|  |  |  | CSECS131C05_CELu0001 | 45770421 | Cabernet Sauvignon | Cell suspension culture | |
|  |  |  | VV_PEa20e01.g1 | 156725118 | Perlette | Bud | Mature |
|  |  |  | WIN026.TB24.1_E13 | 110361223 | Cabernet Sauvignon | Flower, leaf and root | Flower, pre-anthesis; leaf, fully expanded; root, produced by air-layering |
| *VvNAP6* | *VvABCI6* | GSVIVT01031286001 | CA23EI03IIRb_H11 | 28958869 | Cabernet Sauvignon | Leaf | Mid-season leaf material, collected July 25, 2001 |
|  |  |  | sT7aVVM015L24086 | 161719484 | Cabernet Sauvignon | roots | 10 cm high plants grown in Magenta boxes |
|  |  |  | VVD115F11_371443 | 30131552 | Chardonnay | Berries | mixed; 8, 9, 11, 13, 15, 16 weeks daf |
|  |  |  | VVC042D03_141398 | 27584476 | Chardonnay | Berries | mixed; 8, 9, 11, 13, 15, 16 weeks daf |
|  |  |  | sT7aVVM_AER20H11 | 161709972 | Cabernet Sauvignon | Roots | 10 cm high plants grown in Magenta boxes |
|  |  |  | VV_PEb07b01.g1 | 156729155 | Perlette | Bud | Mature |
|  |  |  | WIN101.C21_G21 | 110404860 | Muscat Hamburg | Pericarp | Fruit set to maturity |
|  |  |  | VVA018G12_54279 | 18459114 | Chardonnay | Leaf | Juvenile and adult |
|  |  |  | WIN0542.C21_D01 | 110383257 | Cabernet Sauvignon | Flower, leaf and root | Flower, pre-anthesis; leaf, fully expanded; root, produced by air-layering |
|  |  |  | CGF1000749_D08 | 33405925 | Cabernet Sauvignon | Stem | Pre-bloom (10-11 days before bloom) |
|  |  |  | CA12LIO2IIIR_C08 | 26257979 | Cabernet Sauvignon | Leaf | Late season sample |
|  |  |  | VV_PEb07b01.b1 | 156732414 | Perlette | Bud | Mature |
|  |  |  | WIN0542.C21_L04 | 110383413 | Cabernet Sauvignon | Flower, leaf and root | Flower, pre-anthesis; leaf, fully expanded; root, produced by air-layering |
|  |  |  | WIN022.C21_J04 | 110360559 | Cabernet Sauvignon | Flower, leaf and root | Flower, pre-anthesis; leaf, fully expanded; root, produced by air-layering |
|  |  |  | VVG027H09_756623 | 71856799 | Cabernet Sauvignon | Cell suspension culture | |
|  |  |  | CA23EI03IIFb_H11 | 28959349 | Cabernet Sauvignon | Leaf | Mid-season leaf material, collected July 25, 2001 |
|  |  |  | VVG027H09_756623 | 71856799 | Cabernet Sauvignon | Cell suspension culture | |
