# Supplementary material for: Whole-Genome Survey of the Putative ATP-Binding Cassette Transporter Family Genes in Vitis vinifera
Source: PLoS One. 2013 Nov 11;8(11):e78860. doi: 10.1371/journal.pone.0078860 (PMC3823996; doi:10.1371/journal.pone.0078860)
Supplement: Table S11 — Expressed sequence taqs (ESTs) identified for SMC proteins in Vitis vinifera . The protein name, Vitis proteome 12x ID, GenBank ID, EST name, cultivar/tissue type, and development stage are given for each gene. (DOC) [file pone.0078860.s011.doc]

**Table S11.** Expressed sequence taqs (ESTs) identified for SMC proteins in *Vitis vinifera*.The protein name, *Vitis* proteome 12x ID, GenBank ID, EST name, cultivar/tissue type, and development stage are given for each gene.

| **Name** | | ***Vitis* 12X ID** | **EST Name** | **GenBank ID** | **Species/Cultivar** | **Tissue Type** | **Development Stage** |
| --- | --- | --- | --- | --- | --- | --- | --- |
| *VvSMC1* |  | GSVIVT01011408001 | CAB20005_IVb_Rb_C06 | 33404339 | Cabernet Sauvignon | Flower - Bloom | Bloom |
| *VvSMC2* |  | GSVIVT01018715001 | WIN1148.C21_O01 | 122692151 | Muscat Hamburg | Berry | Anthesis flower to prior to veraison |
|  |  |  | WIN1133.C21_D13 | 122692026 | Muscat Hamburg | Berry | Anthesis flower to prior to veraison |
|  |  |  | GEMMA01_001004 | 37188144 | Pinot Noir | Bud | Bud swelling |
| *VvSMC3* |  |  | VV_PEa16c05.g1 | 156726791 | Perlette | Bud | Mature |
|  |  |  | VV_PEa016c05.b1 | 156723722 | Perlette | Bud | Mature |
|  |  |  | EST 9306 | 32460223 | Chardonnay | Fruit without seeds | Green stage |
|  |  |  | EST 8278 | 22007524 | Cabernet Sauvignon | Fruit without seeds | Harvest - 15,5 weeks post-flowering |
|  |  |  | WIN1032.C21_N19 | 110411184 | Muscat Hamburg | Pericarp | Fruit set to maturity |
|  |  |  | WIN063.C21_L05 | 110393010 | Cabernet Sauvignon | Seed | Fruit set to maturity |
|  |  |  | CA32EN0001_IIaR_F07 | 28962383 | Cabernet Sauvignon | Leaf | Mid-season leaf material, collected July 25, 2001 |
|  |  |  | S9B00725 | 110721065 | Thompson-seedless | Berry | Ripening Berries |
|  |  |  | CA32EN0001_IIaF_F07 | 29784864 | Cabernet Sauvignon | Leaf | Mid-season leaf material, collected July 25, 2001 |
